# Surprisal From Language Models Can Predict ERPs in Processing Predicate-Argument Structures Only if Enriched by an Agent Preference Principle

**DOI:** 10.1162/nol_a_00121

**Published:** 2024-04-01

**Authors:** Eva Huber, Sebastian Sauppe, Arrate Isasi-Isasmendi, Ina Bornkessel-Schlesewsky, Paola Merlo, Balthasar Bickel

**Affiliations:** Department of Comparative Language Science, University of Zurich, Zurich, Switzerland; Center for the Interdisciplinary Study of Language Evolution, University of Zurich, Zurich, Switzerland; Department of Psychology, University of Zurich, Zurich, Switzerland; Cognitive Neuroscience Laboratory, Australian Research Centre for Interactive and Virtual Environments, University of South Australia, Adelaide, Australia; Department of Linguistics, University of Geneva, Geneva, Switzerland; University Center for Computer Science, University of Geneva, Geneva, Switzerland

**Keywords:** artificial neural networks, computational modeling, event cognition, ERP, sentence processing, surprisal, large language models (LLMs)

## Abstract

Language models based on artificial neural networks increasingly capture key aspects of how humans process sentences. Most notably, model-based surprisals predict event-related potentials such as N400 amplitudes during parsing. Assuming that these models represent realistic estimates of human linguistic experience, their success in modeling language processing raises the possibility that the human processing system relies on no other principles than the general architecture of language models and on sufficient linguistic input. Here, we test this hypothesis on N400 effects observed during the processing of verb-final sentences in German, Basque, and Hindi. By stacking Bayesian generalised additive models, we show that, in each language, N400 amplitudes and topographies in the region of the verb are best predicted when model-based surprisals are complemented by an Agent Preference principle that transiently interprets initial role-ambiguous noun phrases as agents, leading to reanalysis when this interpretation fails. Our findings demonstrate the need for this principle independently of usage frequencies and structural differences between languages. The principle has an unequal force, however. Compared to surprisal, its effect is weakest in German, stronger in Hindi, and still stronger in Basque. This gradient is correlated with the extent to which grammars allow unmarked NPs to be patients, a structural feature that boosts reanalysis effects. We conclude that language models gain more neurobiological plausibility by incorporating an Agent Preference. Conversely, theories of human processing profit from incorporating surprisal estimates in addition to principles like the Agent Preference, which arguably have distinct evolutionary roots.

## INTRODUCTION

The brain processes information through an incremental and probabilistic mechanism of updating models of the world (Clark, 2013; Friston, 2010). Over the past twenty years, evidence has accumulated that, in the case of human language, this processing mechanism is largely guided by the preceding linguistic context and previous experience with the statistical distributions of linguistic structure (Hale, 2001; Levy, 2008) and units (Frank, Otten, et al., 2013). In particular, models of such distributions based on artificial neural networks have been remarkably successful at predicting electrophysiological (Frank et al., 2015; Goldstein et al., 2022; Michaelov et al., 2021; Szewczyk & Federmeier, 2022) and fMRI-BOLD (Brennan et al., 2020; Caucheteux & King, 2021; Henderson et al., 2016; Hosseini et al., 2022; Lopopolo et al., 2017; Schrimpf et al., 2020; Willems et al., 2016) responses during language processing.

However, it remains an unresolved question to what extent linguistic processing is also directly guided by independent neurobiological constraints, such as the evolutionarily inherited architecture of neural feedback loops (Cisek, 2022), sensory sampling frequencies (Friston, 2010; Ramstead et al., 2018), bodily states (Foglia & Wilson, 2013), prototypical cognitive event schemata (Bornkessel-Schlesewsky & Schlesewsky, 2009), or nonlinguistic knowledge and behavioural goals (Su et al., 2023). Is the probabilistic linguistic information that is captured by artificial neural network models sufficient to characterise language processing in humans?

Here, we seek to shed light on this question by formalising probabilistic linguistic information in terms of surprisal theory (Gibson et al., 2019; Hale, 2001; Levy, 2008) and neurobiological constraints in the form of what has been called a general *Agent Preference* (Bornkessel-Schlesewsky & Schlesewsky, 2020; V. A. D. Wilson et al., 2022). To this end, we turn to the N400 event-related potential (ERP) component, which has been prominently associated with the processing of probabilistic information and, accordingly, can be viewed as an indicator of how this type of information is processed in real time (Kuperberg & Jaeger, 2016). The N400 is an ERP component peaking around 400 ms post onset of a critical stimulus and is found, among other areas of cognition, in sentence comprehension, where it has been linked to processing difficulties of various types (cf. Kuperberg & Jaeger, 2016; Kutas & Federmeier, 2011, for reviews).

We specifically focus on the processing of predicate-argument structures as illustrated in the German Example 1, where a more pronounced N400 amplitude has been found with stimuli such as 1b compared to stimuli such as 1a. In the following, we will refer to this phenomenon as the *Predicate N400*, because it relates to the integration of a predicate (typically a verb) with its arguments (typically, noun phrases [NPs]) at the end of a sentence.

(1)  a. … dass Julia          alle          grüßt.
     … that Julia.SG.NOM/ACC/DAT everyone.PL.NOM/ACC/DAT greet.SG
     … ‘that Julia greets them all.’
    b. … dass Julia          alle          grüßen.
     … that Julia.SG.NOM/ACC/DAT everyone.PL.NOM/ACC/DAT greet.PL
     …‘that they all greet Julia.’

The Predicate N400 has been interpreted as reflecting a general Agent Preference. We expand on this in what follows and then turn to a possible alternative interpretation in terms of Surprisal Theory.

### The Predicate N400 as the Reflex of an Agent Preference

The Predicate N400 has been interpreted as evidence of semantic role reanalysis. Under this view, comprehenders transiently interpret a locally ambiguous initial NP (e.g., *Julia* in Example 1) as the agent of the verb. An N400 occurs with 1b because the initial NP is disambiguated to be a patient rather than an agent by *grüßen* “greet (plural).” Thus, the human parser arguably prefers initial unmarked NPs to be agents (as opposed to patients) at the level of proto-roles (Bickel, 2011; Dowty, 1991; Primus, 1999) or macro-roles (Van Valin, 2001; Van Valin & Foley, 1980). In other words, the parser expects these NPs to accumulate the most agent properties, such as “volitional,” “sentient,” “causing an event,” or “independently existing” in their event semantics. An alternative way of capturing this is in terms of a preference for initial subjects (Bader & Meng, 1999; Fanselow et al., 1999; Frazier & Flores d’Arcais, 1989; Hemforth et al., 1993), which leads to the same result as long as the sentences are transitive and in active voice (but see Bornkessel et al., 2003, for evidence that the preference operates in terms of semantic roles rather than syntactic functions).

These effects have been demonstrated in German (Haupt et al., 2008), Basque (Erdocia et al., 2009), and Austrian Sign Language (Krebs et al., 2018), using transitive stimuli of the kind illustrated by Example 1. A study on Swedish found the same effect with a slightly different design in which the second NP in a [NP V NP] structure served as the disambiguating region (Hörberg et al., 2013).

Another experimental design was used in Turkish (Demiral et al., 2008), Hindi (Bickel et al., 2015), Chinese (Wang et al., 2009), and Äiwoo (an Oceanic language, Sauppe et al., 2023). In these studies, an initial ambiguous NP was subsequently disambiguated to an agent or a patient by the verb, i.e., in [NP V] structures. Because of frequent omission of agents and word order variation, a [Patient Verb] interpretation of the structure is very probable in these languages. In Hindi, this trend is further strengthened by the fact that agents are marked with ergative case in the perfective aspect, increasing the frequency of unmarked NPs as patients. In Äiwoo, the trend is even stronger, because the syntax of the language builds on a basic object-verb-subject (OVS), that is, patient-initial, order (Næss, 2015, 2021). However, in all four languages, an N400 was found when the unmarked NP in an [NP V] sequence was disambiguated to a patient.

While these studies relied on transitive sentences, experiments on Basque also revealed an N400 for a disambiguation towards the patient role with intransitive verbs (e.g., “The boy fell” as opposed to “The boy danced”; Isasi-Isasmendi et al., 2024). This suggests that the Agent Preference holds independently of transitivity.

The Agent Preference is not limited to animate NPs but has been shown to generalise to inanimate NPs in Chinese, Turkish, and Hindi. Only two exceptions to this generalisation are known. First, the effect was reversed in [NP_inanimate_ NP V] sentences in Chinese, arguably because here an initial agent reading requires two fronted NPs, which is an overly complex structure with strong contextual constraints (Wang et al., 2012). Second, [NP_inanimate_ V NP] sentences reversed the effect in Äiwoo, arguably because the inanimate NP further strengthens its syntactic default interpretation as a patient in this language. This seems to override the Agent Preference observed for human referent NPs in Äiwoo (Sauppe et al., 2023).

### The Predicate N400 as the Reflex of Linguistic Surprisal

Previous research has not examined to what extent the Predicate N400 effect could alternatively be explained by the human experience with probabilistic and contextual information of incoming words. We consider this possibility as part of the larger framework known as *Surprisal Theory* (Hale, 2001; Levy, 2008). According to this theory, the human parser assigns a probability distribution to possible continuations. This is chiefly formalised in terms of *linguistic surprisal*, the logarithm of the inverse probability of a word given its preceding context. Alongside other probabilistic measures such as entropy reduction or linear word probabilities, linguistic surprisal mirrors some kind of graded prediction or expectation (Armeni et al., 2017).

The gradedness of the measure parallels the theoretical models of the N400 effect in which the negative amplitude of the negative ERP component is assumed to mirror precision-weighted prediction errors (Bornkessel-Schlesewsky & Schlesewsky, 2019). Similarly, it is consistent with models that simulate the N400 as a change evoked by the implicit and probabilistic meaning representation of an incoming stimulus (Lopopolo & Rabovsky, 2021; Rabovsky et al., 2018).

Surprisal Theory has taken different forms through the years. Earlier work uses surprisal as a linking function between the predictions of any theoretical model with the neurophysiological or behavioural signals (Hale, 2001; Levy, 2008). More recent work eschews built-in knowledge of syntactic structures and estimates linguistic surprisal with language models based on artificial neural networks. These networks model the distribution of words in context and are constrained only by their general architecture of information flow, and not by specifically linguistic knowledge. Surprisal from such language models thus estimates the predictability of words in context rather than in linguistic structures (e.g., the probability of a verb phrase projecting a noun phrase instead of a complementiser phrase). The more recent approach of Surprisal Theory thus offers a parsimonious account of the precision-weighted prediction errors or changes in probability that the N400 is thought to reflect. If successful, this version of Surprisal Theory would recast the N400 as purely driven by usage and whatever linguistic structures can be estimated from usage, in the context of the specific artificial neural network architecture (Hewitt & Manning, 2019).

Indeed, many studies converge in finding wide-ranging similarities between such models and human processing behavior in, for example, the processing of island constraints (Wilcox et al., 2023), long-distance agreement concord (Gulordava et al., 2018), and garden path effects (Futrell et al., 2019). Intriguingly, recent work has shown that model-based linguistic surprisal can accurately predict electroencephalogram (EEG) amplitudes (Frank et al., 2015; Michaelov et al., 2021; Szewczyk & Federmeier, 2022), reading times (Aurnhammer & Frank, 2019; Brothers & Kuperberg, 2021; Frank, Monsalve, et al., 2013; Goodkind & Bicknell, 2018), and fMRI-BOLD responses (Caucheteux & King, 2021; Schrimpf et al., 2020; Shain et al., 2020).

Some of this work has focused on predicting the N400 amplitude for English words with varying levels of expectability (Michaelov et al., 2021). Model-based linguistic surprisal appears to accurately capture the N400 effect that occurs with nouns of lower lexical predictability. Additionally, model-based linguistic surprisal has been shown to correlate with N400 amplitudes for individual words while reading whole English texts, such as excerpts from novels (Frank et al., 2015; Szewczyk & Federmeier, 2022). Recently, surprisal-based measures have also been shown to track trial-by-trial internal model adaptation during exposure to novel linguistic probability distributions within an experimental context (Bornkessel-Schlesewsky et al., 2022).

Taken together, this research demonstrates that linguistic surprisal estimated by language models is a powerful tool to capture the effect of a human parser’s experience with the distribution of words in usage. Importantly, the language models used in this work have access only to probabilistic linguistic information, but no further knowledge about linguistic structure or event structure, that is, no prior knowledge of such notions as “agent,” “patient,” “transitive verb,” and so on. This allows us to directly assess whether the Predicate N400 can be sufficiently explained by probabilistic linguistic information (as measured by surprisal), or whether we additionally need the Agent Preference to capture the Predicate N400.

The Agent Preference is a binary principle that is either fulfilled (when an initial, ambiguous NP disambiguates to the macro-role agent) or violated (when an initial, ambiguous NP disambiguates to the macro-role patient). Thus, the principle does not follow any probabilistic information as it should become active whenever a role-ambiguous NP is encountered. In contrast, linguistic surprisal is a continuous measure that captures predictability at the level of lexical choices, apart from morphosyntactic information. This includes the individual verbs with their semantic and syntactic properties, specifically the *micro-roles* they assign to arguments. In a sentence such as “The monkey eats a banana,” at the level of macro-roles, the monkey is the agent and the banana is the patient. At the level of micro-roles, the monkey is the “eater” and the banana is the “object-being-eaten.”

### The Current Study

We ask whether the Predicate N400 is best explained by an Agent Preference principle as previously suggested or whether a usage-based account in terms of model-based surprisal is sufficient. To this end, we revisit previously conducted EEG experiments that showed a Predicate N400 in German (Haupt et al., 2008), Hindi (Bickel et al., 2015) and Basque (Isasi-Isasmendi et al., 2024).

The argument marking of agents and patients (as proto- or macro-roles) varies in these languages in ways that are crucial for our question (Table 1, focusing exclusively on active-voice sentences). German assigns agents an unmarked case (nominative), Hindi flags them with a special ergative marker under some conditions, and Basque flags them with an ergative marker throughout. As for patients, German assigns them a marked (accusative or dative) case, Hindi either a marked (accusative) or an unmarked (nominative) case, and Basque consistently an unmarked case (nominative, also called absolutive). In German and Basque, some case forms are formally identical with each other, a phenomenon technically known as *syncretism*, and this allows role-ambiguous stimuli of the kind illustrated by Example 1 to assess the Predicate N400 (where nominative and accusative have the same form).

**Table 1.** Relevant grammatical features for each language together with the hypothesised dominant effect for the Predicate N400.

Language	Case system	Unmarked patients	Hypothesised dominant effect
German	nominative A, accusative or dative P	rare	surprisal
Hindi	nominative or ergative A, nominative or accusative P	mixed	mixed
Basque	ergative A, nominative P	common	Agent Preference

*Note*. The German and Hindi studies concern only active voice transitives, the Basque study only intransitives. The accusative vs. dative split in German is conditioned by the lexical verb choice. The nominative vs. ergative split in Hindi is conditioned by aspect, the nominative vs. accusative split by definiteness and animacy. A = agent macro-role, P = patient macro-role.

These syncretisms aside, the case rules imply that unmarked NPs are rarely patients in German, less rarely so in Hindi, and frequently so in Basque. This suggests that a language model can easily learn to expect unmarked NPs to be agents in German, while this is harder in Hindi and still harder in Basque. Accordingly, we hypothesise that the dominant effect of the Predicate N400 is surprisal for German, surprisal in combination with the Agent Preference principle for Hindi, and the Agent Preference alone for Basque.

We conduct two types of analyses. First, we estimate linguistic surprisal at the critical region of the experimental stimuli with recurrent neural networks (RNNs; e.g., long short-term memory models [LSTMs]; Hochreiter & Schmidhuber, 1997) and transformer-based architectures (Vaswani et al., 2017). By using hierarchical Bayesian models of surprisals, we compare their estimates with the qualitative results found in the EEG analysis. We marginalise over the effects of the experimental conditions which were set under the assumption that humans process sentences on the macro-role level. This will reveal how surprisal estimates qualitatively compare to the N400 effects found in the EEG experiments.

In a second step, we directly estimate the N400 amplitudes (in *μ*V) measured in the EEG experiments, using hierarchical Bayesian generalised additive models (GAMs). We fit several models with different predictors: surprisal (derived either from LSTMs or transformers), an Agent Preference principle, or both. By means of model stacking, we analyse which model explains the variance in the EEG signal best. In other words, we intend to show whether surprisal alone is sufficient to explain the Predicate N400 or whether the Agent Preference principle is needed to model the EEG amplitudes.

## MATERIALS AND METHODS

The EEG experiments contain different experimental designs in which, depending on the language, different structures are exploited to create ambiguous initial NPs (Table 2).

**Table 2.** Overview of the study designs.

Language	Condition 1	Condition 2	Critical region	Disambiguating feature
German	initial NP: A-initial vs. P-initial	type of verb: assigning dative vs. accusative to P	auxiliary	A-agreement in number
Hindi	ambiguity of P: ambiguous vs. unambiguous	aspect: imperfective vs. perfective	main verb	lexical information of the verb
Basque	role of intrans. subject: A vs. P	ambiguity of role: ambiguous vs. unambiguous	main verb	lexical information of the verb

*Note*. German: Haupt et al. (2008), Hindi: Bickel et al. (2015), Basque: Isasi-Isasmendi et al. (2024). A = agent macro-role, P = patient macro-role.

### EEG Experiments

#### German

The experimental stimuli and results for German come from the study by Haupt et al. (2008; Table 3). German allows for both agent-initial and patient-initial sentences. Agent-initial sentences are considered to follow the canonical, discourse-neutral word order in declaratives. In the experiment, the design exploits bare plural feminine nouns (i.e., without articles) and proper nouns which syncretise case, that is, the forms are ambiguous between nominative subject and accusative or dative object functions. These were presented in subordinate clauses, in which verbs are placed in the final position. The EEG signal was recorded at the auxiliary verb, which disambiguated the initial NP to either an agent or a patient. The verb categories were manipulated to test whether disambiguation towards patient-initial was functionally the same irrespective of lexical factors. An N400 effect was found with patient-initial sentences with both accusative and dative verbs.

**Table 3.**
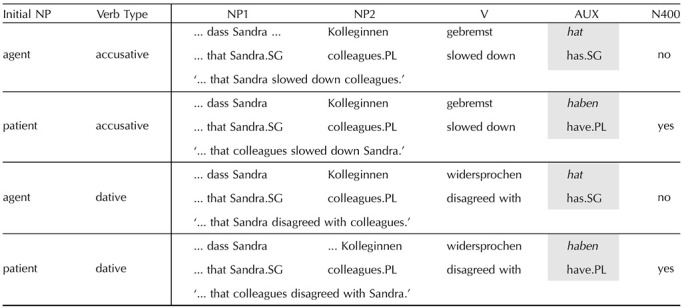
2 × 2 experiment design of Haupt et al. (2008) crossing Initial Noun Phrase (NP) and Verb Type conditions.

*Note*. ERPs were measured at the critical region, shaded in grey.

#### Hindi

The EEG experiment with Hindi speakers stems from Bickel et al. (2015) where a case ambiguity was exploited to create sentences with ambiguous initial NPs (Table 4). In the stimuli, all critical stimuli are patient-initial and the initial NP is always inanimate. These NPs are marked by accusative case if they refer to a definite referent and by the unmarked nominative case if they refer to an indefinite referent. Unmarked nominatives are also used for agents in the imperfective aspect. As a result of this, their role is locally unresolved until it is disambiguated by the verb.

**Table 4.**
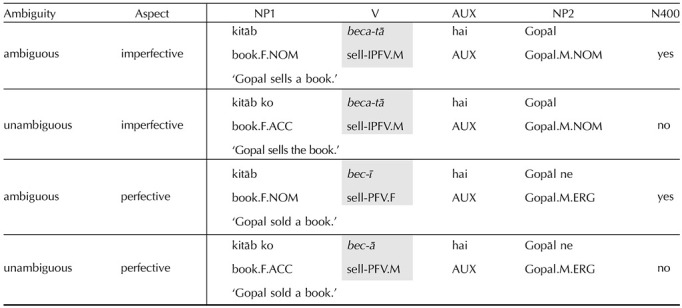
2 × 2 experiment design of Bickel et al. (2015) crossing Ambiguity and Aspect conditions.

*Note*. The critical region is the main verb, shaded in grey.

The experiment design manipulated the ambiguity of the initial NP phrase by leaving it either in the nominative (ambiguous condition) or marking it with the accusative case *ko* (unambiguous condition). Additionally, aspect was manipulated because the perfective aspect restricts nominative NPs to a patient role (since agents are assigned ergative case in this aspect). Hence, the detection of a perfective morphology in the verb might strengthen signals of reanalysis. The EEG signal was recorded at the main verb, the critical region, which disambiguated the initial NP to a patient. To facilitate offline interpretation, the stimuli included a second NP after the critical region, exploiting a common discourse structure in Hindi (with “afterthought” arguments).

The results involved an N400 for the ambiguous condition irrespective of aspect.

#### Basque

For Basque, we use the experiment from Isasi-Isasmendi et al. (2024), which studies the processing of intransitives sentences (Table 5). Intransitive verbs generally fall into two groups, namely those that take agent subjects (unergatives) and those that take patient subjects (unaccusatives) (Borer, 2005; Dowty, 1991; Friedmann et al., 2008; Perlmutter, 1978; Van Valin, 1990). In Basque, subjects of unergative verbs are marked with ergative case, while subjects of unaccusative verbs are marked nominative (Laka, 1996). However, a case syncretism in plural demonstratives creates ambiguity between ergative and nominative cases. Hence, comprehenders reading NPs with plural demonstratives in Basque do not obtain information on the semantic role of the subject until the verb position. The stimuli in Isasi-Isasmendi et al. (2024) exploited this case syncretism in a 2 × 2 design. The sentences differed in whether the initial NP denoted an agent or a patient (as assigned by the two different classes of intransitive verbs) and whether the role of the initial NP was marked unambiguously or ambiguously. In the ambiguous conditions, the verb disambiguated the semantic role of the initial NP to either agent or patient readings.

**Table 5.**
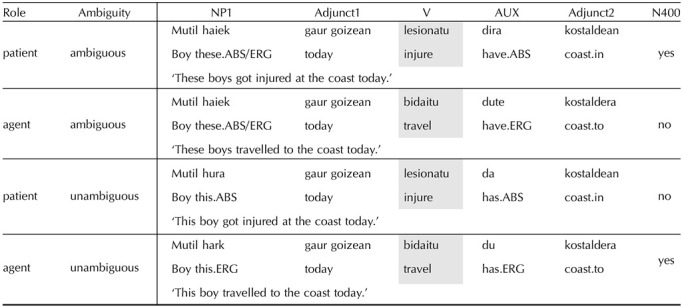
2 × 2 experiment design of Isasi-Isasmendi et al. (2024) crossing Semantic Role and Ambiguity conditions.

*Note*. The critical region is the main verb, shaded in grey.

Isasi-Isasmendi et al. (2024) find that the disambiguation towards patient readings in the verb elicit an N400. In the unambiguous case, however, the picture was reversed; a higher N400 effect was found for agents. As suggested by the authors, a likely reason for this reversed effect is that an unambiguous ergative case leads participants to predict a prototypical agent in a two-participant, transitive scenario. Encountering an intransitive verb violates this prediction and therefore requires revision. In contrast, the unambiguous nominative case marker is expected to be continued by an intransitive verb.

### Language Models

Predictions are shaped by the network architectures that determine how linguistic units are processed, but little is known about how the performance of language models compares across architectures in languages other than English. Therefore, in order to find the best possible estimator of surprisal, we compare language models of three types of architectures: RNNs and both bidirectional and unidirectional transformer-based architectures (Aurnhammer & Frank, 2019; Merkx & Frank, 2021; Michaelov & Bergen, 2020; Schrimpf et al., 2020). We compare the performance of these architectures solely in order to maximise the quality of surprisal estimates as reflexes of language use. It is irrelevant for our purpose whether any quality differences in this reflect how similar a model might be to human language processing, although these similarities vary, as we note in what follows.

RNNs process language incrementally, that is, they process word by word in a sequential order. In this process, the hidden states (i.e., the nodes between the input and output node) receive information from the previously encoded states. Thus, the current state feeds back into the network, making the network recurrent. Hence, RNNs are equipped with a working memory, but the limited size of the hidden vector introduces a memory bottleneck to the unbounded previous context. Due to their incremental processing and limited memory span, RNNs are thought to reflect the human processing system (Frank et al., 2019).

Transformers, by contrast, process language very differently. They process the whole sentence at once. An in-built attention mechanism allows the model to “look back” at previous words directly. These models therefore implement some memory of words that is not limited by temporal distance. A common finding across studies (Michaelov et al., 2021) is that transformers outperform RNNs in terms of their predictive accuracy of EEG results, even when models of both architectures achieve the same language model quality measured by next-word predictability (Merkx & Frank, 2021). This challenges the opinion that transformers have “little cognitive motivation” (Rogers et al., 2020, p. 842).

So far we lack a good cognitive explanation of the consequences of why one architecture exhibits more similar processing behaviours to humans than another, leaving open any neurocognitive interpretation (Armeni et al., 2017). In response to this, we estimate surprisal with an LSTM (Hochreiter & Schmidhuber, 1997), the most successful variant of an RNN, as well as pretrained transformer-based architectures (Devlin et al., 2019; Liu et al., 2019). For each model, we calculate surprisal at the critical region of the EEG experiment, that is, where the electrophysiological response was recorded.

#### LSTMs

We trained a two-layer, unidirectional LSTM on next word prediction for each language with the code provided by Gulordava et al. (2018). The training data are mostly written texts from OSCAR (Ortiz Suárez et al., 2019) and the Wikipedia Corpus for Hindi (∼100K tokens in the training set), the Basque Multimedia Corpus for Basque (Agerri et al., 2020; 195K tokens in the training set), and the Wikipedia Corpus for German (cleaned and provided at https://github.com/t-systems-on-site-services-gmbh/german-wikipedia-text-corpus; ∼189K tokens in the training set. See S1 in the Supporting Information available at https://doi.org/10.1162/nol_a_00121. The German corpus is reduced in size so that the number of tokens is comparable to the Basque corpus while the Hindi corpus size is the smallest due to data availability. The models are implemented in Python, using the library PyTorch (Paszke et al., 2019). We tune several hyperparameters, such as the size of the hidden layers and the learning rate. The models are trained for a maximum number of 10 epochs or when early stopping is reached (see Supporting Information S1 for the full grid of hyperparameters and results).

Surprisal is calculated for the word at the disambiguating, critical region of the experiment. The words for which the model has representations, that is, the vocabulary of the LSTMs, is limited to the 50K most frequent words. Thus, some stimuli contained out-of-vocabulary (OOV) words. We excluded all stimuli that contained OOV words up to and including the critical word. The number of stimuli available for each language can be found in the Supporting Information S3.1.

#### Bidirectional transformers

For bidirectional transformer models, we use BERT architecture (Devlin et al., 2019) for German and Hindi, and RoBERTa (Liu et al., 2019) for Basque. Both of these architectures belong to the same subcategory of transformer models. They are trained on a masked language modeling objective, that is they are trained to predict words, bidirectionally, where the words are masked. We access the pretrained models through HuggingFace (n.d.), the respective pretrained models can be found in the Supporting Information S1. The decision to use this set of models is opportunistically based on their availability. The word at the critical region is masked with a special token, which has to be predicted by the model. Since bidirectional transformers process the whole sequence at once, we only feed part of the sentence up to the critical region so that the model cannot look at words coming after the critical region.

It has been questioned whether such bidirectional models can be evaluated on a unidirectional task like sentence comprehension that is incongruent with the way in which they were trained (Merkx & Frank, 2021). However, surprisal estimates from bidirectional transformers have proven to be good predictors of both behavioural (Hollenstein et al., 2021; Merkx & Frank, 2021) and neurophysiological (Michaelov et al., 2021) measures. Furthermore, while human sentence comprehension is a unidirectional task, it is not necessarily the case that the human parser is only trained unidirectionally. After all, listeners and readers are likely to have access to vast amounts of the linguistic knowledge they have acquired, with no particular sequential order necessarily imposed on memory. For these reasons we find it important to include bidirectional transformer models.

Both BERT and RoBERTa use subword tokenisers: WordPiece (Schuster & Nakajima, 2012) for BERT and Byte-Pair Encoding (Sennrich et al., 2016) for RoBERTa. Therefore, they represent infrequent words at the subword level, and consequently there are no OOV words, so that all words can be represented as vectors and no stimuli have to be excluded.

#### Unidirectional transformers

Lastly, we use GPT-2 models as representatives of unidirectional transformer models. GPT variants have become the standard in the field, which is why it is particularly interesting to analyse their predictive power for the EEG results. As opposed to bidirectional models, such as BERT, GPT only considers the left context when predicting new words. As noted in the preceding section, it is sometimes argued that this is closer to how humans process sentences. We again use pretrained GPT-2 models for Basque and German, accessed on HuggingFace (again, the respective pretrained models can be found in the Supporting Information S1). For Hindi, however, no pretrained GPT-2 is available at the time of writing, which is why we opted for training our own model. To this end, we follow de Vries and Nissim’s (2021) training scheme, which is well suited when computational power and training data are relatively scarce. The training process starts from the English GPT-2 model and unfolds over two steps. After training a new tokeniser on the Hindi training data set, in the first step, only the lexical embeddings are trained while the other layers are frozen. In the second step, the whole model is trained. The model was trained on four NVIDIA A100 Tensor Core GPUs for four days. Again, GPT-2 uses a subword tokeniser based on Byte-Pair-Encoding (Sennrich et al., 2016) so that no stimuli had to be excluded.

#### Evaluation of the language models

Evaluating the performance of language models is commonly done with perplexity (Jurafsky & Martin, 2023), that is, in terms of the inverse probability of an unseen text, divided by the number of words in the text. For our current purposes, perplexity is suboptimal as an evaluation metric for two reasons. First, probability distributions and therefore the observable perplexity of languages vary (Bentz et al., 2017; Coupé et al., 2019). Thus, while we can compare models of the same language using perplexity, the measure is less useful to compare language models across different languages. Second, it is still an open question whether language models with lower perplexity are actually more human-like (Kuribayashi et al., 2021).

Instead, we opt for a grammaticality test that is tailored to each language, that is, measures the model’s performance in relationship to the specific affordances of each language. We compare the average surprisal of sentences with a grammatically correct syntactic structure to the average surprisal of sentences that are scrambled into ungrammatical sentences. This means that our evaluation metric is the surprisal of ungrammatical sentences minus the surprisal of grammatical sentences (Δ*Surprisal*), with larger differences indicating better performance. The sentences are derived from the stimuli of the respective experiments on German, Hindi, and Basque. The ungrammatical sentences are permutations of the original sentences based on two to four ungrammatical word order variants; for example, *… dass Schwimmerinnen Stefan gestoßen haben.* “… that swimmers Stefan pushed have” (grammatical) versus *… dass Schwimmerinnen haben Stefan gestoßen. “… that swimmers have Stefan pushed” (ungrammatical). (The sentences are accessible at https://osf.io/hbj67/, directory grammaticality_test; see also S1.4 in the Supporting Information). The surprisal values are calculated for each word one-by-one in linear order. For BERT/RoBERTa models, we mask the word *w*_*i*_ for which surprisal is calculated and remove any words (*w*_*i*+1+…+*n*_) that occur after it. For each sentence, we then calculate the average surprisal of the sentence.

**Figure 1.**
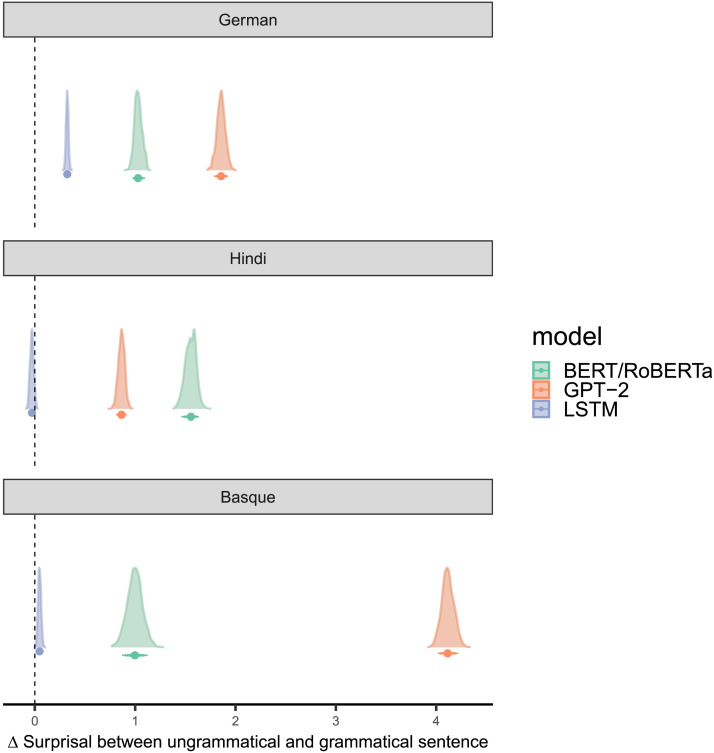
For each language and model, the table shows the posterior estimates of differences in surprisal values (ΔSurprisal) between ungrammatical and grammatical sentences with 50%, 80%, and 90% credible intervals, controlling for differences in sentences. ΔSurprisal is calculated by subtracting the mean surprisal of the grammatical sentence from the ungrammatical sentence. Values are then estimated with a Bayesian model that controls for the variance in the stimuli as a random effect and quantifies the estimates’ probabilities (see Supporting Information S1.4.4). LSTM = long short-term memory models.

Figure 1 shows posterior estimates (controlled for variation between sentences as a random effect) of Δ*Surprisal*. Estimates larger than zero indicate that the model assigns higher surprisal to ungrammatical sentences, that is, that it makes the same decision as humans.

For German, all models pass the grammaticality test (LSTM: mean = 0.32 with 89% credible interval CI = [0.30, 0.34]; BERT: mean = 1.03, CI = [0.97, 1.1]; GPT: mean = 1.85, CI = [1.8, 1.9]). For Hindi and Basque, this is only the case for BERT/RoBERTa (Hindi: mean = 1.56, CI = [1.47, 1.6]; Basque: mean = 0.1, CI = [0.87, 1.1]) and GPT-2 (Hindi: mean = 0.86, CI = [0.81, 0.9]; Basque: mean = 4.13, CI = [4.03, 4.2]). LSTMs perform at chance in the case of Hindi (mean = −0.03, CI = [−0.06, 0.0] and only slightly above change in the case of Basque (mean = 0.048, CI = [0.02, 0.1]). This suggests that apart from German, the LSTM models do not seem to learn grammatical structures sufficiently to determine between grammatical and ungrammatical sentences.

The different results of the LSTMs may be driven by the number of possible word orders in each language. German has a relatively strict word order (Suitner et al., 2021) while Hindi and Basque are much more permissive, making it harder to construct ungrammatical sentences by word order permutation (Laka, 1996; Mohanan, 1994a, 1994b). Thus, a model of a language with less ordering possibilities might show higher surprisal if the word order is ungrammatical while the difference is not so clear in some language models if the language has free word order. Interestingly, these differences in word order freedom have no impact on the transformer models’s performance. Among the BERT models, Hindi shows the highest Δ*Surprisal*, and among the GPT-2 models, Basque shows the highest Δ*Surprisal*. The numeric differences between Δ*Surprisal* across languages is difficult to explain in detail; more research would be needed on this.

### Statistical Analysis

In our first analysis, we qualitatively assess whether surprisal estimated by the language models corresponds to the EEG results reported in the studies (Bickel et al., 2015; Haupt et al., 2008; Isasi-Isasmendi et al., 2024). To this end, we estimate the surprisal under the experimental conditions with Bayesian hierarchical models using the brms (Bürkner, 2017, 2018) interface to Stan (Carpenter et al., 2017) in R (R Core Team, 2020). These models allow us to estimate the differences in surprisal for the role-disambiguating words in the sentences presented in the EEG experiments (disambiguating the initial NP towards agent or patient), together with the probabilities of the differences. We fit three models per language, with surprisals based on LSTMs, BERT/RoBERTa and GPT-2. Visual inspection of the raw surprisal values suggests that there are many outliers, and so we model surprisal as drawn from a Student-*t* distribution. We include the two conditions from the EEG experiments (Initial NP and Verb Type for German, Ambiguity and Aspect for Hindi, and Semantic Role and Ambiguity for Basque) and their interaction as main effects, and experimental stimuli as a random effect, with varying random slopes for the two conditions.

We choose Gamma priors for the degrees of freedom of the Student-*t* likelihood (German and Basque: Gamma(*α* = 2, *β* = 1), Hindi: Gamma(*α* = 2, *β* = 0.1)), half-Cauchy or exponential priors for its standard deviation and also for the standard deviation of the random effects (German: Exponential(*λ* = 1), Hindi and Basque: half-Cauchy(*μ* = 0, *σ* = 4)), and Normal or Student-*t* priors for the intercept (German and Basque: Normal(*μ* = 0, *σ* = 2), Hindi: Student-titt(*ν* = 2, *μ* = 0, *σ* = 2)). The different priors are selected based on diagnostics that indicate convergence (effective sample size measures and $\hat{R}$ statistic; see Supporting Information S3.2, for details).

On request by reviewers, we also provide *p* values of paired *t* tests of the condition of interest (Initial NP for German, Ambiguity for Hindi, Role for Basque) for each control condition and language.

In our second analysis, we compare surprisal and the Agent Preference as predictors of the EEG signal in *μ*V directly. For this we focus on the time window of 300–500 ms post-critical region because this is the commonly used time window for N400 analyses. We average *μ*V values in this window. We then apply GAMs to predict mean *μ*V in response to the predictors while controlling for signal topography across the entire scalp. By using GAMs we depart from traditional EEG regression models, which reduce the spatial information of the data by choosing regions of interest (ROIs within which signals are averaged) or a small set of electrodes. This way, we avoid a priori decisions on ROIs or electrode choice while at the same time preserving the spatial relationship between electrodes in a nonlinear way (De Cat et al., 2015; Isasi-Isasmendi et al., 2024; Tremblay & Newman, 2015). The main advantage of this approach is that it lets the data decide the topography of the signal. This is particularly important when investigating different languages because topographies might differ across them. In these regards, the GAM approach is similar in spirit to traditional cluster-based permutation tests but unlike these tests, GAMs allow multiple predictors, which is key to comparing effects of surprisal and the Agent Preference (De Cat et al., 2015; Sauppe et al., 2023; Tremblay & Newman, 2015).

For each language, we fit five models of the N400 amplitude in *μ*V with different predictors. We fit one model with an Agent Preference predictor alone, three models with a surprisal predictor alone–estimated by LSTMs, BERT (RoBERTa, in the case of Basque) or GPT-2–and three models with both the Agent Preference and surprisal predictors. Additionally, we fit a baseline model that contains neither of the two predictors.

Table 6 lists the models in more detail, using code notation for GAMs in R. The predictors Agent Preference condition (cond in Table 6) and surprisal (surp.lstm, surp.bert, and surp.gpt) are modeled as fixed effects, together with trial number (trial.n) to control for within-experiment effects. We expect the Agent Preference condition and surprisal effecs to vary over the spatial distribution of the electrodes. To capture this variation, we use tensor products (the t2() function in Table 6) which smooth effects between the *x*- and *y*-coordinates that represent electrode position. Since tensor products account for the marginalised effect of *x* and *y*, there is no assumption of the smoothness being consistent between the two coordinates. We let the smooth (tensor) function vary over the main predictors (implemented by the by parameter in Table 6, where, for instance, t2(x, y, by = surp.lstm) represents the smooth for electrode position (indicated by *x* and *y*) for each surprisal value estimated by an LSTM).

**Table 6.** Generalised additive models for estimating *μ*V using R notation.

Model name	Regression
Agent Preference alone	*μ* V ∼ 1 + cond + trial.n + t2(x, y, by=cond) + t2(x, y, part, bs=’re’) + t2(x, y, item, bs = ’re’)
surprisal lstm alone	*μ* V ∼ 1 + surp.lstm + trial.n + t2(x, y, by=surp.lstm) + t2(x, y, part, bs=’re’) + t2(x, y, item, bs = ’re’)
surprisal BERT/RoBERTa alone	*μ* V ∼ 1 + revsurp.bert + trial.n + t2(x, y, by=revsurp.bert) + t2(x, y, part, bs=’re’) + t2(x, y, item, bs = ’re’)
revsurprisal GPT-2 alone	*μ* V ∼ 1 + surp.gpt + trial.n + t2(x, y, by=surp.gpt) + t2(x, y, part, bs=’re’) + t2(x, y, item, bs = ’re’)
surprisal lstm and Agent Preference	*μ* V ∼ 1 + surp.lstm + cond + trial.n + t2(x, y, by=surp.lstm) + t2(x, y, by=cond) + t2(x, y, part, bs=’re’) + t2(x, y, item, bs = ’re’)
surpisal BERT/RoBERTa and Agent Preference	*μ* V ∼ 1 + surp.bert + cond + trial.n + t2(x, y, by=surp.bert) + t2(x, y, by=cond) + t2(x, y, part, bs=’re’) + t2(x, y, item, bs = ’re’)
surpisal GPT-2 and Agent Preference	*μ* V ∼ 1 + surp.gpt + cond + trial.n + t2(x, y, by=surp.gpt) + t2(x, y, by=cond) + t2(x, y, part, bs=’re’) + t2(x, y, item, bs = ’re’)
baseline	*μ* V ∼ 1 + trial.n + t2(x, y, part, bs=’re’) + t2(x, y, item, bs = ’re’)

*Note*. We include the Agent Preference condition (cond), surprisal values (surp.* for surprisal values estimated by LSTMs, RoBERTa, and GPT) and trial number (trial.n) as linear main effects. The latter two are *z*-transformed. We additionally include tensors for smooths of the predictors cond and surp.* over the electrode positions (indicated by their coordinates *x* and *y*). Random effects (bs = ’re’) are included as smooths for each participant (part) and item (item). Condition (cond) is the binary Agent Preference condition (reanalysis or no reanalysis). LSTM = long short-term memory models.

We model the variation between stimuli and participant as random effects, smoothing over the electrodes with random coefficients for each level of the participant or item ID. These effects are again captured by tensor products (e.g., t2(x, y, item, bs = ’re’) in Table 6, where bs = ’re’ declares a random effect). This allows for the nonlinear relations that are needed to capture the topography. Due to convergence issues, we could not add random slopes to the model. However, the posterior residuals are fairly constant across items and individuals and in fact do not diverge far from the population level estimates, thus not biasing estimates in one or the other direction (cf. Supporting Information S6).

At the spatial resolution of EEG, GAMs cannot fully separate actual spatial trends in the EEG signal from contingent residual autocorrelation (cf. Simpson, 2018). In response to this, we also perform separate analyses on individual electrodes that show the strongest signal in the GAM model (using the same model structure but replacing the tensor product smoother by random slopes, see Supporting Information S7). This approach removes all residual autocorrelation because no spatial structure is present in the data. However, we caution that single electrodes capture only a limited part of the potentially relevant EEG signal, which is inherently distributed over the scalp because it stems from cortical processes that are transported through the head’s conductive volumes.

The Agent Preference condition is a binary variable that categorises the stimuli into whether or not a reanalysis towards the patient is expected due to the Agent Preference taking effect. The Agent Preference should lead to a reanalysis only when an ambiguous NP is disambiguated towards a patient (Table 7).

**Table 7.** Overview of the categorisation of the experiment conditions (Condition 1 and Condition 2) according to what they predict in terms of the Agent Preference, together with whether a stronger N400 amplitude was observed.

Language	Condition 1	Condition 2	Agent Preference condition	N400 observed in the original study
German	agent initial	accusative	no reanalysis	no
German	patient initial	accusative	reanalysis	yes
German	agent initial	dative	no reanalysis	no
German	patient initial	dative	reanalysis	yes
Hindi	ambiguous	imperfective	reanalysis	yes
Hindi	unambiguous	imperfective	no reanalysis	no
Hindi	ambiguous	perfective	reanalysis	yes
Hindi	unambiguous	perfective	no reanalysis	no
Basque	patient	ambiguous	reanalysis	yes
Basque	agent	ambiguous	no reanalysis	no
Basque	patient	unambiguous	no reanalysis	no
Basque	agent	unambiguous	no reanalysis	yes

Consequently, for German in both patient-initial conditions, a reanalysis is expected (reanalysis), whereas no reanalysis is expected in the agent-initial conditions (no reanalysis). In Hindi, ambiguous patients in both aspects should lead to a reanalysis (reanalysis) whereas unambiguous patients should not lead to a reanalysis (no reanalysis). In Basque, a reanalysis towards the patient is only expected with ambiguous patients (reanalysis), while the other three conditions are assigned the no reanalysis condition. All continuous predictors (i.e., trial number and surprisal) are *z*-scored.

As in the qualitative analysis, we fit the models in a Bayesian framework, in order to quantify the probability of the estimates. We assume that the *μ*V values are drawn from a normal distribution, and we choose weakly informative priors for the slope, Normal (*μ* = 0, *σ* = 2), and an Exponential (*λ* = 1) for the standard deviation of the random effects (McElreath, 2020).

We compare models via their performance under leave-one-out cross-validation (McElreath, 2020; Vehtari et al., 2017) in a technique known as stacking (Yao et al., 2018). While traditionally used for improving predictions by ensembling different models, stacking has excellent statistical behaviour also for comparing the relative performance of models (guarding against under-fitting and over-fitting) and has come to serve as a substitute for other approaches like the Akaike information criterion and variants thereof (Bürkner et al., 2021; Höge et al., 2020). Model stacking allocates weights to models in such a way that they jointly maximise prediction accuracy. Concretely, the weight for each model is determined by maximising the log-probability under leave-one-out cross-validation, that is, $\hat{w}=\arg\max_{w}\frac{1}{n}\sum_{i=1}^{n}\;\log\sum_{k=1}^{K}$ *w*_*k*_*P*(*y*_*i*_|*y*_−*i*_, *M*_*k*_), where *n* is the number of observations, *K* the number of models in the stack, and *P* the probability density of *y*_*i*_ when fitting model *M*_*k*_ without that observation (“−*i*”), approximated by Pareto-smoothed importance sampling from the posterior (Vehtari et al., 2017); $\hat{w}$ is constrained to sum to 1.

As a result, higher weight of a model indicates that this model contributes better predictions to the ensemble than models with lower weights. By stacking models with each predictor alone and models with both together we can therefore assess their relative prediction success. We then inspect the heighest-weight model(s) and report the effects of its predictors and the posterior probability distributions of these effects.

Throughout, we only select the sentences from the experiments for which a surprisal estimate is available from both LSTM and transformer language models.

## RESULTS

### Qualitative Comparison Between Surprisal Estimates and EEG Results

**Figure 2.**
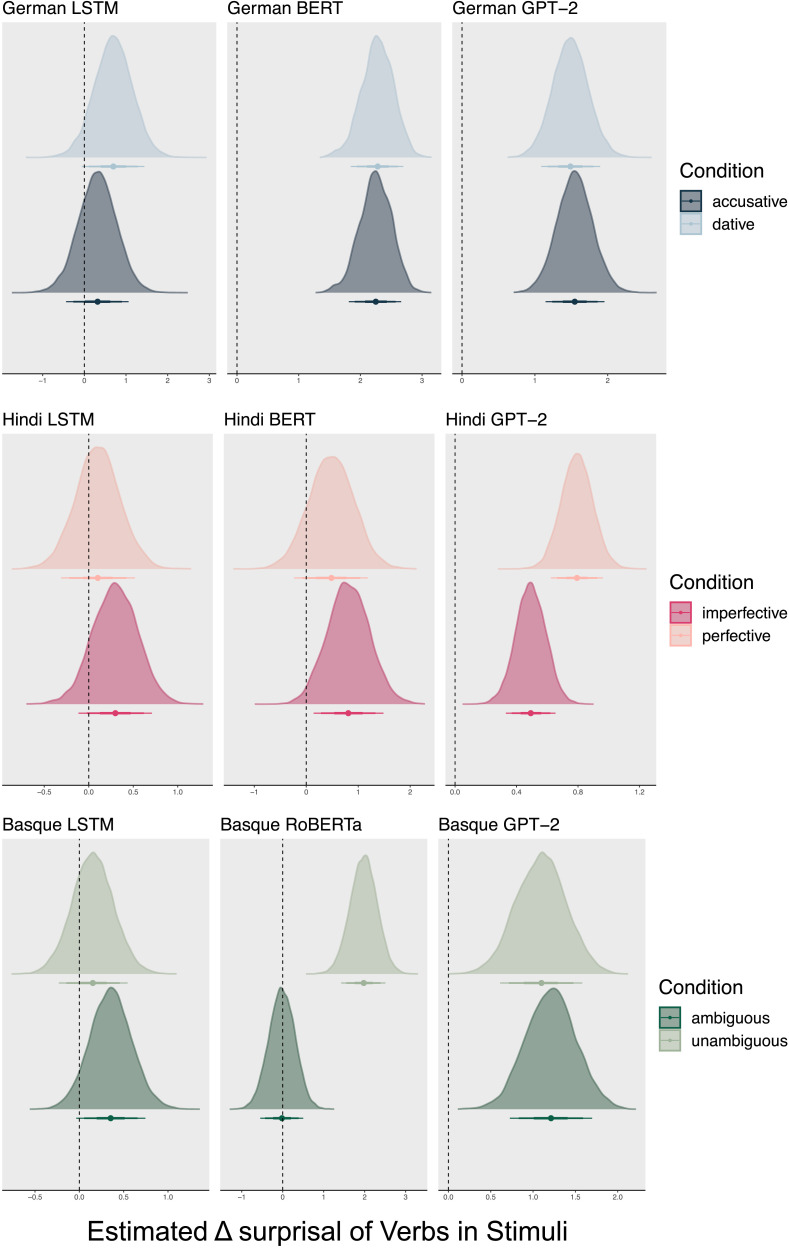
Posterior distributions of the estimated surprisal difference (ΔSurprisal) between the experimental conditions that elicited the Predicate N400 (German: patient initial–agent initial; Hindi: ambiguous–unambiguous; Basque: patient–agent) across control conditions (Condition 2 in Table 7). Horizontal bars indicate 50%, 80%, and 90% highest-density credible intervals. In order to show a substantial difference between conditions, ΔSurprisal estimates are expected to exclude 0. The estimated ΔSurprisal on the sentence-level can be found in the Supporting Information S2 (Analysis 1: Predicting Surprisal).

Figure 2 shows, for each experiment, the posterior distributions of Δ*Surprisal* estimates between the condition that elicited the Predicate N400 minus the condition where it is not expected (German: patient initial–agent initial; Hindi: ambiguous *x* unambiguous; Basque: patient–agent) for each of the control condition (Condition 2 in Table 7) and language model. Since the number of stimuli is lower for LSTM estimates than for transformer estimates for the reasons mentioned above, we replaced missing stimuli with possible alternatives to improve statistical power. The results of the stimuli set including the replaced stimuli are in line with the original, limited set of stimuli. Here we only show the original stimuli, but see the Supporting Information S3.4 for the expanded set.

#### German, Hindi, and Basque

##### German.

The LSTM estimates higher surprisal values for patient-initial sentences than agent-initial sentences although posterior credible intervals include 0 (LSTM/accusative: mean = 0.3, 89% CI = [−0.39, 1.1], paired *t* = −7.38, *df* = 287, *p* < 0.001; LSTM/dative: mean = 0.7, CI [−0.01, 1.4], *t* = −2.87, *df* = 223, *p* = 0.004). Δ*Surprisal* is slightly larger with accusative verbs than dative verbs. The smaller difference in dative verbs can be explained by the fact patient-initial sentences are more common with dative verbs than accusative verbs (Bader & Häussler, 2010). Both the German BERT and GPT-2 models assign substantially higher surprisal values for patient-initial than agent-initial sentences with both accusative and dative verbs, with posterior credible intervals excluding 0 (BERT/accusative: mean = 2.2, CI = [1.84, 2.7], *t* = −29.95, *df* = 1,343, *p* < 0.001; BERT/dative: mean = 2.3, CI = [1.86, 2.7], *t* = –31.58, *df* = 1,343, *p* < 0.001; GPT-2/accusative: mean = 1.5, CI = [1.16, 1.9], *t* = −12.45, *df* = 335, *p* < 0.001; GPT-2/dative: mean = 1.6, CI = [1.10, 1.9], *t* = −10.46, *df* = 335, *p* < 0.001). Therefore, the surprisal values estimated by these models are in line with the EEG results. The results are also in line with the results from the grammaticality test in which BERT and GPT-2 performed better than LSTM.

##### Hindi.

The Δ*Surprisal* values estimated by the LSTM language model are higher for the critical unambiguous condition in both control conditions, but substantial proportions of the posterior includes 0 (LSTM/imperfective: mean = 0.3, = CI [−0.11, 0.7], *t* = −1.93, *df* = 18, *p* = 0.07; LSTM/perfective: mean = 0.1, CI = [−0.31, 0.5], *t* = −0.76, *df* = 18, *p* = 0.46). The results from the BERT model look similar, but the credible intervals overlap slightly less with 0 (BERT/imperfective: mean = 0.8, CI = [0.16, 1.5], *t* = −2.77, *df* = 59, *p* = 0.007; BERT/perfective: mean = 0.9, CI = [0.24, 1.6], *t* = −0.82, *df* = 59, *p* = 0.42). The surprisal values estimated by GPT-2 show Δ*Surprisal* systematically higher than 0 (GPT-2/imperfective: mean = 0.5, CI = [0.34, 0.6], *t* = −5.32, *df* = 59, *p* < 0.001; GPT-2/perfective: mean = 0.8, CI = [0.63, 1.0] *t* = −6.92, *df* = 59, *p* < 0.001). This is in line with the EEG results and also with the results from the grammaticality test in Figure 1. At the same time, the GPT-2 surprisal estimates differ from the EEG results insofar as they suggest a difference between perfective and imperfective aspect that was not present in the EEG data.

##### Basque.

The Δ*Surprisal* estimated by the LSTM model indicates higher surprisal for patientive subjects than agentive subjects in both conditions. However, the credible intervals strongly overlap with 0, limiting the evidence (LSTM/ambiguous: mean = 0.4, CI = [−0.02, 0.7], *t* = −1.11, *df* = 62, *p* = 0.27; LSTM/unambiguous: mean = 0.2, CI = [−0.23, 05], *t* = −0.26, *df* = 62, *p* = 0.79). The RoBERTa model estimates high Δ*Surprisal* in the unambiguous condition (RoBERTa/unambiguous: mean = 2.0, CI = [1.46, 2.5], *t* = −5.74, *df* = 191, *p* < 0.001), but not in the ambiguous condition (RoBERTa/ambiguous: mean = 0.0, CI = [−0.53, 0.5], *t* = 0.40, *df* = 191, *p* = 0.69), contrary to the EEG results where the results were reversed in the unambiguous condition. The Δ*Surprisal* estimated by the GPT-2 model are in line with the EEG results in the ambiguous condition (GPT-2/ambiguous: mean = 1.2, CI = [0.73, 1.7], *t* = −4.26, *df* = 191, *p* < 0.001), but they again estimate the opposite of the EEG results in the unambiguous condition (GPT-2/unambiguous: mean = 1.1, CI = [0.62, 1.6], *t* = −4.0, *df* = 191, *p* < 0.001). GPT-2 was the model for Basque that estimated the highest surprisal values for ungrammatical sentences, yet it estimates surprisal values of the stimuli that are not in line with the EEG results.

#### Trends

Overall, we see some trends in line with the EEG experiment results. For German, the surprisal values consistently correlate with the EEG findings, especially in the transformer models (BERT and GPT-2). The Hindi results are also mostly in line with the EEG findings, but where the fit is best (GPT-2 surprisals), the models also estimate a difference between aspect conditions that was not present in the EEG data. The results in Basque are not in line with the EEG results except for the surprisal values in the ambiguous condition estimated by GPT-2 models and with reduced evidence in the LSTM models. The fact that both the LSTM and GPT-2 predict higher surprisal for patients in the unambiguous condition, which was reversed in the EEG experiment, may suggest that the predictions are mainly lexically driven. Nevertheless, the models show that they assign, in most cases, distinct probabilities to ambiguous and unambiguous cases (Basque and Hindi) and initial NPs (German), which suggests that the models are sensitive to morphosyntactic information.

### Predicting N400 Amplitudes

**Figure 3.**
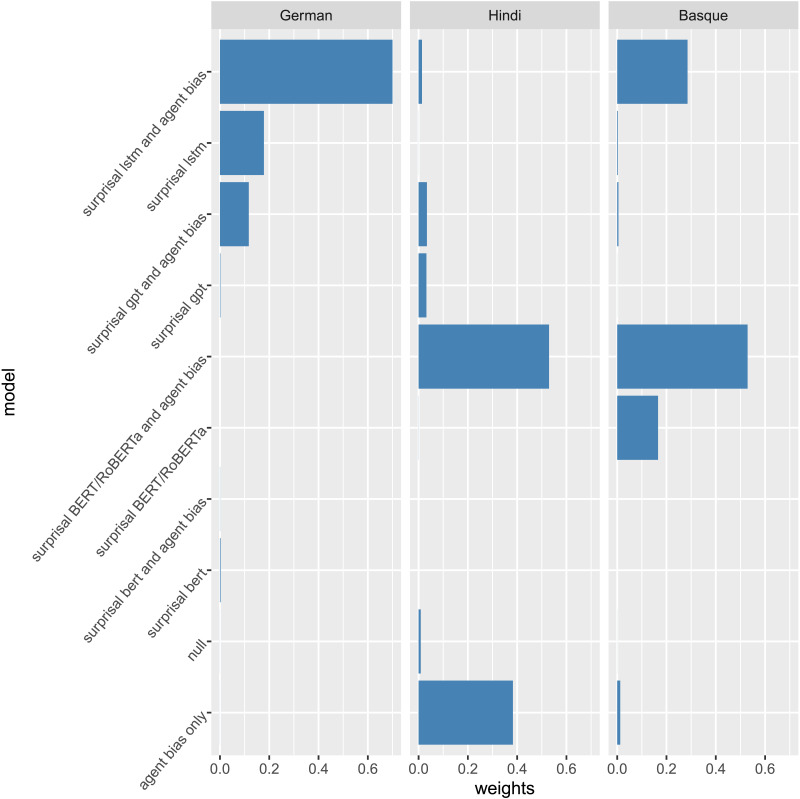
Relative weights of models as determined by model stacking. Weights are allocated to models in such a way that they jointly maximise prediction accuracy. Each model is a Bayesian generalised additive model with the following predictors (in addition to random effects of sentence and participant and a main effect of trial number): surprisal alone, Agent Preference alone, surprisal and Agent Preference together, or neither of the two (null). Agent Preference is a binary variable, categorising sentences into those where the Agent Preference principle predicts role reanalysis at the position of the verb (because the initial ambiguous NP turns out to be a patient) vs. those where no reanalysis is predicted (because the NP is indeed an agent). Surprisal is a continuous variable derived from LSTM, BERT/RoBERTa, or GPT-2 models. For all languages, models with both Agent Preference and surprisal (estimated by BERT/RoBERTa models for Basque and Hindi, and an LSTM model for German) leverage most of the weight. NP = noun phrase.

**Figure 4.**
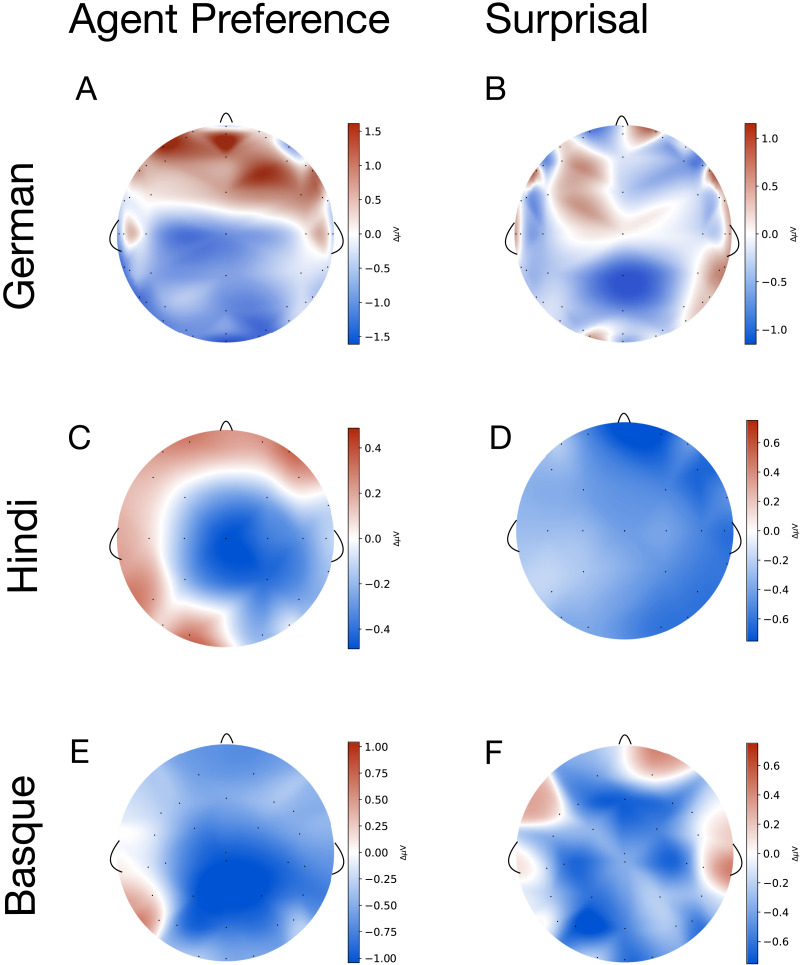
Pair-wise grand mean differences of event related potentials in the N400 time window (300–500 ms relative to verb onset). (Left column) Topography plots of observed grand mean differences in amplitudes between sentences with vs. without reanalysis as predicted by the Agent Preference principle. (Right column) Topography plots of observed grand mean differences in amplitudes for sentences with high vs. low surprisal verbs (>0 and <0), as estimated by the highest-weighted model (cf. Figure 3).

Figure 3 depicts the relative weights determined by model stacking for each language. In each language the best-performing model includes both surprisal and the Agent Preference as predictors. Figure 4 displays the grand mean difference in amplitudes between sentences with reanalysis versus without reanalysis, as predicted by the Agent Preference principle, as well as between sentences with high versus low surprisal verbs, as predicted by Surprisal Theory. For display purposes in the figure, we bin the continuous surprisal values into high and low surprisal verbs (larger than 0 and lower than 0).

**Figure 5.**
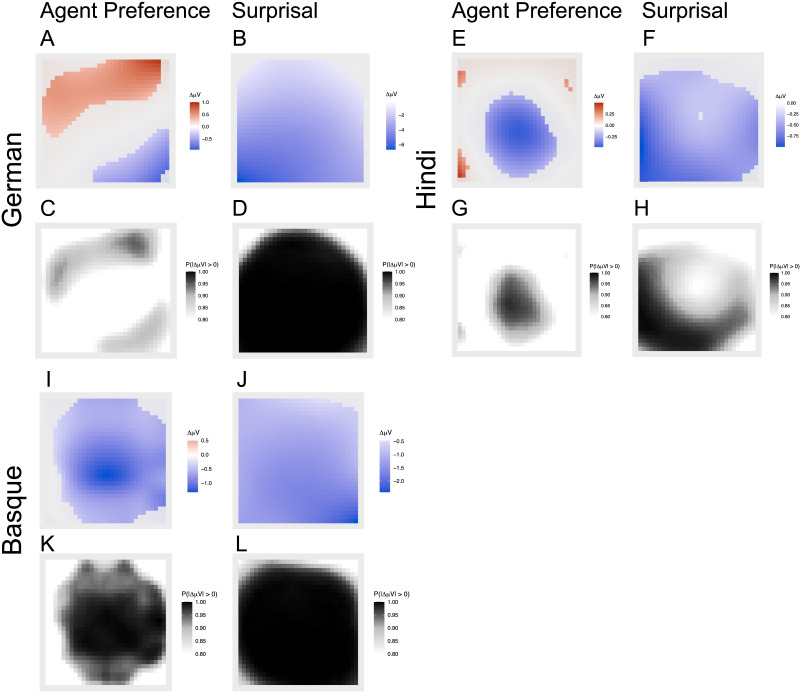
Pair-wise fitted differences of event related potentials in the N400 time window (300–500 ms relative to verb onset), drawn from the highest-weighted model (cf. Figure 3). Upper panels in each language (A–B, E–F, I–J) quantify effect size and electrode regions through the posterior mean differences of smooth surfaces at each scalp coordinate for sentences with vs. sentences without the predicted reanalysis (A, E, and I) and for sentences with high vs. low surprisal verbs (+2 vs. −2 *SD* from the mean; B, F, and J). Mean differences with posterior probability <0.8 are plotted grey. Lower panels (C–D, G–H, K–L) quantify the evidence through the proportion of posterior differences that are below or above 0 at each coordinate. Proportions <0.8 are left white. In German, the Agent Preference principle has a considerably smaller (A) and less supported (C) effect than surprisal (B and D), a difference that is less strong in Hindi and even weaker in Basque.

Figure 5 shows the fitted values for the highest-ranked model in model stacking (Figure 3). The upper panels in each language (Figure 5A–B, E–F, and I–J) quantify effect size and electrode regions through the posterior mean differences of smooth surfaces at each scalp coordinate. The plots in the left column show posterior mean differences for sentences where reanalysis due to the Agent Preference principle is expected versus where no reanalysis is expected (Figure 5A, E, and I). The plots on the right-hand side show posterior mean differences for sentences with high versus low surprisal verbs (+2 vs. −2 standard deviations from the *z*-scored mean; Figure 5B, F, and J). (The model captured surprisal as a continuous predictor, but for display purposes we selected these differences; see Supporting Information for the full results, S3.3, S4.3 and S5.3.) In the lower panels (Figure 5C–D, G–H, and K–L), the evidence is quantified by the proportion of posterior draws with non-zero difference between conditions. The Supporting Information S3.3, S4.3 and S5.3 show results of all models, including those that receive little or no weight in model stacking.

#### German, Hindi, and Basque

##### German.

The model with surprisal (LSTM) and Agent Preference condition leverages most of the weight (70%) for predicting the N400 amplitude (Figure 3). The second-highest ranking model has LSTM-based surprisal as its only predictor, but this model leverages only 18% of the total weight. To some extent this is in contrast to what we would expect based on the grammaticality test, where the LSTM performed slightly worse than the transformer models, although it still passed the test.

The posterior mean difference between the predicted *μ*V for the Agent Preference condition shows an effect of reanalysis towards patients (i.e., more negative *μ*V) in the right-posterior region (Figure 5A). The effect of surprisal is more widely distributed across coordinates (Figure 5B). The estimated effect size of the Agent Preference is only approximately one-sixth (strongest mean estimate Δ*μ*V = −0.95) of the one of surprisal (strongest mean estimate Δ*μ*V = −6.625), indicating that surprisal is a more important predictor. This is confirmed by the probability mass of the posterior distributions of the mean difference, with consistently high proportions of the probability mass below 0 across coordinates for surprisal (Figure 5D, strongest effect with *P*(|Δ*μ*V| > 0) = 1) but not for the Agent Preference (Figure 5C, strongest effect with *P*(|Δ*μ*V| > 0) = 0.90). An analysis of the electrodes with the strongest effects confirms these results, although of course with reduced power. We estimate a much stronger effect and higher support for surprisal (estimated mean effect at electrode CP5 Δ*μ*V = −5.71, *P*(|Δ*μ*V| > 0) = 0.98) than for the Agent Preference (estimated mean effect at electrode P8 Δ*μ*V = − 1.01, *P*(|Δ*μ*V| > 0) = 0.82; cf. Supporting Information S7).

##### Hindi.

For Hindi, the model that receives the highest proportion (53%) of the total weight is the one incorporating both the Agent Preference condition and surprisal estimated by transformers (Figure 3) , which were also the models that performed best in the grammaticality task (Figure 1). This is followed by the model that has only the Agent Preference as a predictor (38%). The posterior mean differences of the Agent Preference predictor show an effect in the centroparietal region where the N400 is usually located (Figure 5E). Surprisal shows a stronger effect in the posterior region (Figure 5F). Both the surprisal and the Agent Preference conditions are well supported, with substantial amounts of the probability mass of the posterior distribution below zero for both conditions (Figure 5G and 5H). The estimated effect size of the Agent Preference is a bit less than half (strongest mean estimate Δ*μ*V = −0.46) of the one of surprisal (strongest mean estimate Δ*μ*V = −0.99), suggesting that the difference is not as pronounced as in German. The support is equally strong for both (strongest effects with both close to 1, *P*(|Δ*μ*V| > 0) > 0). Focusing on the electrodes with the strongest effects, the pattern is similar but with a stronger difference between the two conditions (estimated mean effect for Agent Preference at electrode Cz Δ*μ*V = −0.49, *P*(|Δ*μ*V| > 0) = 0.85; estimated mean effect for surprisal at electrode CP5 Δ*μ*V = −1.65, *P*(|Δ*μ*V| > 0) = 0.98; cf. Supporting Information S7.

##### Basque.

The model including RoBERTa-based surprisal and the Agent Preference condition leverages the highest weight (53%), which is followed by the one including LSTM-based surprisal and the Agent Preference condition (29%) (see panel “Basque” in Figure 3). The RoBERTa model also performed better than the LSTM model in the grammaticality test (Figure 1). But there, the GPT-2 model performed still far better while it does not predict EEG results well.

In both conditions, the best-fitting model estimates negative *μ*V difference values across a large part of the scalp coordinates. The estimated relative difference of the Agent Preference effect (strongest mean estimate Δ*μ*V = −1.33) is a bit more than half of that of surprisal (strongest mean estimate Δ*μ*V = −2.43), diminishing the difference slightly more than in Hindi. The support for the estimates is equally strong, with *P*(|Δ*μ*V| > 0) close to 1 in both conditions (Figure 5K and 5L). The single-electrode analyses confirm these results (estimated mean effect for Agent Preference at electrode Cz Δ*μ*V = −0.963, *P*(|Δ*μ*V| > 0) = 0.98; estimated mean effect for surprisal at electrode P8 Δ*μ*V = −1.76, *P*(|Δ*μ*V| > 0) = 0.99).

#### Differences

Consistently across languages, the models with both the Agent Preference condition and surprisal have considerably better predictive performance than simpler models. Thus, the Agent Preference is indeed required to accurately capture the EEG signals, and correspondingly, surprisal is not sufficient to capture the Predicate N400 observed with German, Hindi and Basque speakers. There are some interesting differences between the languages, however. For German, surprisal estimated by the LSTMs was a better predictor of the EEG signal than surprisal estimated by the transformer models. For both Basque and Hindi, the models including surprisal estimated by transformers yield a better model fit.

We further find different effect sizes and support for the predictors. In German, the Agent Preference principle has a smaller (only about one-sixth of the estimated effect size of surprisal) and less supported effect, which indicates that surprisal captures most of the variance in the EEG signal. In Hindi, the estimated effect size of the Agent Preference principle is a bit less than half of the one of surprisal, showing that surprisal is a stronger predictor than the Agent Preference, but the picture is more balanced than for German. In Basque, the estimated effect size of the Agent Preference is a bit more than half that of surprisal, that is, the relative effect size of the Agent Preference is slightly higher than in Hindi and much higher than in German.

At first sight, this result seems in conflict with the picture that emerges from the model stacking. In Hindi, the second highest-weighted model only contains the Agent Preference predictor, whereas in Basque, the second highest-weighted model contains both the Agent Preference and surprisal (LSTM) as predictors. This seems at odds with a relatively slightly lower effect of the Agent Preference in Hindi compared to Basque, a difference that is even stronger in the single-electrode analysis. However, the contradiction is resolved by considering the way in which the weights are allocated by model stacking. In Hindi much of the variance is already explained by surprisal in the top-ranking model, so that the next best contribution to prediction comes from the Agent Preference. The difference between the two predictors is weaker in Basque and so the next best contribution does not come from a single-predictor model but from one where surprisal is estimated by a different model (LSTM instead of RoBERTa).

## DISCUSSION

We asked whether probabilistic linguistic information is sufficient to characterise human language processing or whether additional principles are needed. To this end, we turned to what we call the Predicate N400, an effect that has been observed with sentences such as *… dass Julia alle grüssen* (“that they all greet Julia”), but not with *… dass Julia alle grüsst* (“that Julia greets them all”). We formalised the probabilistic linguistic information grounded in language experience in terms of a specific version of Surprisal Theory, according to which higher surprisal, as estimated by language models, correlates with a larger N400 amplitude. We contrasted Surprisal Theory with a theory claiming that the N400 amplitude difference reflects a processing principle at the macro-role level: the Agent Preference, which predicts an N400 whenever a verb disambiguates an NP to the dispreferred patient role, independently of statistical distributions in the input.

We estimated surprisal with three different language model architectures and compared the extent to which surprisal estimates capture the Predicate N400 both qualitatively (predicting the presence of the amplitude difference) and quantitively (predicting actual amplitude differences).

### An Agent Preference and Surprisal Are Both Needed to Capture the Predicate N400

Our qualitative analysis indicates that verb surprisal tends to be higher in sentences in which an N400 is expected under the hypothesis of an Agent Preference. While this would appear to support Surprisal Theory, the evidence is not as crisp. Especially for Basque, even though surprisals estimated by models are in line with the EEG results, this is true only in the ambiguous condition (Figure 2). With the Basque GPT-2 model, surprisal estimates also predict a Predicate N400 in the unambiguous condition. This is in contradiction to the experimental results, where the N400 was found to be reversed. The amplitude was higher in agent-initial sentences, a result that Isasi-Isasmendi et al. (2024) ascribe to the specific distribution of case markers (see Basque in the EEG Experiments section). In Hindi, GPT2-based surprisal estimates predict an additional contrast between the perfective versus imperfective conditions which was not found in the EEG experiments.

Thus, our qualitative findings suggest that probabilistic linguistic information cannot fully capture the Predicate N400. This is confirmed by our second analysis (see the Predicting N400 Amplitudes section), which shows that the Agent Preference is necessary in addition to surprisal to successfully predict N400 amplitudes. In all three languages (Hindi, Basque, and German), the top-ranking model includes both surprisal and the Agent Preference as predictors (Figure 3).

What do the respective contributions of surprisal and the Agent Preference imply for the Predicate N400? The Agent Preference account differs from Surprisal Theory in two major aspects. First, the Agent Preference targets processing at the level of macro-roles, that is, the preference applies to semantic roles in a general way, independent of the lexical specifics of a predicate-argument combination and independently of how well an argument approximates the agent prototype (Dowty, 1991). By contrast, surprisal is based on specific lexical choices and therefore targets predicate-argument combinations at the level of micro-roles (e.g., the “eater” and the “object being eaten,” rather than the agent and the patient of “eat”). Second, the Agent Preference account assumes that the bias is independent of linguistic experience. By contrast, Surprisal Theory, as operationalised here, is based on language experience and thus implies that the Predicate N400 is the product of a learned mechanism that arises in tandem with the acquisition of the language.

Therefore, our results show that humans process predicate-argument structures not only at the level of micro-roles but also at the level of macro-roles, where there appears to be a distinct tendency to interpret initial, ambiguous NPs as agents. The contribution of surprisal indicates that the Agent Preference may be modulated by specific predicate-argument combination and the probabilistic contingencies of the particular language being processed.

### Agent Preference in the Predicate N400

The Agent Preference has been considered a universal principle that is likely grounded in general event cognition (V. A. D. Wilson et al., 2022) or in linguistic event conceptualisation (e.g., in terms of minimal structures; Bornkessel & Schlesewsky, 2006; Bornkessel-Schlesewsky & Schlesewsky, 2009). Research on event cognition has shown that agents play a role that is distinct from other participants when we apprehend events. When viewing two-participant events, subjects spontaneously extract participants and their corresponding roles from brief depictions (less than 100 ms Dobel et al., 2007; Hafri et al., 2013; Hafri et al., 2018; Isasi-Isasmendi et al., 2023), fixating on the agent earlier (Gerwien & Flecken, 2016; Isasi-Isasmendi et al., 2023; Sauppe & Flecken, 2021; F. Wilson et al., 2011) and longer (Cohn & Paczynski, 2013) than on the patient. The parallels found between human sentence processing and event cognition raise the possibility that the Agent principle is also recruited during event cognition and is potentially shared with other primates (V. A. D. Wilson et al., 2022).

An alternative account derives the Agent Preference from an agent-initial principle in production. Specifically, the Production-Distribution-Comprehension Theory (MacDonald, 2013) assumes that comprehenders expect an agent in initial position because of a bias for agent-initial utterances that has been found in production data (Futrell et al., 2015; Goldin-Meadow et al., 2008; Schouwstra & de Swart, 2014). Like the Surprisal Theory, this theory predicts that the Predicate N400 can be derived from the probabilistic information that comprehenders learn from patterns in language use alone. In conflict with this prediction, our findings suggest that N400 amplitudes can be explained only when probabilistic information is enriched by an explicit Agent Preference that is not derived from usage patterns. Moreover, the Production-Distribution-Comprehension Theory fails to account for the Predicate N400 found in Äiwoo, an OVS language, where usage patterns directly violate an agent-initial principle in production. Similarly, signers of emerging sign languages follow a human-initial and not agent-initial principle in their production (Meir et al., 2017), although we are not aware of EEG experiments on the Predicate N400 in these languages. Finally, we note that the use of overt NPs varies greatly across languages (Bickel, 2003; Stoll & Bickel, 2009). This is at odds with the robustness of the Agent Preference against cross-linguistic variation in usage patterns.

### Contributions of the Agent Preference and Surprisal Vary Between Languages

The varying effect sizes across languages can be interpreted based on their grammatical differences. Different languages have different affordances, and so the experience with a particular language likely influences the processing of predicate-argument structures. This is reflected in our results, where the contribution of the predictors (Agent Preference or surprisal) varied across languages (Figure 2). The German surprisal estimates (especially when estimated by transformer models) were much more in line with the EEG results than the estimates in the other languages (see Qualitative Comparison Between Surprisal Estimates and EEG Results in the Results section). Moreover, surprisal has a much stronger effect on the N400 amplitude than the Agent Preference condition in German and the effect is also much more strongly supported, although the Agent Preference is still needed to provide the best model fit. In Basque and Hindi, the surprisal estimates did not fully mirror the EEG results in the qualitative comparison (Figure 2), and this was confirmed when predicting amplitudes, where the Agent Preference was needed for good *μ*V prediction (Figures 3 and Figure 5).

These difference between languages can be explained by the corresponding case systems (Table 2). German usually marks patients with case (accusative or dative) which in turn makes unmarked agents more predictable, hence easier to learn from probabilistic linguistic information. This is different in Hindi and Basque where unmarked agents are less common because agents are marked by ergative case. For Hindi, we predicted that the effect will be mixed due to the split case system, while for Basque Agent Preference will be the dominant predictor. Our results partially confirm this prediction: Compared to surprisal, the effect of the Agent Preference is weaker in Hindi than in Basque, although the difference is small in the whole-scalp (GAM) analysis and more noticeable only in the single electrode analysis.

An alternative explanation of the differences between languages invokes the fact that the Basque and Hindi transformer models were trained on smaller data sets than German and that the training data sets varied across languages. Such differences are particularly important for our study because, unlike in the German experiment, in the Hindi and Basque experiments, disambiguation relied on the selectional preferences and the argument structures of lexical verbs. It is plausible that more training data are needed so that a model can predict such properties of lexical items. However, the transformer models all passed our grammaticality test, and this makes it unlikely that the quality of the Basque and Hindi models is lower. Any quality difference emerges only in the LSTMs. These passed the grammaticality test only in German and, consistent with this, they received no weight in predicting EEG amplitudes in Hindi, and less weight in Basque.

### The Agent Preference and Surprisal From the View of Predictive Coding Theory

The interaction of the Agent Preference and language experience can be explained in a neurobiologically plausible theory based on predictive coding and free energy minimisation in the brain (Bornkessel-Schlesewsky & Schlesewsky, 2019; Clark, 2013; Friston, 2010). From the perspective of this theory, brains are “prediction engines” that constantly engage in matching incoming, sensory information with top-down predictions. Possible prediction errors are minimised in a hierarchical generative model instantiated within a bidirectional cascade of cortical processing (Clark, 2013). Representations of the world consist of probabilities that are induced by such hierarchical generative models, and these models are updated in a Bayesian fashion, so that the input and prediction errors inform future predictions (Clark, 2013; Constant et al., 2022; Knill & Pouget, 2004; Perconti & Plebe, 2020; Su et al., 2023). The N400 has been associated with the processing of probabilistic linguistic information and model updating (Kuperberg & Jaeger, 2016; Lindborg et al., 2023; Rabovsky et al., 2018). According to the descriptive model of Bornkessel-Schlesewsky and Schlesewsky (2019, section N400 Effects Reflect Precision-Weighted Prediction Errors), it reflects “precision-weighted prediction errors,” with information that more strongly determines sentence interpretation in a given language assigned higher precision weighting (Bornkessel-Schlesewsky & Schlesewsky, 2020).

Since predictive coding integrates the processing of any kind of information in a single theoretical framework, an approach along these lines can incorporate both principles: one (probabilistic linguistic information) that is based on the experience with a particular language and another (Agent Preference) that is universal and potentially recruited in the processing of events.

Considering the brain as a “Bayesian brain” that updates its priors based on previous prediction errors raises the question of why the Agent Preference could not ever be overridden by linguistic input. Particularly for Basque and Hindi speakers, we may ask why they still show an Agent Preference despite the high frequency of patient-initial sentences. A possible answer comes from the specific ways in which general cognitive mechanisms, such as those driven by the Agent Preference, interact with language-specific processing mechanisms. For a language like Basque or Hindi, where the canonical word order is agent-initial, an Agent Preference may not be overridden; the overall probabilistic signal from agent-initial sentences is too strong. This is strikingly different for an OVS language like Äiwoo, where the Agent Preference is indeed overriden when the role-ambiguous initial NP has nonhuman reference (Sauppe et al., 2023), or for Chinese, where it is overriden for inanimate referents when an agent-initial interpretation is pragmatically extremely marked (Wang et al., 2009). Varying affordances across languages result in different processing behaviours, and in the present case, the Agent Preference principle may be resistant to the language input in German, Basque, and Hindi, but not for inanimate NPs in Äiwoo and Chinese.

### Predictive Coding and Language Models

Even though they are loosely inspired by neural connections in the brain (Perconti & Plebe, 2020; Rumelhart et al., 1986; Rumelhart & McClelland, 1987), artificial neural network models are considered neurobiologically unrealistic models both at the level of implementation in the human brain (McClelland & Botvinick, 2020; Rosenbaum, 2022; Thomas & McClelland, 2008) as well as in regard to their functional similarity to human language processing (Arehalli et al., 2022; Arehalli & Linzen, 2020) and learning (Stevenson & Merlo, 2022; Warstadt & Bowman, 2022). This notwithstanding, the engagement in constant next-word prediction is an important functional property shared between language models and human sentence processing (Goldstein et al., 2022).

However, in line with our findings, several studies have revealed shortcomings of surprisal as a predictor of eye-tracking and EEG signals (Arehalli et al., 2022; Arehalli & Linzen, 2020; Brennan & Hale, 2019; Nelson et al., 2017; Slaats & Martin, 2023; van Schijndel & Linzen, 2018; E. Wilcox et al., 2021). For instance, E. Wilcox et al. (2021) showed that language models underestimate the difference in difficulty of processing grammatical versus ungrammatical sentences. A similar finding is observed with garden-path effects which surprisal sytematically underestimates and fails to predict their relative severity across different constructions (van Schijndel & Linzen, 2021). Adding further processing principles into language models may thus be conducive to creating neurobiologically more realistic models, in line with other recent suggestions for enriching language models (Su et al., 2023; E. Wilcox et al., 2021).

Such an endeavour would further allow us to disentangle different processing principles and their respective contribution across languages. In the present study we demonstrated that the Agent Preference is a necessary principle but that its effect differs across languages. Building such mechanisms into models might improve their inductive biases (van Schijndel et al., 2019; E. Wilcox et al., 2021) and reduce the amount of data needed for a model to acquire linguistic structures. Moreover, integrating neurobiological principles like the Agent Preference could play well together with recent “down-scaling” efforts to create functionally more plausible language models (Huebner et al., 2021; Warstadt & Bowman, 2022). Confirmation for down-scaled approaches also comes from evidence showing that models trained on realistic amounts of data can predict functional magnetic resonance imaging (blood oxygen level dependent) responses (Hosseini et al., 2022). A different approach for modelling the neurobiology of language is suggested by Slaats and Martin (2023), who argue that surprisal should be used as a cue representing distributional information in a model combined with other mechanisms.

Our results further suggest that it is important to compare different neural network architectures, despite the fact that transformer-based language models have been shown to predict EEG signals better than RNNs (Michaelov et al., 2021). In our German results, LSTMs are better at predicting the N400 amplitudes (Figure 5). For Basque and Hindi, by contrast, surprisal values derived from bidirectional transformer models yield a better fit. Curiously, GPT-2, despite yielding good results in the grammaticality test (see Figure 1), never leverages most weight in the model stacking. This may be due to different reasons for each language. In German and Hindi, GPT-2 may overestimate the difference between agent- and patient-initial word orders. In Basque, the EEG results show an N400 with agentive subjects in the unambiguous condition, while GPT-2 shows the converse predictions and RoBERTa seems to be agnostic about it. More cross-linguistic computational work is needed to assess the reasons for these differences.

The unequal performance of RNNs can be explained by the frequency of overt agents in these languages. In German, the frequent presence of an agent argument before the verb is likely to yield structures that are more easily predictable by a sequential model like an RNN. For Basque and Hindi, where overt agents are often dropped, accessing the morphosyntactic information in previous and upcoming units may be more conducive to accurate word prediction. The weights learned during training may then be helpful during testing when the model only has access to the previous words.

Thus, comparing different architectures may reveal processing differences across languages that are driven by systematic differences in usage patterns. While a German speaker might be able to more strongly rely on sequential integration of upcoming units to build dependencies, a Basque or Hindi speaker might have to directly access preceding units kept in memory, similar to the attention mechanism in transformers.

### Outlook

While the language models that we tested do not capture the amplitudes of the Predicate N400 sufficiently well, the question arises whether this is because surprisal on its own is insufficient to provide the best predictor for human sentence processing or because the estimation of surprisal has a shortcoming. In the present work we have estimated surprisal with the best available language models for this task at hand and have validated their performance in the grammaticality task. Improving these models is important but challenging in the absence of a gold standard, that is, of the true surprisal that a subject experiences in a specific tasks. Every observational or experimental estimate comes with assumptions and constraints, as is indeed also the case for the N400 amplitudes measured in EEG experiments. For example, observational methods like cloze probability tasks, are less suitable for modeling online sentence processing measurements since the cloze task is carried out offline (Michaelov et al., 2021).

An important issue is that the language models are generally trained on written texts whereas humans frequently engage with spoken language. Future research may thus benefit from training language models on data sets that better reflect the linguistic reality of a human. This will be possible once corpora of spoken and signed language of sufficient size are available.

Another open question is how to best conceptualise the Agent Preference predictor. An alternative to our present approach is to reconceptualise this predictor as a gradient instead of a binary principle. Previous experiments have shown that semantic features of the referents, such as animacy or noun-specific properties, result in stronger or weaker reanalysis effects (Frenzel et al., 2015; Gennari, 2008; Mak et al., 2002; Wang et al., 2012). Thus, the Predicate N400 could be captured by only a subset of the semantic features entailed by a prototypical agent role, and these subsets might be easier to learn from probabilistic linguistic information.

The fact that surprisal is, indeed, an essential predictor in all three languages has implications for the design of experiments on language comprehension. We suggest that it may be useful to routinely account for surprisal in experiments on language comprehension. This is particularly necessary to disentangle different drivers or mechanisms behind processing patterns and to interpret results by taking into account the linguistic reality of humans.

## CONCLUSION

Our study demonstrates that both probabilistic linguistic information (surprisal) and the Agent Preference principle contribute to capturing the EEG signal in the processing of predicate-argument structures. Given the striking centrality of agents in both the processing of sentences and the processing of events, it is plausible that the Agent Preference is rooted in general principles of event cognition, possibly continuing a preference shared with nonhuman primates and other animals. Incorporating such universal processing principles may be conducive to building neurobiologically more plausible models as well as to disentangling different processing principles and their contribution across languages.

Our study further shows that processing principles may operate differently across languages: the importance of the Agent Preference in predicting the Predicate N400 compared to that of surprisal correlates with the structure in each language. Thus, an essential endeavour to advance our models of human sentence processing is to integrate a wider range of typologically diverse languages. Furthermore, our findings hinge on the assumption that the model-based surprisals are sufficiently accurate representations of human linguistic experience. Thus, in order to draw more final conclusions about the respective contribution of the Agent Preference principle and surprisal, more research will be needed to build language models of spontaneous spoken or signed data and to seek ways of improving their performance while staying at realistic levels of data size.

## ACKNOWLEDGMENTS

We thank Erik Ringen and Chundra Cathcart for support with the statistical analysis. We also thank Roger Levy and two anonymous reviewers for valuable comments on an earlier version of this paper.

## FUNDING INFORMATION

Balthasar Bickel, National Center of Competence Evolving Language, Award ID: No. 51NF40_180888. Balthasar Bickel, Swiss National Science Foundation Grant, Award ID: 100015_182845. Paola Merlo, Swiss National Science Foundation Grant, Award ID: TMAG-1_209426/1. Ina Bornkessel-Schlesewsky, Centre of Excellence in Future Low-Energy Electronics Technologies, Australian Research Council (https://dx.doi.org/10.13039/501100019891), Award ID: FT160100437.

## AUTHOR CONTRIBUTIONS

**Eva Huber**: Conceptualization: Equal; Data curation: Lead; Formal analysis: Lead; Investigation: Lead; Methodology: Lead; Project administration: Lead; Software: Lead; Validation: Lead; Visualization: Lead; Writing – original draft: Lead; Writing – review & editing: Supporting. **Sebastian Sauppe**: Investigation: Supporting; Methodology: Supporting; Resources: Equal; Software: Supporting; Writing – review & editing: Supporting. **Arrate Isasi-Isasmendi**: Resources: Equal; Writing – review & editing: Supporting. **Ina Bornkessel-Schlesewsky**: Resources: Equal; Writing – review & editing: Supporting. **Paola Merlo**: Conceptualization: Supporting; Funding acquisition: Equal; Methodology: Supporting; Supervision: Equal; Writing – review & editing: Supporting. **Balthasar Bickel**: Conceptualization: Equal; Formal analysis: Supporting; Funding acquisition: Equal; Methodology: Supporting; Supervision: Equal; Validation: Supporting; Visualization: Supporting; Writing – review & editing: Lead.

## DATA AND CODE AVAILABILITY STATEMENT

Data and analysis scripts are available from https://osf.io/hbj67.

## Supplementary Material



## TECHNICAL TERMS

Agent Preference: A bias towards the more agent-like participant as opposed to the more patient-like participant in the comprehension of language and the observations of events.
Predicate N400: Effect that has been observed when a predicate, typically a verb, disambiguates the role of a preceding noun phrase.
Semantic Role: The underlying relationship of an argument and its predicate, which can be conceptualised at the level of macro-roles (agent vs. patient) or at the level of micro-roles (hitter vs. hittee).
Ergative: A case marker that signals the agent role of a noun phrase.
Linguistic surprisal: The information conveyed by any linguistic event defined in bits: *S*(*x*_*i*_) = −log_2_*p*(*x*_*i*_|*h*_*i*−1_).
Language models: Language models (based on artificial neural networks) estimate the probability distribution over a sequence of words by predicting future input based on previous input (in the case of next-word prediction as implemented in recurrent neural networks or GPT-2) or by predicting masked input (in the case of masked language modelling as implemented in BERT).
Bayesian surprise: Measures how any sensory input affects an observer in terms of the difference between prior and posterior beliefs.
Predictive coding: A theory on how the brain actively constructs explanations for the causes of its sensory inputs by constantly testing an internal model’s hypothesised sensory predictions against actual sensory input.
