# Supplementary material for: Surprisal From Language Models Can Predict ERPs in Processing Predicate-Argument Structures Only if Enriched by an Agent Preference Principle
Source: Neurobiol Lang (Camb). 2024 Apr 1;5(1):167–200. doi: 10.1162/nol_a_00121 (PMC11025647; doi:10.1162/nol_a_00121)
Supplement: Supplementary file 1 [file nol-5-1-167-s001.pdf]

Supplementary Information for "Surprisal from language models can predict ERPs in processing predicate-argument structures only if enriched by an Agent Preference principle"

Eva Huber[a,b,+], Sebastian Sauppe[a,b], Arrate Isasi-Isasmendi[a,b], Ina Bornkessel-Schlesewsky[c], Paola Merlo[d,e], and Balthasar Bickel[a,b]

[a]Department of Comparative Language Science, University of Zurich, Affolternstrasse 56, 8050 Zurich, Switzerland
[b]Center for the Interdisciplinary Study of Language Evolution, University of Zurich, Affolternstrasse 56, 8050 Zurich, Switzerland
[c]Cognitive Neuroscience Laboratory, Australian Research Centre for Interactive and Virtual Environments, University of South Australia, GPO Box 2471, Adelaide, SA 5001, Australia
[d]Department of Linguistics, University of Geneva, 5 Rue De-Candolle, 1211 Genève 4, Switzerland
[e]University Center for Computer Science

August 29, 2023

Contents

1 Language Models **2**

2 Analysis 1: Predicting Surprisal **14**

3 Analysis 2: Predicting ERPs for German **117**

4 Analysis 2: Predicting ERPs for Hindi **149**

5 Analysis 2: Predicting ERPs for Basque **179**

6 Residual Slopes in ERP models **212**

7 Single Electrode Analyses **228**

1 Language Models

S1: Estimating Surprisals with Language Models*

Eva Huber Sebastian Sauppe Arrate Isasi-Isasmendi
Ina Bornkessel-Schlesewsky Paola Merlo Balthasar Bickel

August 29, 2023

S1 Language Models

We extract the surprisal values from two different types of models: 1) a Long-Short-Term-Model (LSTM) which is a variant of a Recurrent Neural Network (RNN) and 2) a transformer-based architecture.

S1.1 LSTMs

We train a two-layer uni-directional LSTM for which we use the code provided by Gulordava et al. (2018). We tune several hyperparameters: size of the hidden layers (200 or 650, *HL size*), the batch size (20 or 64 (*batch size*)), dropout (0.1 or 0.2, *dropout*) and the learning rate (5 or 10, *LR*). Similarly to Gulordava et al. (2018), large hidden layers yield lower perplexity values.

S1.1.1 Training Data

language	data source	nr tokens train	nr tokens valid	nr tokens test
Hindi	Wikipedia dump and OSCAR (webcrawl) (Ortiz Suárez, Sagot, and Romary 2019)	100,362,268	15,918,937	9,169,274
Basque	Basque Multimedia Corpus (Agerri et al. 2020)	195,715,895	24,481,432	24,462,787
German	wikipedia dump of 2018, cleaned and provided by *t-systems-on-site-services-gmbh*	189,647,309	23,718,786	23,686,839

S1.1.2 Hyperparameter Tuning

*This document was rmarkdown::rendered from an Rmd script main_scripts/language_models.Rmd available at https://osf.io/hbj67

Model	Language	Epochs	HL size	batch_size	dropout	LR	valid_ppl	test_ppl
1	Hindi	10	200	20	0.1	5	61.68	46.8
2	Hindi	10	200	20	0.1	10	65.25	50.38
3	Hindi	10	200	20	0.2	5	67.38	52.11
4	Hindi	10	200	20	0.2	10	72.11	57.07
5	Hindi	10	200	64	0.1	5	60.51	46.11
6	Hindi	10	200	64	0.1	10	60.92	46.49
7	Hindi	10	200	64	0.2	5	66.72	51.82
8	Hindi	10	200	64	0.2	10	67.17	52.19
9	Hindi	10	650	20	0.1	5	41.64	30.34
10	Hindi	10	650	20	0.1	10	47.89	35.92
11	Hindi	10	650	20	0.2	5	43.58	32.42
12	Hindi	10	650	20	0.2	10	53.85	41.59
13	Hindi	10	650	64	0.1	5	41.57	30.36
14	Hindi	10	650	64	0.1	10	**40.7**	**29.86**
15	Hindi	10	650	64	0.2	5	43.61	32.27
16	Hindi	10	650	64	0.2	10	44.44	33.1
1	German	10	200	20	0.1	5	51.37	51.24
2	German	10	200	20	0.1	10	54.76	54.63
3	German	10	200	20	0.2	5	54.38	54.25
4	German	10	200	20	0.2	10	58.28	58.09
5	German	10	200	64	0.1	5	50.92	50.8
6	German	10	200	64	0.1	10	51.61	51.48
7	German	10	200	64	0.2	5	54.28	54.14
8	German	10	200	64	0.2	10	54.44	54.31
9	German	10	650	20	0.1	5	**38.52**	**38.46**
10	German	10	650	20	0.1	10	40.25	40.17
11	German	10	650	20	0.2	5	39.45	39.37
12	German	10	650	20	0.2	10	41.09	41.01
13	German	10	650	64	0.1	5	38.76	38.68
14	German	10	650	64	0.1	10	38.58	38.51
15	German	10	650	64	0.2	5	39.64	39.56
16	German	10	650	64	0.2	10	39.35	39.28
1	Basque	10	200	20	0.1	5	104.31	103.84
2	Basque	10	200	20	0.1	10	109.79	109.29
3	Basque	10	200	20	0.2	5	110.12	109.62
4	Basque	10	200	20	0.2	10	115.98	115.47
5	Basque	10	200	64	0.1	5	103.37	102.91
6	Basque	10	200	64	0.1	10	103.95	103.5
7	Basque	10	200	64	0.2	5	109.57	109.07
8	Basque	10	200	64	0.2	10	109.88	109.38
9	Basque	10	650	20	0.1	5	82.61	82.29
10	Basque	10	650	20	0.1	10	86.12	85.75
11	Basque	10	650	20	0.2	5	84.54	84.18
12	Basque	10	650	20	0.2	10	87.97	87.59
13	Basque	10	650	64	0.1	5	82.98	82.61
14	Basque	10	650	64	0.1	10	**82.48**	**82.14**
15	Basque	10	650	64	0.2	5	84.97	84.6
16	Basque	10	650	64	0.2	10	84.2	83.85

The perplexities for German and Hindi are comparable, however the perplexity for Basque is considerably higher. Recent work showed that more powerful language models estimate N400 amplitudes more accurately

Wilcox et al. (2020), however Goodkind and Bicknell (2018) has also found that at least for eye-tracking data, even basic n-gram models estimate surprisal values show linear relationships to reading time.

S1.2 Transformers: BERT/RoBERTa

- Hindi: *Indic-Transformers Hindi BERT*
  - provided by Neural Space, accessed on HuggingFace
  - trained on ~3 GB of monoloingual training corpus, mainly taken from OSCAR
  - BERT architecture
- Basque: *ixa-ehu/roberta-eus-euscrawl-large-cased*
  - published as part of Artetxe et al. (2022) and accessed on HuggingFace
  - trained on the Euscrawl, a corpus created using tailored crawling from Basque sites (12,528k documents and 423M tokens
  - architecture RoBERTa, an architecture that builds on BERT and modifies key hypoerparamters. Next-sentence pretraining objective is removed and the training is done with much larger mini-batches and learning rates, leading to performance gains and reducing computational expense
- German: *bert-base-german-cased*
  - provided by deepset, accessed on HuggingFace
  - trained on Wikipedia dump, OpenLegalData dump and news articles (~12 GB of data)
  - BERT architecture

S1.3 Transformers: GPT-2

- German: *dbmdz/german-gpt2*
  - Wikipedia dump, EU Bookshop corpus, Open Subtitles, CommonCrawl, ParaCrawl and News Crawl.(16GB and 2,350,234,427 tokens)
- Basque: *ClassCat/gpt2-small-basque-v2*
  - Subset of CC-100/eu and Oscar (creators do not disclose data size)

S1.3.1 Hindi GPT-2

No pretrained Hindi GPT-2 model is available, which is why we trained our own model. We follow Vries and Nissim (2021)'s training scheme, which is well-suited if computational power and training data is scarce. The training process starts from the English GPT-2 model and unfolds over two steps. In the first, we trained a new tokenizer on the Hindi training dataset and then only the lexical embeddings are trained while the other layers are frozen. In the second, step the entire model is trained. The model was trained on four NVIDIA A100 Tensor Core GPUs for four days. The training data consists of Hindi wikipedia and crawl data from OSCAR (Ortiz Suárez, Sagot, and Romary 2019), resulting in approximately 6.5 GB of data.

S1.4 Grammaticality Test

In order to evaluate the language models' knowledge of the language, we test their capability of distinguishing between sentences with grammatically incorrect word orders and sentences with grammatically correct word orders.

The permuted (i.e. grammatically incorrect) sentences consist of several variants of syntactically incorrect word orders. We calculate the average word surprisal per sentence. After that the surprisal of the correct

sentence is subtracted from the surprisal of the incorrect sentences (*Delta Surprisal*). Thus, if a model assigns higher surprisal to "wrong" sentences (which is desired from a language model), the final value would be positive.

The files including grammatical and ungrammatical can be found in the folder 'grammaticality_test'. The results can be found in the folder 'result'.

S1.4.1 German

```
de.lstm <- read.csv('../results/grammaticality_german_lstm.txt',
                    sep='\t', col.names = c('sent_id', 'word', 'surprisal', 'scrambled')) %>%
  separate(sent_id, c('sent_id', 'cond'), sep=':') %>%
  unite(sent_id, c('sent_id', 'scrambled'), sep=':', remove=F)

# calculate mean surprisal value per sentence and subtract correct
# sentence from incorrect sentence

de.lstm.mean <- de.lstm %>%
  group_by(sent_id, scrambled,cond) %>%
  summarise(surprisal_mean = mean(surprisal)) %>%
  ungroup() %>%
  separate(sent_id, c('sent_id', 'scrambled'), sep=':') %>%
  pivot_wider(names_from = scrambled, values_from = surprisal_mean) %>%
  mutate(diff = false - true) %>%
  mutate(model = 'LSTM')

de.bert <- read.table('../results/grammaticality_german_bert.csv',
                      sep='\t', col.names = c('sent_id', 'word', 'surprisal')) %>%
  filter(surprisal > 0) %>%
  separate(sent_id, c('sent_id', 'cond', 'original'), sep=':')

# calculate mean surprisal value per sentence and subtract correct sentence
# from incorrect sentence
de.bert.mean <- de.bert %>%
        group_by(sent_id, cond, original) %>%
  summarise(surprisal_mean = mean(surprisal)) %>%
  ungroup() %>%
  pivot_wider(names_from = original, values_from = surprisal_mean) %>%
  mutate(diff = false - true) %>%
  mutate(model = 'BERT/RoBERTa')


de.gpt <- read.table('../results/grammaticality_german_gpt.csv', sep='\t',
                     header=T) %>%
  rename(word = cond) %>%
  separate(sent_id, c('sent_id', 'cond', 'original'), sep=':') %>%
  filter(surprisal > 0)

# calculate mean surprisal value per sentence and subtract correct sentence
# from incorrect sentence
de.gpt.mean <- de.gpt %>%
        group_by(sent_id, cond, original) %>%
  summarise(surprisal_mean = mean(surprisal)) %>%
```

```
  ungroup() %>%
  pivot_wider(names_from = original, values_from = surprisal_mean) %>%
  mutate(diff = false - true) %>%
  mutate(model = 'GPT-2')
```

S1.4.2 Hindi

```
hi.lstm <-  read.csv('../results/grammaticality_hindi_lstm.txt', sep='\t',
                     col.names = c('sent_id', 'word', 'surprisal', 'scrambled')) %>%
    separate(sent_id, c('sent_id', 'cond'), sep=':') %>%
  unite(sent_id, c('sent_id', 'scrambled'), sep=':', remove=F)

# calculate mean surprisal value per sentence and subtract correct
# sentence from incorrect sentence
hi.lstm.mean <- hi.lstm %>%
  group_by(sent_id, scrambled,cond) %>%
  summarise(surprisal_mean = mean(surprisal)) %>%
  ungroup() %>%
  separate(sent_id, c('sent_id', 'scrambled'), sep=':') %>%
  pivot_wider(names_from = scrambled, values_from = surprisal_mean) %>%
  mutate(diff = false - true) %>%
  mutate(model = 'LSTM')


hi.bert <- read.table('../results/grammaticality_hindi_bert.csv',
                      sep='\t', col.names = c('sent_id', 'cond', 'scrambled', 'word', 'surprisal')) %>%
  filter(surprisal > 0)

# calculate mean surprisal value per sentence and subtract correct sentence
# from incorrect sentence
hi.bert.mean <- hi.bert %>%
      group_by(sent_id, cond, scrambled) %>%
  summarise(surprisal_mean = mean(surprisal)) %>%
  ungroup() %>%
  pivot_wider(names_from = scrambled,
              values_from = surprisal_mean) %>%
  mutate(diff = false - true) %>%
  mutate(model = 'BERT/RoBERTa')


hi.gpt <- read.table('../results/grammaticality_hindi_gpt.csv',
                     sep='\t', col.names = c('sent_id', 'cond', 'scrambled', 'word', 'surprisal')) %>%
  filter(surprisal > 0)

# calculate mean surprisal value per sentence and subtract correct sentence
# from incorrect sentence
hi.gpt.mean <- hi.gpt %>%
      group_by(sent_id, cond, scrambled) %>%
  summarise(surprisal_mean = mean(surprisal)) %>%
  ungroup() %>%
  pivot_wider(names_from = scrambled, values_from = surprisal_mean) %>%
```

```
  mutate(diff = false - true) %>%
  mutate(model = 'GPT-2')
```

S1.4.3 Basque

```
eu.lstm <-  read.csv('../results/grammaticality_basque_lstm.txt', sep='\t',
                     col.names = c('sent_id', 'word', 'surprisal', 'scrambled')) %>%
    separate(sent_id, c('sent_id', 'cond'), sep=':') %>%
  unite(sent_id, c('sent_id', 'scrambled'), sep=':', remove=F)

# calculate mean surprisal value per sentence and subtract correct
# sentence from incorrect sentence
eu.lstm.mean <- eu.lstm %>%
  group_by(sent_id, scrambled,cond) %>%
  summarise(surprisal_mean = mean(surprisal)) %>%
  ungroup() %>%
  separate(sent_id, c('sent_id', 'scrambled'), sep=':') %>%
  pivot_wider(names_from = scrambled, values_from = surprisal_mean) %>%
  mutate(diff = false - true) %>%
  mutate(model = 'LSTM')


eu.roberta <-  read.table('../results/grammaticality_basque_roberta.csv',
                          sep='\t', col.names = c('sent_id', 'cond', 'scrambled',
                                                  'word', 'surprisal')) %>%
  filter(surprisal > 0)
# calculate mean surprisal value per sentence and subtract correct
# sentence from incorrect sentence

eu.roberta.mean <- eu.roberta %>%
  group_by(sent_id, cond, scrambled) %>%
  summarise(surprisal_mean = mean(surprisal)) %>%
  ungroup() %>%
  pivot_wider(names_from = scrambled, values_from = surprisal_mean) %>%
  mutate(diff = false - true) %>%
  mutate(model = 'BERT/RoBERTa')

eu.gpt<-  read.table('../results/grammaticality_basque_gpt.csv', sep='\t',
                     col.names = c('sent_id', 'cond', 'scrambled', 'word', 'surprisal')) %>%
  filter(surprisal > 0)
# calculate mean surprisal value per sentence and subtract correct sentence
# from incorrect sentence
eu.gpt.mean <- eu.gpt %>%
    group_by(sent_id, cond, scrambled) %>%
  summarise(surprisal_mean = mean(surprisal)) %>%
  ungroup() %>%
  pivot_wider(names_from = scrambled, values_from = surprisal_mean) %>%
  mutate(diff = false - true) %>%
  mutate(model = 'GPT-2')
```

S1.4.4 Model

For each language and language model, we fit a Bayesian regression model to estimate the Delta Surprisal difference, controlling for differences in sentences.

```
de.lstm.mean$sent_id <- paste(de.lstm.mean$sent_id, de.lstm.mean$cond, sep='_')
de.bert.mean$sent_id <- paste(de.bert.mean$sent_id, de.bert.mean$cond, sep='_')
de.gpt.mean$sent_id <- paste(de.gpt.mean$sent_id, de.gpt.mean$cond, sep='_')

de.model.lstm <- brm(diff ~ 1 + (1|sent_id),
                     data=de.lstm.mean,
                     file = '../models/grammaticality_de_lstm')

de.model.tr <- brm(diff ~ 1 + (1|sent_id),
                   data=de.bert.mean,
                   file = '../models/grammaticality_de_bert')
de.model.gpt <-brm(diff ~ 1 + (1|sent_id),
                   data=de.gpt.mean,
                   file = '../models/grammaticality_de_gpt')

samps.de.lstm <- posterior_samples(de.model.lstm)  %>% mutate(model = 'LSTM') %>%
  mutate(language = 'German') %>% select(language, model, b_Intercept)
samps.de.bert <- posterior_samples(de.model.tr) %>% mutate(model = 'BERT/RoBERTa') %>%
  mutate(language = 'German')  %>% select(language, model, b_Intercept)
samps.de.gpt <- posterior_samples(de.model.gpt) %>% mutate(model = 'GPT-2') %>%
  mutate(language = 'German')  %>% select(language, model, b_Intercept)

hi.lstm.mean$sent_id <- paste(hi.lstm.mean$sent_id, hi.lstm.mean$cond, sep='_')
hi.bert.mean$sent_id <- paste(hi.bert.mean$sent_id, hi.bert.mean$cond, sep='_')
hi.gpt.mean$sent_id <- paste(hi.gpt.mean$sent_id, hi.gpt.mean$cond, sep='_')


hi.model.lstm <- brm(diff ~ 1 + (1|sent_id),
                      data = hi.lstm.mean,
                      file = '../models/grammaticality_hi_lstm')

hi.model.tr <- brm(diff ~ 1 + (1|sent_id),
                   data = hi.bert.mean,
                   file = '../models/grammaticality_hi_tr')

hi.model.gpt <- brm(diff ~ 1 + (1|sent_id),
                    data = hi.gpt.mean,
                    file = '../models/grammaticality_hi_gpt')

samps.hi.lstm <- posterior_samples(hi.model.lstm)  %>% mutate(model = 'LSTM') %>%
  mutate(language = 'Hindi')  %>% select(language, model, b_Intercept)
samps.hi.bert <- posterior_samples(hi.model.tr) %>% mutate(model = 'BERT/RoBERTa') %>%
  mutate(language = 'Hindi') %>% select(language, model, b_Intercept)
samps.hi.gpt <- posterior_samples(hi.model.gpt) %>% mutate(model = 'GPT-2') %>%
  mutate(language = 'Hindi') %>% select(language, model, b_Intercept)


eu.lstm.mean$sent_id <- paste(eu.lstm.mean$sent_id, eu.lstm.mean$cond, sep='_')
eu.roberta.mean$sent_id <- paste(eu.roberta.mean$sent_id, eu.roberta.mean$cond, sep='_')
```

```
eu.gpt.mean$sent_id <- paste(eu.gpt.mean$sent_id, eu.gpt.mean$cond, sep='_')


eu.model.lstm <- brm(diff ~ 1 + (1|sent_id),
                      data = eu.lstm.mean,
                     file = '../models/grammaticality_eu_lstm')

eu.model.tr <- brm(diff ~ 1 + (1|sent_id),
                      data = eu.roberta.mean,
                   file = '../models/grammaticality_eu_tr')
eu.model.gpt <- brm(diff ~ 1 + (1|sent_id),
                      data = eu.gpt.mean,
                    file = '../models/grammaticality_eu_gpt')

samps.eu.lstm <- posterior_samples(eu.model.lstm) %>% mutate(model = 'LSTM') %>%
  mutate(language = 'Basque') %>% select(language, model, b_Intercept)
samps.eu.bert <- posterior_samples(eu.model.tr) %>% mutate(model = 'BERT/RoBERTa') %>%
  mutate(language = 'Basque') %>% select(language, model, b_Intercept)
samps.eu.gpt <- posterior_samples(eu.model.gpt) %>% mutate(model = 'GPT-2') %>%
  mutate(language = 'Basque') %>% select(language, model, b_Intercept)
```

```
all.diff <- rbind(samps.de.lstm, samps.de.bert, samps.de.gpt,
                     samps.hi.lstm, samps.hi.bert, samps.hi.gpt,
                     samps.eu.lstm, samps.eu.bert, samps.eu.gpt)

all.diff$language <- factor(all.diff$language, levels=c("German","Hindi","Basque"))

diff.plot <- all.diff %>%
  ggplot(aes(x=b_Intercept, fill= model, color = model)) +
  stat_slab(alpha = .5, normalize = 'groups') +
  stat_pointinterval(position = position_dodge(width = .2, preserve = "single"), .width = c(.50,.80,.90
  ylab("") +
  xlab(expression(paste(Delta," Surprisal between ungrammatical and grammatical sentence"))) +
  scale_color_brewer(palette = 'Set2') +
  scale_fill_brewer(palette = 'Set2') +
  geom_vline(xintercept = 0, linetype="dashed") +
  theme(panel.grid = element_blank(), panel.border = element_blank(),
        axis.line.x = element_line(size = 1), axis.ticks.y = element_blank(),
        axis.text.y = element_blank(), #element_text(family = 'Arial Unicode MS', size = 12),
        axis.title.x = element_text( size = 16),
        axis.text.x =  element_text(size = 12),
        plot.title = element_text(size=22),
        plot.background = element_rect(fill = "transparent", colour = NA),
        strip.text.x = element_text(size = 15)) +
  facet_wrap(~language,  ncol = 1) +
  scale_y_discrete(expand = c(0, 0.5))


diff.plot
```

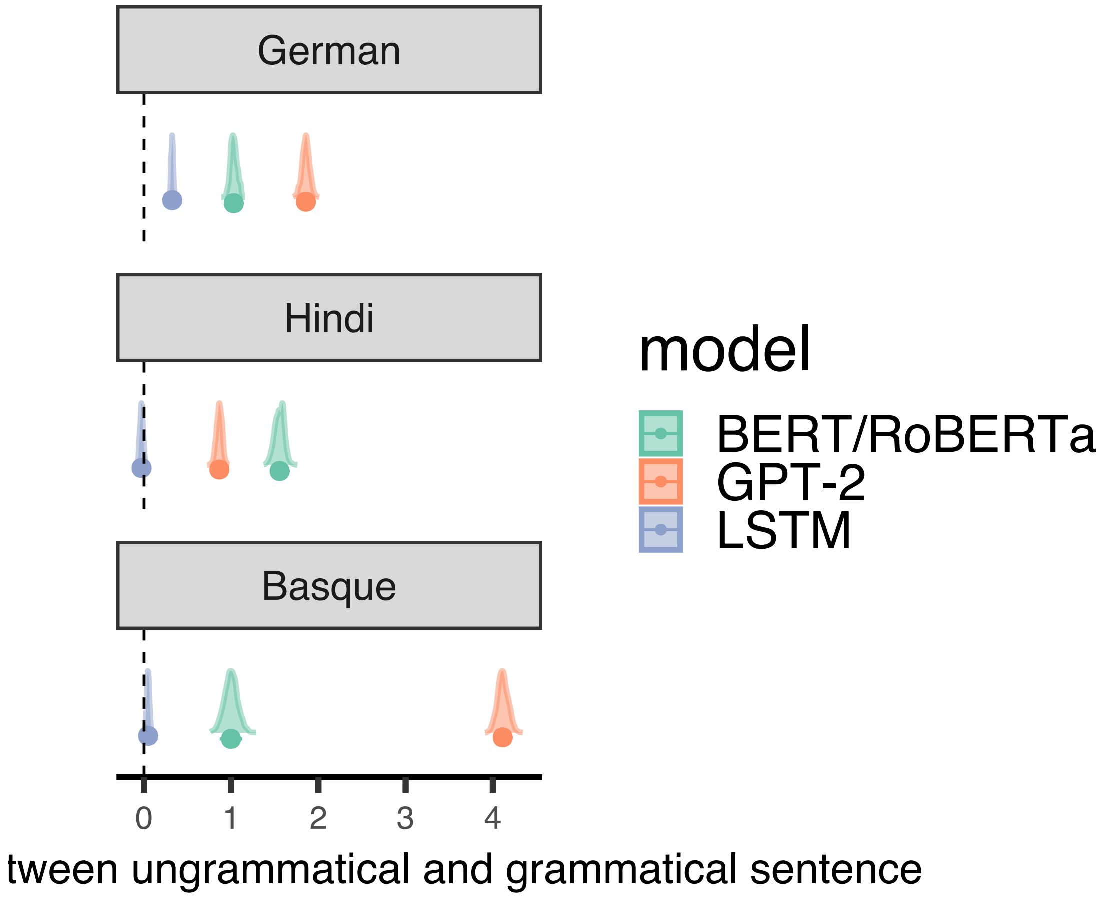


```
ci <- rbind(samps.de.lstm %>%
                      bayestestR::ci(ci=0.89, method='HDI')   %>%
                      mutate(language = 'German') %>%
                      mutate(model = 'LSTM'),
                   samps.de.bert %>%
                     bayestestR::ci(ci=0.89, method='HDI') %>%
                     mutate(language = 'German') %>%
                     mutate(model = 'BERT'),
                   samps.de.gpt %>%
                     bayestestR::ci(ci=0.89, method='HDI')%>%
                     mutate(language = 'German') %>%
                     mutate(model = 'GPT'),
                   samps.hi.lstm %>%
                     bayestestR::ci(ci=0.89, method='HDI') %>%
                     mutate(language = 'Hindi') %>%
                     mutate(model = 'LSTM'),
                   samps.hi.bert %>%
                      bayestestR::ci(ci=0.89, method='HDI') %>%
                     mutate(language = 'Hindi') %>%
                     mutate(model = 'BERT'),
                   samps.hi.gpt %>%
                     bayestestR::ci(ci=0.89, method='HDI') %>%
                     mutate(language = 'Hindi') %>%
                     mutate(model = 'GPT'),
                   samps.eu.lstm %>%
```

```
                      bayestestR::ci(ci=0.89, method='HDI') %>%
                      mutate(language = 'Basque') %>%
                      mutate(model = 'LSTM'),
                    samps.eu.bert %>%
                      bayestestR::ci(ci=0.89, method='HDI')%>%
                      mutate(language = 'Basque') %>%
                      mutate(model = 'RoBERTa'),
                    samps.eu.gpt %>%
                      bayestestR::ci(ci=0.89, method='HDI') %>%
                      mutate(language = 'Basque') %>%
                      mutate(model = 'GPT'))

means <- rbind(samps.de.lstm %>%
                       summarise(Mean = mean(b_Intercept)),
                    samps.de.bert %>%
                     summarise(Mean = mean(b_Intercept)),
                    samps.de.gpt %>%
                     summarise(Mean = mean(b_Intercept)),
                    samps.hi.lstm %>%
                      summarise(Mean = mean(b_Intercept)),
                    samps.hi.bert %>%
                      summarise(Mean = mean(b_Intercept)),
                    samps.hi.gpt %>%
                     summarise(Mean = mean(b_Intercept)),
                    samps.eu.lstm %>%
                     summarise(Mean = mean(b_Intercept)),
                    samps.eu.bert %>%
                      summarise(Mean = mean(b_Intercept)),
                    samps.eu.gpt %>%
                     summarise(Mean = mean(b_Intercept)))

means.ci <- cbind(ci, means)
col.order <- c('model', 'language', 'Mean', 'CI_low', 'CI_high')
means.ci <-means.ci[,col.order]

means.ci %>%
  kable(., digits = 1:4, booktabs = T, linesep = "",
        caption = "Means and 89 percent credibility intervals  of Delta Surprisal
        for each language and language model") %>%
  kable_styling(font_size = 10,latex_options = 'hold_position')
```

Table S3: Means and 89 percent credibility intervals of Delta Surprisal for each language and language model

model	language	Mean	CI_low	CI_high
LSTM	German	0.323	0.3044	0.3
BERT	German	1.031	0.9754	1.1
GPT	German	1.856	1.7931	1.9
LSTM	Hindi	-0.029	-0.0585	0.0
BERT	Hindi	1.554	1.4625	1.6
GPT	Hindi	0.864	0.8115	0.9
LSTM	Basque	0.047	0.0251	0.1
RoBERTa	Basque	0.997	0.8630	1.1
GPT	Basque	4.116	4.0140	4.2

References

Agerri, Rodrigo, Iñaki San Vicente, Jon Ander Campos, Ander Barrena, Xabier Saralegi, Aitor Soroa, and Eneko Agirre. 2020. "Give Your Text Representation Models Some Love: The Case for Basque." In, 4781–88. European Language Resources Association. https://aclanthology.org/2020.lrec-1.588.

Artetxe, Mikel, Itziar Aldabe, Rodrigo Agerri, Olatz Perez-de-Viñaspre, and Aitor Soroa. 2022. "Does Corpus Quality Really Matter for Low-Resource Languages?" *arXiv Preprint arXiv:2203.08111*.

Goodkind, Adam, and Klinton Bicknell. 2018. "Predictive Power of Word Surprisal for Reading Times Is a Linear Function of Language Model Quality." In *Proceedings of the 8th Workshop on Cognitive Modeling and Computational Linguistics (CMCL 2018)*, 10–18.

Gulordava, Kristina, Piotr Bojanowski, Edouard Grave, Tal Linzen, and Marco Baroni. 2018. "Colorless Green Recurrent Networks Dream Hierarchically." *arXiv Preprint arXiv:1803.11138*.

Ortiz Suárez, Pedro Javier, Benoît Sagot, and Laurent Romary. 2019. "Asynchronous Pipelines for Processing Huge Corpora on Medium to Low Resource Infrastructures." In, edited by Piotr Bański, Adrien Barbaresi, Hanno Biber, Evelyn Breiteneder, Simon Clematide, Marc Kupietz, Harald Lüngen, and Caroline Iliadi, 9–16. Proceedings of the Workshop on Challenges in the Management of Large Corpora (CMLC-7) 2019. Cardiff, 22nd July 2019. Mannheim: Leibniz-Institut für Deutsche Sprache. https://doi.org/10.14618/ids-pub-9021.

Vries, Wietse de, and Malvina Nissim. 2021. "As Good as New. How to Successfully Recycle English GPT-2 to Make Models for Other Languages." In *Findings of the Association for Computational Linguistics: ACL-IJCNLP 2021*, 836–46. Online: Association for Computational Linguistics. https://doi.org/10.18653/v1/2021.findings-acl.74.

Wilcox, Ethan Gotlieb, Jon Gauthier, Jennifer Hu, Peng Qian, and Roger Levy. 2020. "On the Predictive Power of Neural Language Models for Human Real-Time Comprehension Behavior." *arXiv Preprint arXiv:2006.01912*.

2 Analysis 1: Predicting Surprisal

S2: Study 1: Predicting Surprisal*

Eva Huber Sebastian Sauppe Arrate Isasi-Isasmendi
Ina Bornkessel-Schlesewsky Paola Merlo Balthasar Bickel

August 29, 2023

Contents

S3 Predicting Surprisal **1**
S3.1 Data Preprocessing . . . 1
S3.2 Models: Estimates of Surprisal . . . 10
S3.3 Posterior Distributions . . . 52
S3.4 Plots . . . 74
S3.5 T-tests . . . 95

REPRODUCIBILITY STATEMENT: All of the models included here were fitted on an M1 processor using R Version 4.2.2 and brms version 2.14.0.

S3 Predicting Surprisal

S3.1 Data Preprocessing

All files are preprocessed with '/data_preprocessing.R'.

The data below includes the predicted surprisal at each word in the stimuli estimated with LSTMs, (Ro)BERT(A) and a GPT.
Since we exclude stimuli with out-of-vocabulary items up to and including the target word (critical region) for LSTM models, we replaced the oov sentences with words that occur in the vocabulary and are appropriate replacements for the oov-words (called 'extended stimuli'). This is to ensure that the lower number of stimuli for LSTMs does not skew the results in any direction. For (Ro)BERT(A) and GPT, no original stimuli have to be excluded, but we nevertheless estimate the extended stimuli with RoBERTA as a proof of concept to ensure that the change in words does not lead to a change in surprisal values associated with the conditions. For each stimuli set, we extract the surprisal values at each word. For the models below, we only keep the word at the critical region, i.e. the surprisal value at the main verb. ### German

Original stimuli, surprisal estimated by LSTM

```
# some preprocessing

mapping <- read.csv('../stimuli/german_sent_id_mappings.csv', sep=',') %>% select(sent_id, new_sent)
info.original <- read.csv('../data_experiments/german_original_nouns_verbs.csv', sep='\t') %>%
  separate(cond, c('verb.type', 'first.NP'), sep='_', remove=F) %>%
  left_join(mapping, by='sent_id') %>%
  select(-sent_id) %>%
```

*This document was `rmarkdown::render`'ed from an `R` script 'main_scripts/analysis1' available at https://osf.io/hbj67

```
  dplyr::rename(sent_id = new_sent)

de.lstm.original <- read.csv('../results/german_LSTM_original_prep.csv' , sep='\t') %>%
  left_join(mapping, by='sent_id') %>%
  select(-sent_id) %>%
  dplyr::rename(sent_id = new_sent)

de.lstm.original.m <- de.lstm.original %>%
  filter(region %in% 'AUX') %>% # filter surprisal value at critical region
  left_join(info.original, by=c('sent_id','first.NP','verb.type'))
```

Extended stimuli, surprisal estimated by LSTM

```
de.lstm.extended <- read.csv('../results/german_LSTM_extended_prep.csv',
                              sep='\t') %>%
  left_join(mapping, by='sent_id') %>%
  select(-sent_id) %>%
  dplyr::rename(sent_id = new_sent)

de.lstm.extended.m <- de.lstm.extended %>%
  filter(region %in% 'AUX') %>% # filter surprisal value at critical region
  left_join(info.original, by=c('sent_id','first.NP','verb.type'))
```

Original stimuli, surprisal estimated by BERT

```
de.tr.original <- read.csv('../results/german_transformer_original_prep.csv',
                            sep='\t') %>%
  left_join(mapping, by='sent_id') %>%
  select(-sent_id) %>%
  dplyr::rename(sent_id = new_sent)

de.tr.original.m <- de.tr.original %>%
  filter(region %in% 'AUX') %>% # filter surprisal value at critical region
  left_join(info.original, by=c('sent_id','first.NP','verb.type'))
```

Extended stimuli, surprisal estimated by BERT

```
de.tr.extended <- read.csv('../results/german_transformer_extended_prep.csv',
                            sep='\t') %>%
  left_join(mapping, by='sent_id') %>%
  select(-sent_id) %>%
  dplyr::rename(sent_id = new_sent)

de.tr.extended.m <- de.tr.extended %>%
  filter(region %in% 'AUX') %>% # filter surprisal value at critical region
  left_join(info.original, by=c('sent_id','first.NP','verb.type'))
```

```
de.tr.extended.m$verb.type <- as.factor(paste(de.tr.extended.m$verb.type))
de.tr.extended.m$first.NP <- as.factor(paste(de.tr.extended.m$first.NP))
de.tr.extended.m$surprisal <- as.numeric(paste(de.tr.extended.m$surprisal))
de.tr.extended.m$sent_id <- as.factor(paste(de.tr.extended.m$sent_id))
```

```r
de.tr.extended.m$first.NP <- relevel(de.tr.extended.m$first.NP, ref='subject')
```

Original stimuli, surprisal estimated by GPT-2

```r
de.gpt.original <- read.csv('../results/german_gpt_original_prep.csv', sep='\t') %>%
  left_join(mapping, by='sent_id') %>%
  select(-sent_id) %>%
  dplyr::rename(sent_id = new_sent)

de.gpt.original.m <- de.gpt.original %>%
  separate(cond, c('verb.type', 'first.NP'), sep='_')

de.gpt.original.m$verb.type <- as.factor(paste(de.gpt.original.m$verb.type))
de.gpt.original.m$first.NP <- as.factor(paste(de.gpt.original.m$first.NP))
de.gpt.original.m$surprisal <- as.numeric(paste(de.gpt.original.m$surprisal))
de.gpt.original.m$sent <- as.factor(paste(de.gpt.original.m$sent_id))

de.gpt.original.m$first.NP <- relevel(de.gpt.original.m$first.NP, ref='subject')


contrasts(de.gpt.original.m$first.NP)
```

```
##         object
## subject      0
## object       1
```

```r
contrasts(de.gpt.original.m$verb.type)
```

```
##     dat
## acc   0
## dat   1
```

Extended stimuli, surprisal estimated by GPT-2

```r
de.gpt.extended <- read.csv('../results/german_gpt_extended_prep.csv',
                            sep='\t') %>%
  left_join(mapping, by='sent_id') %>%
  select(-sent_id) %>%
  dplyr::rename(sent_id = new_sent)

de.gpt.extended.m <- de.gpt.extended %>%
  separate(cond, c('verb.type', 'first.NP'), sep='_')

de.gpt.extended.m$verb.type <- as.factor(paste(de.gpt.extended.m$verb.type))
de.gpt.extended.m$first.NP <- as.factor(paste(de.gpt.extended.m$first.NP))
de.gpt.extended.m$surprisal <- as.numeric(paste(de.gpt.extended.m$surprisal))
de.gpt.extended.m$sent <- as.factor(paste(de.gpt.extended.m$sent_id))

de.gpt.extended.m$first.NP <- relevel(de.gpt.extended.m$first.NP, ref='subject')


contrasts(de.gpt.extended.m$first.NP)
```

```
##         object
## subject      0
## object       1
```

```
contrasts(de.gpt.extended.m$verb.type)
```

```
##     dat
## acc   0
## dat   1
```

Table S1 gives an overview of the number of stimuli per condition for each dataset and model. The number of stimuli is (almost) equal to the number of stimuli from the original experiment apart for the LSTM original dataset.

Table S1: Nr of Sentences per condition and dataset

Dataset	Models	Agent_1st_acc	Patient_1st_acc	Agent_1st_dat	Patient_1st_dat	total
original	LSTM	288	288	224	224	1024
extended	LSTM	1312	1312	1152	1152	4928
original	BERT	1344	1344	1344	1344	5376
extended	BERT	1328	1328	1328	1328	5312
original	GPT-2	336	336	336	336	1344
extended	GPT-2	332	332	332	332	1328

S3.1.1 Hindi

Original stimuli, surprisal estimated by LSTM

```
hi.lstm.original <- read.csv('../results/hindi_LSTM_original_prep.csv',
                             sep='\t')


hi.lstm.original.m <- hi.lstm.original %>%
  filter(region %in% 'VERB')


hi.lstm.original.m$ambiguity <- as.factor(paste(hi.lstm.original.m$ambiguity))
hi.lstm.original.m$aspect <- as.factor(paste(hi.lstm.original.m$aspect))
hi.lstm.original.m$surprisal <- as.numeric(paste(hi.lstm.original.m$surprisal))
hi.lstm.original.m$sent_id <- as.factor(paste(hi.lstm.original.m$sent_id))


contrasts(hi.lstm.original.m$ambiguity)
```

```
##       UNAMB
## AMB       0
## UNAMB     1
```

```
contrasts(hi.lstm.original.m$aspect)
```

```
##      PFV
## IPFV   0
## PFV    1
```

Extended stimuli, surprisal estimated by LSTM

```
hi.lstm.extended <- read.csv('../results/hindi_LSTM_extended_prep.csv',
                             sep='\t')


hi.lstm.extended.m <- hi.lstm.extended %>%
  filter(region %in% 'VERB')


hi.lstm.extended.m$ambiguity <- as.factor(paste(hi.lstm.extended.m$ambiguity))
hi.lstm.extended.m$aspect <- as.factor(paste(hi.lstm.extended.m$aspect))
hi.lstm.extended.m$surprisal <- as.numeric(paste(hi.lstm.extended.m$surprisal))
hi.lstm.extended.m$sent_id <- as.factor(paste(hi.lstm.extended.m$sent_id))


contrasts(hi.lstm.extended.m$ambiguity)
```

```
##       UNAMB
## AMB       0
## UNAMB     1
```

```
contrasts(hi.lstm.extended.m$aspect)
```

```
##      PFV
## IPFV   0
## PFV    1
```

Original stimuli, surprisal estimated by BERT

```
hi.tr.original <- read.csv('../results/hindi_transformer_original_prep.csv' ,
                           sep='\t')


hi.tr.original.m <- hi.tr.original %>%
  filter(region %in% 'VERB')


hi.tr.original.m$ambiguity <- as.factor(paste(hi.tr.original.m$ambiguity))
hi.tr.original.m$aspect <- as.factor(paste(hi.tr.original.m$aspect))
hi.tr.original.m$surprisal <- as.numeric(paste(hi.tr.original.m$surprisal))
hi.tr.original.m$sent_id <- as.factor(paste(hi.tr.original.m$sent_id))


contrasts(hi.tr.original.m$ambiguity)
```

```
##       UNAMB
## AMB       0
## UNAMB     1
```

```r
contrasts(hi.tr.original.m$aspect)
```

```
##      PFV
## IPFV   0
## PFV    1
```

Extended stimuli, surprisal estimated by BERT

```r
hi.tr.extended <- read.csv('../results/hindi_transformer_extended_prep.csv',
                           sep='\t')


hi.tr.extended.m <- hi.tr.extended %>%
  filter(region %in% 'VERB')


hi.tr.extended.m$ambiguity <- as.factor(paste(hi.tr.extended.m$ambiguity))
hi.tr.extended.m$aspect <- as.factor(paste(hi.tr.extended.m$aspect))
hi.tr.extended.m$surprisal <- as.numeric(paste(hi.tr.extended.m$surprisal))
hi.tr.extended.m$sent_id <- as.factor(paste(hi.tr.extended.m$sent_id))


contrasts(hi.tr.extended.m$ambiguity)
```

```
##       UNAMB
## AMB       0
## UNAMB     1
```

```r
contrasts(hi.tr.extended.m$aspect)
```

```
##      PFV
## IPFV   0
## PFV    1
```

Original stimuli, surprisal estimated by GPT-2

```r
hi.gpt.original <- read.csv('../results/hindi_gpt_original_prep.csv' ,
                            sep='\t') %>%
  mutate('ambiguity' = ifelse(grepl('A', cond), 'AMB', 'UNAMB')) %>%
  mutate('aspect' = ifelse(grepl('I', cond), 'IPFV', 'PFV'))


hi.gpt.original.m <- hi.gpt.original

hi.gpt.original.m$ambiguity <- as.factor(paste(hi.gpt.original.m$ambiguity))
hi.gpt.original.m$aspect <- as.factor(paste(hi.gpt.original.m$aspect))
hi.gpt.original.m$surprisal <- as.numeric(paste(hi.gpt.original.m$surprisal))
hi.gpt.original.m$sent_id <- as.factor(paste(hi.gpt.original.m$sent_id))
```

Extended stimuli, surprisal estimated by GPT-2

```r
hi.gpt.extended <- read.csv('../results/hindi_gpt_extended_prep.csv' ,
                            sep='\t') %>%
  mutate('ambiguity' = ifelse(grepl('A', cond), 'AMB', 'UNAMB')) %>%
  mutate('aspect' = ifelse(grepl('I', cond), 'IPFV', 'PFV'))


hi.gpt.extended.m <- hi.gpt.extended

hi.gpt.extended.m$ambiguity <- as.factor(paste(hi.gpt.extended.m$ambiguity))
hi.gpt.extended.m$aspect <- as.factor(paste(hi.gpt.extended.m$aspect))
hi.gpt.extended.m$surprisal <- as.numeric(paste(hi.gpt.extended.m$surprisal))
hi.gpt.extended.m$sent_id <- as.factor(paste(hi.gpt.extended.m$sent_id))
```

Table S2 gives an overview of the number of stimuli per condition for each dataset and model.

Table S2: Nr of Sentences per condition and dataset

Dataset	Models	Amb_IPFV	Unamb_IPFV	Amb_PFV	Unamb_IPFV.1	total
original	LSTM	23	23	29	41	75
extended	LSTM	47	45	48	52	140
original	BERT	60	60	60	60	180
extended	BERT	60	60	60	60	180
original	GPT-2	60	60	60	60	180
extended	GPT-2	60	60	60	60	180

S3.1.2 Basque

Original stimuli, surprisal estimated by LSTM

```
eu.lstm.original <- read.csv('../results/basque_LSTM_original_prep.csv',
                             sep='\t')%>%
  separate(sent_id, c('sent_id', 'cond_id'), sep='_')



eu.lstm.original.m <- eu.lstm.original %>%
  filter(region %in% 'Verb') %>% # filter surprisal value at critical region
  separate(cond, c('Ambiguity', 'Semantic.Role'), remove=F, sep='_')
```

Extended stimuli, surprisal estimated by LSTM

```
eu.lstm.extended <- read.csv('../results/basque_LSTM_extended_prep.csv',
                             sep='\t') %>%
  separate(sent_id, c('sent_id', 'cond_id'), sep='_')
#mutate(sent_id = str_replace(sent_id, '_', ':'))


eu.lstm.extended.m <- eu.lstm.extended %>%
  filter(region %in% 'Verb')  %>% # filter surprisal value at critical region
  separate(cond, c('Ambiguity', 'Semantic.Role'), remove=F, sep='_')
```

Original stimuli, surprisal estimated by RoBERTa

```
eu.tr.original <- read.csv('../results/basque_transformer_original_prep.csv',
                           sep='\t') %>% # mutate(sent_id = str_replace(sent_id, '_', ':')) %>%
  rename(cond = condition) %>%
  separate(sent_id, c('sent_id', 'cond_id'), sep='_')

eu.tr.original.m <- eu.tr.original %>%
  filter(region_nr == 5) %>% # filter surprisal value at critical region
  separate(cond, c('Ambiguity', 'Semantic.Role'), remove=F, sep='_')
```

Extended stimuli, surprisal estimated by RoBERTa

```
eu.tr.extended <- read.csv('../results/basque_transformer_extended_prep.csv',
                           sep='\t') %>%
  separate(sent_id, c('sent_id', 'cond_id'), sep='_') %>%
  rename(cond = condition)
# mutate(sent_id = str_replace(sent_id, '_', ':'))

eu.tr.extended.m <- eu.tr.extended %>%
  filter(region_nr == 5) %>% # filter surprisal value at critical region
  separate(cond, c('Ambiguity', 'Semantic.Role'), remove=F, sep='_')
```

Original stimuli, surprisal estimated by GPT-2

```r
eu.gpt.original <- read.csv('../results/basque_GPT_original_prep.csv',sep='\t') %>%
  separate(cond, c('Ambiguity', 'Semantic.Role'), sep='_') %>%
  separate(sent_id, c('sent_id', 'cond_it'), sep='_')

eu.gpt.original.m <- eu.gpt.original
eu.gpt.original.m$surprisal <- as.numeric(paste(eu.gpt.original.m$surprisal))
eu.gpt.original.m$Semantic.Role <- as.factor(paste(eu.gpt.original.m$Semantic.Role))
eu.gpt.original.m$Ambiguity <- as.factor(paste(eu.gpt.original.m$Ambiguity))
eu.gpt.original.m$sent_id <- as.factor(paste(eu.gpt.original.m$sent_id))
```

Extended stimuli, surprisal estimated by GPT

```r
eu.gpt.extended <- read.csv('../results/basque_GPT_extended_prep.csv',sep='\t') %>%
  separate(cond, c('Ambiguity', 'Semantic.Role'), sep='_') %>%
  separate(sent_id, c('sent_id', 'cond_it'), sep='_')

eu.gpt.extended.m <- eu.gpt.extended
eu.gpt.extended.m$surprisal <- as.numeric(paste(eu.gpt.extended.m$surprisal))
eu.gpt.extended.m$Semantic.Role <- as.factor(paste(eu.gpt.extended.m$Semantic.Role))
eu.gpt.extended.m$Ambiguity <- as.factor(paste(eu.gpt.extended.m$Ambiguity))
eu.gpt.extended.m$sent_id <- as.factor(paste(eu.gpt.extended.m$sent_id))
```

Table S3 gives an overview of the number of stimuli per condition for each dataset and model. The number of stimuli is (almost) equal to the number of stimuli from the original experiment apart for the LSTM original dataset. A small deviation of LSTM extended in number from transformer dataset sizes exists because replacement words were accidentally selected that were not in the model vocabulary.

Table S3: Nr of Sentences per condition and dataset

Dataset	Models	Amb_Patient	Amb_Agent	Unamb_Patient	Unamb_Agent	total
original	LSTM	86	72	85	72	315
extended	LSTM	190	190	190	189	759
original	RoBERTa	192	192	192	192	768
extended	RoBERTa	192	192	192	192	768
original	GPT-2	192	192	192	192	768
extended	GPT-2	192	192	192	192	768

S3.2 Models: Estimates of Surprisal

For each model and dataset, we fit a model to estimate surprisal. We use Bayesian hierarchical models to include the effect of conditions (semantic role of subject and ambiguity) and random intercepts and slopes for item ID. We check convergence by ensuring that Effective Sample Size $ESS > 100$ and diagnostic $\hat{R} < 1.05$. Additionally, we perform prior and posterior predictive checks to 1) compare whether the posterior distribution moved away from the prior distribution and 2) compare the predicted values to the observed data.

We perform leave-one-out with Pareto smoothed importance sampling using the `loo` package [@vehtari2017practical]. We further fit a baseline model to examine whether the conditions improve the model fit.

Lastly, in response to a reviewer's request, we replicate the results with a paired t-test for the original stimul only. ### German

Original stimuli, surprisal estimated by LSTM

```
prior.de.lstm.original <- c(
  prior(normal(0,1), class = b),
  prior(exponential(1), class = sigma),
  prior(exponential(1), class = sd)
)

prior.de.lstm.original.null  <- c(
  prior(exponential(1), class = sigma)
)

contrasts(de.lstm.original.m$first.NP)
```

```
##         object
## subject      0
## object       1
```

```
contrasts(de.lstm.original.m$verb.type)
```

```
##     dat
## acc   0
## dat   1
```

```
model.de.lstm.original <-
  brm(surprisal ~ verb.type * first.NP + (1+verb.type+first.NP|sent_id),
      family = gaussian,
      save_pars = save_pars(all = T),
      sample_prior = T,
      data = de.lstm.original.m,
      prior = prior.de.lstm.original,
      file = '../models/de.lstm.original',
      chains = 4, iter = 4000, warmup=1000, cores = 4,
      control = list(adapt_delta = 0.96, max_treedepth=15)
  )

model.de.lstm.original.prior <-
  brm(surprisal ~ verb.type * first.NP + (1+verb.type+first.NP|sent_id),
```

```
      family = gaussian,
      sample_prior = T,
      data = de.lstm.original.m,
      file = '../models/de.lstm.original.prior',
      prior = prior.de.lstm.original
  )

model.de.lstm.original.null <-
  brm(surprisal ~ 1 + (1|sent_id),
      family = gaussian,
      save_pars = save_pars(all = T),
      sample_prior = T,
      data = de.lstm.original.m,
      prior = prior.de.lstm.original.null ,
      file = '../models/de.lstm.original_null',
      chains = 4, iter = 4000, warmup=1000, cores = 4
  )


pp_check(model.de.lstm.original.prior, nsamples=1e2)
```

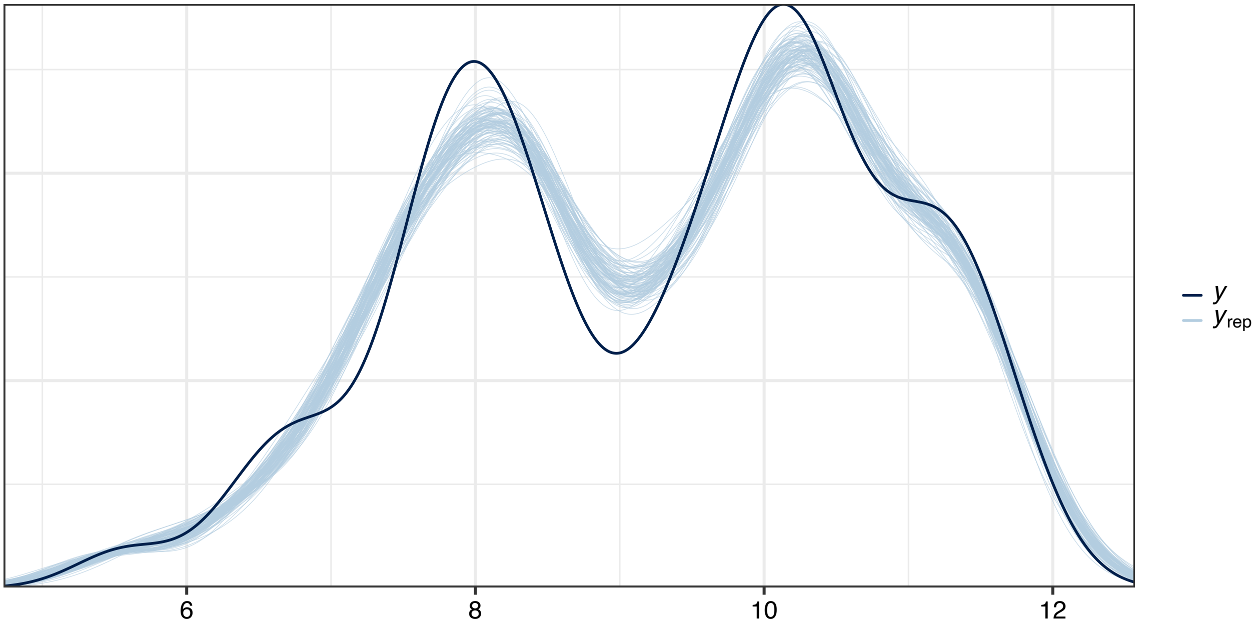


```
pp_check(model.de.lstm.original, nsamples=1e2)
```

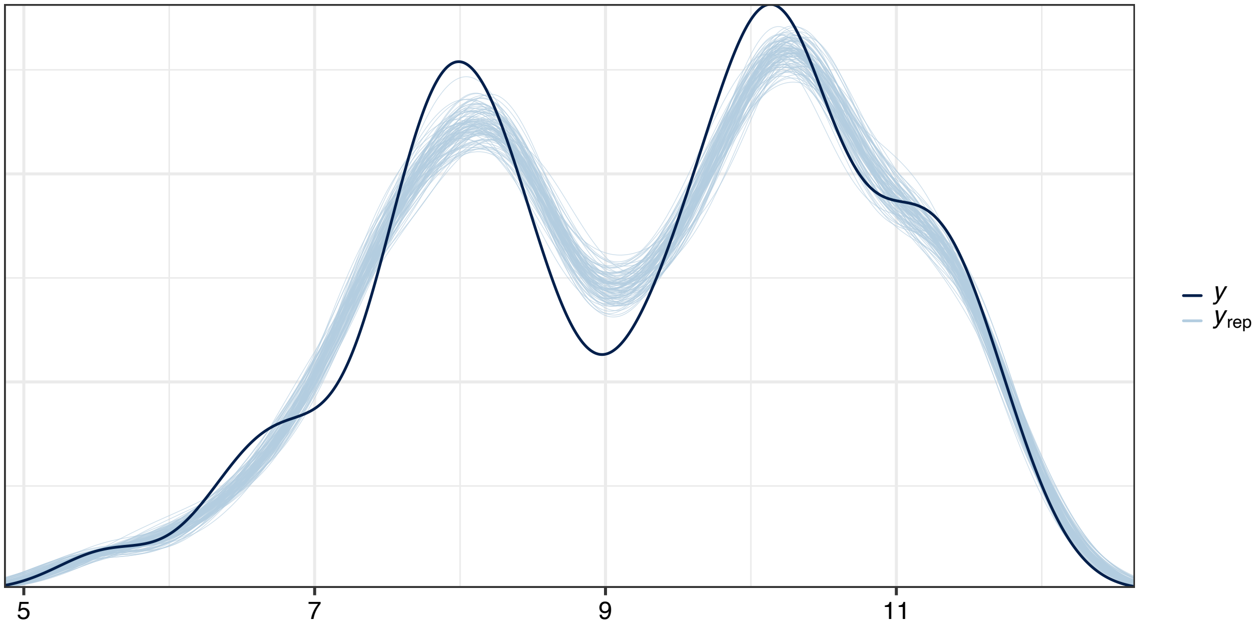


```
loo.m.de.lstm.orig <- loo(model.de.lstm.original)
loo.m.de.lstm.orig.null <- loo(model.de.lstm.original.null)

loo_model_weights(list("hypothesis" = loo.m.de.lstm.orig ,
                       "null" = loo.m.de.lstm.orig.null))
```

```
## Method: stacking
## ------
##            weight
## hypothesis 1.000
## null       0.000
```

```
loo_compare(list("hypothesis" = loo.m.de.lstm.orig,
                 "null" = loo.m.de.lstm.orig.null))
```

```
##            elpd_diff se_diff
## hypothesis     0.0       0.0
## null       -1827.9      24.9
```

Extended stimuli, surprisal estimated by LSTM

```
contrasts(de.lstm.extended.m$first.NP)
```

```
##         object
## subject      0
## object       1
```

```
contrasts(de.lstm.extended.m$verb.type)
```

```
##     dat
## acc   0
## dat   1
```

```
model.de.lstm.extended <-
  brm(surprisal ~ verb.type * first.NP + (1+verb.type+first.NP|sent_id),
      family = gaussian,
      save_pars = save_pars(all = T),
      sample_prior = T,
      data = de.lstm.extended.m,
      prior = prior.de.lstm.original,
      file = '../models/de.lstm.extended',
      chains = 4, iter = 4000, warmup=1000, cores = 4
  )

model.de.lstm.extended.prior <-
  brm(surprisal ~ verb.type * first.NP + (1+verb.type+first.NP|sent_id),
      family = gaussian,
      sample_prior = 'only',
      file = '../models/de.lstm.extended.prior',
      data = de.lstm.extended.m,
      prior = prior.de.lstm.original
  )

model.de.lstm.extended.null <-
  brm(surprisal ~ 1 + (1|sent_id),
      family = student(),
      save_pars = save_pars(all = T),
      sample_prior = T,
      data = de.lstm.extended.m,
      prior = prior.de.lstm.original.null,
      file = '../models/de.lstm.extended_null',
      chains = 4, iter = 4000, warmup=1000, cores = 4
  )


pp_check(model.de.lstm.extended.prior, nsamples=1e2)
```

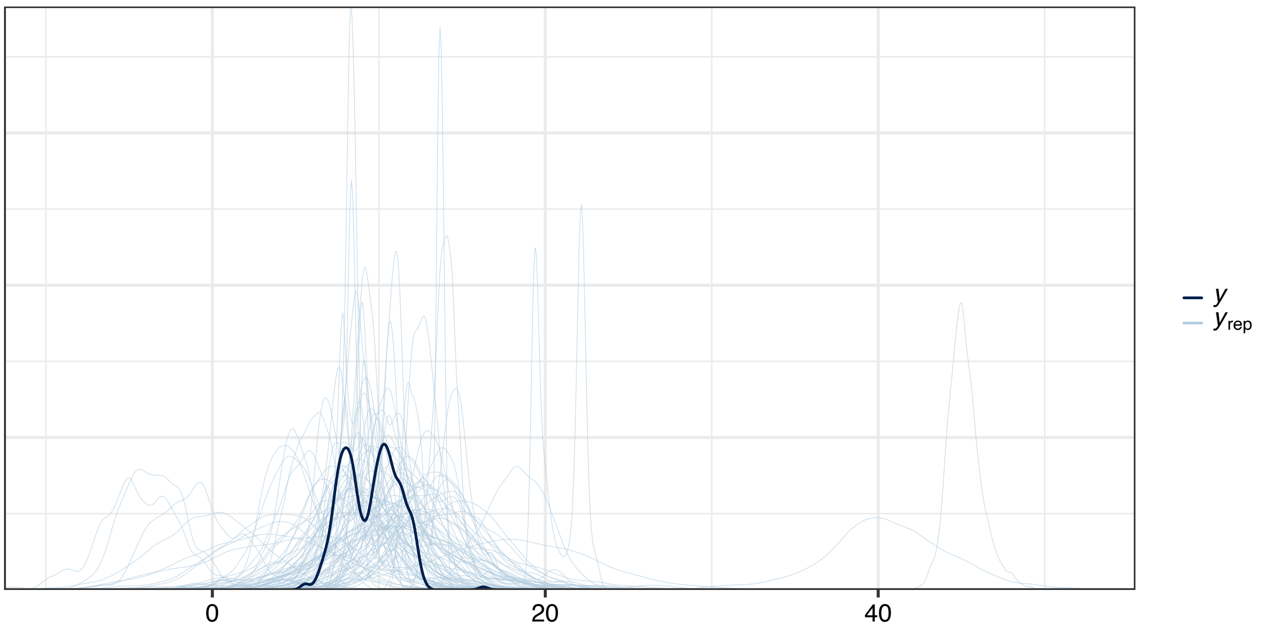

```r
pp_check(model.de.lstm.extended, nsamples=1e2)
```

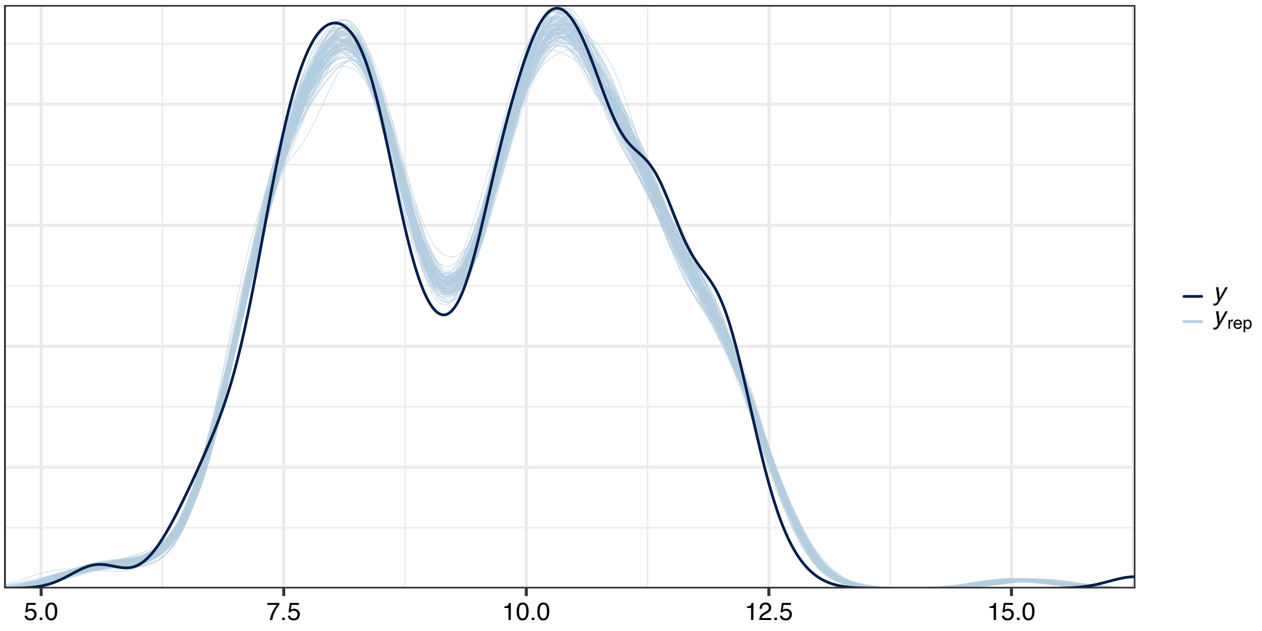


```r
loo.m.de.lstm.extended <- loo(model.de.lstm.extended)
loo.m.de.lstm.extended.null <- loo(model.de.lstm.extended.null)

loo_model_weights(list("hypothesis" = loo.m.de.lstm.extended ,
                       "null" = loo.m.de.lstm.extended.null))
```

```
## Method: stacking
## ------
##            weight
## hypothesis 0.983
## null       0.017
```

```r
loo_compare(list("hypothesis" = loo.m.de.lstm.extended,
                 "null" = loo.m.de.lstm.extended.null))
```

```
##            elpd_diff se_diff
## hypothesis     0.0       0.0
## null       -8502.1      85.7
```

Original stimuli, surprisal estimated by transformer

```r
contrasts(de.tr.original.m$first.NP)
```

```
##         object
## subject      0
## object       1
```

```r
contrasts(de.tr.original.m$verb.type)
```

```
##     dat
## acc   0
## dat   1
```

```r
model.de.tr.original <-
  brm(surprisal ~ verb.type * first.NP + (1+first.NP+verb.type|sent_id),
      family = gaussian,
      save_pars = save_pars(all = T),
      sample_prior = T,
      data = de.tr.original.m,
      file = '../models/de.tr.original',
      prior = prior.de.lstm.original,
      chains = 4, iter = 8000, warmup=1000, cores = 4,
      control = list(adapt_delta = 0.96, max_treedepth=15)
  )

model.de.tr.original.prior <-
  brm(surprisal ~ verb.type * first.NP + (1+first.NP+verb.type|sent_id),
      family = gaussian,
      sample_prior = 'only',
      data = de.tr.original.m,
      file = '../models/de.tr.original.prior',
      prior = prior.de.lstm.original
  )

model.de.tr.original.null <-
  brm(surprisal ~ 1+ (1|sent_id),
      family = gaussian,
      save_pars = save_pars(all = T),
      sample_prior = T,
      data = de.tr.original.m,
      prior = prior.de.lstm.original.null,
      file = '../models/de.tr.original_null',
      chains = 4, iter = 8000, warmup=1000, cores = 4,
      control = list(adapt_delta = 0.96, max_treedepth=15)
  )


pp_check(model.de.tr.original.prior, nsamples=1e2)
```

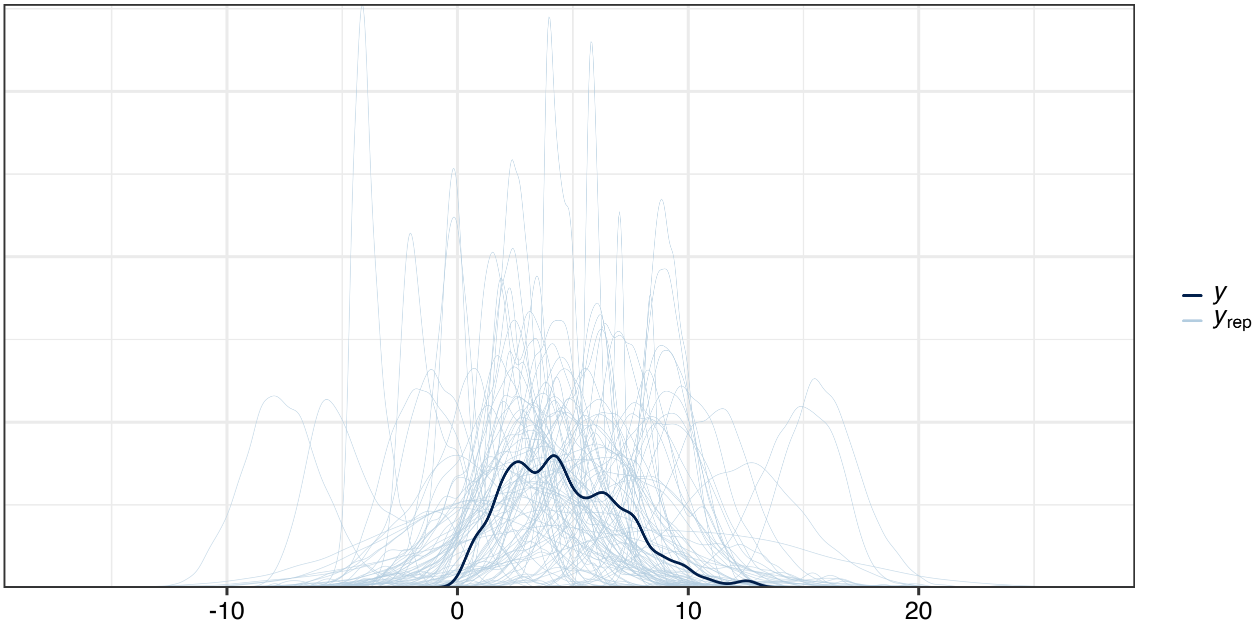


```
pp_check(model.de.tr.original, nsamples=1e2)
```

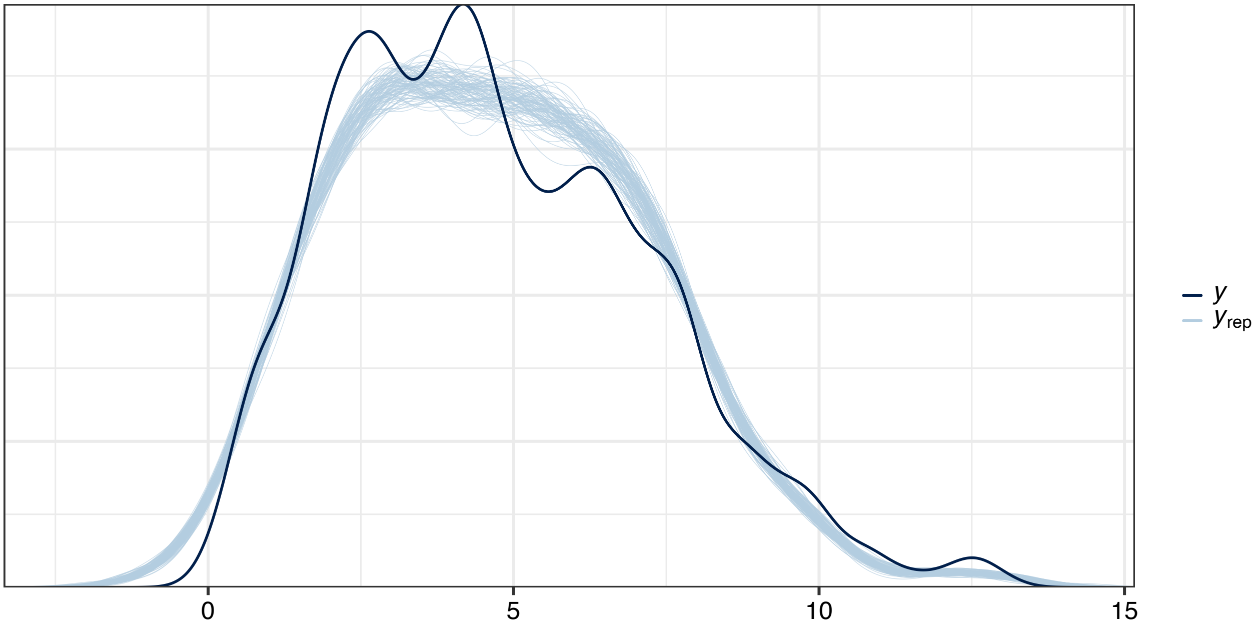


```
loo.m.de.tr.orig <- loo(model.de.tr.original)
loo.m.de.tr.orig.null <- loo(model.de.tr.original.null)

loo_model_weights(list("hypothesis" = loo.m.de.tr.orig ,
                       "null" = loo.m.de.tr.orig.null))
```

```
## Method: stacking
## ------
##            weight
## hypothesis 0.983
## null       0.017
```

```r
loo_compare(list("hypothesis" = loo.m.de.tr.orig,
                 "null" = loo.m.de.tr.orig.null))
```

```
##            elpd_diff se_diff
## hypothesis     0.0       0.0
## null       -5978.0      79.3
```

Extended stimuli, surprisal estimated by transformer

```r
contrasts(de.tr.extended.m$first.NP)
```

```
##         object
## subject      0
## object       1
```

```r
contrasts(de.tr.extended.m$verb.type)
```

```
##     dat
## acc   0
## dat   1
```

```r
model.de.tr.extended <-
  brm(surprisal ~ verb.type * first.NP + (1+first.NP+verb.type|sent_id),
      family = gaussian,
      save_pars = save_pars(all = T),
      sample_prior = T,
      data = de.tr.extended.m,
      file = '../models/de.tr.extended',
      prior = prior.de.lstm.original,
      chains = 4, iter = 8000, warmup=1000, cores = 4,
      control = list(adapt_delta = 0.96, max_treedepth=15)
  )

model.de.tr.extended.prior <-
  brm(surprisal ~ verb.type * first.NP + (1+first.NP+verb.type|sent_id),
      family = gaussian,
      sample_prior = 'only',
      file = '../models/de.tr.extended.prior',
      data = de.tr.extended.m,
      prior = prior.de.lstm.original
  )

model.de.tr.extended.null <-
  brm(surprisal ~ 1+ (1|sent_id),
      family = gaussian,
      save_pars = save_pars(all = T),
      sample_prior = T,
      data = de.tr.extended.m,
      prior = prior.de.lstm.original.null,
      file = '../models/de.tr.extended_null',
      chains = 4, iter = 8000, warmup=1000, cores = 4,
```

```
      control = list(adapt_delta = 0.96, max_treedepth=15)
  )


pp_check(model.de.tr.extended.prior, nsamples=1e2)
```

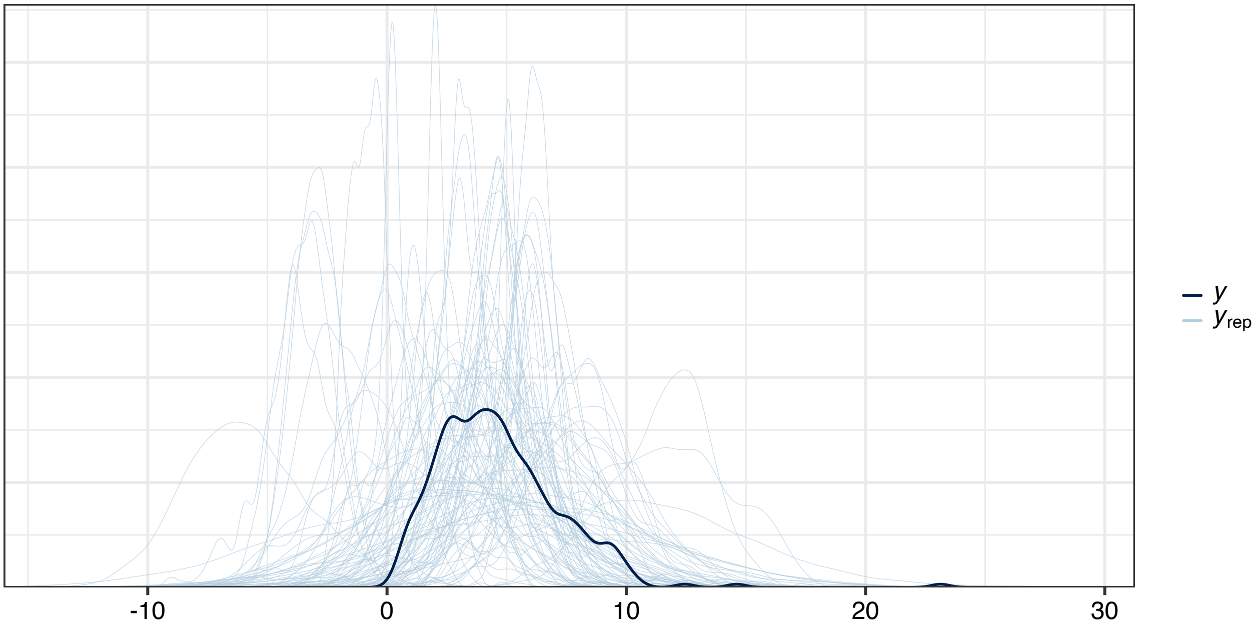


```
pp_check(model.de.tr.extended, nsamples=1e2)
```

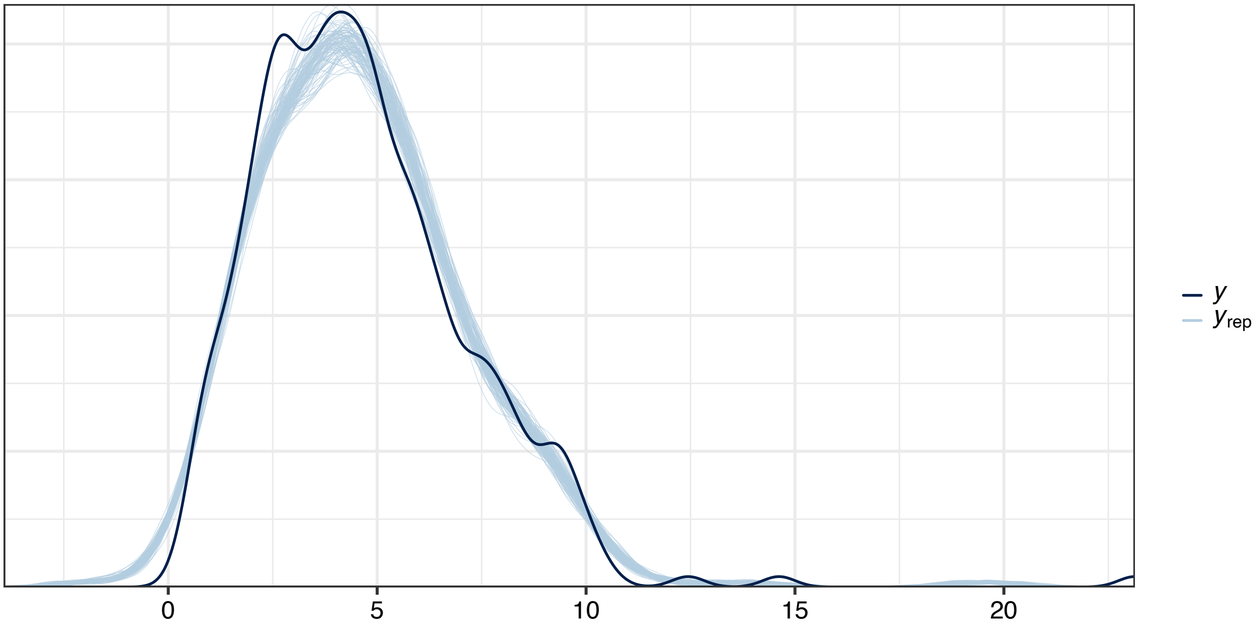


```
loo.m.de.tr.ext <- loo(model.de.tr.extended)
loo.m.de.tr.ext.null <- loo(model.de.tr.extended.null)
```

```r
loo_model_weights(list("hypothesis" = loo.m.de.tr.ext ,
                       "null" = loo.m.de.tr.ext.null))
```

```
## Method: stacking
## ------
##            weight
## hypothesis 0.986
## null       0.014
```

```r
loo_compare(list("hypothesis" = loo.m.de.tr.ext,
                 "null" = loo.m.de.tr.ext.null))
```

```
##            elpd_diff se_diff
## hypothesis     0.0       0.0
## null       -5741.3      88.8
```

Original stimuli, surprisal estimated by GPT-2

```r
contrasts(de.gpt.original.m$first.NP)
```

```
##         object
## subject      0
## object       1
```

```r
contrasts(de.gpt.original.m$verb.type)
```

```
##     dat
## acc   0
## dat   1
```

```r
model.de.gpt.original <-
  brm(surprisal ~ verb.type * first.NP + (1+first.NP+verb.type|sent),
      family = gaussian,
      save_pars = save_pars(all = T),
      sample_prior = T,
      data = de.gpt.original.m,
      file = '../models/de.gpt.original',
      prior = prior.de.lstm.original,
      chains = 4, iter = 8000, warmup=1000, cores = 4,
      control = list(adapt_delta = 0.96, max_treedepth=15)
  )

model.de.gpt.original.prior <-
  brm(surprisal ~ verb.type * first.NP + (1+first.NP+verb.type|sent),
      family = gaussian,
      sample_prior = 'only',
      data = de.gpt.original.m,
      file = '../models/de.gpt.original.prior',
      prior = prior.de.lstm.original
  )
```

```r
model.de.gpt.original.null <-
  brm(surprisal ~ 1+ (1|sent_id),
      family = gaussian,
      save_pars = save_pars(all = T),
      sample_prior = T,
      data = de.gpt.original.m,
      prior = prior.de.lstm.original.null,
      file = '../models/de.gpt.original_null',
      chains = 4, iter = 8000, warmup=1000, cores = 4,
      control = list(adapt_delta = 0.96, max_treedepth=15)
  )

pp_check(model.de.gpt.original.prior, nsamples=1e2)
```

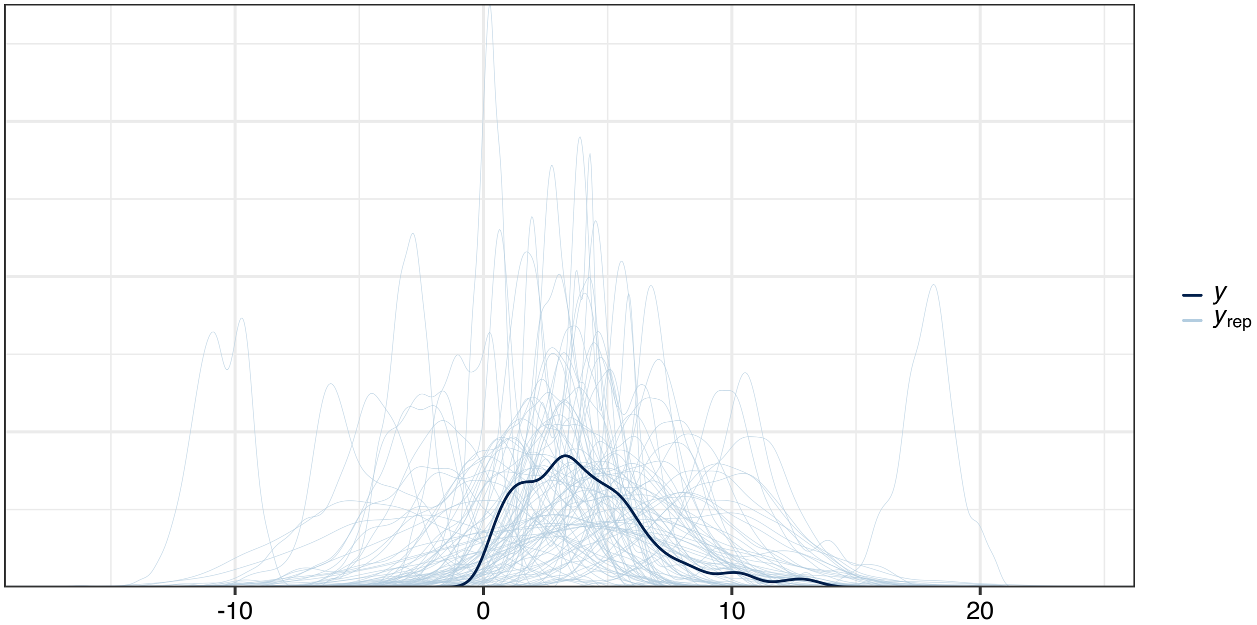


```r
pp_check(model.de.gpt.original, nsamples=1e2)
```

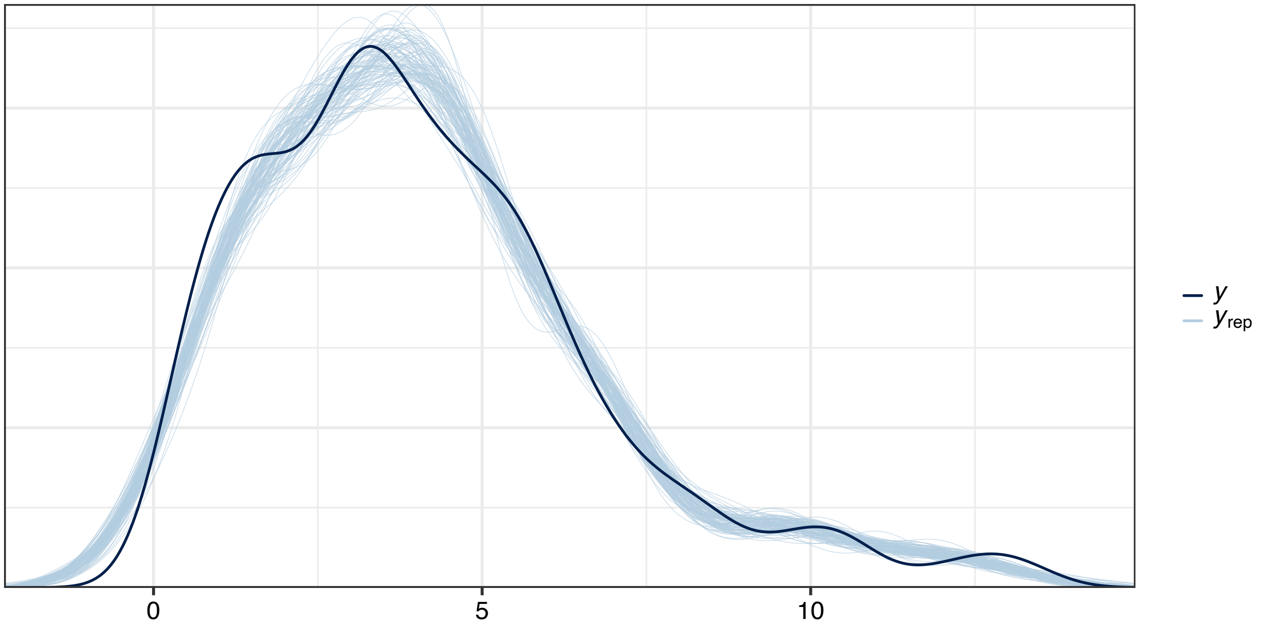


```
loo.m.de.gpt.orig <- loo(model.de.gpt.original)
loo.m.de.gpt.orig.null <- loo(model.de.gpt.original.null)

loo_model_weights(list("hypothesis" = loo.m.de.gpt.orig ,
                       "null" = loo.m.de.gpt.orig.null))
```

```
## Method: stacking
## ------
##            weight
## hypothesis 1.000
## null       0.000
```

```
loo_compare(list("hypothesis" = loo.m.de.gpt.orig,
                 "null" = loo.m.de.gpt.orig.null))
```

```
##            elpd_diff se_diff
## hypothesis     0.0       0.0
## null       -1513.4      34.7
```

Extended stimuli, surprisal estimated by GPT-2

```
contrasts(de.gpt.extended.m$first.NP)
```

```
##         object
## subject      0
## object       1
```

```
contrasts(de.gpt.extended.m$verb.type)
```

```
##     dat
## acc   0
## dat   1
```

```r
model.de.gpt.extended <-
  brm(surprisal ~ verb.type * first.NP + (1+first.NP+verb.type|sent_id),
      family = gaussian,
      save_pars = save_pars(all = T),
      sample_prior = T,
      data = de.gpt.extended.m,
      file = '../models/de.gpt.extended',
      prior = prior.de.lstm.original,
      chains = 4, iter = 8000, warmup=1000, cores = 4,
      control = list(adapt_delta = 0.96, max_treedepth=15)
  )

model.de.gpt.extended.prior <-
  brm(surprisal ~ verb.type * first.NP + (1+first.NP+verb.type|sent_id),
      family = gaussian,
      sample_prior = 'only',
      file = '../models/de.gpt.extended.prior',
      data = de.gpt.extended.m,
      prior = prior.de.lstm.original
  )

model.de.gpt.extended.null <-
  brm(surprisal ~ 1+ (1|sent_id),
      family = gaussian,
      save_pars = save_pars(all = T),
      sample_prior = T,
      data = de.gpt.extended.m,
      prior = prior.de.lstm.original.null,
      file = '../models/de.gpt.extended_null',
      chains = 4, iter = 8000, warmup=1000, cores = 4,
      control = list(adapt_delta = 0.96, max_treedepth=15)
  )

pp_check(model.de.gpt.extended.prior, nsamples=1e2)
```

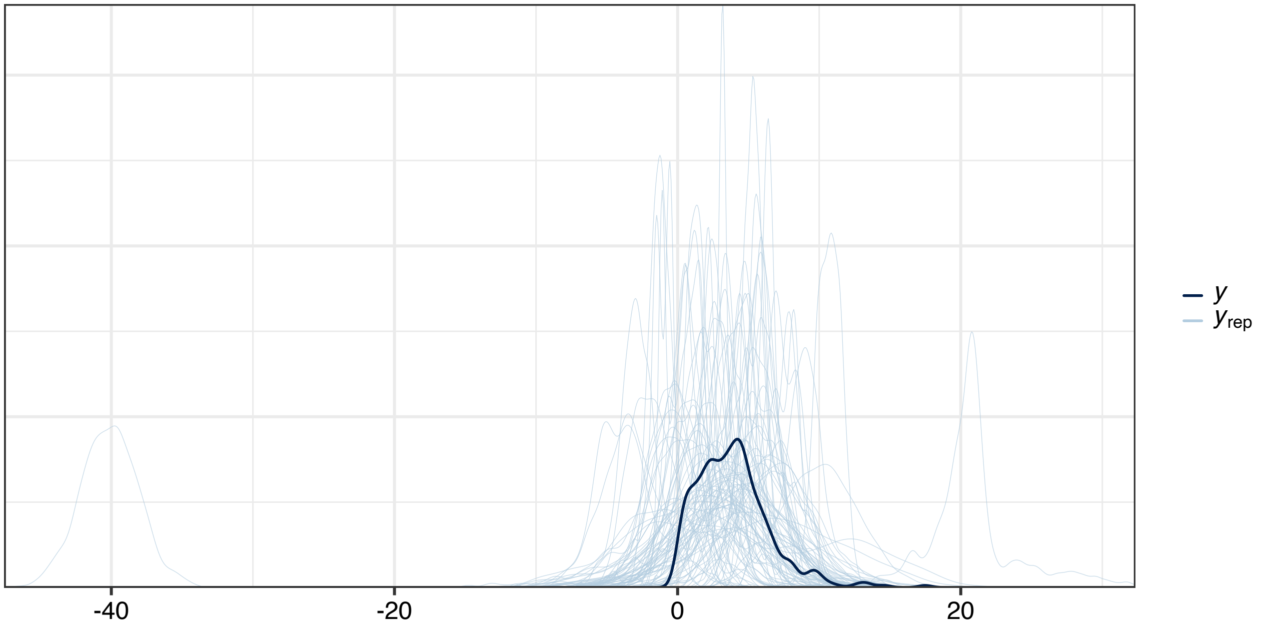


```
pp_check(model.de.gpt.extended, nsamples=1e2)
```

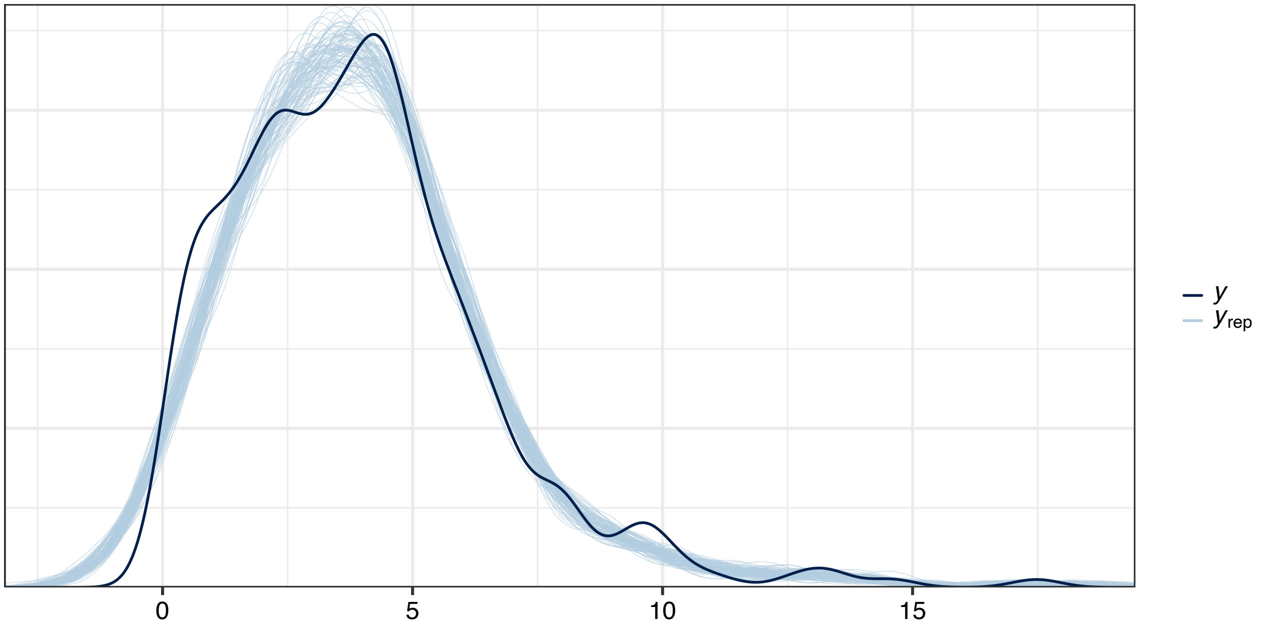


```
loo.m.de.gpt.ext <- loo(model.de.gpt.extended)
loo.m.de.gpt.ext.null <- loo(model.de.gpt.extended.null)

loo_model_weights(list("hypothesis" = loo.m.de.gpt.ext ,
                       "null" = loo.m.de.gpt.ext.null))
```

```
## Method: stacking
## ------
##            weight
## hypothesis 1.000
## null       0.000
```

```r
loo_compare(list("hypothesis" = loo.m.de.gpt.ext,
                 "null" = loo.m.de.gpt.ext.null))
```

```
##            elpd_diff se_diff
## hypothesis     0.0       0.0
## null       -1464.9      38.0
```

S3.2.1 Hindi

Original stimuli, surprisal estimated by LSTM

```
prior.hi <- c(
  prior(normal(0,1), class = b),
  prior(exponential(1), class = sigma),
  prior(exponential(1), class = sd))


prior.hi.null  <- c(
  prior(exponential(1), class = sigma)
)


model.hi.lstm.original <-
  brm(surprisal ~ ambiguity * aspect + (1+ambiguity+aspect|sent_id),
      family = gaussian,
      save_pars = save_pars(all = T),
      sample_prior = T,
      data = hi.lstm.original.m,
      prior = prior.hi.gpt,
      file = '../models/hi.lstm.original',
      chains = 4, iter = 4000, warmup=1000, cores = 4,
      control = list(adapt_delta = 0.96, max_treedepth=15)
  )

model.hi.lstm.original.prior <-
  brm(surprisal ~ ambiguity * aspect + (1+ambiguity+aspect|sent_id),
      family = gaussian,
      sample_prior = 'only',
      file = '../models/hi_lstm_original_prior',
      data = hi.lstm.original.m,
      prior = prior.hi
  )

model.hi.lstm.original.null <-
  brm(surprisal ~ 1 + (1|sent_id),
      family = gaussian,
      save_pars = save_pars(all = T),
      sample_prior = T,
      data = hi.lstm.original.m,
      prior = prior.hi.null,
      file = '../models/hi.lstm.original_null',
      chains = 4, iter = 4000, warmup=1000, cores = 4,
      control = list(adapt_delta = 0.99, max_treedepth=15)
  )


pp_check(model.hi.lstm.original.prior, nsamples=1e2)
```

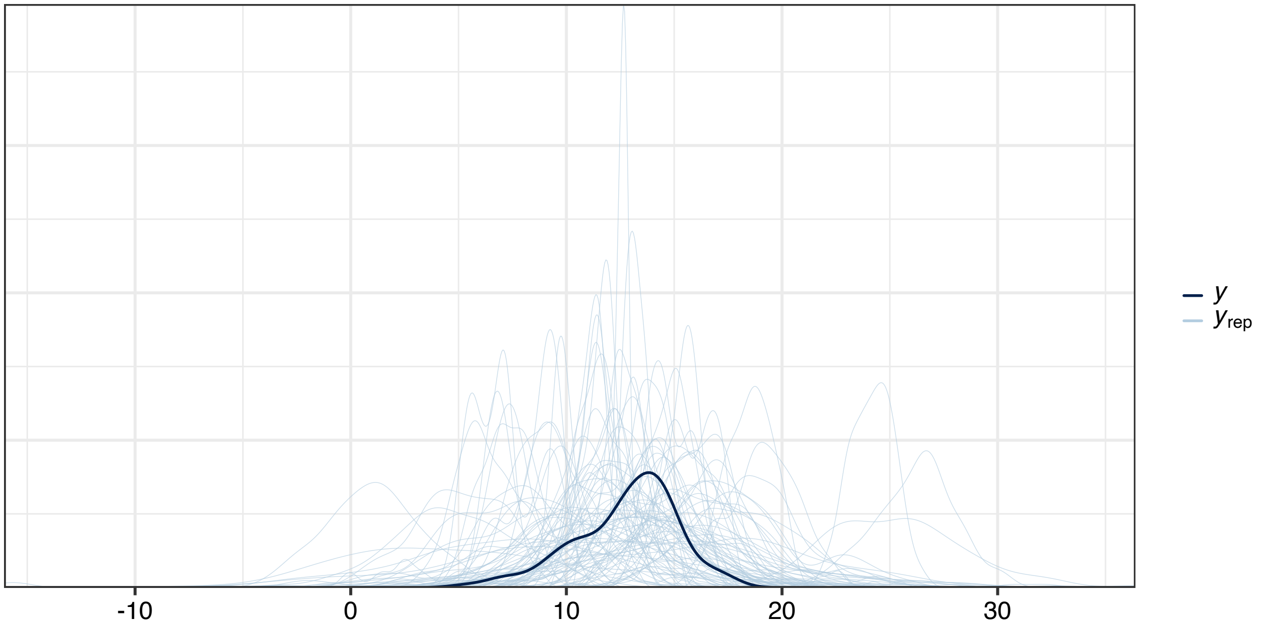


```
pp_check(model.hi.lstm.original, nsamples=1e2)
```

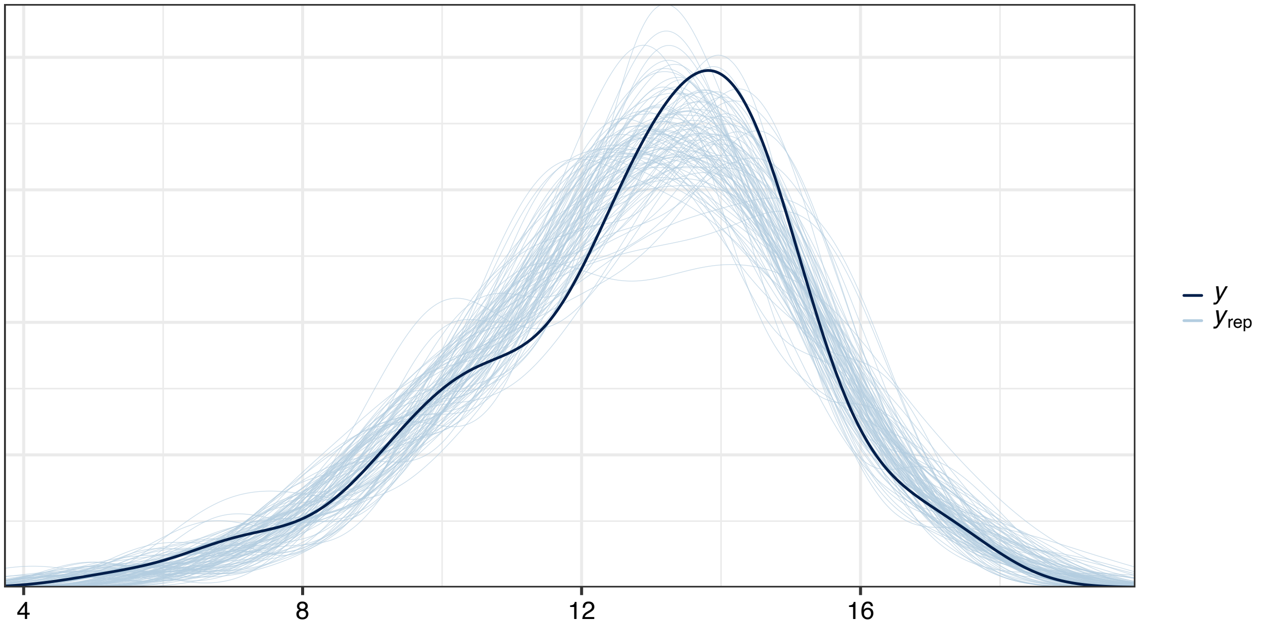


```
loo.m.hi.lstm.orig <- loo(model.hi.lstm.original)
loo.m.hi.lstm.orig.null <- loo(model.hi.lstm.original.null)

loo_model_weights(list("hypothesis" = loo.m.hi.lstm.orig ,
                       "null" = loo.m.hi.lstm.orig.null))
```

```
## Method: stacking
## ------
##            weight
## hypothesis 0.989
## null       0.011
```

```r
loo_compare(list("hypothesis" = loo.m.hi.lstm.orig,
                 "null" = loo.m.hi.lstm.orig.null))
```

```
##            elpd_diff se_diff
## hypothesis   0.0       0.0
## null       -55.9       8.2
```

Extended stimuli, surprisal estimated by LSTM

```r
model.hi.lstm.extended <-
  brm(surprisal ~ ambiguity * aspect + (1|sent_id),
      family = gaussian,
      save_pars = save_pars(all = T),
      sample_prior = T,
      data = hi.lstm.extended.m,
      prior = prior.hi,
      file = '../models/hi_lstm_extended',
      chains = 4, iter = 4000, warmup=1000, cores = 4,
      control = list(adapt_delta = 0.99, max_treedepth=15)
  )

model.hi.lstm.extended.prior <-
  brm(surprisal ~ ambiguity * aspect + (1|sent_id),
      family = gaussian,
      sample_prior = 'only',
      file = '../models/hi_lstm_extended_prior',
      data = hi.lstm.extended.m,
      prior = prior.hi
  )

model.hi.lstm.extended.null <-
  brm(surprisal ~ 1 + (1|sent_id),
      family = gaussian,
      save_pars = save_pars(all = T),
      sample_prior = T,
      data = hi.lstm.extended.m,
      prior = prior.hi.null,
      file = '../models/hi_lstm_extended_null',
      chains = 4, iter = 4000, warmup=1000, cores = 4,
      control = list(adapt_delta = 0.99, max_treedepth=15)
  )


pp_check(model.hi.lstm.extended.prior, nsamples=1e2)
```

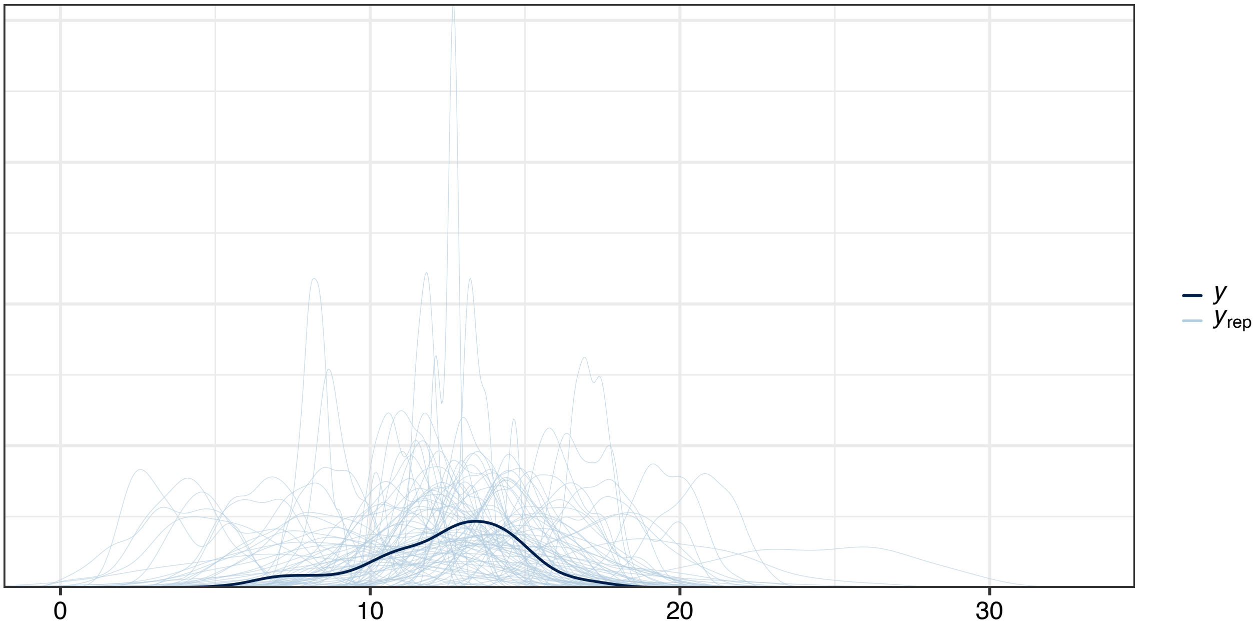


```
pp_check(model.hi.lstm.extended, nsamples=1e2)
```

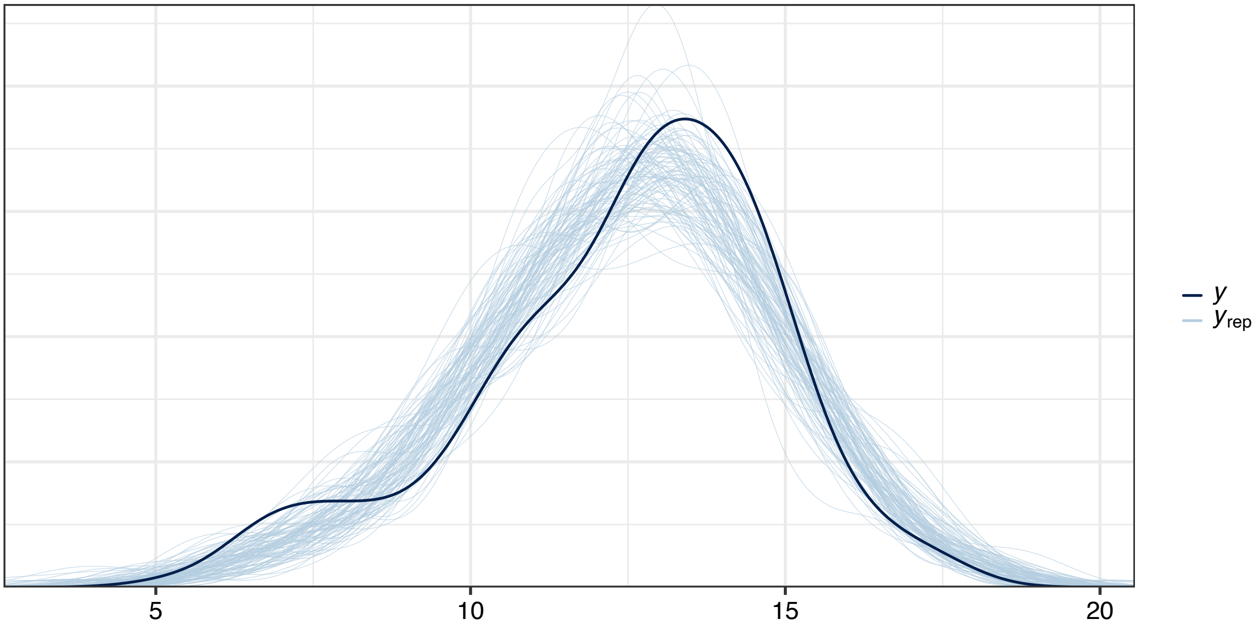


```
loo.m.hi.lstm.ext <- loo(model.hi.lstm.extended, moment_match = T)
loo.m.hi.lstm.ext.null <- loo(model.hi.lstm.extended.null, moment_match = T)

loo_model_weights(list("hypothesis" = loo.m.hi.lstm.ext ,
                       "null" = loo.m.hi.lstm.ext.null))
```

```
## Method: stacking
## ------
##            weight
## hypothesis 0.896
## null       0.104
```

```
loo_compare(list("hypothesis" = loo.m.hi.lstm.ext,
                 "null" = loo.m.hi.lstm.ext.null))
```

```
##            elpd_diff se_diff
## hypothesis   0.0       0.0
## null       -17.9       6.6
```

Original stimuli, surprisal estimated by transformer

```
model.hi.bert.original <-
  brm(surprisal ~ ambiguity * aspect + (1+ambiguity+aspect|sent_id),
      family = gaussian,
      save_all_pars = T,
      sample_prior = T,
      data = hi.tr.original.m,
      prior = prior.hi,
      file = '../models/hi_tr_original',
      chains = 4, iter = 4000, warmup=1000, cores = 4,
      control = list(adapt_delta = 0.96, max_treedepth=15)
  )

model.hi.bert.original.prior <-
  brm(surprisal ~ ambiguity * aspect +  (1+ambiguity+aspect|sent_id),
      family = gaussian,
      sample_prior = 'only',
      file = '../models/hi_bert_original_prior',
      data = hi.tr.original.m,
      prior = prior.hi
  )


model.hi.bert.original.null <-
  brm(surprisal ~ 1 + (1|sent_id),
      family = gaussian,
      save_all_pars = T,
      sample_prior = T,
      data = hi.tr.original.m,
      prior = prior.hi.null,
      file = '../models/hi_tr_original_null',
      chains = 4, iter = 4000, warmup=1000, cores = 4,
      control = list(adapt_delta = 0.96, max_treedepth=15)
  )


pp_check(model.hi.bert.original.prior, nsamples=1e2)
```

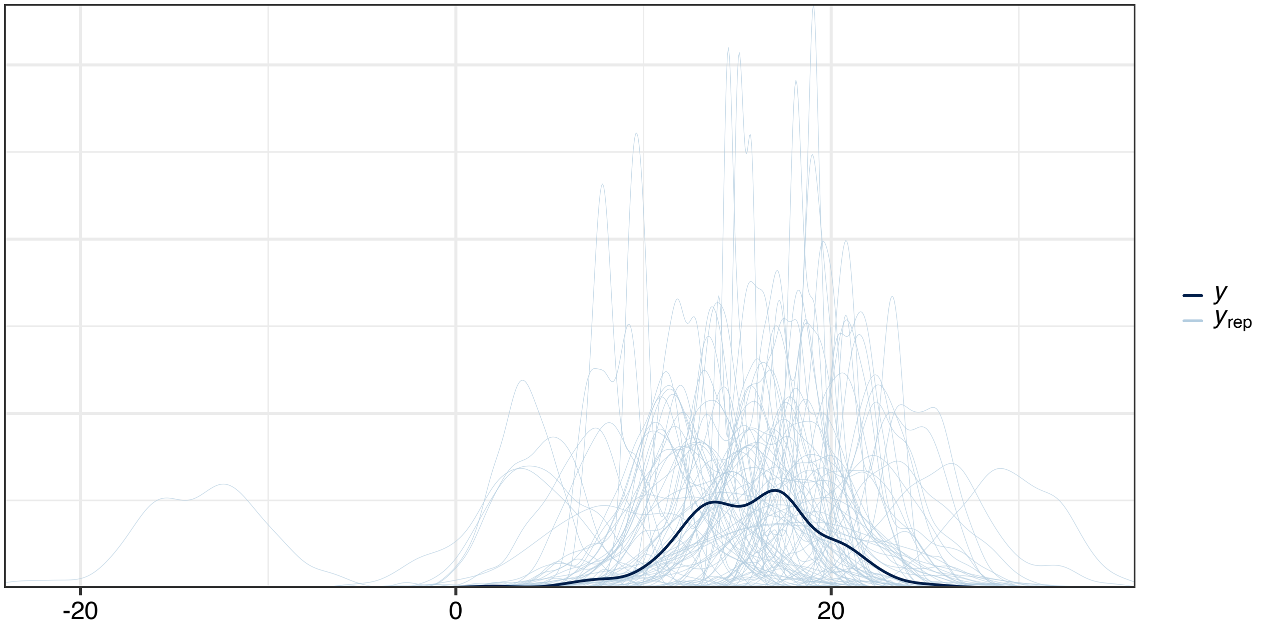


```
pp_check(model.hi.bert.original, nsamples=1e2)
```

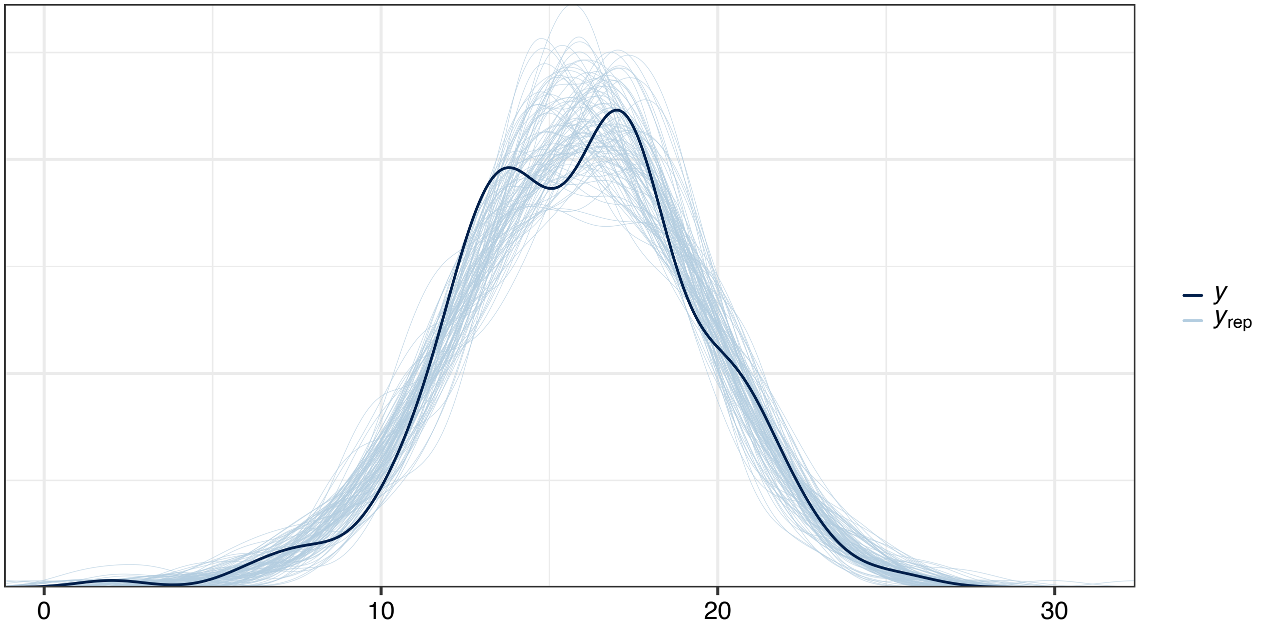


```
loo.m.hi.transformer.orig <- loo(model.hi.bert.original)
loo.m.hi.transformer.orig.null <- loo(model.hi.bert.original.null)

loo_model_weights(list("hypothesis" = loo.m.hi.transformer.orig ,
                       "null" = loo.m.hi.transformer.orig.null))
```

```
## Method: stacking
## ------
##            weight
## hypothesis 0.970
## null       0.030
```

```
loo_compare(list("hypothesis" = loo.m.hi.transformer.orig,
                 "null" = loo.m.hi.transformer.orig.null))
```

```
##            elpd_diff se_diff
## hypothesis   0.0       0.0
## null       -35.2       7.7
```

Extended stimuli, surprisal estimated by transformer

```
model.hi.bert.extended <-
  brm(surprisal ~ ambiguity * aspect +  (1+ambiguity+aspect|sent_id),
      family = gaussian,
      save_pars = save_pars(all = T),
      sample_prior = T,
      data = hi.tr.extended.m,
      prior = prior.hi,
      file = '../models/hi_transformer_extended',
      chains = 4, iter = 4000, warmup=1000, cores = 4,
      control = list(adapt_delta = 0.99, max_treedepth=15)
  )

model.hi.bert.extended.prior <-
  brm(surprisal ~ ambiguity * aspect +  (1+ambiguity+aspect|sent_id),
      family = gaussian,
      sample_prior = 'only',
      file = '../models/hi_bert_extended_prior',
      data = hi.tr.extended.m,
      prior = prior.hi
  )

model.hi.bert.extended.null <-
  brm(surprisal ~ 1 + (1|sent_id),
      family = gaussian,
      save_pars = save_pars(all = T),
      sample_prior = T,
      data = hi.tr.extended.m,
      prior = prior.hi.null,
      file = '../models/hi_transformer_extended_null',
      chains = 4, iter = 4000, warmup=1000, cores = 4,
      control = list(adapt_delta = 0.99, max_treedepth=15)
  )


pp_check(model.hi.bert.extended.prior, nsamples=1e2)
```

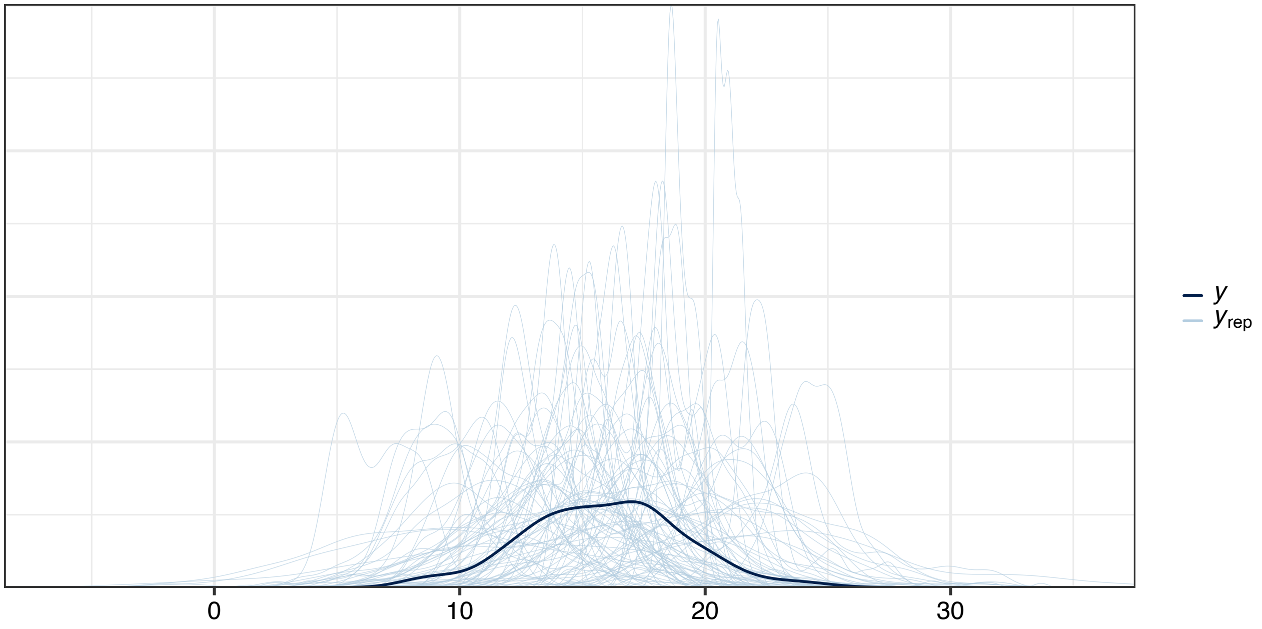


```r
pp_check(model.hi.bert.extended, nsamples=1e2)
```

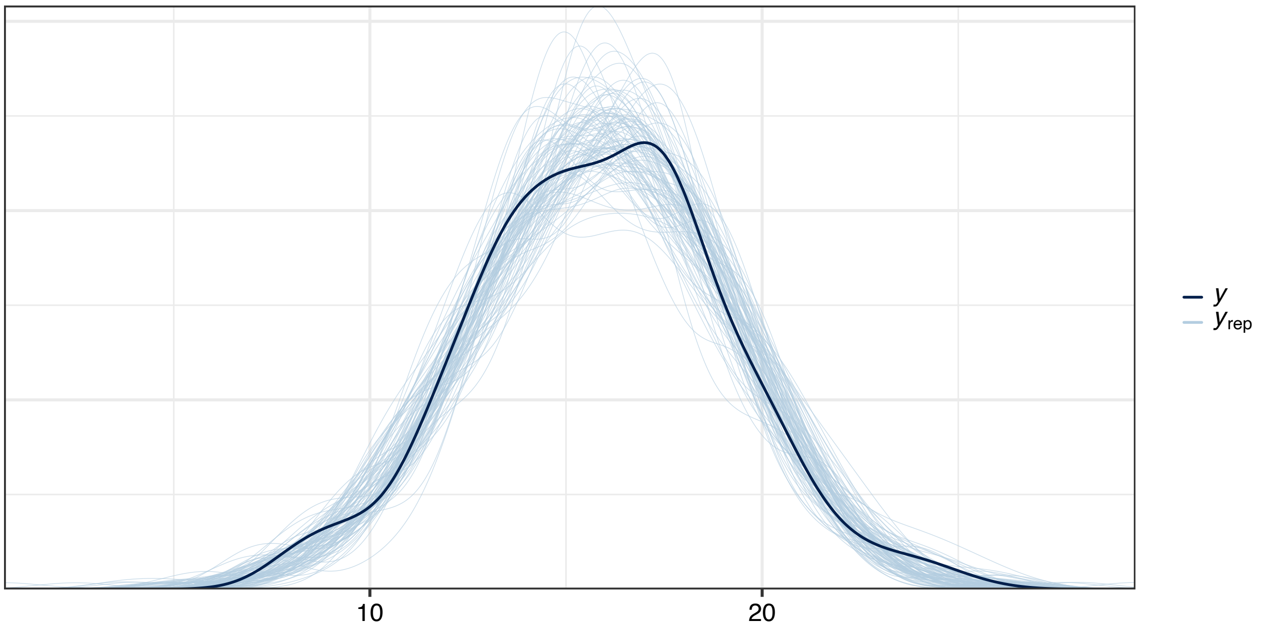


```r
loo.m.hi.transformer.ext <- loo(model.hi.bert.extended)
loo.m.hi.transformer.ext.null <- loo(model.hi.bert.extended.null)

loo_model_weights(list("hypothesis" = loo.m.hi.transformer.ext ,
                       "null" = loo.m.hi.transformer.ext.null))
```

```
## Method: stacking
## ------
##            weight
## hypothesis 1.000
## null       0.000
```

```r
loo_compare(list("hypothesis" = loo.m.hi.transformer.ext,
                 "null" = loo.m.hi.transformer.ext.null))
```

```
##             elpd_diff se_diff
## hypothesis   0.0       0.0
## null       -31.6       7.0
```

Original stimuli, surprisal estimated by GPT-2

```r
contrasts(hi.gpt.original.m$ambiguity)
```

```
##       UNAMB
## AMB       0
## UNAMB     1
```

```r
contrasts(hi.gpt.original.m$aspect)
```

```
##      PFV
## IPFV   0
## PFV    1
```

```r
model.hi.gpt.original <-
  brm(surprisal ~ ambiguity * aspect + (1+ambiguity+aspect|sent_id),
      family = gaussian,
      save_pars = save_pars(all = T),
      sample_prior = T,
      data = hi.gpt.original.m,
      file = '../models/hi_gpt_original_prior',
      prior = prior.hi,
      chains = 4, iter = 4000, warmup=1000, cores = 4,
      control = list(adapt_delta = 0.96, max_treedepth=15)
  )

model.hi.gpt.original.prior <-
  brm(surprisal ~ ambiguity * aspect + (1+ambiguity+aspect|sent_id),
      family = gaussian,
      sample_prior = 'only',
      file = '../models/hi_gpt_original_prior',
      data = hi.gpt.original.m,
      prior = prior.hi
  )

model.hi.gpt.original.null <-
  brm(surprisal ~ 1 + (1|sent_id),
      family = gaussian,
      save_pars = save_pars(all = T),
      sample_prior = T,
      data = hi.gpt.original.m,
      prior = prior.hi.null,
      file = '../models/hi_gpt_original_null',
      chains = 4, iter = 4000, warmup=1000, cores = 4,
```

```
      control = list(adapt_delta = 0.96, max_treedepth=15)
  )

pp_check(model.hi.gpt.original.prior, nsamples=1e2)
```

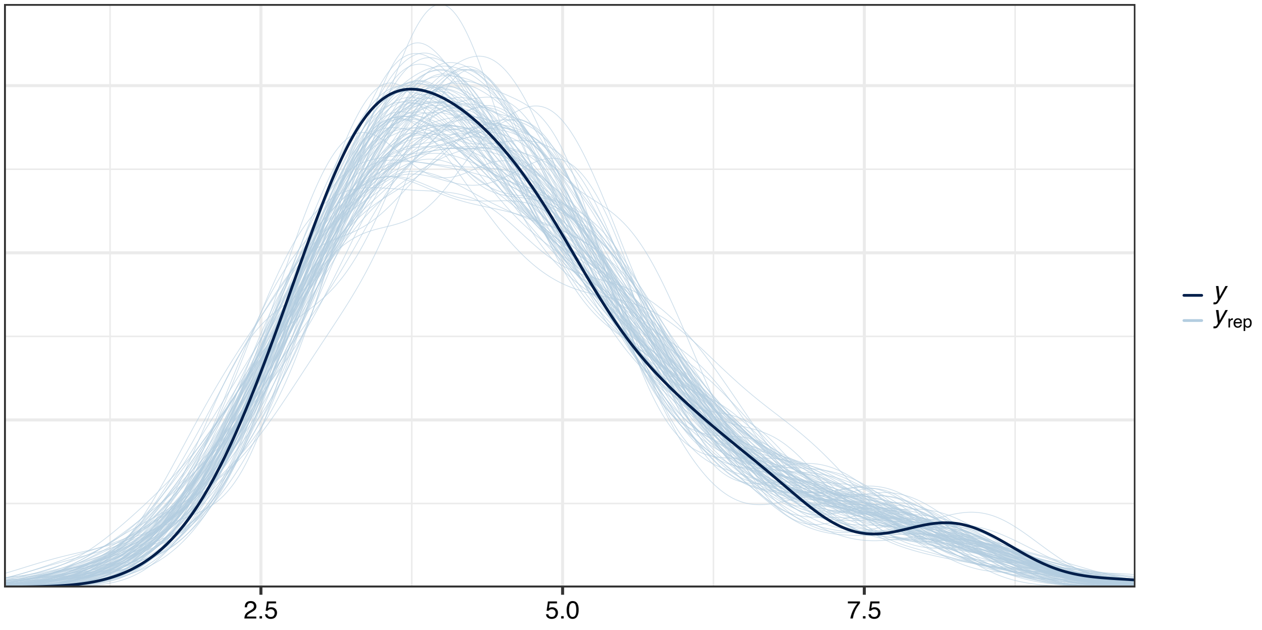


```
pp_check(model.hi.gpt.original, nsamples=1e2)
```

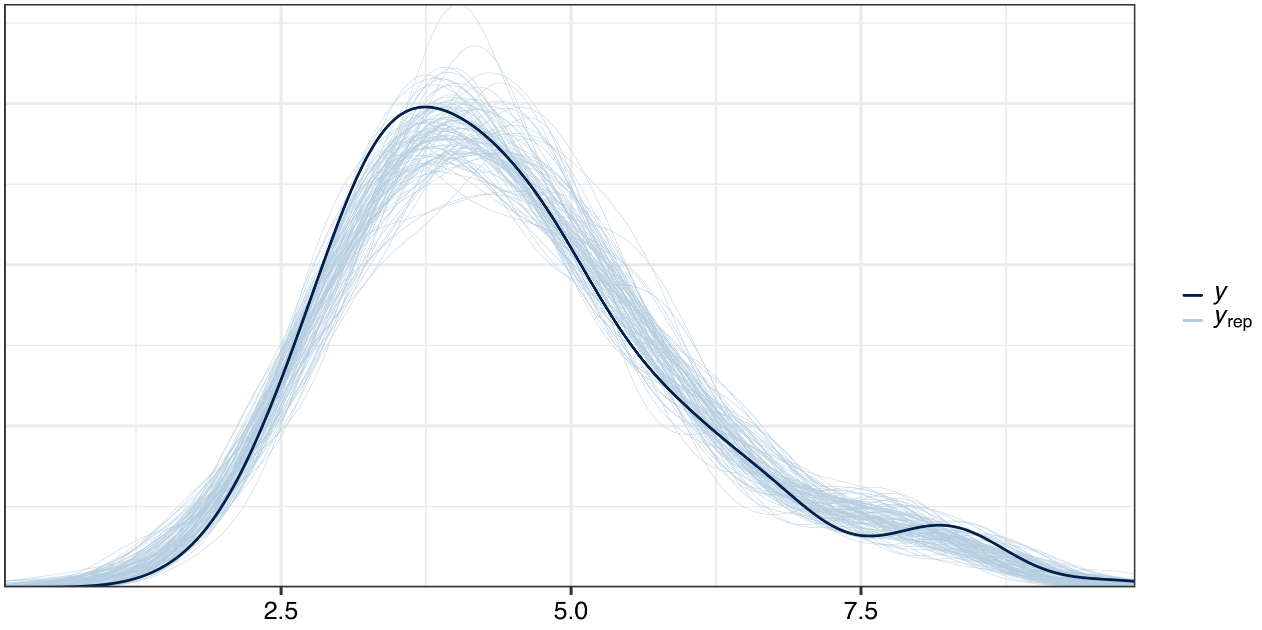


```
loo.m.hi.gpt.orig <- loo(model.hi.gpt.original)
loo.m.hi.gpt.orig.null <- loo(model.hi.gpt.original.null)

loo_model_weights(list("hypothesis" = loo.m.hi.gpt.orig ,
                       "null" = loo.m.hi.gpt.orig.null))
```

```
## Method: stacking
## ------
##            weight
## hypothesis 1.000
## null       0.000
```

```
loo_compare(list("hypothesis" = loo.m.hi.gpt.orig,
                 "null" = loo.m.hi.gpt.orig.null))
```

```
##            elpd_diff se_diff
## hypothesis    0.0       0.0
## null       -111.0      11.9
```

Extended stimuli, surprisal estimated by GPT-2

```
contrasts(hi.gpt.extended.m$ambiguity)
```

```
##       UNAMB
## AMB       0
## UNAMB     1
```

```
contrasts(hi.gpt.extended.m$aspect)
```

```
##      PFV
## IPFV   0
## PFV    1
```

```
model.hi.gpt.extended <-
  brm(surprisal ~ ambiguity * aspect + (1+ambiguity+aspect|sent_id),
      family = gaussian,
      save_pars = save_pars(all = T),
      sample_prior = T,
      data = hi.gpt.extended.m,
      prior = prior.hi,
      file = '../models/hi_gpt_extended',
      chains = 4, iter = 8000, warmup=1000, cores = 4,
      control = list(adapt_delta = 0.996, max_treedepth=15)
  )

model.hi.gpt.extended.prior <-
  brm(surprisal ~ ambiguity * aspect + (1+ambiguity+aspect|sent_id),
      family = gaussian,
      sample_prior = 'only',
      data = hi.gpt.extended.m,
      file = '../models/hi_gpt_extended_prior',
      prior = prior.hi
  )

model.hi.gpt.extended.null <-
  brm(surprisal ~ 1 + (1|sent_id),
      family = gaussian,
```

```r
      save_pars = save_pars(all = T),
      sample_prior = T,
      data = hi.gpt.extended.m,
      prior = prior.hi.null,
      file = '../models/hi_gpt_extended_null',
      chains = 4, iter = 8000, warmup=1000, cores = 4,
      control = list(adapt_delta = 0.99, max_treedepth=15)
  )

pp_check(model.hi.gpt.extended.prior, nsamples=1e2)
```

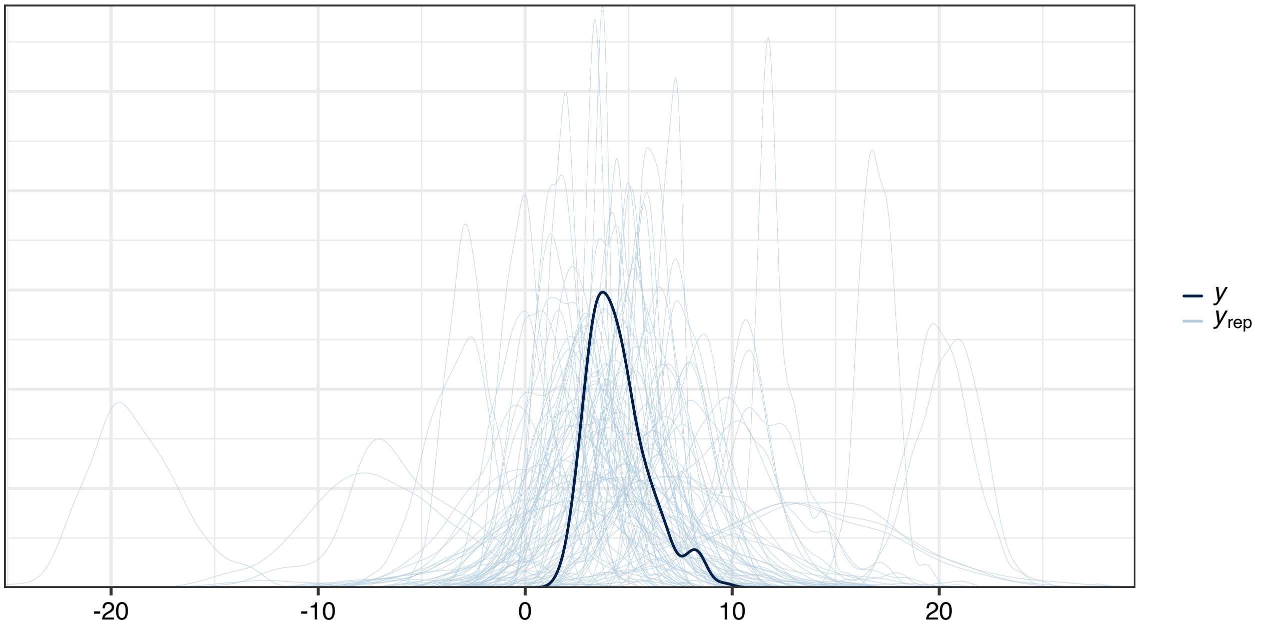


```r
pp_check(model.hi.gpt.extended, nsamples=1e2)
```

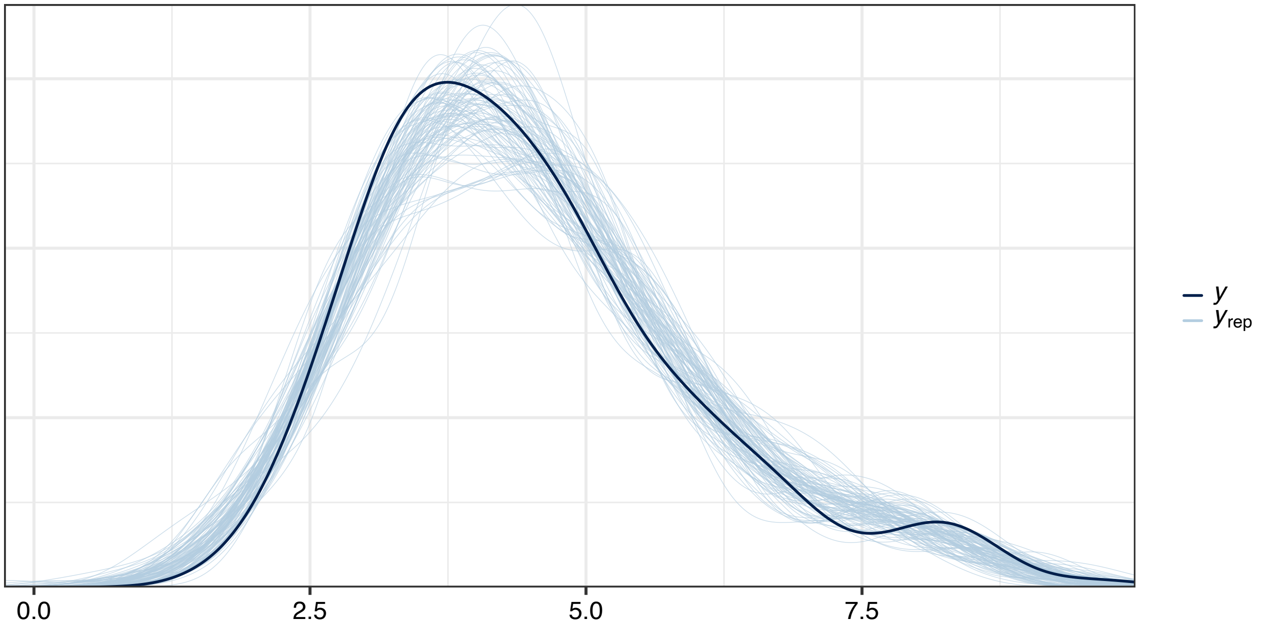

```r
loo.m.hi.gpt.ext <- loo(model.hi.gpt.extended)
loo.m.hi.gpt.ext.null <- loo(model.hi.gpt.extended.null)

loo_model_weights(list("hypothesis" = loo.m.hi.gpt.ext ,
                       "null" = loo.m.hi.gpt.ext.null))
```

```
## Method: stacking
## ------
##            weight
## hypothesis 1.000
## null       0.000
```

```r
loo_compare(list("hypothesis" = loo.m.hi.gpt.ext,
                 "null" = loo.m.hi.gpt.ext.null))
```

```
##            elpd_diff se_diff
## hypothesis    0.0       0.0
## null       -109.9      11.9
```

Original stimuli, surprisal estimated by LSTM

```
contrasts(eu.lstm.original.m$Semantic.Role)
```

```
##     Erg
## Abs   0
## Erg   1
```

```
contrasts(eu.lstm.original.m$Ambiguity)
```

```
##       Unamb
## Amb       0
## Unamb     1
```

```
prior.eu <- c(
  prior(normal(0,1), class = b),
  prior(gamma(2, 1), class = nu),
  prior(cauchy(0,4), class = sigma),
  prior(cauchy(0,4), class = sd)
)

prior.eu.null <- c(
  prior(gamma(2, 1), class = nu),
  prior(cauchy(0,4), class = sigma)
)


model.basque.lstm.original <-
  brm(surprisal ~ 1 + Semantic.Role*Ambiguity +
        (1 + Semantic.Role + Ambiguity|sent_id),
      family = student(),
      save_pars = save_pars(all = T),
      sample_prior = T,
      prior = prior.eu,
      data = eu.lstm.original.m,
      file = '../models/eu.lstm.original',
      chains = 4, iter = 4000, warmup = 2000, cores = 4,
      control = list(adapt_delta = 0.999, max_treedepth=15)
  )


model.basque.lstm.original.prior <-
  brm(surprisal ~ 1 + Semantic.Role*Ambiguity +
        (1 + Semantic.Role + Ambiguity|sent_id),
      family = student(),
      sample_prior = 'only',
      file = '../models/eu.lstm.original.prior',
      prior = prior.eu,
      data = eu.lstm.original.m
  )

model.null.basque.lstm.original <-
```

```r
  brm(surprisal ~ 1 + (1|sent_id),
      family = student(),
      sample_prior = T,
      prior = prior.eu.null,
      data = eu.lstm.original.m,
      file = '../models/eu.lstm.original_null',
      chains = 4, iter = 4000, warmup = 2000, cores = 4,
      control = list(adapt_delta = 0.96, max_treedepth=15)
  )


pp_check(model.basque.lstm.original.prior, nsamples=1e2)
```

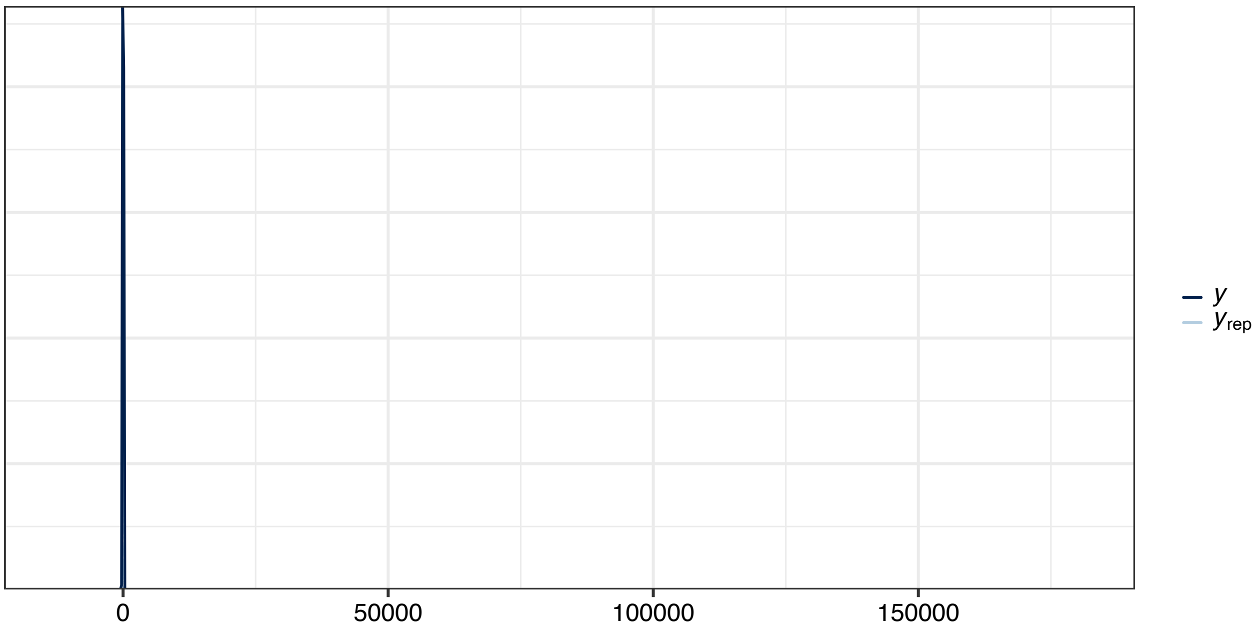


```r
pp_check(model.basque.lstm.original, nsamples=1e2)
```

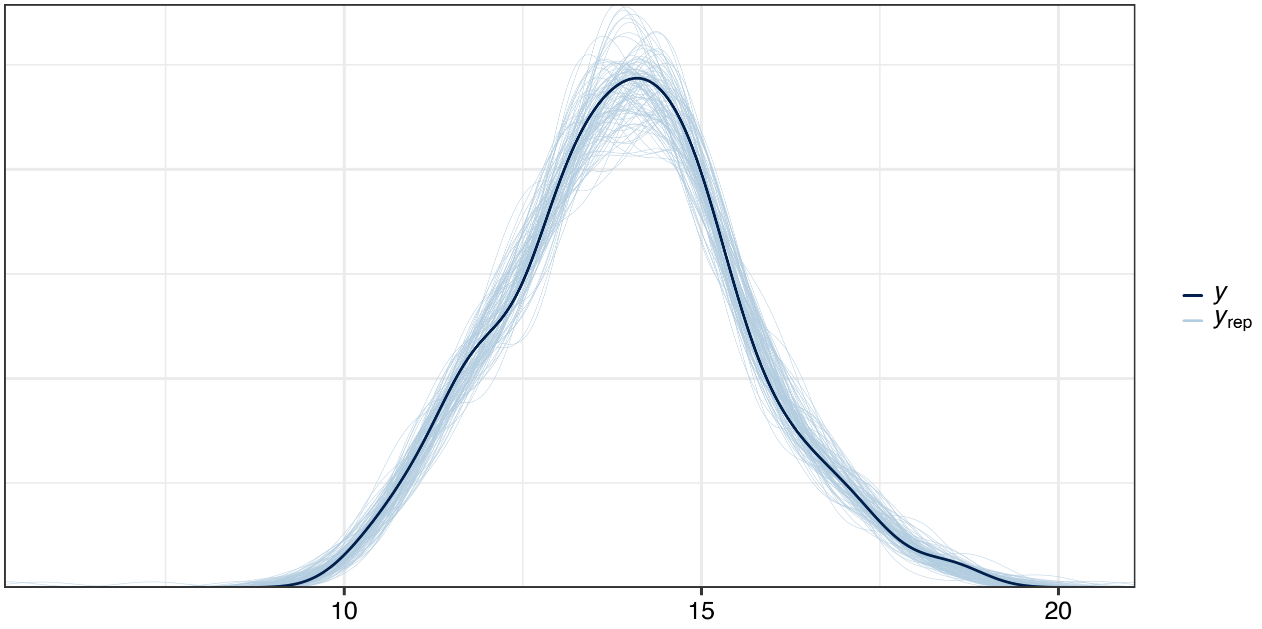


```
loo.m.eu.lstm.orig <- loo(model.basque.lstm.original)
loo.m.eu.lstm.orig.null <- loo(model.null.basque.lstm.original)

loo_model_weights(list("hypothesis" = loo.m.eu.lstm.orig ,
                       "null" = loo.m.eu.lstm.orig.null))
```

```
## Method: stacking
## ------
##            weight
## hypothesis 1.000
## null       0.000
```

```
loo_compare(list("hypothesis" = loo.m.eu.lstm.orig,
                 "null" = loo.m.eu.lstm.orig.null))
```

```
##            elpd_diff se_diff
## hypothesis    0.0       0.0
## null       -233.2      19.6
```

Extended stimuli, surprisal estimated by LSTM

```
contrasts(eu.lstm.extended.m$Ambiguity)
```

```
##       Unamb
## Amb       0
## Unamb     1
```

```
contrasts(eu.lstm.extended.m$Semantic.Role)
```

```
##     Erg
## Abs   0
## Erg   1
```

```r
model.basque.lstm.extended <-
  brm(surprisal ~ 1 + Semantic.Role*Ambiguity +
        (1 + Semantic.Role + Ambiguity|sent_id),
      family = student(),
      sample_prior = T,
      prior = prior.eu,
      data = eu.lstm.extended.m,
      file = '../models/eu.lstm.extended',
      chains = 4, iter = 4000, warmup = 2000, cores = 4,
      control = list(adapt_delta = 0.996, max_treedepth=15)
  )

model.basque.lstm.extended.prior <-
  brm(surprisal ~ 1 + Semantic.Role*Ambiguity +
        (1 + Semantic.Role + Ambiguity|sent_id),
      family = student(),
      sample_prior = 'only',
      file = '../models/eu.lstm.extended.prior',
      prior = prior.eu,
      data = eu.lstm.extended.m
  )


model.null.basque.lstm.extended <-
  brm(surprisal ~ 1 + (1|sent_id),
      family =student(),
      sample_prior = T,
      prior = prior.eu.null,
      data = eu.lstm.extended.m,
      file = '../models/eu.lstm.extended_null',
      chains = 4, iter = 4000, warmup = 2000, cores = 4,
      control = list(adapt_delta = 0.96, max_treedepth=15)
  )


pp_check(model.basque.lstm.extended.prior, nsamples=1e2)
```

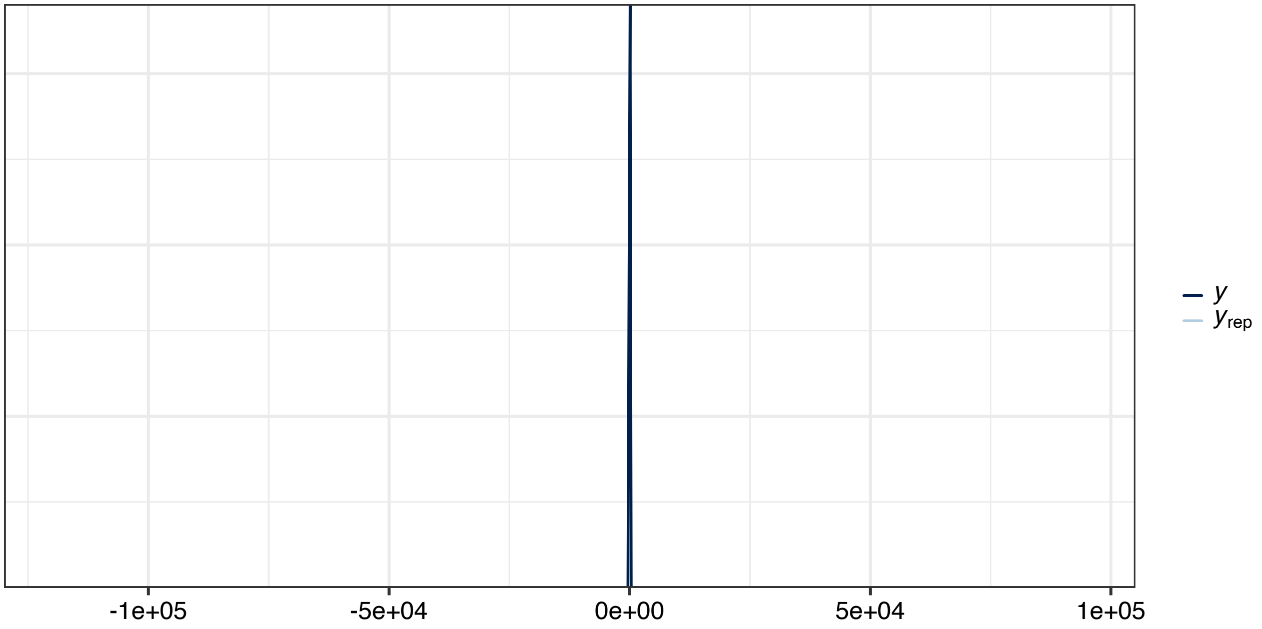


```r
pp_check(model.basque.lstm.extended, nsamples=1e2)
```

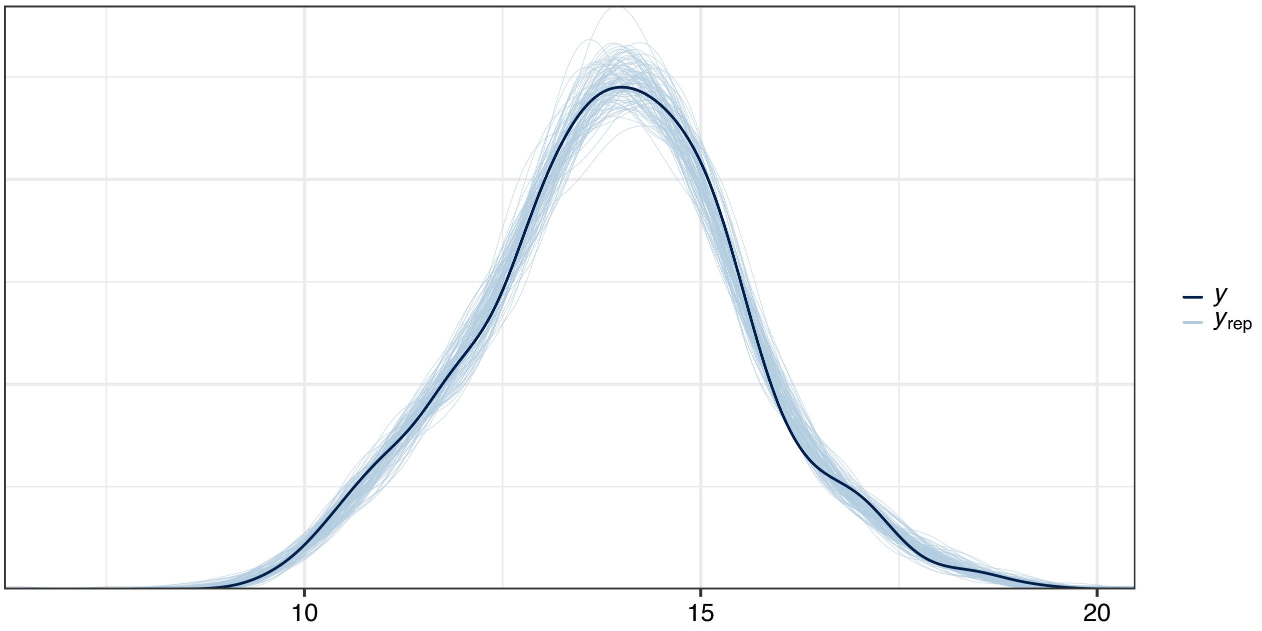


```r
loo.m.eu.lstm.ext <- loo(model.basque.lstm.extended)
loo.m.eu.lstm.ext.null <- loo(model.null.basque.lstm.extended)

loo_model_weights(list("hypothesis" = loo.m.eu.lstm.ext ,
                       "null" = loo.m.eu.lstm.ext.null))
```

```
## Method: stacking
## ------
##            weight
## hypothesis 1.000
## null       0.000
```

Original stimuli, surprisal estimated by RoBERTa

```
contrasts(eu.tr.original.m$Ambiguity)
```

```
##       Unamb
## Amb       0
## Unamb     1
```

```
contrasts(eu.tr.original.m$Semantic.Role)
```

```
##     Erg
## Abs   0
## Erg   1
```

```
model.basque.tr.original <-
  brm(surprisal ~ 1 + Semantic.Role*Ambiguity +
        (1 + Semantic.Role + Ambiguity|sent_id),
      family = student(),
      save_pars = save_pars(all = T),
      sample_prior = T,
      prior = prior.eu,
      data = eu.tr.original.m,
      file = '../models/eu.tr.original',
      chains = 4, iter = 8000, warmup = 2000, cores = 4,
      control = list(adapt_delta = 0.999, max_treedepth=15)
  )

model.basque.tr.original.prior <-
  brm(surprisal ~ 1 + Semantic.Role*Ambiguity +
        (1 + Semantic.Role + Ambiguity|sent_id),
      family = student(),
      sample_prior = 'only',
      file = '../models/eu.tr.original.prior',
      prior = prior.eu,
      data = eu.tr.original.m)


model.null.basque.tr.original <-
  brm(surprisal ~ 1 + (1|sent_id),
      family = student(),
      save_pars = save_pars(all = T),
      sample_prior = T,
      prior = prior.eu.null,
      data = eu.tr.original.m,
      file = '../models/eu.tr.original_null',
      chains = 4, iter = 8000, warmup = 2000, cores = 4,
      control = list(adapt_delta = 0.999, max_treedepth=15)
  )


pp_check(model.basque.tr.original.prior, nsamples=1e2)
```

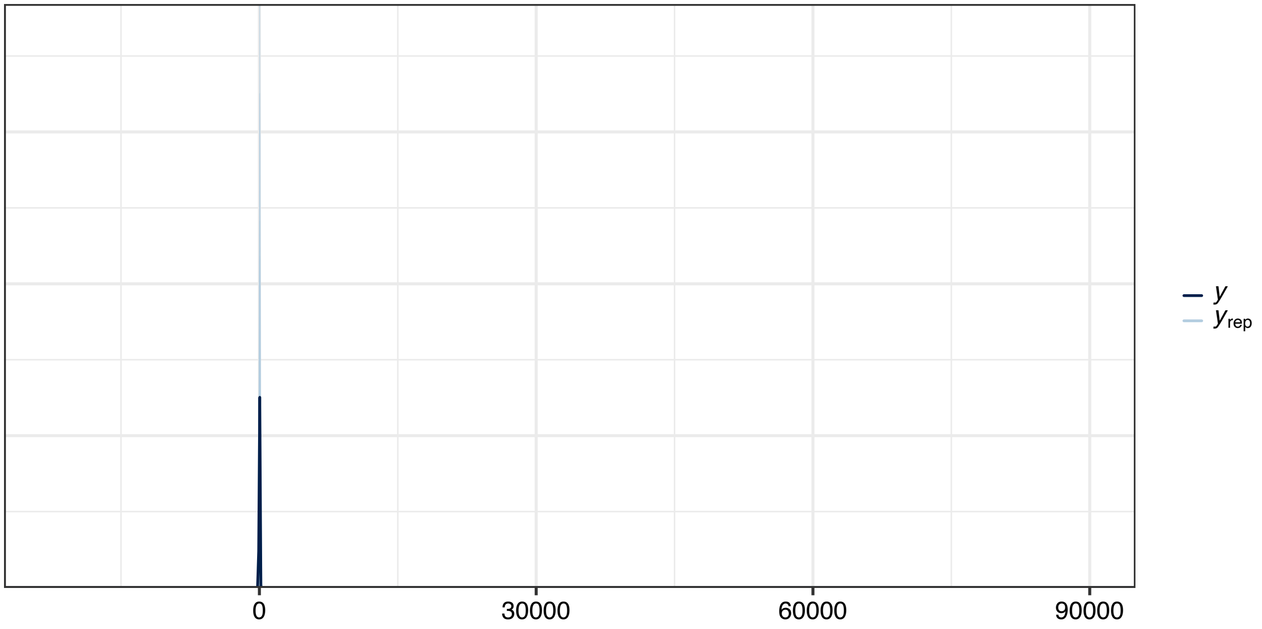


```r
pp_check(model.basque.tr.original, nsamples=1e2)
```

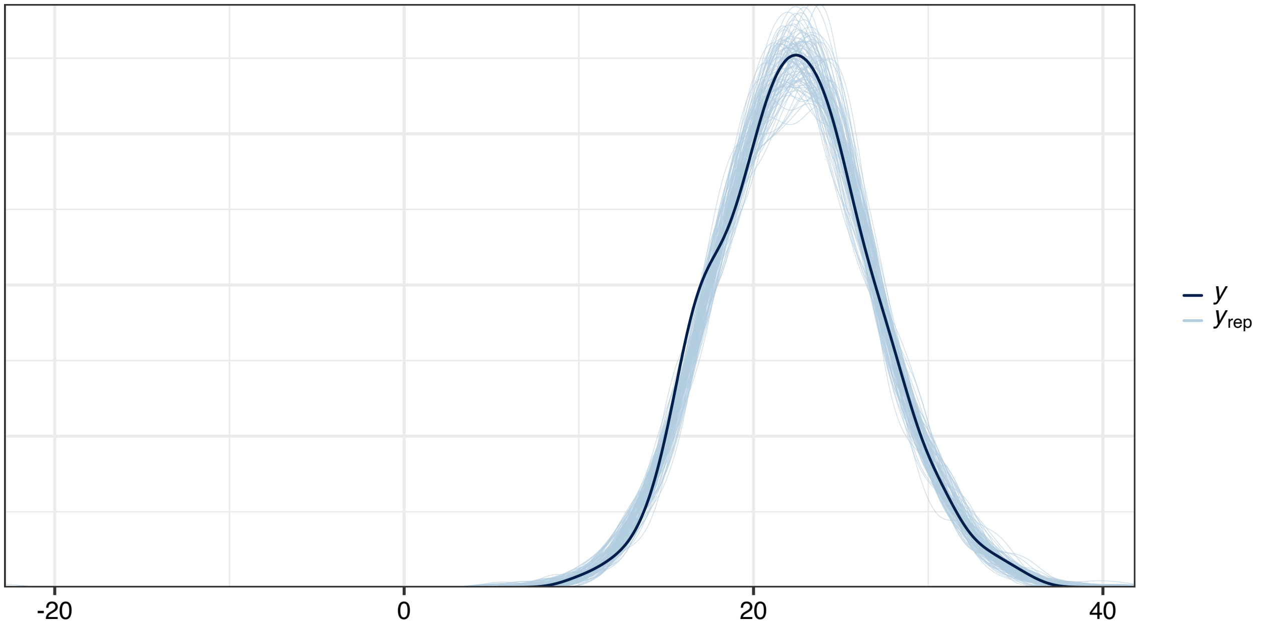


```r
loo.m.eu.tr.orig <- loo(model.basque.tr.original)
loo.m.eu.tr.orig.null <- loo(model.null.basque.tr.original)

loo_model_weights(list("hypothesis" = loo.m.eu.tr.orig ,
                       "null" = loo.m.eu.tr.orig.null))
```

```
## Method: stacking
## ------
##            weight
## hypothesis 1.000
## null       0.000
```

```
loo_compare(list("hypothesis" = loo.m.eu.tr.orig,
                 "null" = loo.m.eu.tr.orig.null))
```

```
##            elpd_diff se_diff
## hypothesis    0.0       0.0
## null       -261.3      20.1
```

Extended stimuli, surprisal estimated by RoBERTa

```
contrasts(eu.tr.extended.m$Ambiguity)
```

```
##       Unamb
## Amb       0
## Unamb     1
```

```
contrasts(eu.tr.extended.m$Semantic.Role)
```

```
##     Erg
## Abs   0
## Erg   1
```

```
model.basque.tr.extended <-
  brm(surprisal ~ 1 + Semantic.Role*Ambiguity +
        (1 + Semantic.Role + Ambiguity|sent_id),
      family = student(),
      save_pars = save_pars(all = T),
      sample_prior = T,
      prior = prior.eu,
      data = eu.tr.extended.m,
      file = '../models/eu.tr.extended',
      chains = 4, iter = 4000, warmup = 2000, cores = 4,
      control = list(adapt_delta = 0.96, max_treedepth=15)
  )

model.basque.tr.extended.prior <-
  brm(surprisal ~ 1 + Semantic.Role *Ambiguity +
        (1 + Semantic.Role + Ambiguity|sent_id),
      family = student(),
      sample_prior = 'only',
      file = '../models/eu.tr.extended.prior',
      prior = prior.eu,
      data = eu.tr.extended.m
  )

model.null.basque.tr.extended <-
  brm(surprisal ~ 1 + (1|sent_id),
      family = student(),
      save_pars = save_pars(all = T),
      sample_prior = T,
      prior = prior.eu.null,
      data = eu.tr.extended.m,
```

```r
      file = '../models/eu.tr.extended_null',
      chains = 4, iter = 4000, warmup = 2000, cores = 4,
      control = list(adapt_delta = 0.96, max_treedepth=15)
  )

pp_check(model.basque.tr.extended.prior, nsamples=1e2)
```

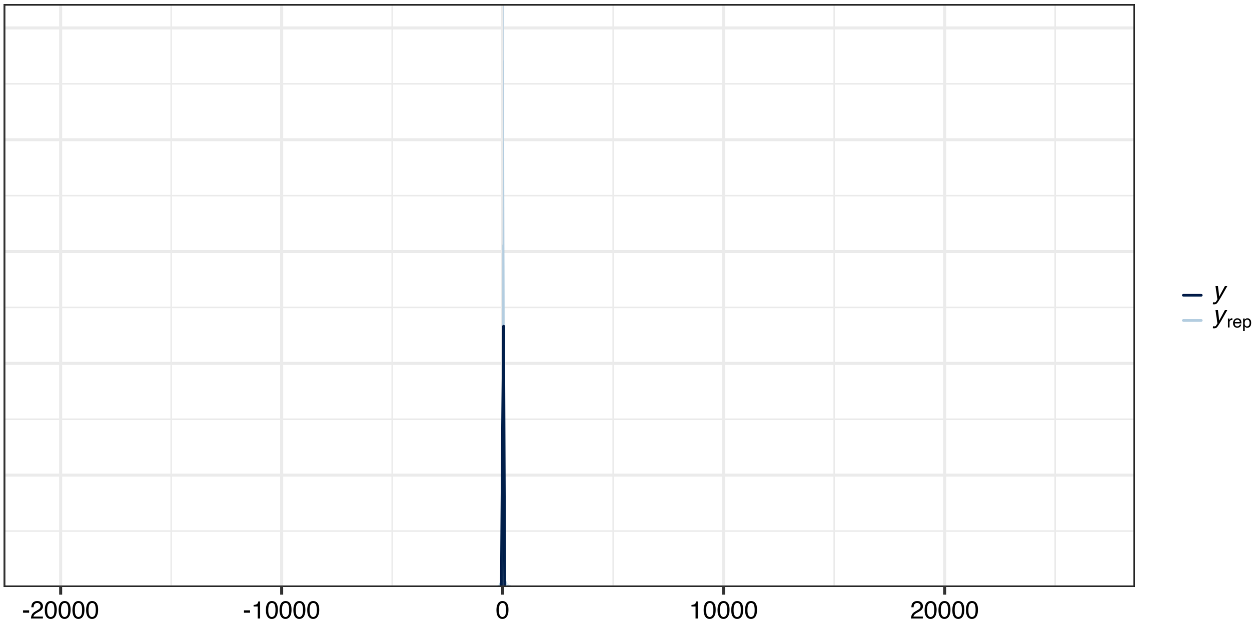


```r
pp_check(model.basque.tr.extended, nsamples=1e2)
```

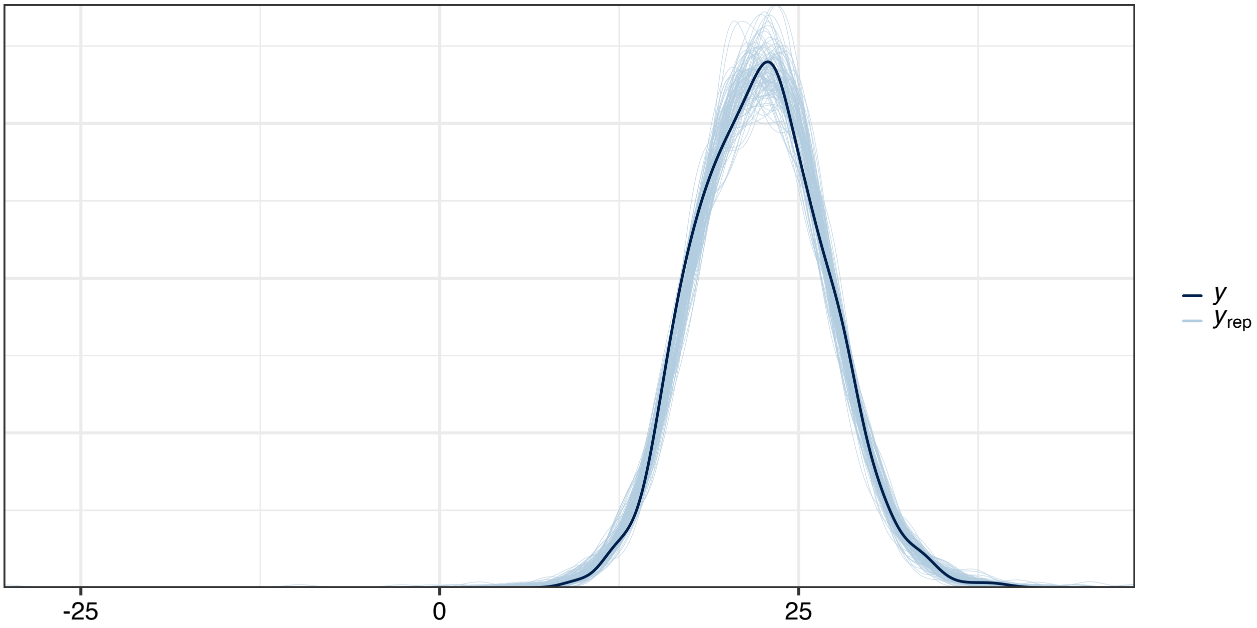


```r
loo.m.eu.tr.ext <- loo(model.basque.tr.extended)
loo.m.eu.tr.ext.null <- loo(model.null.basque.tr.extended)
```

```r
loo_model_weights(list("hypothesis" = loo.m.eu.tr.ext ,
                       "null" = loo.m.eu.tr.ext.null))
```

```
## Method: stacking
## ------
##            weight
## hypothesis 1.000
## null       0.000
```

Original stimuli, surprisal estimated by GPT-2

```r
contrasts(eu.gpt.original.m$Ambiguity)
```

```
##       Unamb
## Amb       0
## Unamb     1
```

```r
contrasts(eu.gpt.original.m$Semantic.Role)
```

```
##     Erg
## Abs   0
## Erg   1
```

```r
model.basque.gpt.original <-
  brm(surprisal ~ 1 + Semantic.Role*Ambiguity +
        (1 + Semantic.Role + Ambiguity|sent_id),
      family = student(),
      save_pars = save_pars(all = T),
      sample_prior = T,
      prior = prior.eu,
      data = eu.gpt.original.m,
      file = '../models/eu.gpt.original',
      chains = 4, iter = 8000, warmup = 2000, cores = 4,
      control = list(adapt_delta = 0.96, max_treedepth=15)
  )

model.basque.gpt.original.prior <-
  brm(surprisal ~ 1 + Semantic.Role*Ambiguity +
        (1 + Semantic.Role + Ambiguity|sent_id),
      family = student(),
      sample_prior = 'only',
      file = '../models/eu.gpt.original.prior',
      prior = prior.eu,
      data = eu.gpt.original.m
  )

model.null.basque.gpt.original <-
  brm(surprisal ~ 1 + (1|sent_id),
      family = student(),
      save_pars = save_pars(all = T),
      sample_prior = T,
```

```
      prior = prior.eu.null,
      data = eu.gpt.original.m,
      file = '../models/eu.gpt.original_null',
      chains = 4, iter = 8000, warmup = 2000, cores = 4,
      control = list(adapt_delta = 0.96, max_treedepth=15)
  )

pp_check(model.basque.gpt.original.prior, nsamples=1e2)
```

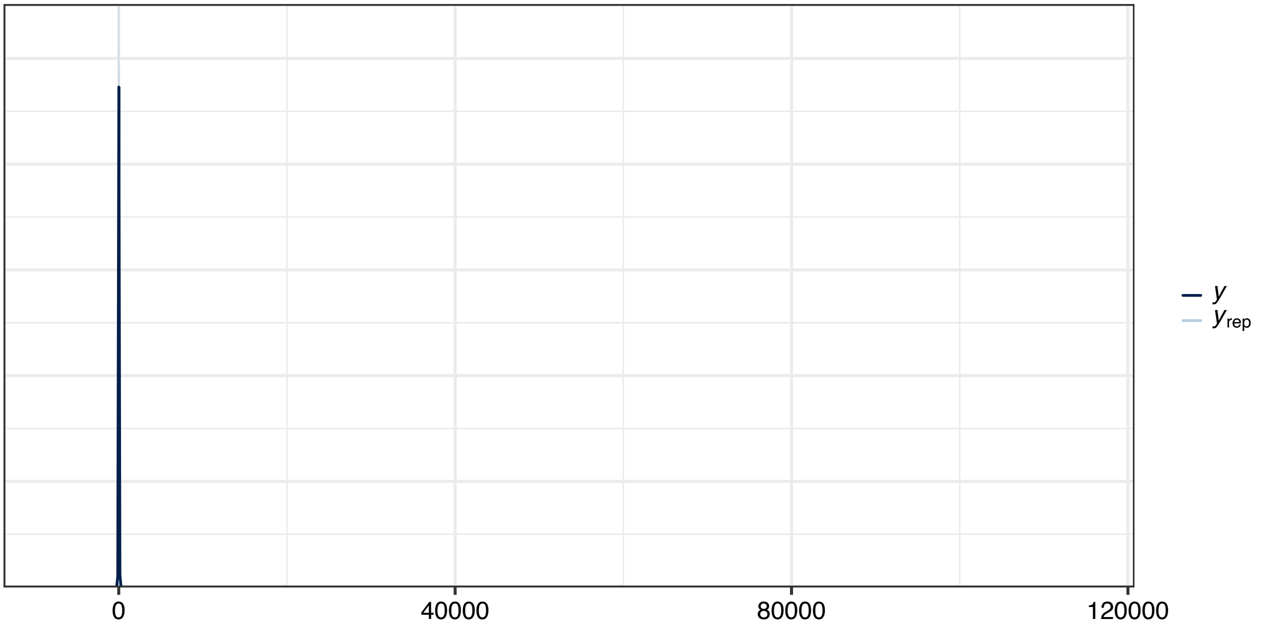


```
pp_check(model.basque.gpt.original, nsamples=1e2)
```

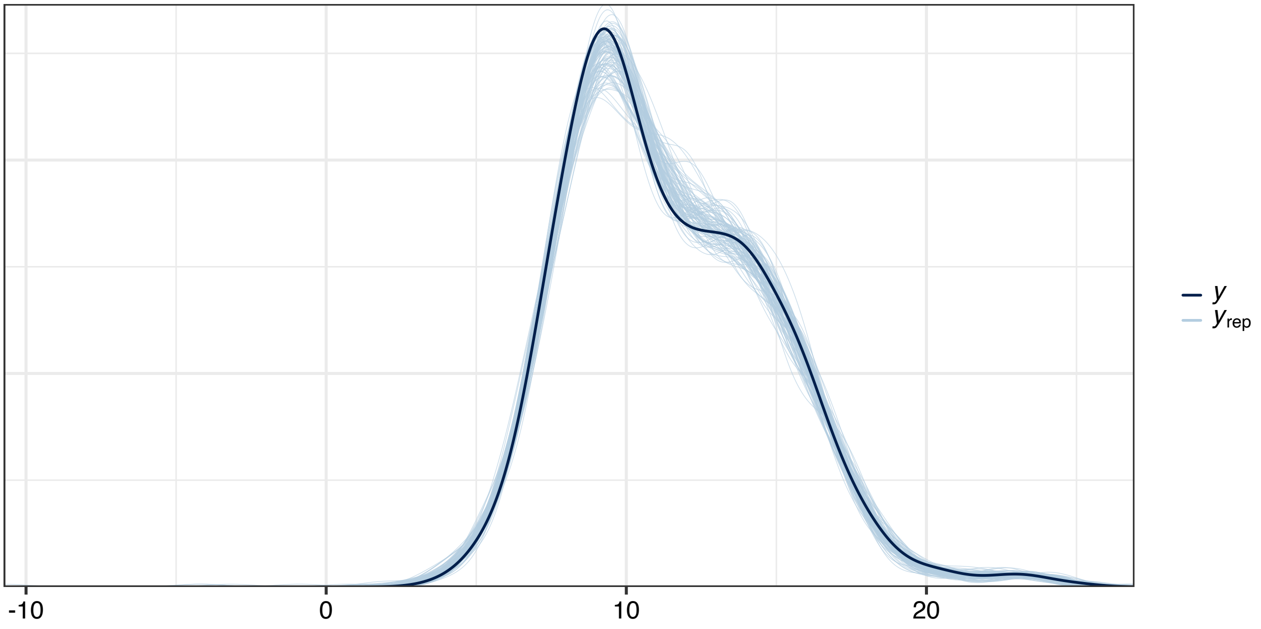

```
loo.m.eu.gpt.orig <- loo(model.basque.gpt.original)
loo.m.eu.gpt.orig.null <- loo(model.null.basque.gpt.original)

loo_model_weights(list("hypothesis" = loo.m.eu.gpt.orig ,
                       "null" = loo.m.eu.gpt.orig.null))
```

```
## Method: stacking
## ------
##            weight
## hypothesis 0.995
## null       0.005
```

Extended stimuli, surprisal estimated by GPT-2

```
contrasts(eu.gpt.extended.m$Ambiguity)
```

```
##       Unamb
## Amb       0
## Unamb     1
```

```
contrasts(eu.gpt.extended.m$Semantic.Role)
```

```
##     Erg
## Abs   0
## Erg   1
```

```
model.basque.gpt.extended <-
  brm(surprisal ~ 1 + Semantic.Role*Ambiguity +
        (1 + Semantic.Role + Ambiguity|sent_id),
      family = student(),
      save_pars = save_pars(all = T),
      sample_prior = T,
      prior = prior.eu,
      data = eu.gpt.extended.m,
      file = '../models/eu.gpt.extended',
      chains = 4, iter = 8000, warmup = 2000, cores = 4,
      control = list(adapt_delta = 0.96, max_treedepth=15)
  )

model.basque.gpt.extended.prior <-
  brm(surprisal ~ 1 + Semantic.Role*Ambiguity +
        (1 + Semantic.Role + Ambiguity|sent_id),
      family = student(),
      sample_prior = 'only',
      file = '../models/eu.gpt.extended.prior',
      prior = prior.eu,
      data = eu.gpt.extended.m
  )

model.null.basque.gpt.extended <-
  brm(surprisal ~ 1 + (1|sent_id),
```

```
      family = student(),
      save_pars = save_pars(all = T),
      sample_prior = T,
      prior = prior.eu.null,
      data = eu.gpt.extended.m,
      file = '../models/eu.gpt.extended_null',
      chains = 4, iter = 8000, warmup = 2000, cores = 4,
      control = list(adapt_delta = 0.96, max_treedepth=15)
  )

pp_check(model.basque.gpt.extended.prior, nsamples=1e2)
```

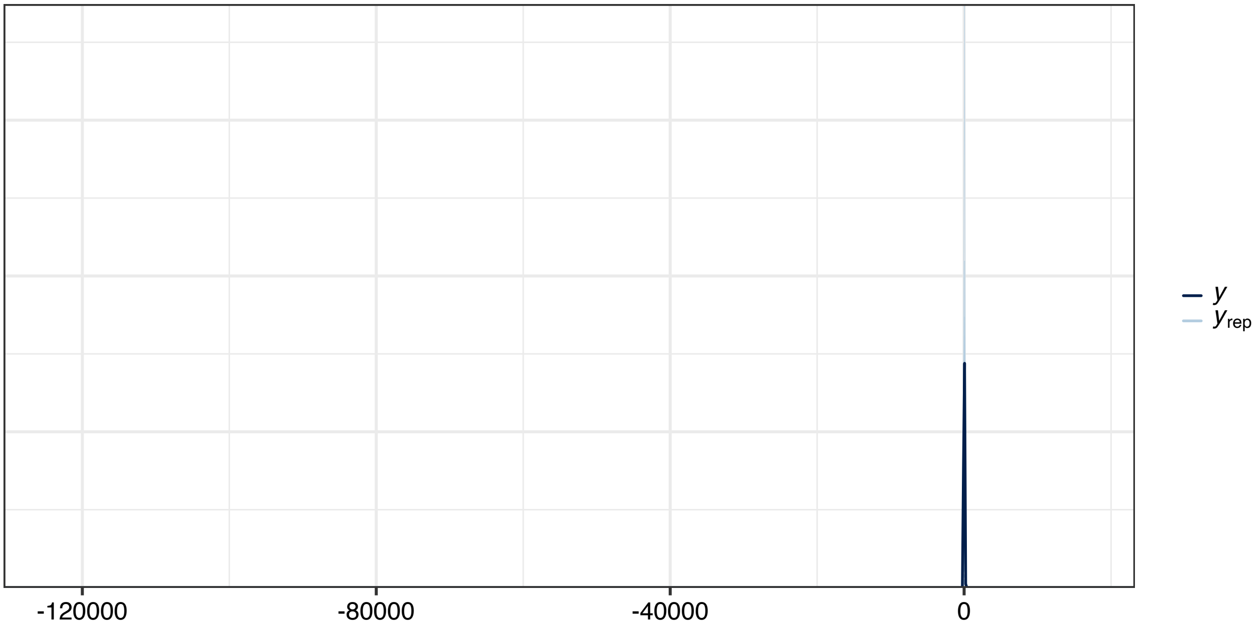


```
pp_check(model.basque.gpt.extended, nsamples=1e2)
```

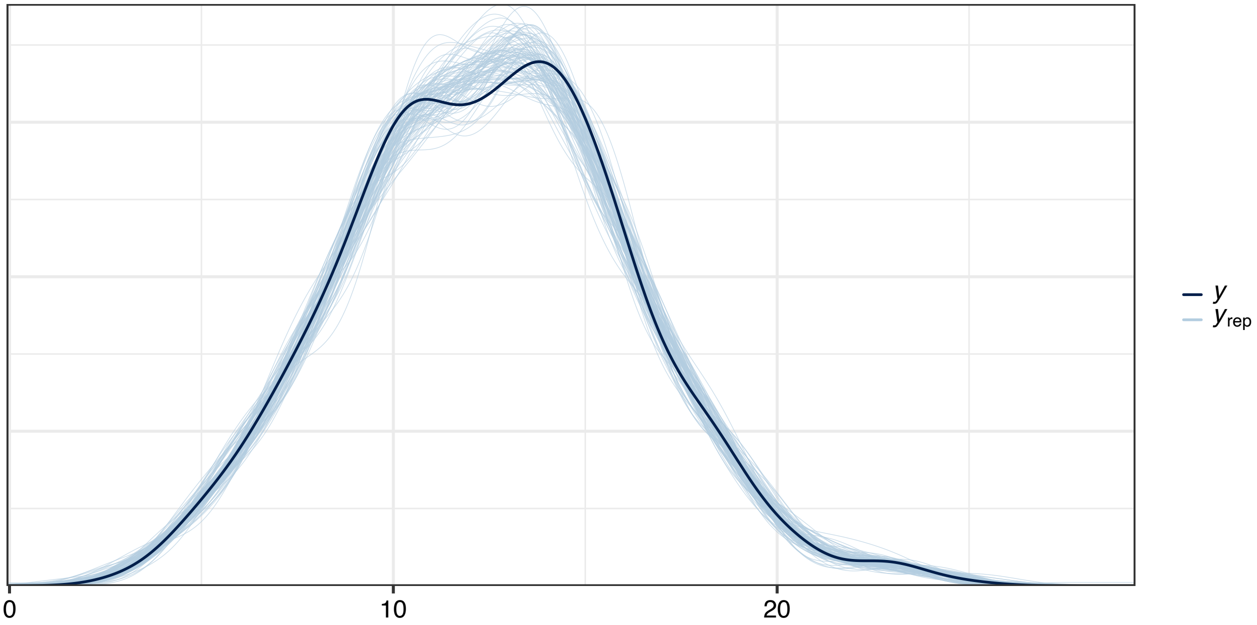

```r
loo.m.eu.gpt.ext <- loo(model.basque.gpt.extended)
loo.m.eu.gpt.ext.null <- loo(model.null.basque.gpt.extended)

loo_model_weights(list("hypothesis" = loo.m.eu.gpt.ext ,
                       "null" = loo.m.eu.gpt.ext.null))
```

```
## Method: stacking
## ------
##            weight
## hypothesis 1.000
## null       0.000
```

S3.3 Posterior Distributions

Now we sample estimated surprisal values from posterior distribution of the fitted models for each group level and main effect. We calculate the posterior distributions of *Delta Surprisal* estimates between the condition that elicited the Predicate N400 (patient initial) minus the condition where it is not expected (agent initial).

S4, S6 and S8 displays the credibility intervals and means for each model and condition for the original dataset and S5, S7 and S9 for the extended datasets. The plots in S1/S6/S11, S2/ S7/ S12 and S3/S8/S13 show posterior distributions *Delta Surprisal* for both population and group levels. S4/S9/S14 and S5/S10/S15 shows posterior predictions at the population level for both extended and original datasets. One can see that the posterior distributions of *Delta Surprisal* are similar between the original and the extended dataset.

Lastly, the results from the paired t-tests mirror the results from the multilevel Bayesian models.

```
if (!file.exists("../temp_data/post_samples_de_lstm_orig.rds")){

  draws.de.lstm <- model.de.lstm.original %>%
    spread_draws(r_sent[sent,term], b_Intercept, b_verb.typedat,
                 b_first.NPobject, `b_verb.typedat:first.NPobject`)

  # calculate estimates of each condition level
  draws.de.lstm <- draws.de.lstm  %>%
    mutate(AgentACC = b_Intercept,
           AgentDAT = b_Intercept + b_verb.typedat,
           PatientACC = b_Intercept + b_first.NPobject,
           PatientDAT = b_Intercept + b_verb.typedat + b_first.NPobject +
             `b_verb.typedat:first.NPobject`) %>%
    select(!starts_with('b')) %>%
    pivot_wider(names_from = c('sent', 'term'), values_from = r_sent) %>%
    select(!matches('.iteration|.chain'))

  draws.de.lstm <- draws.de.lstm %>%
    pivot_longer(!.draw, values_to = 'surprisal', names_to = 'Condition') %>%
    mutate(Condition = ifelse(!grepl('_', Condition),
                              paste0('Population _', Condition ), Condition)) %>%
    separate(Condition, c('Level', 'Condition'), sep='_')

  draws.de.lstm.pop <- draws.de.lstm %>% filter(Level %in% 'Population ')
  draws.de.lstm.randomlevel <- draws.de.lstm %>%
    filter(!Level %in% 'Population ')

  draws.de.lstm.pop <- draws.de.lstm.pop %>%
    pivot_wider(names_from = Condition, values_from = surprisal)

  de.lstm.pop.diff <- draws.de.lstm.pop %>%
    mutate(diff_ACC = PatientACC - AgentACC) %>%
    mutate(diff_DAT = PatientDAT - AgentDAT) %>%
    select(.draw, Level, diff_ACC, diff_DAT)

  #AgentDAT, AgentACC, PatientDAT, PatientACC

  draws.de.lstm.randomlevel <- draws.de.lstm.randomlevel %>%
    group_by(.draw, Level) %>%
    mutate('PatientACC' = surprisal[Condition == 'Intercept'] +
             surprisal[Condition=='first.NPobject']) %>%
```

```r
    mutate('PatienDAT' = surprisal[Condition == 'first.NPobject'] +
             surprisal[Condition == 'verb.typedat'] ) %>%
    mutate('AgentACC' = surprisal[Condition == 'Intercept']) %>%
    mutate('AgentDAT'= surprisal[Condition == 'Intercept'] +
             surprisal[Condition == 'verb.typedat']) %>%
    select(-Condition, -surprisal) %>%
    distinct()



  de.lstm.randomlevel.diff <- draws.de.lstm.randomlevel %>%
    mutate(diff_DAT = PatienDAT - AgentDAT) %>%
    mutate(diff_ACC = PatientACC - AgentACC) %>%
    select(.draw, Level, diff_ACC, diff_DAT)


  draws.de.lstm.all <- rbind(de.lstm.pop.diff, de.lstm.randomlevel.diff)

  draws.de.lstm.all.long <- draws.de.lstm.all %>%
    select(diff_DAT, diff_ACC, Level, .draw) %>%
    pivot_longer(!c('Level', '.draw'), names_to = 'Condition',
                 values_to = 'delta_surprisal')



  saveRDS(draws.de.lstm.all.long, '../temp_data/post_samples_de_lstm_orig.rds')
}

if (!file.exists('../temp_data/post_samples_de_lstm_ext.rds')){
  draws.de.lstm <- model.de.lstm.extended %>%
    spread_draws(r_sent_id[sent_id,term], b_Intercept,
                 b_verb.typedat,b_first.NPobject,
                 `b_verb.typedat:first.NPobject`)


  draws.de.lstm <- draws.de.lstm  %>%
    mutate(AgentACC = b_Intercept,
           AgentDAT = b_Intercept + b_verb.typedat,
           PatientACC = b_Intercept + b_first.NPobject,
           PatientDAT = b_Intercept + b_verb.typedat + b_first.NPobject +
             `b_verb.typedat:first.NPobject`) %>%
    select(!starts_with('b')) %>%
    pivot_wider(names_from = c('sent_id', 'term'), values_from = r_sent_id) %>%
    select(!matches('.iteration|.chain'))

  draws.de.lstm <- draws.de.lstm %>%
    pivot_longer(!.draw, values_to = 'surprisal', names_to = 'Condition') %>%
    mutate(Condition = ifelse(!grepl('_', Condition),
                              paste0('Population _', Condition ), Condition)) %>%
    separate(Condition, c('Level', 'Condition'), sep='_')

  draws.de.lstm.pop <- draws.de.lstm %>% filter(Level %in% 'Population ')
```

```
  draws.de.lstm.pop <- draws.de.lstm.pop %>%
    pivot_wider(names_from = Condition, values_from = surprisal)

  de.lstm.pop.diff <- draws.de.lstm.pop %>%
    mutate(diff_ACC = PatientACC - AgentACC) %>%
    mutate(diff_DAT = PatientDAT - AgentDAT) %>%
    select(.draw, Level, diff_ACC, diff_DAT)


  draws.de.lstm.ext <- de.lstm.pop.diff %>%
    select(diff_DAT, diff_ACC, Level, .draw) %>%
    pivot_longer(!c('Level', '.draw'), names_to = 'Condition',
                 values_to = 'delta_surprisal')



  saveRDS(draws.de.lstm.ext, '../temp_data/post_samples_de_lstm_ext.rds')
}

if (!file.exists('../temp_data/post_samples_de_tr_orig.rds')){
  draws.de.tr <- model.de.tr.original %>%
    spread_draws(r_sent_id[sent_id,term], b_Intercept, b_verb.typedat,
                 b_first.NPobject, `b_verb.typedat:first.NPobject`)


  draws.de.tr <- draws.de.tr  %>%
    mutate(AgentACC = b_Intercept,
           AgentDAT = b_Intercept + b_verb.typedat,
           PatientACC = b_Intercept + b_first.NPobject,
           PatientDAT = b_Intercept + b_verb.typedat + b_first.NPobject +
             `b_verb.typedat:first.NPobject`) %>%
    select(!starts_with('b')) %>%
    pivot_wider(names_from = c('sent', 'term'), values_from = r_sent) %>%
    select(!matches('.iteration|.chain'))

  draws.de.tr <- draws.de.tr %>%
    pivot_longer(!.draw, values_to = 'surprisal', names_to = 'Condition') %>%
    mutate(Condition = ifelse(!grepl('_', Condition) ,
                              paste0('Population _', Condition ), Condition)) %>%
    separate(Condition, c('Level', 'Condition'), sep='_')

  draws.de.tr.pop <- draws.de.tr %>% filter(Level %in% 'Population ')
  draws.de.tr.randomlevel <- draws.de.tr %>% filter(!Level %in% 'Population ')

  draws.de.tr.pop <- draws.de.tr.pop %>%
    pivot_wider(names_from = Condition, values_from = surprisal)

  de.tr.pop.diff <- draws.de.tr.pop %>%
    mutate(diff_ACC = PatientACC - AgentACC) %>%
    mutate(diff_DAT = PatientDAT - AgentDAT) %>%
    select(.draw, Level, diff_ACC, diff_DAT)

  #AgentDAT, AgentACC, PatientDAT, PatientACC
```

```
  draws.de.tr.randomlevel <- draws.de.tr.randomlevel %>%
    group_by(.draw, Level) %>%
    mutate('PatientACC' = surprisal[Condition == 'Intercept'] +
             surprisal[Condition=='first.NPobject']) %>%
    mutate('PatienDAT' = surprisal[Condition == 'first.NPobject'] +
             surprisal[Condition == 'verb.typedat'] ) %>%
    mutate('AgentACC' = surprisal[Condition == 'Intercept']) %>%
    mutate('AgentDAT'= surprisal[Condition == 'Intercept'] +
             surprisal[Condition == 'verb.typedat']) %>%
    select(-Condition, -surprisal) %>%
    distinct()



  de.tr.randomlevel.diff <- draws.de.tr.randomlevel %>%
    mutate(diff_DAT = PatienDAT - AgentDAT) %>%
    mutate(diff_ACC = PatientACC - AgentACC) %>%
    select(.draw, Level, diff_ACC, diff_DAT)


  draws.de.tr.all <- rbind(de.tr.pop.diff, de.tr.randomlevel.diff)

  draws.de.tr.all.long <- draws.de.tr.all %>%
    select(diff_DAT, diff_ACC, Level, .draw) %>%
    pivot_longer(!c('Level', '.draw'), names_to = 'Condition',
                 values_to = 'delta_surprisal')

  saveRDS(draws.de.tr.all.long, '../temp_data/post_samples_de_tr_orig.rds')
}

if (!file.exists('../temp_data/post_samples_de_tr_ext.rds')){
  draws.de.tr <- model.de.tr.extended %>%
    spread_draws(r_sent_id[sent_id,term], b_Intercept,
                 b_verb.typedat,b_first.NPobject, `b_verb.typedat:first.NPobject`)


  draws.de.tr <- draws.de.tr  %>%
    mutate(AgentACC = b_Intercept,
           AgentDAT = b_Intercept + b_verb.typedat,
           PatientACC = b_Intercept + b_first.NPobject,
           PatientDAT = b_Intercept + b_verb.typedat + b_first.NPobject +
             `b_verb.typedat:first.NPobject`) %>%
    select(!starts_with('b')) %>%
    pivot_wider(names_from = c('sent_id', 'term'), values_from = r_sent_id) %>%
    select(!matches('.iteration|.chain'))

  draws.de.tr <- draws.de.tr %>%
    pivot_longer(!.draw, values_to = 'surprisal', names_to = 'Condition') %>%
    mutate(Condition = ifelse(!grepl('_', Condition),
                              paste0('Population _', Condition ), Condition)) %>%
    separate(Condition, c('Level', 'Condition'), sep='_')

  draws.de.tr.pop <- draws.de.tr %>% filter(Level %in% 'Population ')
```

```r
  draws.de.tr.pop <- draws.de.tr.pop %>%
    pivot_wider(names_from = Condition, values_from = surprisal)

  de.tr.pop.diff <- draws.de.tr.pop %>%
    mutate(diff_ACC = PatientACC - AgentACC) %>%
    mutate(diff_DAT = PatientDAT - AgentDAT) %>%
    select(.draw, Level, diff_ACC, diff_DAT)

  draws.de.tr.ext <- de.tr.pop.diff%>%
    select(diff_DAT, diff_ACC, Level, .draw) %>%
    pivot_longer(!c('Level', '.draw'), names_to = 'Condition',
                 values_to = 'delta_surprisal')

  saveRDS(draws.de.tr.ext, '../temp_data/post_samples_de_tr_ext.rds')

}

if (!file.exists('../temp_data/post_samples_de_gpt_orig.rds')) {
  draws.de.gpt <- model.de.gpt.original %>%
    spread_draws(r_sent[sent,term], b_Intercept,
                 b_verb.typedat,b_first.NPobject,
                 `b_verb.typedat:first.NPobject`)

  # calculate estimates of each condition level
  draws.de.gpt <- draws.de.gpt  %>%
    mutate(AgentACC = b_Intercept,
           AgentDAT = b_Intercept + b_verb.typedat,
           PatientACC = b_Intercept + b_first.NPobject,
           PatientDAT = b_Intercept + b_verb.typedat + b_first.NPobject +
             `b_verb.typedat:first.NPobject`) %>%
    select(!starts_with('b')) %>%
    pivot_wider(names_from = c('sent', 'term'), values_from = r_sent) %>%
    select(!matches('.iteration|.chain'))

  draws.de.gpt <- draws.de.gpt %>%
    pivot_longer(!.draw, values_to = 'surprisal', names_to = 'Condition') %>%
    mutate(Condition = ifelse(!grepl('_', Condition) ,
                              paste0('Population _', Condition ), Condition)) %>%
    separate(Condition, c('Level', 'Condition'), sep='_')

  draws.de.gpt.pop <- draws.de.gpt %>% filter(Level %in% 'Population ')
  draws.de.gpt.randomlevel <- draws.de.gpt %>% filter(!Level %in% 'Population ')

  draws.de.gpt.pop <- draws.de.gpt.pop %>%
    pivot_wider(names_from = Condition, values_from = surprisal)

  de.gpt.pop.diff <- draws.de.gpt.pop %>%
    mutate(diff_ACC = PatientACC - AgentACC) %>%
    mutate(diff_DAT = PatientDAT - AgentDAT) %>%
    select(.draw, Level, diff_ACC, diff_DAT)

  #AgentDAT, AgentACC, PatientDAT, PatientACC
```

```
  draws.de.gpt.randomlevel <- draws.de.gpt.randomlevel %>%
    group_by(.draw, Level) %>%
    mutate('PatientACC' = surprisal[Condition == 'Intercept'] +
             surprisal[Condition=='first.NPobject']) %>%
    mutate('PatienDAT' = surprisal[Condition == 'first.NPobject'] +
             surprisal[Condition == 'verb.typedat'] ) %>%
    mutate('AgentACC' = surprisal[Condition == 'Intercept']) %>%
    mutate('AgentDAT'= surprisal[Condition == 'Intercept'] +
             surprisal[Condition == 'verb.typedat']) %>%
    select(-Condition, -surprisal) %>%
    distinct()



  de.gpt.randomlevel.diff <- draws.de.gpt.randomlevel %>%
    mutate(diff_DAT = PatienDAT - AgentDAT) %>%
    mutate(diff_ACC = PatientACC - AgentACC) %>%
    select(.draw, Level, diff_ACC, diff_DAT)


  draws.de.gpt.all <- rbind(de.gpt.pop.diff, de.gpt.randomlevel.diff)

  draws.de.gpt.all.long <- draws.de.gpt.all %>%
    select(diff_DAT, diff_ACC, Level, .draw) %>%
    pivot_longer(!c('Level', '.draw'), names_to = 'Condition',
                 values_to = 'delta_surprisal')


  saveRDS(draws.de.gpt.all.long, '../temp_data/post_samples_de_gpt_orig.rds')

}


if (!file.exists('../temp_data/post_samples_de_gpt_ext.rds')) {
  draws.de.gpt <- model.de.gpt.extended %>%
    spread_draws(r_sent_id[sent_id,term], b_Intercept,
                 b_verb.typedat,b_first.NPobject, `b_verb.typedat:first.NPobject`)


  draws.de.gpt <- draws.de.gpt  %>%
    mutate(AgentACC = b_Intercept,
           AgentDAT = b_Intercept + b_verb.typedat,
           PatientACC = b_Intercept + b_first.NPobject,
           PatientDAT = b_Intercept + b_verb.typedat + b_first.NPobject +
             `b_verb.typedat:first.NPobject`) %>%
    select(!starts_with('b')) %>%
    pivot_wider(names_from = c('sent_id', 'term'), values_from = r_sent_id) %>%
    select(!matches('.iteration|.chain'))

  draws.de.gpt <- draws.de.gpt %>%
    pivot_longer(!.draw, values_to = 'surprisal', names_to = 'Condition') %>%
    mutate(Condition = ifelse(!grepl('_', Condition),
                              paste0('Population _', Condition ), Condition)) %>%
```

```
    separate(Condition, c('Level', 'Condition'), sep='_')

  draws.de.gpt.pop <- draws.de.gpt %>% filter(Level %in% 'Population ')

  draws.de.gpt.pop <- draws.de.gpt.pop %>%
    pivot_wider(names_from = Condition, values_from = surprisal)

  de.gpt.pop.diff <- draws.de.gpt.pop %>%
    mutate(diff_ACC = PatientACC - AgentACC) %>%
    mutate(diff_DAT = PatientDAT - AgentDAT) %>%
    select(.draw, Level, diff_ACC, diff_DAT)

  draws.de.gpt.ext <- de.gpt.pop.diff %>%
    select(diff_DAT, diff_ACC, Level, .draw) %>%
    pivot_longer(!c('Level', '.draw'), names_to = 'Condition',
                 values_to = 'delta_surprisal')


  saveRDS(draws.de.gpt.ext, '../temp_data/post_samples_de_gpt_ext.rds')

}




if (!file.exists("../temp_data/post_samples_hi_lstm_orig.rds")){

  draws.hi.lstm <- model.hi.lstm.original %>%
    spread_draws(r_sent_id[sent_id,term], b_Intercept,
                 b_ambiguityUNAMB, b_aspectPFV, `b_ambiguityUNAMB:aspectPFV`)

  # calculate estimates of each condition level
  draws.hi.lstm <- draws.hi.lstm  %>%
    mutate(AmbIpfv = b_Intercept,
           UnambIpfv = b_Intercept + b_ambiguityUNAMB,
           AmbPfv = b_Intercept + b_aspectPFV,
           UnambPfv = b_Intercept +  b_ambiguityUNAMB +
             b_aspectPFV + `b_ambiguityUNAMB:aspectPFV`) %>%
    select(!starts_with('b')) %>%
    pivot_wider(names_from = c('sent_id', 'term'), values_from = r_sent_id) %>%
    select(!matches('.iteration|.chain'))

  draws.hi.lstm <- draws.hi.lstm %>%
    pivot_longer(!.draw, values_to = 'surprisal', names_to = 'Condition') %>%
    mutate(Condition = ifelse(!grepl('_', Condition) ,
                              paste0('Population _', Condition ), Condition)) %>%
    separate(Condition, c('Level', 'Condition'), sep='_')

  draws.hi.lstm.pop <- draws.hi.lstm %>% filter(Level %in% 'Population ')
  draws.hi.lstm.randomlevel <- draws.hi.lstm %>%
    filter(!Level %in% 'Population ')

  draws.hi.lstm.pop <- draws.hi.lstm.pop %>%
```

```
    pivot_wider(names_from = Condition, values_from = surprisal)

  hi.lstm.pop.diff <- draws.hi.lstm.pop %>%
    mutate(diff_Pfv = AmbPfv - UnambPfv) %>%
    mutate(diff_Ipfv = AmbIpfv - UnambIpfv) %>%
    select(.draw, Level, diff_Pfv, diff_Ipfv)

  #AgentDAT, AgentACC, PatientDAT, PatientACC

  draws.hi.lstm.randomlevel <- draws.hi.lstm.randomlevel %>%
    group_by(.draw, Level) %>%
    mutate('UnambPfv' = surprisal[Condition == 'ambiguityUNAMB'] +
             surprisal[Condition=='aspectPFV']) %>%
    mutate('AmbPfv' = surprisal[Condition == 'aspectPFV'] +
             surprisal[Condition == 'Intercept'] ) %>%
    mutate('AmbIpfv' = surprisal[Condition == 'Intercept']) %>%
    mutate('UnambIpfv'= surprisal[Condition == 'Intercept'] +
             surprisal[Condition == 'ambiguityUNAMB']) %>%
    select(-Condition, -surprisal) %>%
    distinct()



  hi.lstm.randomlevel.diff <- draws.hi.lstm.randomlevel %>%
    mutate(diff_Pfv = AmbPfv - UnambPfv) %>%
    mutate(diff_Ipfv = AmbIpfv - UnambIpfv) %>%
    select(.draw, Level, diff_Pfv, diff_Ipfv)


  draws.hi.lstm.all <- rbind(hi.lstm.pop.diff, hi.lstm.randomlevel.diff)

  draws.hi.lstm.all.long <- draws.hi.lstm.all %>%
    select(diff_Pfv, diff_Ipfv, Level, .draw) %>%
    pivot_longer(!c('Level', '.draw'), names_to = 'Condition',
                 values_to = 'delta_surprisal')


  saveRDS(draws.hi.lstm.all.long, '../temp_data/post_samples_hi_lstm_orig.rds')
}

if (!file.exists('../temp_data/post_samples_hi_lstm_ext.rds')){

  draws.hi.lstm <- model.hi.lstm.extended %>%
    spread_draws(r_sent_id[sent_id,term], b_Intercept,
                 b_ambiguityUNAMB, b_aspectPFV, `b_ambiguityUNAMB:aspectPFV`)

  # calculate estimates of each condition level
  draws.hi.lstm <- draws.hi.lstm %>%
    mutate(AmbIpfv = b_Intercept,
           UnambIpfv = b_Intercept + b_ambiguityUNAMB,
           AmbPfv = b_Intercept + b_aspectPFV,
           UnambPfv = b_Intercept +  b_ambiguityUNAMB +
             b_aspectPFV + `b_ambiguityUNAMB:aspectPFV`) %>%
```

```
    select(!starts_with('b')) %>%
    pivot_wider(names_from = c('sent_id', 'term'), values_from = r_sent_id) %>%
    select(!matches('.iteration|.chain'))

  draws.hi.lstm <- draws.hi.lstm %>%
    pivot_longer(!.draw, values_to = 'surprisal', names_to = 'Condition') %>%
    mutate(Condition = ifelse(!grepl('_', Condition) ,
                              paste0('Population _', Condition ), Condition)) %>%
    separate(Condition, c('Level', 'Condition'), sep='_')

  draws.hi.lstm.pop <- draws.hi.lstm %>% filter(Level %in% 'Population ')

  draws.hi.lstm.pop <- draws.hi.lstm.pop %>%
    pivot_wider(names_from = Condition, values_from = surprisal)

  hi.lstm.pop.diff <- draws.hi.lstm.pop %>%
    mutate(diff_Pfv = AmbPfv - UnambPfv) %>%
    mutate(diff_Ipfv = AmbIpfv - UnambIpfv) %>%
    select(.draw, Level, diff_Pfv, diff_Ipfv)


  draws.hi.lstm.ext <- hi.lstm.pop.diff %>%
    select(diff_Pfv, diff_Ipfv, Level, .draw) %>%
    pivot_longer(!c('Level', '.draw'), names_to = 'Condition',
                 values_to = 'delta_surprisal')


  saveRDS(draws.hi.lstm.ext, '../temp_data/post_samples_hi_lstm_ext.rds')
}

if (!file.exists('../temp_data/post_samples_hi_bert_orig.rds')){
  draws.hi.tr <- model.hi.bert.original %>%
    spread_draws(r_sent_id[sent_id,term], b_Intercept,
                 b_ambiguityUNAMB, b_aspectPFV, `b_ambiguityUNAMB:aspectPFV`)

  # calculate estimates of each condition level
  draws.hi.tr <- draws.hi.tr  %>%
    mutate(AmbIpfv = b_Intercept,
           UnambIpfv = b_Intercept + b_ambiguityUNAMB,
           AmbPfv = b_Intercept + b_aspectPFV,
           UnambPfv = b_Intercept +  b_ambiguityUNAMB +
             b_aspectPFV + `b_ambiguityUNAMB:aspectPFV`) %>%
    select(!starts_with('b')) %>%
    pivot_wider(names_from = c('sent_id', 'term'), values_from = r_sent_id) %>%
    select(!matches('.iteration|.chain'))

  draws.hi.tr <- draws.hi.tr %>%
    pivot_longer(!.draw, values_to = 'surprisal', names_to = 'Condition') %>%
    mutate(Condition = ifelse(!grepl('_', Condition) ,
                              paste0('Population _', Condition ), Condition)) %>%
    separate(Condition, c('Level', 'Condition'), sep='_')

  draws.hi.tr.pop <- draws.hi.tr %>% filter(Level %in% 'Population ')
```

```
  draws.hi.tr.randomlevel <- draws.hi.tr %>% filter(!Level %in% 'Population ')

  draws.hi.tr.pop <- draws.hi.tr.pop %>%
    pivot_wider(names_from = Condition, values_from = surprisal)

  hi.tr.pop.diff <- draws.hi.tr.pop %>%
    mutate(diff_Pfv = AmbPfv - UnambPfv) %>%
    mutate(diff_Ipfv = AmbIpfv - UnambIpfv) %>%
    select(.draw, Level, diff_Pfv, diff_Ipfv)

  #AgentDAT, AgentACC, PatientDAT, PatientACC

  draws.hi.tr.randomlevel <- draws.hi.tr.randomlevel %>%
    group_by(.draw, Level) %>%
    mutate('UnambPfv' = surprisal[Condition == 'ambiguityUNAMB'] +
             surprisal[Condition=='aspectPFV']) %>%
    mutate('AmbPfv' = surprisal[Condition == 'aspectPFV'] +
             surprisal[Condition == 'Intercept'] ) %>%
    mutate('AmbIpfv' = surprisal[Condition == 'Intercept']) %>%
    mutate('UnambIpfv'= surprisal[Condition == 'Intercept'] +
             surprisal[Condition == 'ambiguityUNAMB']) %>%
    select(-Condition, -surprisal) %>%
    distinct()



  hi.tr.randomlevel.diff <- draws.hi.tr.randomlevel %>%
    mutate(diff_Pfv = AmbPfv - UnambPfv) %>%
    mutate(diff_Ipfv = AmbIpfv - UnambIpfv) %>%
    select(.draw, Level, diff_Pfv, diff_Ipfv)


  draws.hi.tr.all <- rbind(hi.tr.pop.diff, hi.tr.randomlevel.diff)

  draws.hi.tr.all.long <- draws.hi.tr.all %>%
    select(diff_Pfv, diff_Ipfv, Level, .draw) %>%
    pivot_longer(!c('Level', '.draw'), names_to = 'Condition',
                 values_to = 'delta_surprisal')

  saveRDS(draws.hi.tr.all.long, '../temp_data/post_samples_hi_bert_orig.rds')
}

if (!file.exists('../temp_data/post_samples_hi_tr_ext.rds')){
  draws.hi.tr <- model.hi.bert.extended %>%
    spread_draws(r_sent_id[sent_id,term], b_Intercept, b_ambiguityUNAMB,
                 b_aspectPFV, `b_ambiguityUNAMB:aspectPFV`)

  # calculate estimates of each condition level
  draws.hi.tr <- draws.hi.tr  %>%
    mutate(AmbIpfv = b_Intercept,
           UnambIpfv = b_Intercept + b_ambiguityUNAMB,
           AmbPfv = b_Intercept + b_aspectPFV,
           UnambPfv = b_Intercept +  b_ambiguityUNAMB + b_aspectPFV +
```

```
              `b_ambiguityUNAMB:aspectPFV`) %>%
    select(!starts_with('b')) %>%
    pivot_wider(names_from = c('sent_id', 'term'), values_from = r_sent_id) %>%
    select(!matches('.iteration|.chain'))

  draws.hi.tr <- draws.hi.tr %>%
    pivot_longer(!.draw, values_to = 'surprisal', names_to = 'Condition') %>%
    mutate(Condition = ifelse(!grepl('_', Condition) ,
                              paste0('Population _', Condition ), Condition)) %>%
    separate(Condition, c('Level', 'Condition'), sep='_')

  draws.hi.tr.pop <- draws.hi.tr %>% filter(Level %in% 'Population ')

  draws.hi.tr.pop <- draws.hi.tr.pop %>%
    pivot_wider(names_from = Condition, values_from = surprisal)

  hi.tr.pop.diff <- draws.hi.tr.pop %>%
    mutate(diff_Pfv = AmbPfv - UnambPfv) %>%
    mutate(diff_Ipfv = AmbIpfv - UnambIpfv) %>%
    select(.draw, Level, diff_Pfv, diff_Ipfv)


  draws.hi.tr.ext <- hi.tr.pop.diff %>%
    select(diff_Pfv, diff_Ipfv, Level, .draw) %>%
    pivot_longer(!c('Level', '.draw'),
                 names_to = 'Condition', values_to = 'delta_surprisal')

  saveRDS(draws.hi.tr.ext, '../temp_data/post_samples_hi_tr_ext.rds')
}

if (!file.exists('../temp_data/post_samples_hi_gpt_orig.rds')){

  draws.hi.gpt <- model.hi.gpt.original %>%
    spread_draws(r_sent_id[sent_id,term], b_Intercept,
                 b_ambiguityUNAMB, b_aspectPFV, `b_ambiguityUNAMB:aspectPFV`)

  # calculate estimates of each condition level
  draws.hi.gpt <- draws.hi.gpt  %>%
    mutate(AmbIpfv = b_Intercept,
           UnambIpfv = b_Intercept + b_ambiguityUNAMB,
           AmbPfv = b_Intercept + b_aspectPFV,
           UnambPfv = b_Intercept +  b_ambiguityUNAMB + b_aspectPFV +
             `b_ambiguityUNAMB:aspectPFV`) %>%
    select(!starts_with('b')) %>%
    pivot_wider(names_from = c('sent_id', 'term'), values_from = r_sent_id) %>%
    select(!matches('.iteration|.chain'))

  draws.hi.gpt <- draws.hi.gpt %>%
    pivot_longer(!.draw, values_to = 'surprisal', names_to = 'Condition') %>%
    mutate(Condition = ifelse(!grepl('_', Condition) ,
                              paste0('Population _', Condition ), Condition)) %>%
    separate(Condition, c('Level', 'Condition'), sep='_')
```

```
  draws.hi.gpt.pop <- draws.hi.gpt %>% filter(Level %in% 'Population ')
  draws.hi.gpt.randomlevel <- draws.hi.gpt %>% filter(!Level %in% 'Population ')

  draws.hi.gpt.pop <- draws.hi.gpt.pop %>%
    pivot_wider(names_from = Condition, values_from = surprisal)

  hi.gpt.pop.diff <- draws.hi.gpt.pop %>%
    mutate(diff_Pfv = AmbPfv - UnambPfv) %>%
    mutate(diff_Ipfv = AmbIpfv - UnambIpfv) %>%
    select(.draw, Level, diff_Pfv, diff_Ipfv)

  #AgentDAT, AgentACC, PatientDAT, PatientACC

  draws.hi.gpt.randomlevel <- draws.hi.gpt.randomlevel %>%
    group_by(.draw, Level) %>%
    mutate('UnambPfv' = surprisal[Condition == 'ambiguityUNAMB'] +
             surprisal[Condition=='aspectPFV']) %>%
    mutate('AmbPfv' = surprisal[Condition == 'aspectPFV'] +
             surprisal[Condition == 'Intercept'] ) %>%
    mutate('AmbIpfv' = surprisal[Condition == 'Intercept']) %>%
    mutate('UnambIpfv'= surprisal[Condition == 'Intercept'] +
             surprisal[Condition == 'ambiguityUNAMB']) %>%
    select(-Condition, -surprisal) %>%
    distinct()



  hi.gpt.randomlevel.diff <- draws.hi.gpt.randomlevel %>%
    mutate(diff_Pfv = AmbPfv - UnambPfv) %>%
    mutate(diff_Ipfv = AmbIpfv - UnambIpfv) %>%
    select(.draw, Level, diff_Pfv, diff_Ipfv)


  draws.hi.gpt.all <- rbind(hi.gpt.pop.diff, hi.gpt.randomlevel.diff)

  draws.hi.gpt.all.long <- draws.hi.gpt.all %>%
    select(diff_Pfv, diff_Ipfv, Level, .draw) %>%
    pivot_longer(!c('Level', '.draw'), names_to = 'Condition',
                 values_to = 'delta_surprisal')

  saveRDS(draws.hi.gpt.all.long, '../temp_data/post_samples_hi_gpt_orig.rds')
}

if (!file.exists('../temp_data/post_samples_hi_gpt_ext.rds')){
  draws.hi.gpt <- model.hi.gpt.extended %>%
    spread_draws(r_sent_id[sent_id,term], b_Intercept, b_ambiguityUNAMB,
                 b_aspectPFV, `b_ambiguityUNAMB:aspectPFV`)

  # calculate estimates of each condition level
  draws.hi.gpt <- draws.hi.gpt  %>%
    mutate(AmbIpfv = b_Intercept, UnambIpfv = b_Intercept + b_ambiguityUNAMB,
           AmbPfv = b_Intercept + b_aspectPFV,
           UnambPfv = b_Intercept +  b_ambiguityUNAMB + b_aspectPFV +
```

```
            `b_ambiguityUNAMB:aspectPFV`) %>%
    select(!starts_with('b')) %>%
    pivot_wider(names_from = c('sent_id', 'term'), values_from = r_sent_id) %>%
    select(!matches('.iteration|.chain'))

  draws.hi.gpt <- draws.hi.gpt %>%
    pivot_longer(!.draw, values_to = 'surprisal', names_to = 'Condition') %>%
    mutate(Condition = ifelse(!grepl('_', Condition) ,
                              paste0('Population _', Condition ), Condition)) %>%
    separate(Condition, c('Level', 'Condition'), sep='_')

  draws.hi.gpt.pop <- draws.hi.gpt %>% filter(Level %in% 'Population ')

  draws.hi.gpt.pop <- draws.hi.gpt.pop %>%
    pivot_wider(names_from = Condition, values_from = surprisal)

  hi.gpt.pop.diff <- draws.hi.gpt.pop %>%
    mutate(diff_Pfv = AmbPfv - UnambPfv) %>%
    mutate(diff_Ipfv = AmbIpfv - UnambIpfv) %>%
    select(.draw, Level, diff_Pfv, diff_Ipfv)

  hi.gpt.pop.ext <- hi.gpt.pop.diff %>%
    select(diff_Pfv, diff_Ipfv, Level, .draw) %>%
    pivot_longer(!c('Level', '.draw'), names_to = 'Condition',
                 values_to = 'delta_surprisal')

  saveRDS(hi.gpt.pop.ext, '../temp_data/post_samples_hi_gpt_ext.rds')
}



if (!file.exists("'../temp_data/post_samples_eu_lstm_orig.rds'")){

  draws.eu.lstm <- model.basque.lstm.original %>%
    spread_draws(r_sent_id[sent_id,term], b_Intercept, b_Semantic.RoleErg,
                 b_AmbiguityUnamb, `b_Semantic.RoleErg:AmbiguityUnamb`)

  # calculate estimates of each condition level
  draws.eu.lstm <- draws.eu.lstm  %>%
    mutate(AmbAbs = b_Intercept,
           AmbErg = b_Intercept + b_Semantic.RoleErg,
           UnambAbs = b_Intercept + b_AmbiguityUnamb,
           UnambErg = b_Intercept +  b_Semantic.RoleErg +
             b_AmbiguityUnamb + `b_Semantic.RoleErg:AmbiguityUnamb`) %>%
    select(!starts_with('b')) %>%
    pivot_wider(names_from = c('sent_id', 'term'), values_from = r_sent_id) %>%
    select(!matches('.iteration|.chain'))

  draws.eu.lstm <- draws.eu.lstm %>%
    pivot_longer(!.draw, values_to = 'surprisal', names_to = 'Condition') %>%
    mutate(Condition = ifelse(!grepl('_', Condition) ,
                              paste0('Population _', Condition ), Condition)) %>%
    separate(Condition, c('Level', 'Condition'), sep='_')
```

```
  draws.eu.lstm.pop <- draws.eu.lstm %>% filter(Level %in% 'Population ')
  draws.eu.lstm.randomlevel <- draws.eu.lstm %>%
    filter(!Level %in% 'Population ')

  draws.eu.lstm.pop <- draws.eu.lstm.pop %>%
    pivot_wider(names_from = Condition, values_from = surprisal)

  eu.lstm.pop.diff <- draws.eu.lstm.pop %>%
    mutate(diff_Amb = AmbAbs - AmbErg) %>%
    mutate(diff_Unamb = UnambAbs - UnambErg) %>%
    select(.draw, Level, diff_Amb, diff_Unamb)

  #AgentDAT, AgentACC, PatientDAT, PatientACC

  draws.eu.lstm.randomlevel <- draws.eu.lstm.randomlevel %>%
    group_by(.draw, Level) %>%
    mutate('AmbErg' = surprisal[Condition == 'Intercept'] +
             surprisal[Condition=='Semantic.RoleErg']) %>%
    mutate('UnambAbs' = surprisal[Condition == 'AmbiguityUnamb'] +
             surprisal[Condition == 'Intercept'] ) %>%
    mutate('AmbAbs' = surprisal[Condition == 'Intercept']) %>%
    mutate('UnambErg'= surprisal[Condition == 'AmbiguityUnamb'] +
             surprisal[Condition == 'Semantic.RoleErg']) %>%
    select(-Condition, -surprisal) %>%
    distinct()



  eu.lstm.randomlevel.diff <- draws.eu.lstm.randomlevel %>%
    mutate(diff_Amb = AmbAbs - AmbErg) %>%
    mutate(diff_Unamb = UnambAbs - UnambErg) %>%
    select(.draw, Level, diff_Amb, diff_Unamb)


  draws.eu.lstm.all <- rbind(eu.lstm.pop.diff, eu.lstm.randomlevel.diff)

  draws.eu.lstm.all.long <- draws.eu.lstm.all %>%
    select(diff_Amb, diff_Unamb, Level, .draw) %>%
    pivot_longer(!c('Level', '.draw'), names_to = 'Condition',
                 values_to = 'delta_surprisal')



  saveRDS(draws.eu.lstm.all.long,
          '../temp_data/post_samples_eu_lstm_orig.rds')
}

if (!file.exists('../temp_data/post_samples_eu_lstm_ext.rds')){
  draws.eu.lstm.extended <- model.basque.lstm.extended %>%
    spread_draws(r_sent_id[sent_id,term], b_Intercept,
                 b_Semantic.RoleErg, b_AmbiguityUnamb,
                 `b_Semantic.RoleErg:AmbiguityUnamb`)
```

```r
  # calculate estimates of each condition level
  draws.eu.lstm.extended  <- draws.eu.lstm.extended   %>%
    mutate(AmbAbs = b_Intercept, AmbErg = b_Intercept + b_Semantic.RoleErg,
           UnambAbs = b_Intercept + b_AmbiguityUnamb,
           UnambErg = b_Intercept +  b_Semantic.RoleErg + b_AmbiguityUnamb +
             `b_Semantic.RoleErg:AmbiguityUnamb`) %>%
    select(!starts_with('b')) %>%
    pivot_wider(names_from = c('sent_id', 'term'), values_from = r_sent_id) %>%
    select(!matches('.iteration|.chain'))

  draws.eu.lstm.extended  <- draws.eu.lstm.extended  %>%
    pivot_longer(!.draw, values_to = 'surprisal', names_to = 'Condition') %>%
    mutate(Condition = ifelse(!grepl('_', Condition) ,
                              paste0('Population _', Condition ), Condition)) %>%
    separate(Condition, c('Level', 'Condition'), sep='_')

  draws.eu.lstm.extended.pop <- draws.eu.lstm.extended %>%
    filter(Level %in% 'Population ')

  draws.eu.lstm.extended.pop <- draws.eu.lstm.extended.pop %>%
    pivot_wider(names_from = Condition, values_from = surprisal)

  draws.eu.lstm.extended.pop.diff <- draws.eu.lstm.extended.pop %>%
    mutate(diff_Amb = AmbAbs - AmbErg) %>%
    mutate(diff_Unamb = UnambAbs - UnambErg) %>%
    select(.draw, Level, diff_Amb, diff_Unamb)

  draws.eu.lstm.extended.long <- draws.eu.lstm.extended.pop.diff %>%
    select(diff_Amb, diff_Unamb, Level, .draw) %>%
    pivot_longer(!c('Level', '.draw'),
                 names_to = 'Condition', values_to = 'delta_surprisal')
  saveRDS(draws.eu.lstm.extended.long, '../temp_data/post_samples_eu_lstm_ext.rds')
}
if (!file.exists('../temp_data/post_samples_eu_tr_orig.rds')){
  draws.eu.tr <- model.basque.tr.original %>%
    spread_draws(r_sent_id[sent_id,term], b_Intercept,
                 b_Semantic.RoleErg, b_AmbiguityUnamb,
                 `b_Semantic.RoleErg:AmbiguityUnamb`)

  # calculate estimates of each condition level
  draws.eu.tr <- draws.eu.tr  %>%
    mutate(AmbAbs = b_Intercept,
           AmbErg = b_Intercept + b_Semantic.RoleErg,
           UnambAbs = b_Intercept + b_AmbiguityUnamb,
           UnambErg = b_Intercept +  b_Semantic.RoleErg +
             b_AmbiguityUnamb + `b_Semantic.RoleErg:AmbiguityUnamb`) %>%
    select(!starts_with('b')) %>%
    pivot_wider(names_from = c('sent_id', 'term'), values_from = r_sent_id) %>%
    select(!matches('.iteration|.chain'))

  draws.eu.tr <- draws.eu.tr %>%
    pivot_longer(!.draw, values_to = 'surprisal', names_to = 'Condition') %>%
    mutate(Condition = ifelse(!grepl('_', Condition) ,
```

```
                              paste0('Population _', Condition ), Condition)) %>%
    separate(Condition, c('Level', 'Condition'), sep='_')

  draws.eu.tr.pop <- draws.eu.tr %>% filter(Level %in% 'Population ')
  draws.eu.tr.randomlevel <- draws.eu.tr %>% filter(!Level %in% 'Population ')

  draws.eu.tr.pop <- draws.eu.tr.pop %>%
    pivot_wider(names_from = Condition, values_from = surprisal)

  eu.tr.pop.diff <- draws.eu.tr.pop %>%
    mutate(diff_Amb = AmbAbs - AmbErg) %>%
    mutate(diff_Unamb = UnambAbs - UnambErg) %>%
    select(.draw, Level, diff_Amb, diff_Unamb)

  #AgentDAT, AgentACC, PatientDAT, PatientACC

  draws.eu.tr.randomlevel <- draws.eu.tr.randomlevel %>%
    group_by(.draw, Level) %>%
    mutate('AmbErg' = surprisal[Condition == 'Intercept'] +
             surprisal[Condition=='Semantic.RoleErg']) %>%
    mutate('UnambAbs' = surprisal[Condition == 'AmbiguityUnamb'] +
             surprisal[Condition == 'Intercept'] ) %>%
    mutate('AmbAbs' = surprisal[Condition == 'Intercept']) %>%
    mutate('UnambErg'= surprisal[Condition == 'AmbiguityUnamb'] +
             surprisal[Condition == 'Semantic.RoleErg']) %>%
    select(-Condition, -surprisal) %>%
    distinct()



  eu.tr.randomlevel.diff <- draws.eu.tr.randomlevel %>%
    mutate(diff_Amb = AmbAbs - AmbErg) %>%
    mutate(diff_Unamb = UnambAbs - UnambErg) %>%
    select(.draw, Level, diff_Amb, diff_Unamb)


  draws.eu.tr.all <- rbind(eu.tr.pop.diff, eu.tr.randomlevel.diff)

  draws.eu.tr.all.long <- draws.eu.tr.all %>%
    select(diff_Amb, diff_Unamb, Level, .draw) %>%
    pivot_longer(!c('Level', '.draw'),
                 names_to = 'Condition', values_to = 'delta_surprisal')
  saveRDS(draws.eu.tr.all.long, '../temp_data/post_samples_eu_tr_orig.rds')
}

if (!file.exists('../temp_data/post_samples_eu_tr_ext.rds')){

  draws.eu.tr.ext <- model.basque.tr.extended %>%
    spread_draws(r_sent_id[sent_id,term], b_Intercept, b_Semantic.RoleErg,
                 b_AmbiguityUnamb, `b_Semantic.RoleErg:AmbiguityUnamb`)
  # calculate estimates of each condition level
  draws.eu.tr.ext <- draws.eu.tr.ext  %>%
    mutate(AmbAbs = b_Intercept,
```

```
          AmbErg = b_Intercept + b_Semantic.RoleErg,
          UnambAbs = b_Intercept + b_AmbiguityUnamb,
          UnambErg = b_Intercept +  b_Semantic.RoleErg +
            b_AmbiguityUnamb + `b_Semantic.RoleErg:AmbiguityUnamb`) %>%
    select(!starts_with('b')) %>%
    pivot_wider(names_from = c('sent_id', 'term'), values_from = r_sent_id) %>%
    select(!matches('.iteration|.chain'))

  draws.eu.tr.ext <- draws.eu.tr.ext %>%
    pivot_longer(!.draw, values_to = 'surprisal', names_to = 'Condition') %>%
    mutate(Condition = ifelse(!grepl('_', Condition) ,
                              paste0('Population _', Condition ), Condition)) %>%
    separate(Condition, c('Level', 'Condition'), sep='_')

  draws.eu.tr.ext.pop <- draws.eu.tr.ext %>% filter(Level %in% 'Population ')

  draws.eu.tr.ext.pop <- draws.eu.tr.ext.pop %>%
    pivot_wider(names_from = Condition, values_from = surprisal)

  draws.eu.tr.ext.diff<- draws.eu.tr.ext.pop %>%
    mutate(diff_Amb = AmbAbs - AmbErg) %>%
    mutate(diff_Unamb = UnambAbs - UnambErg) %>%
    select(.draw, Level, diff_Amb, diff_Unamb)

  draws.eu.tr.ext.long <- draws.eu.tr.ext.diff%>%
    select(diff_Amb, diff_Unamb, Level, .draw) %>%
    pivot_longer(!c('Level', '.draw'),
                 names_to = 'Condition', values_to = 'delta_surprisal')

  saveRDS(draws.eu.tr.ext.long, '../temp_data/post_samples_eu_tr_ext.rds')
}

if (!file.exists('../temp_data/post_samples_eu_gpt_orig.rds')){
  draws.eu.gpt <- model.basque.gpt.original %>%
    spread_draws(r_sent_id[sent_id,term], b_Intercept, b_Semantic.RoleErg,
                 b_AmbiguityUnamb, `b_Semantic.RoleErg:AmbiguityUnamb`)

  # calculate estimates of each condition level
  draws.eu.gpt <- draws.eu.gpt  %>%
    mutate(AmbAbs = b_Intercept,
          AmbErg = b_Intercept + b_Semantic.RoleErg,
          UnambAbs = b_Intercept + b_AmbiguityUnamb,
          UnambErg = b_Intercept +  b_Semantic.RoleErg +
            b_AmbiguityUnamb + `b_Semantic.RoleErg:AmbiguityUnamb`) %>%
    select(!starts_with('b')) %>%
    pivot_wider(names_from = c('sent_id', 'term'), values_from = r_sent_id) %>%
    select(!matches('.iteration|.chain'))

  draws.eu.gpt <- draws.eu.gpt %>%
    pivot_longer(!.draw, values_to = 'surprisal', names_to = 'Condition') %>%
    mutate(Condition = ifelse(!grepl('_', Condition) ,
                              paste0('Population _', Condition ), Condition)) %>%
    separate(Condition, c('Level', 'Condition'), sep='_')
```

```
  draws.eu.gpt.pop <- draws.eu.gpt %>% filter(Level %in% 'Population ')
  draws.eu.gpt.randomlevel <- draws.eu.gpt %>% filter(!Level %in% 'Population ')

  draws.eu.gpt.pop <- draws.eu.gpt.pop %>%
    pivot_wider(names_from = Condition, values_from = surprisal)

  eu.gpt.pop.diff <- draws.eu.gpt.pop %>%
    mutate(diff_Amb = AmbAbs - AmbErg) %>%
    mutate(diff_Unamb = UnambAbs - UnambErg) %>%
    select(.draw, Level, diff_Amb, diff_Unamb)

  #AgentDAT, AgentACC, PatientDAT, PatientACC

  draws.eu.gpt.randomlevel <- draws.eu.gpt.randomlevel %>%
    group_by(.draw, Level) %>%
    mutate('AmbErg' = surprisal[Condition == 'Intercept'] +
             surprisal[Condition=='Semantic.RoleErg']) %>%
    mutate('UnambAbs' = surprisal[Condition == 'AmbiguityUnamb'] +
             surprisal[Condition == 'Intercept'] ) %>%
    mutate('AmbAbs' = surprisal[Condition == 'Intercept']) %>%
    mutate('UnambErg'= surprisal[Condition == 'AmbiguityUnamb'] +
             surprisal[Condition == 'Semantic.RoleErg']) %>%
    select(-Condition, -surprisal) %>%
    distinct()



  eu.gpt.randomlevel.diff <- draws.eu.gpt.randomlevel %>%
    mutate(diff_Amb = AmbAbs - AmbErg) %>%
    mutate(diff_Unamb = UnambAbs - UnambErg) %>%
    select(.draw, Level, diff_Amb, diff_Unamb)


  draws.eu.gpt.all <- rbind(eu.gpt.pop.diff, eu.gpt.randomlevel.diff)

  draws.eu.gpt.all.long <- draws.eu.gpt.all %>%
    select(diff_Amb, diff_Unamb, Level, .draw) %>%
    pivot_longer(!c('Level', '.draw'),
                 names_to = 'Condition', values_to = 'delta_surprisal')
  saveRDS(draws.eu.gpt.all.long, '../temp_data/post_samples_eu_gpt_orig.rds')
}

if (!file.exists('../temp_data/post_samples_eu_gpt_ext.rds')){

  draws.eu.gpt.ext <- model.basque.gpt.extended %>%
    spread_draws(r_sent_id[sent_id,term], b_Intercept, b_Semantic.RoleErg,
                 b_AmbiguityUnamb, `b_Semantic.RoleErg:AmbiguityUnamb`)
  # calculate estimates of each condition level
  draws.eu.gpt.ext <- draws.eu.gpt.ext  %>%
    mutate(AmbAbs = b_Intercept,
           AmbErg = b_Intercept + b_Semantic.RoleErg,
           UnambAbs = b_Intercept + b_AmbiguityUnamb,
           UnambErg = b_Intercept +  b_Semantic.RoleErg +
```

```
            b_AmbiguityUnamb + `b_Semantic.RoleErg:AmbiguityUnamb`) %>%
    select(!starts_with('b')) %>%
    pivot_wider(names_from = c('sent_id', 'term'), values_from = r_sent_id) %>%
    select(!matches('.iteration|.chain'))

  draws.eu.gpt.ext <- draws.eu.gpt.ext %>%
    pivot_longer(!.draw, values_to = 'surprisal', names_to = 'Condition') %>%
    mutate(Condition = ifelse(!grepl('_', Condition) ,
                              paste0('Population _', Condition ), Condition)) %>%
    separate(Condition, c('Level', 'Condition'), sep='_')

  draws.eu.gpt.ext.pop <- draws.eu.gpt.ext %>% filter(Level %in% 'Population ')

  draws.eu.gpt.ext.pop <- draws.eu.gpt.ext.pop %>%
    pivot_wider(names_from = Condition, values_from = surprisal)

  draws.eu.gpt.ext.diff<- draws.eu.gpt.ext.pop %>%
    mutate(diff_Amb = AmbAbs - AmbErg) %>%
    mutate(diff_Unamb = UnambAbs - UnambErg) %>%
    select(.draw, Level, diff_Amb, diff_Unamb)

  draws.eu.gpt.ext.long <- draws.eu.gpt.ext.diff %>%
    select(diff_Amb, diff_Unamb, Level, .draw) %>%
    pivot_longer(!c('Level', '.draw'), names_to = 'Condition',
                 values_to = 'delta_surprisal')

  saveRDS(draws.eu.gpt.ext.long , '../temp_data/post_samples_eu_gpt_ext.rds')
}
```

```
cis.orig.de <- rbind(draws.de.lstm.orig %>%
                       filter(Level%in% 'Population ') %>%
                       filter(Condition %in% 'diff_ACC') %>%
                       bayestestR::ci(ci=0.89, method='HDI') %>%
                       as.data.frame() %>% mutate(Model = 'LSTM') %>%
                       mutate(Condition = 'Accusative') %>%
                       filter(!Parameter %in% '.draw'),
                     draws.de.lstm.orig %>%
                       filter(Level%in% 'Population ') %>%
                       filter(Condition %in% 'diff_DAT') %>%
                       bayestestR::ci(ci=0.89, method='HDI') %>%
                       as.data.frame() %>% mutate(Model = 'LSTM') %>%
                       mutate(Condition = 'Dative') %>%
                       filter(!Parameter %in% '.draw'),
                     draws.de.tr.orig %>%
                       filter(Level%in% 'Population ') %>%
                       filter(Condition %in% 'diff_ACC') %>%
                       bayestestR::ci(ci=0.89, method='HDI') %>%
                       as.data.frame() %>% mutate(Model = 'BERT') %>%
                       mutate(Condition = 'Accusative') %>%
                       filter(!Parameter %in% '.draw'),
                     draws.de.tr.orig %>%
                       filter(Level%in% 'Population ') %>%
                       filter(Condition %in% 'diff_DAT') %>%
```

```
                    bayestestR::ci(ci=0.89, method='HDI') %>%
                    as.data.frame() %>% mutate(Model = 'BERT') %>%
                    mutate(Condition = 'Dative') %>%
                    filter(!Parameter %in% '.draw' ),
                  draws.de.gpt.orig %>%
                    filter(Level%in% 'Population ') %>%
                    filter(Condition %in% 'diff_ACC') %>%
                    bayestestR::ci(ci=0.89, method='HDI') %>%
                    as.data.frame() %>% mutate(Model = 'GPT-2') %>%
                    mutate(Condition = 'Accusative') %>%
                    filter(!Parameter %in% '.draw' ),
                  draws.de.gpt.orig %>%
                    filter(Level%in% 'Population ') %>%
                    filter(Condition %in% 'diff_DAT') %>%
                    bayestestR::ci(ci=0.89, method='HDI') %>%
                    as.data.frame() %>% mutate(Model = 'GPT-2') %>%
                    mutate(Condition = 'Dative') %>%
                    filter(!Parameter %in% '.draw' )
) %>%
  select(CI_low, CI_high, Condition, Model)


means.orig.de <- rbind(
  draws.de.lstm.orig %>%
    filter(Level%in% 'Population ') %>%
    filter(Condition %in% 'diff_ACC') %>%
    summarise(Mean = mean(delta_surprisal)),
  draws.de.lstm.orig %>%
    filter(Level%in% 'Population ') %>%
    filter(Condition %in% 'diff_DAT') %>%
    summarise(Mean = mean(delta_surprisal)),
  draws.de.tr.orig %>%
    filter(Level%in% 'Population ') %>%
    filter(Condition %in% 'diff_ACC') %>%
    summarise(Mean = mean(delta_surprisal)),
  draws.de.tr.orig %>%
    filter(Level%in% 'Population ') %>%
    filter(Condition %in% 'diff_DAT') %>%
    summarise(Mean = mean(delta_surprisal)),
  draws.de.gpt.orig %>%
    filter(Level%in% 'Population ') %>%
    filter(Condition %in% 'diff_ACC') %>%
    summarise(Mean = mean(delta_surprisal)),
  draws.de.gpt.orig %>%
    filter(Level%in% 'Population ') %>%
    filter(Condition %in% 'diff_DAT') %>%
    summarise(Mean = mean(delta_surprisal)))

means.ci.orig.de <- cbind(cis.orig.de, means.orig.de)


col.order <- c('Model', 'Condition', 'Mean', 'CI_low', 'CI_high')
```

```r
means.ci.orig.re.de <- means.ci.orig.de[,col.order]
```

Table S4: German: Means and 89% credibility intervals for original dataset

Model	Condition	Mean	CI_low	CI_high
LSTM	Accusative	0.3	-0.39	1.1
LSTM	Dative	0.7	-0.01	1.4
BERT	Accusative	2.2	1.84	2.7
BERT	Dative	2.3	1.86	2.7
GPT-2	Accusative	1.5	1.16	1.9
GPT-2	Dative	1.5	1.10	1.9

Extended Stimuli

Table S5: German: Means and 89% credibility intervals for extended dataset

Model	Condition	Mean	CI_low	CI_high
LSTM	Accusative	0.2	-0.24	0.7
LSTM	Dative	0.2	-0.25	0.7
BERT	Accusative	2.0	1.57	2.4
BERT	Dative	0.9	0.45	1.3
GPT-2	Accusative	2.1	1.70	2.5
GPT-2	Dative	1.3	0.91	1.7

Table S6: Hindi: Means and 89% credibility intervals for original dataset

Model	Condition	Mean	CI_low	CI_high
LSTM	imperfective	0.3	-0.11	0.7
LSTM	perfective	0.1	-0.31	0.5
BERT	imperfective	0.7	0.05	1.3
BERT	perfective	0.6	-0.03	1.2
GPT-2	imperfective	0.5	0.34	0.6
GPT-2	perfective	0.8	0.63	1.0

Table S7: Hindi: Means and 89% credibility intervals for extended dataset

Model	Condition	Mean	CI_low	CI_high
LSTM	imperfective	0.3	-0.21	0.7
BERT	perfective	0.3	-0.19	0.8
LSTM	imperfective	0.8	0.16	1.4
LSTM	BERT	0.9	0.24	1.6
GPT-2	imperfective	0.5	0.34	0.7
GPT-2	perfective	0.8	0.63	1.0

Table S8: Basque: Means and 89 percent credibility intervals for original dataset

Model	Condition	Mean	CI_low	CI_high
LSTM	ambiguous	0.4	-0.02	0.7
LSTM	unambiguous	0.2	-0.22	0.5
RoBERTa	ambiguous	0.0	-0.53	0.5
RoBERTa	unambiguous	2.0	1.46	2.5
GPT-2	ambiguous	1.2	0.73	1.7
GPT-2	unambiguous	1.1	0.62	1.6

Table S9: Basque: Means and 89 percent credibility intervals for extended dataset

Model	Condition	Mean	CI_low	CI_high
LSTM	Amb_A	0.5	0.23	0.7
LSTM	Amb_P	0.3	0.04	0.5
RoBERTa	Amb_A	0.0	-0.53	0.5
LSTM	Amb_P	2.0	1.46	2.5
GPT-2	Amb_A	0.8	0.27	1.4
GPT-2	Amb_P	0.7	0.13	1.3

S3.4 Plots

```
plot.de.lstm.ext <- draws.de.lstm.ext %>%
  filter(Level %in% 'Population ') %>%
  mutate(Condition =
           ifelse(Condition %in% 'diff_ACC', 'accusative', 'dative')) %>%
  ggplot(aes(x = delta_surprisal, y =
               Condition,color=Condition, fill= Condition)) +
  stat_slab(alpha = .5) +
  stat_pointinterval(position = position_dodge(width = .2, preserve = "single"),
                     .width = c(.50,.80,.90)) +
  geom_vline(xintercept = 0, linetype="dashed") +
  scale_color_manual(values = c('accusative' = '#143548', 'dative' = '#AAC4D1')) +
  scale_fill_manual(values = c('accusative' = '#143548', 'dative' = '#AAC4D1')) +
  labs(y=NULL, x = element_blank()) +
  guides(fill="none", color="none") +
  theme(axis.text.y = element_blank(),
        #axis.text.x = element_blank(),
        panel.grid = element_blank(),
        panel.border = element_blank(),
        strip.text = element_text(size=10),
        axis.line.x = element_line(size = 0.5), axis.ticks.y = element_blank(),
        plot.background = element_rect(fill = "transparent", colour = NA),
        plot.title = element_text(size=22)) +
  labs(
    title = "German LSTM (extended dataset)"
  )

plot.de.tr.ext <- draws.de.tr.ext %>%
  filter(Level %in% 'Population ') %>%
  mutate(Condition = ifelse(Condition %in% 'diff_ACC', 'accusative', 'dative'))  %>%
  ggplot(aes(x = delta_surprisal, y = Condition,color=Condition, fill= Condition)) +
  stat_slab(alpha = .5) +
  stat_pointinterval(position = position_dodge(width = .2, preserve = "single"),
                     .width = c(.50,.80,.90)) +
  geom_vline(xintercept = 0, linetype="dashed") +
  scale_color_manual(values = c('accusative' = '#143548', 'dative' = '#AAC4D1')) +
  scale_fill_manual(values = c('accusative' = '#143548', 'dative' = '#AAC4D1')) +
  labs(y=NULL, x = element_blank()) +
  guides(fill="none", color="none") +
  theme(axis.text.y = element_blank(),
        #axis.text.x = element_blank(),
        panel.grid = element_blank(),
        panel.border = element_blank(),
        strip.text = element_text(size=10),
        axis.line.x = element_line(size = 0.5), axis.ticks.y = element_blank(),
        plot.background = element_rect(fill = "transparent", colour = NA),
        plot.title = element_text(size=22)) +
  labs(
    title = "German BERT (extended dataset)"
  )

plot.de.gpt.ext <- draws.de.gpt.ext  %>%
```

```r
filter(Level %in% 'Population ') %>%
mutate(Condition = ifelse(Condition %in% 'diff_ACC', 'accusative', 'dative'))  %>%
ggplot(aes(x = delta_surprisal, y = Condition,color=Condition, fill= Condition)) +
stat_slab(alpha = .5) +
stat_pointinterval(position = position_dodge(width = .2, preserve = "single"),
                   .width = c(.50,.80,.90)) +
geom_vline(xintercept = 0, linetype="dashed") +
scale_color_manual(values = c('accusative' = '#143548', 'dative' = '#AAC4D1')) +
scale_fill_manual(values = c('accusative' = '#143548', 'dative' = '#AAC4D1')) +
labs(y=NULL, x = element_blank()) +
theme(axis.text.y = element_blank(),
      #axis.text.x = element_blank(),
      panel.grid = element_blank(),
      panel.border = element_blank(),
      strip.text = element_text(size=10),
      axis.line.x = element_line(size = 0.5), axis.ticks.y = element_blank(),
      plot.background = element_rect(fill = "transparent", colour = NA),
      plot.title = element_text(size=22),
      legend.key.size = unit(1, 'cm'), #change legend key size
      legend.key.height = unit(1, 'cm'), #change legend key height
      legend.key.width = unit(1, 'cm'), #change legend key width
      legend.text = element_text(size=20),
      legend.title = element_text(size=25))+

labs(
  title = "German GPT-2 (extended dataset)"
)
```

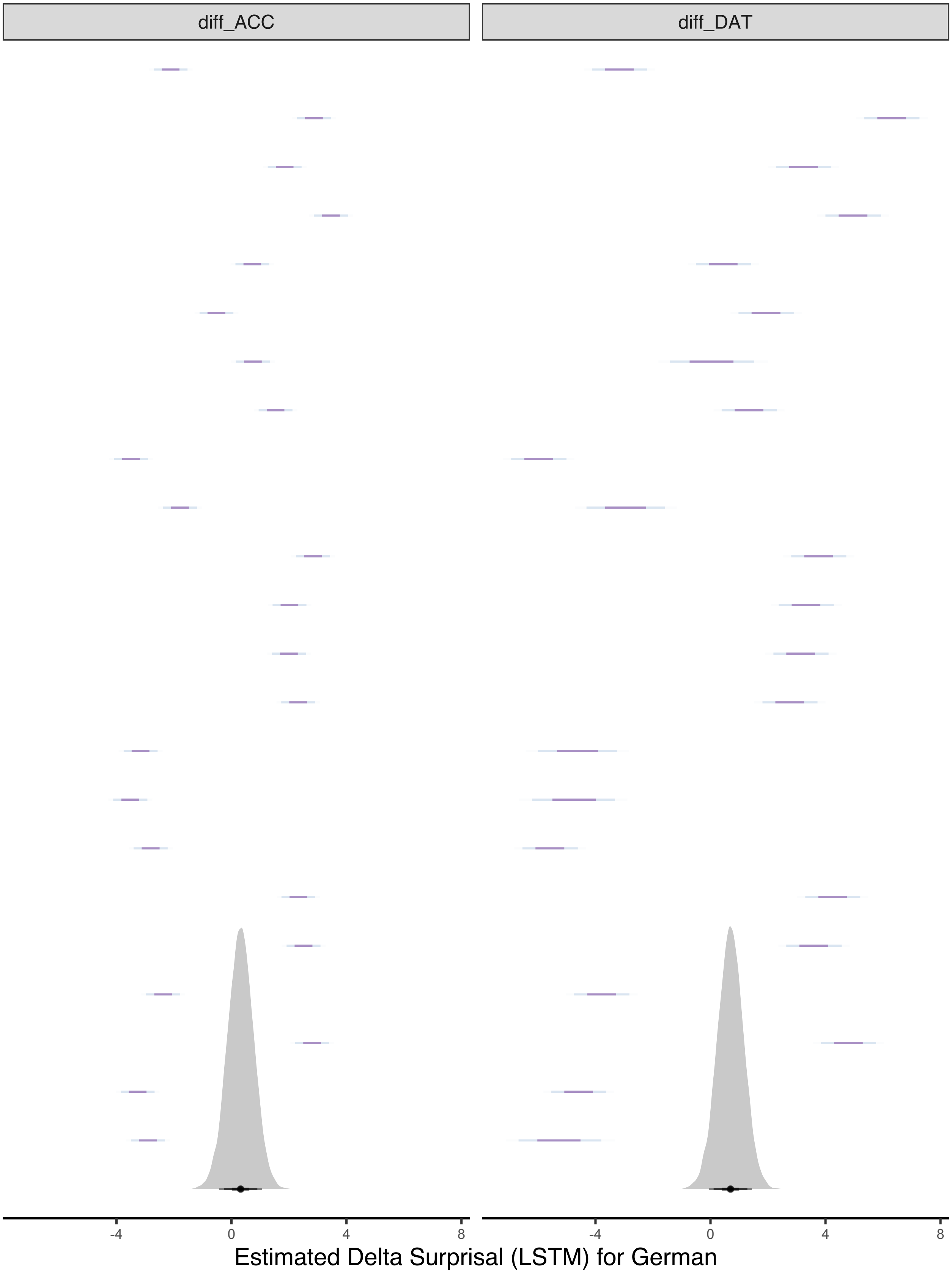

Figure S1: posterior distributions of the estimated surprisal difference between the critical conditions (agent-initial vs. patient-initial sentences), displayed for both conditions (accusative vs. dative). Surprisal is estimated by LSTMs and both population and group level estimates are displayed. LSTM and original dataset

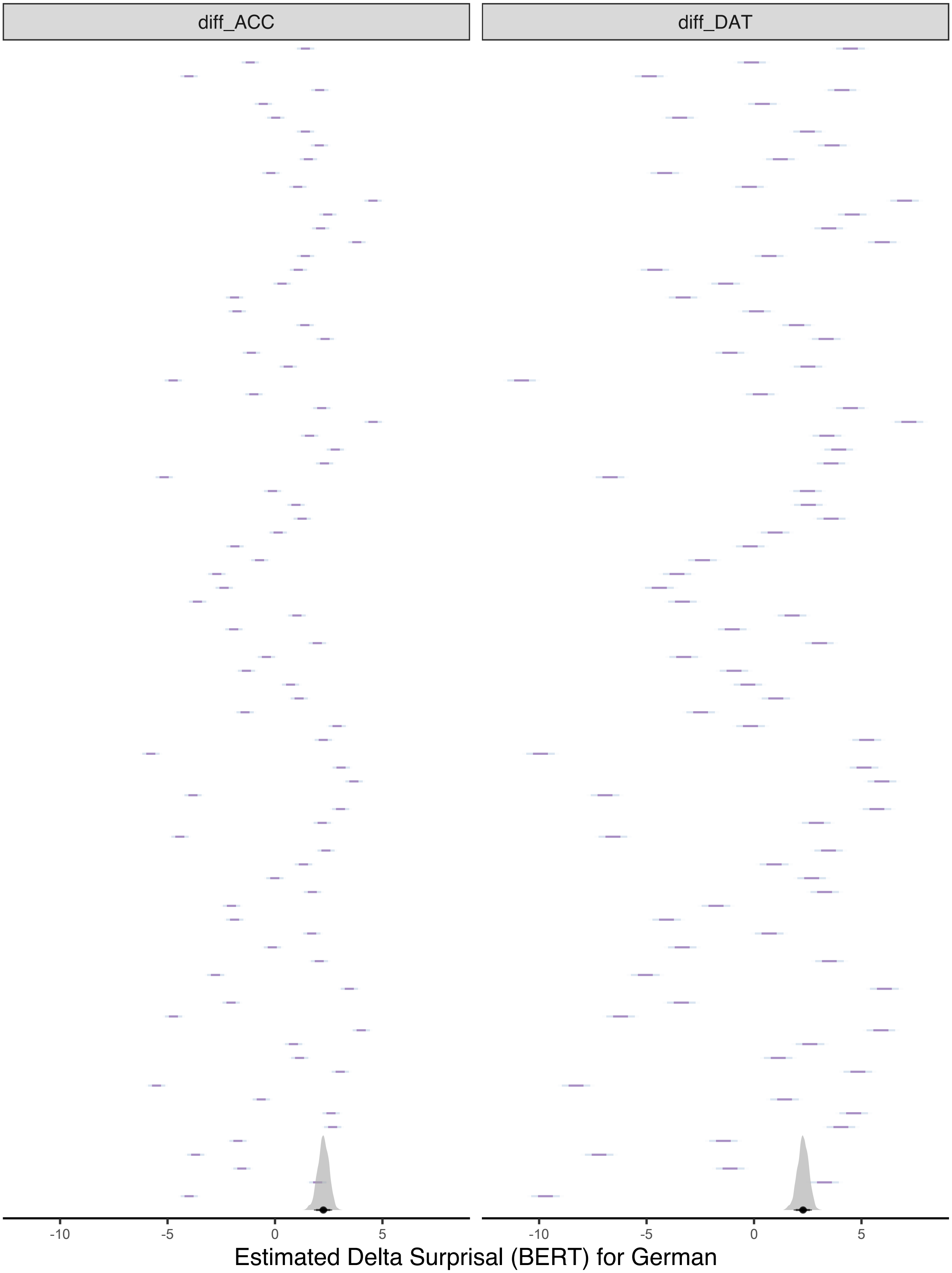

Figure S2: posterior distributions of the estimated surprisal difference between the critical conditions (agent-initial vs. patient-initial sentences), displayed for both conditions (accusative vs. dative). Surprisal is estimated by BERT and both population and group level estimates are displayed. LSTM and original dataset

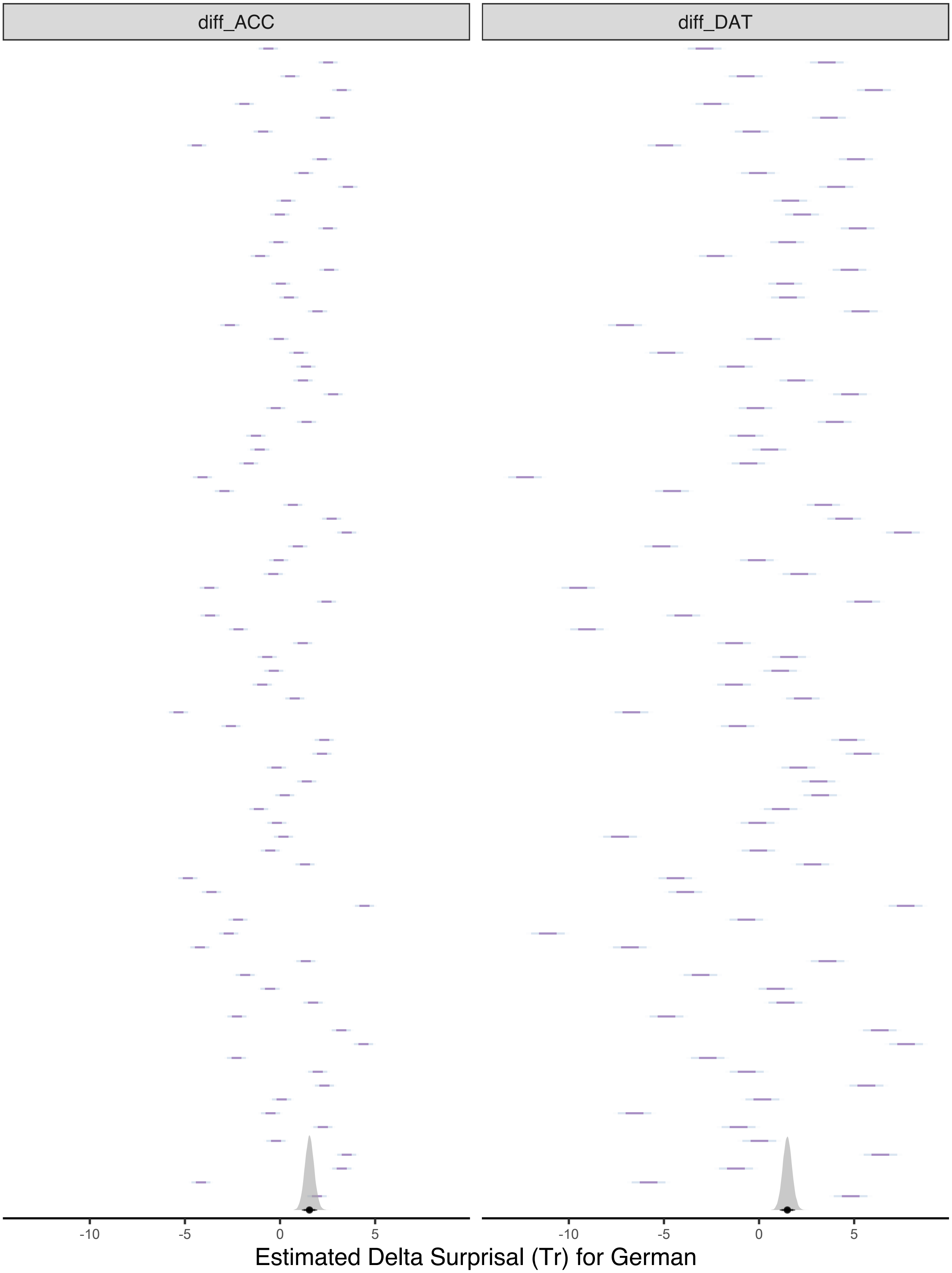

Figure S3: posterior distributions of the estimated surprisal difference between the critical conditions (agent-initial vs. patient-initial sentences), displayed for both conditions (accusative vs. dative). Surprisal is estimated by GPT-2 and both population and group level estimates are displayed. LSTM and original dataset

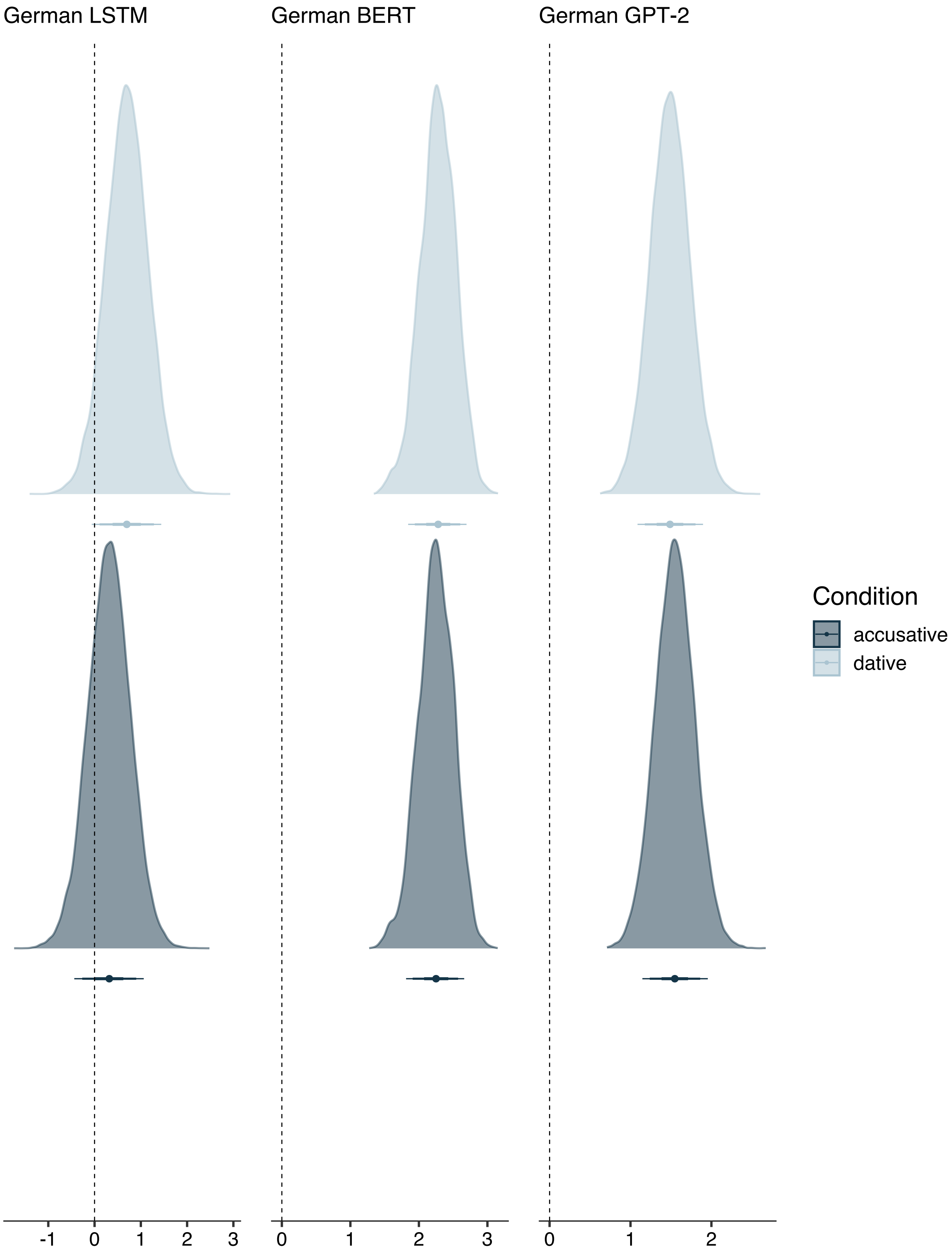

Figure S4: posterior distributions of the estimated surprisal difference between the critical conditions (agent-initial vs. patient-initial sentences), displayed for both conditions (accusative vs. dative). Surprisal is estimated by three types of language models. Only the population level is diplayed.

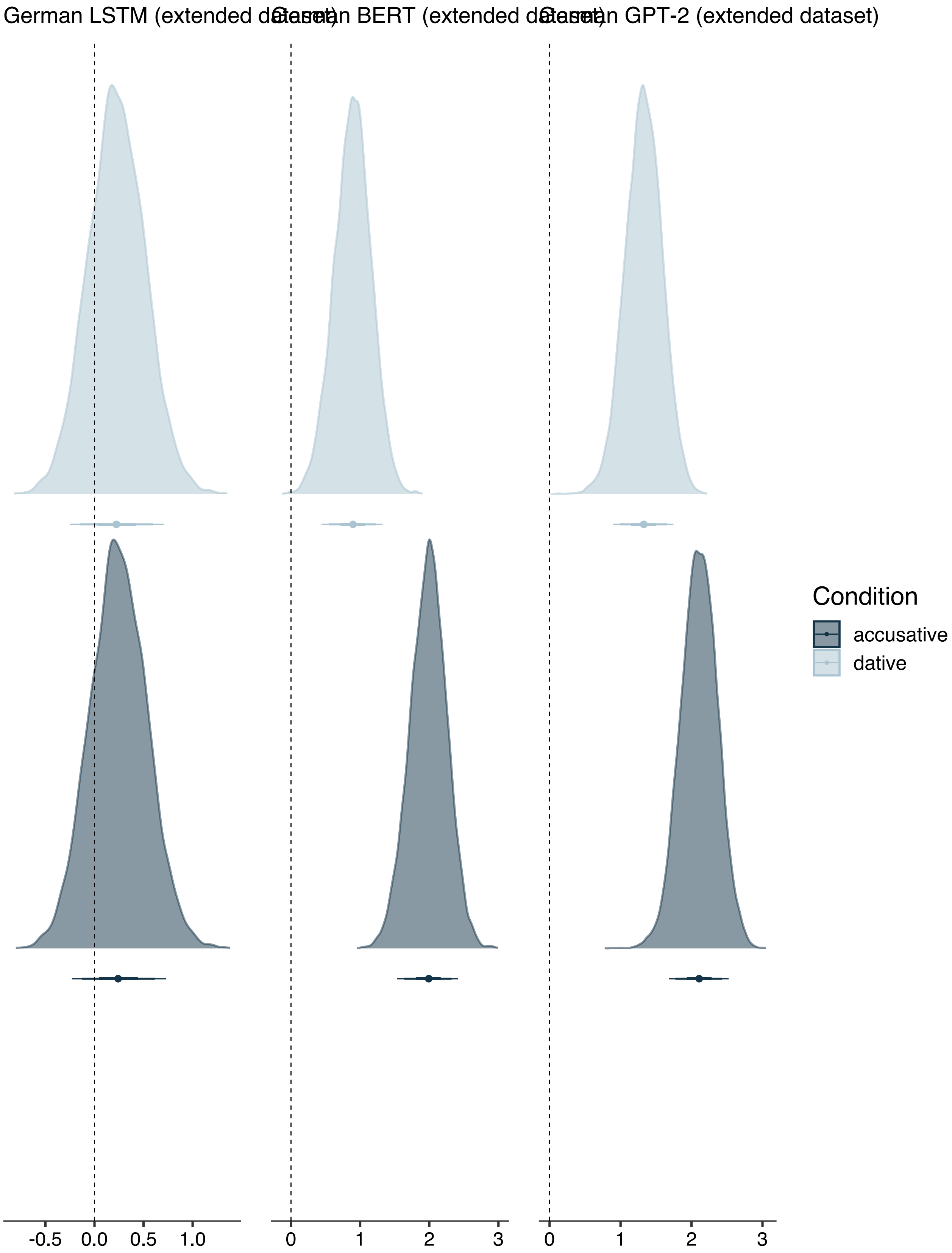

Figure S5: Results for the extended dataset: posterior distributions of the estimated surprisal difference between the critical conditions (agent-initial vs. patient-initial sentences), displayed for both conditions (accusative vs. dative). Surprisal is estimated by three types of language models. Only the population level is diplayed.

```r
plot.hi.lstm.ext <- draws.hi.lstm.ext %>%
  filter(Level %in% 'Population ') %>%
  mutate(Condition = ifelse(Condition %in%
                              'diff_Ipfv', 'imperfective', 'perfective'))  %>%
  ggplot(aes(x = delta_surprisal, y =
               Condition,color=Condition, fill= Condition)) +
  stat_slab(alpha = .5) +
  stat_pointinterval(position = position_dodge(width = .2, preserve = "single"),
                     .width = c(.50,.80,.90)) +
  geom_vline(xintercept = 0, linetype="dashed") +
  scale_color_manual(values =
                       c('imperfective' = '#DA3C6C', 'perfective' = '#FFB5AC')) +
  scale_fill_manual(values =
                      c('imperfective' = '#DA3C6C', 'perfective' = '#FFB5AC')) +
  labs(y=NULL, x = element_blank()) +
  guides(fill="none", color="none") +
  theme(axis.text.y = element_blank(),
        #axis.text.x = element_blank(),
        panel.grid = element_blank(),
        plot.title = element_text(size=22),
        panel.border = element_blank(),
        strip.text = element_text(size=10),
        axis.line.x = element_line(size = 0.5), axis.ticks.y = element_blank(),
        plot.background = element_rect(fill = "transparent", colour = NA)) +
  labs(
    title = "Hindi LSTM (extended dataset)"
  )

plot.hi.bert.ext <- draws.hi.bert.ext  %>%
  filter(Level %in% 'Population ') %>%
  mutate(Condition = ifelse(Condition %in%
                              'diff_Ipfv', 'imperfective', 'perfective'))  %>%
  ggplot(aes(x = delta_surprisal, y = Condition,color=Condition, fill= Condition)) +
  stat_slab(alpha = .5) +
  stat_pointinterval(position = position_dodge(width = .2, preserve = "single"),
                     .width = c(.50,.80,.90)) +
  geom_vline(xintercept = 0, linetype="dashed") +
  scale_color_manual(values =
                       c('imperfective' = '#DA3C6C', 'perfective' = '#FFB5AC')) +
  scale_fill_manual(values =
                      c('imperfective' = '#DA3C6C', 'perfective' = '#FFB5AC')) +
  labs(y=NULL, x = element_blank()) +
  guides(fill="none", color="none") +
  theme(axis.text.y = element_blank(),
        #axis.text.x = element_blank(),
        panel.grid = element_blank(),
        panel.border = element_blank(),
        plot.title = element_text(size=22),
        strip.text = element_text(size=10),
        axis.line.x = element_line(size = 0.5), axis.ticks.y = element_blank(),
        plot.background = element_rect(fill = "transparent", colour = NA)) +
  labs(
    title = "Hindi BERT (extended dataset)"
```

```r
  )

plot.hi.gpt.ext <- draws.hi.gpt.ext %>%
  filter(Level %in% 'Population ') %>%
  mutate(Condition = ifelse(Condition %in%
                              'diff_Ipfv', 'imperfective', 'perfective'))  %>%
  ggplot(aes(x = delta_surprisal,
             y = Condition,color=Condition, fill= Condition)) +
  stat_slab(alpha = .5) +
  stat_pointinterval(position = position_dodge(width = .2, preserve = "single"),
                     .width = c(.50,.80,.90)) +
  geom_vline(xintercept = 0, linetype="dashed") +
  scale_color_manual(values =
                       c('imperfective' = '#DA3C6C', 'perfective' = '#FFB5AC')) +
  scale_fill_manual(values =
                      c('imperfective' = '#DA3C6C', 'perfective' = '#FFB5AC')) +
  labs(y=NULL, x = element_blank()) +
  theme(axis.text.y = element_blank(),
        #axis.text.x = element_blank(),
        panel.grid = element_blank(),
        panel.border = element_blank(),
        strip.text = element_text(size=10),
        axis.line.x = element_line(size = 0.5), axis.ticks.y = element_blank(),
        plot.background = element_rect(fill = "transparent", colour = NA),
        plot.title = element_text(size=22),
        legend.key.size = unit(1, 'cm'), #change legend key size
        legend.key.height = unit(1, 'cm'), #change legend key height
        legend.key.width = unit(1, 'cm'), #change legend key width
        legend.text = element_text(size=20),
        legend.title = element_text(size=25)) +
  labs(
    title = "Hindi GPT-2 (extended dataset)"
  )
```

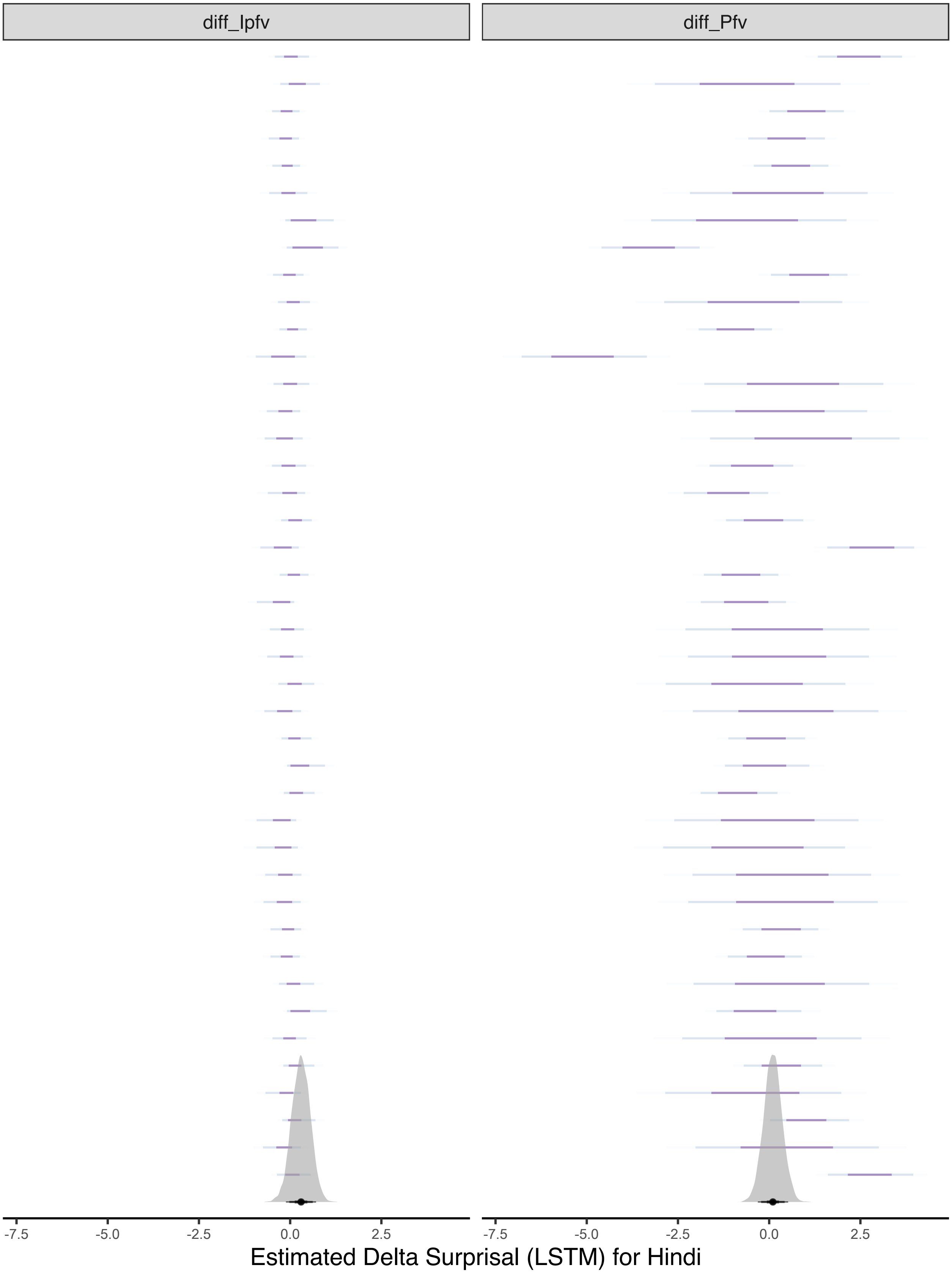

Figure S6: posterior distributions of the estimated surprisal difference between the critical conditions (ambiguous vs. unambiguous sentences), displayed for both conditions (imperfective vs. perfective). Surprisal is estimated by LSTMs and both population and group level estimates are displayed. LSTM and original dataset

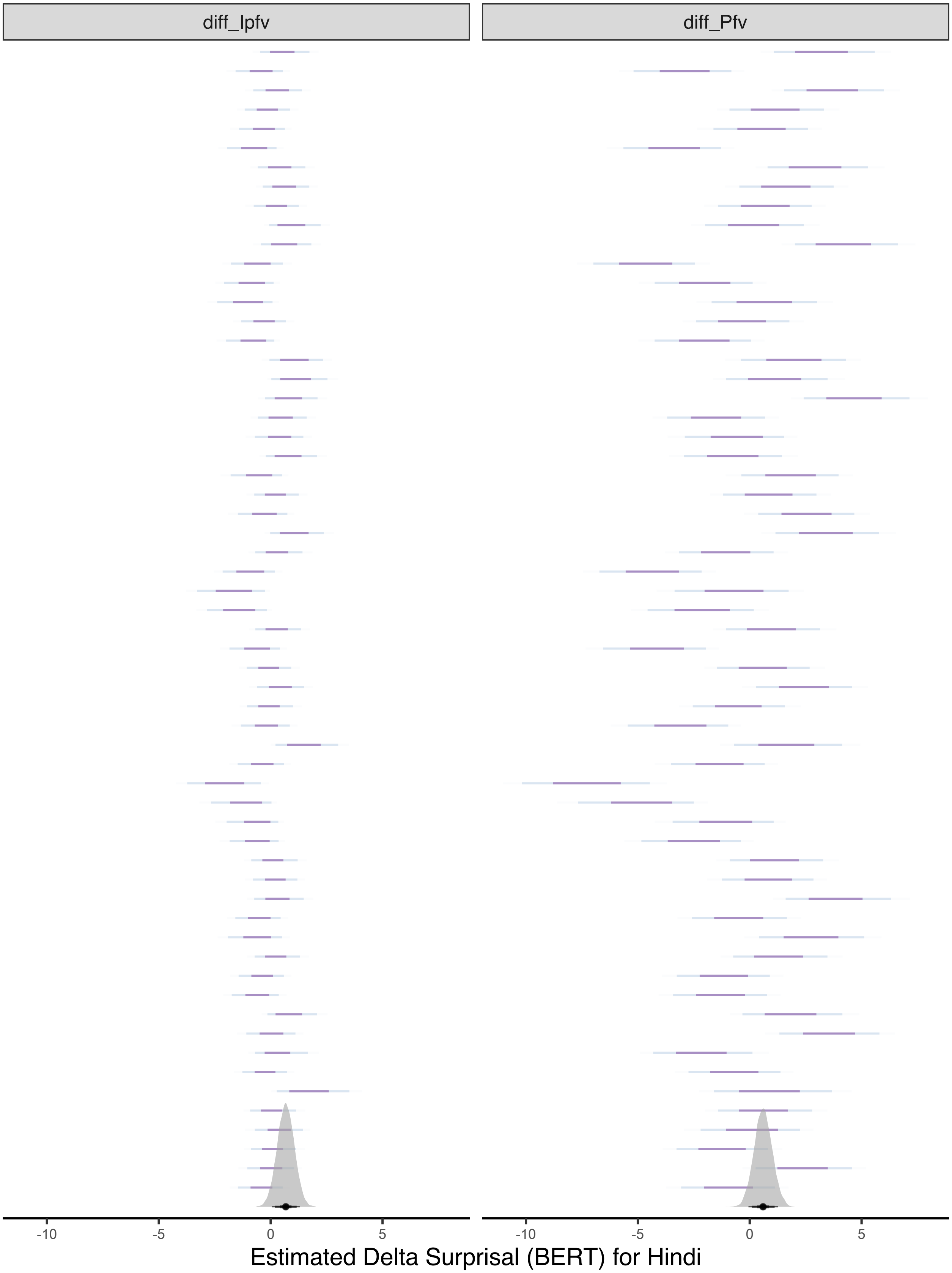

Figure S7: posterior distributions of the estimated surprisal difference between the critical conditions (ambiguous vs. unambiguous sentences), displayed for both conditions (imperfective vs. perfective). Surprisal is estimated by BERT and both population and group level estimates are displayed. LSTM and original dataset

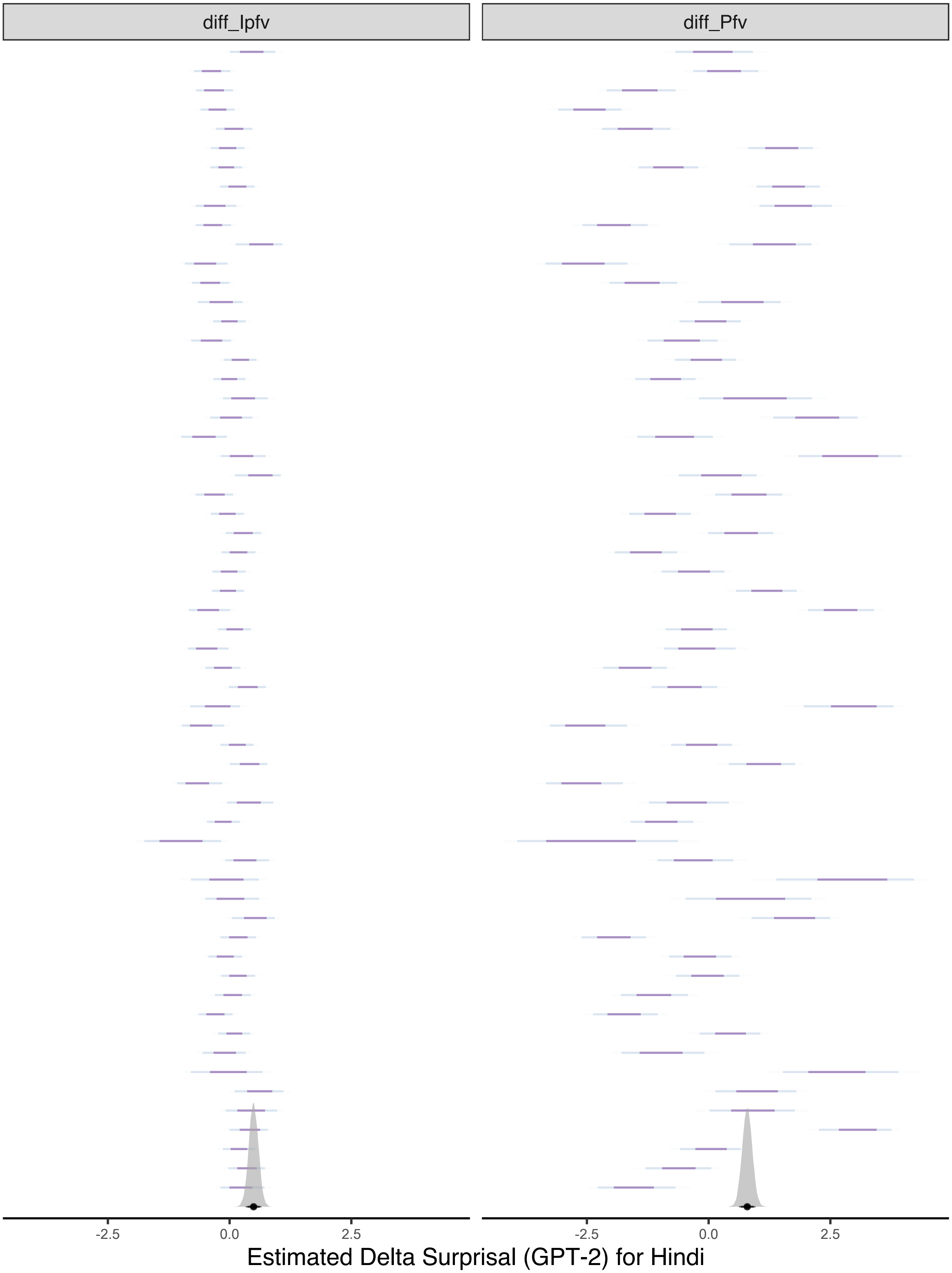

Figure S8: posterior distributions of the estimated surprisal difference between the critical conditions (ambiguous vs. unambiguous sentences), displayed for both conditions (imperfective vs. perfective). Surprisal is estimated by GPT-2 and both population and group level estimates are displayed. LSTM and original dataset

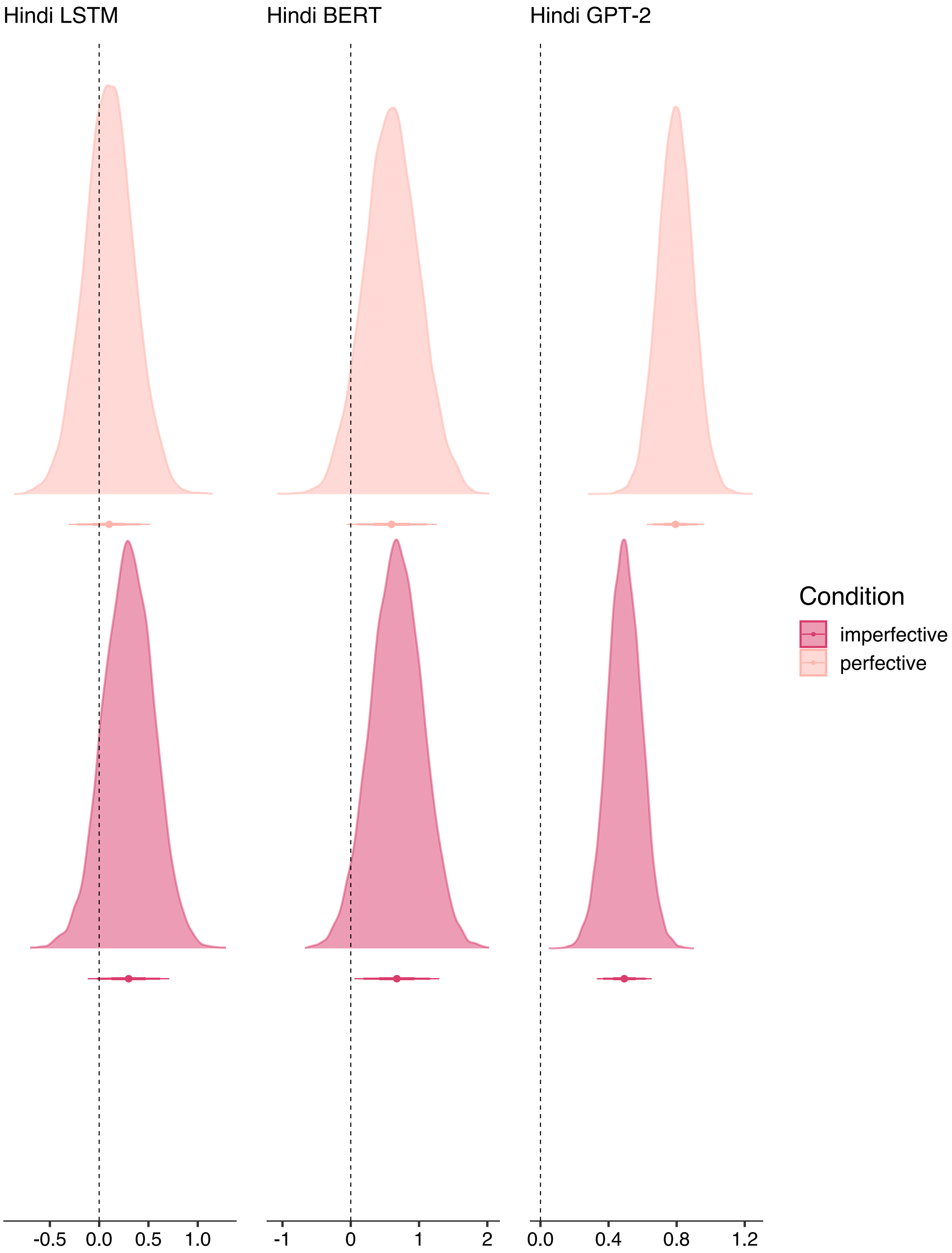

Figure S9: posterior distributions of the estimated surprisal difference between the critical conditions (ambiguous vs. unambiguous sentences), displayed for both conditions (imperfective vs. perfective). Surprisal is estimated by three types of language models. Only the population level is diplayed.

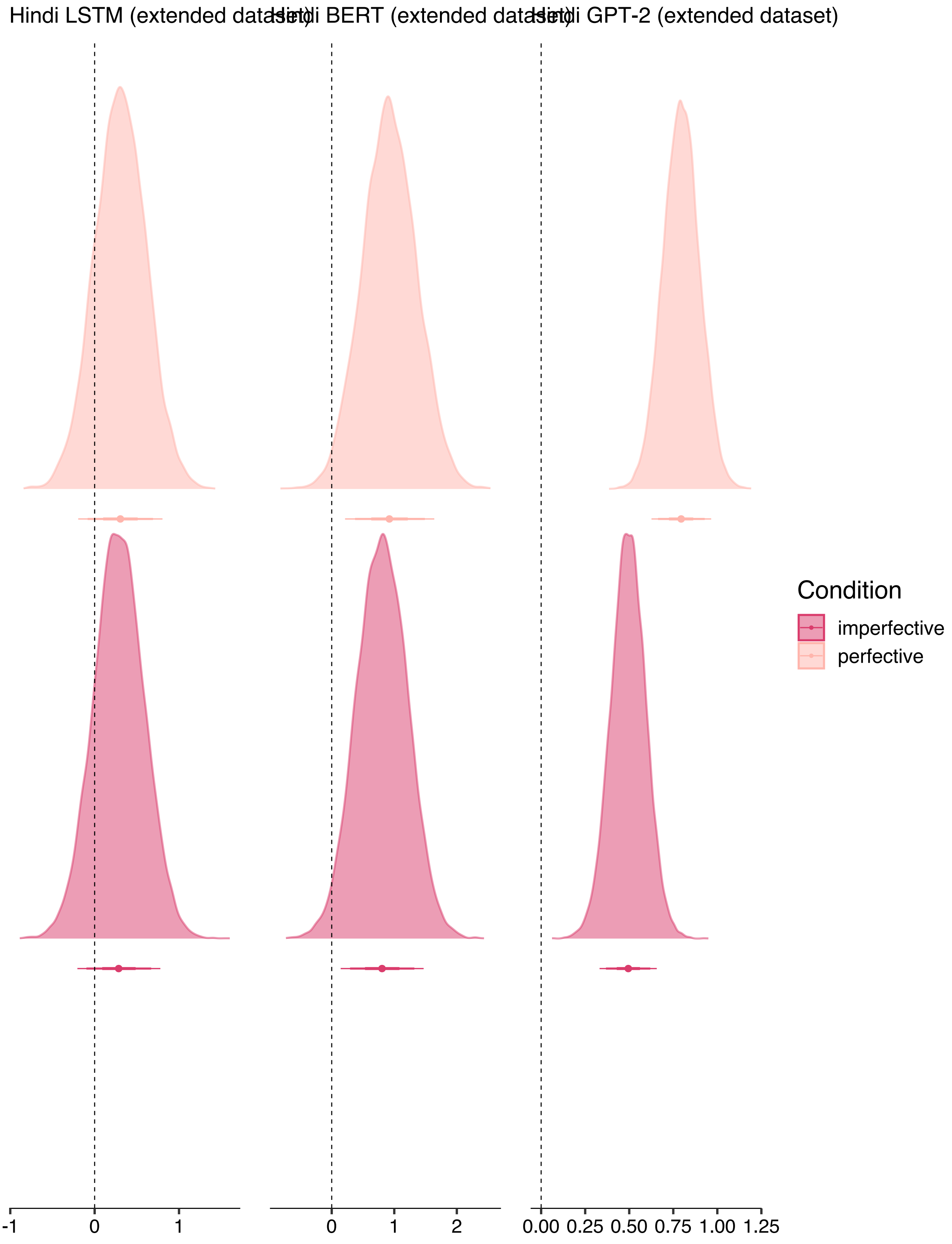

Figure S10: Results for the extended dataset: posterior distributions of the estimated surprisal difference between the critical conditions (ambiguous vs. unambiguous sentences), displayed for both conditions (imperfective vs. perfective). Surprisal is estimated by three types of language models. Only the population level is diplayed.

```r
plot.eu.lstm.ext <- draws.eu.lstm.ext.long %>%
  filter(Level %in% 'Population ') %>%
  mutate(Condition =
           ifelse(Condition %in% 'diff_Amb', 'ambiguous', 'unambiguous'))  %>%
  ggplot(aes(x = delta_surprisal,
             y = Condition,color=Condition, fill= Condition)) +
  stat_slab(alpha = .5) +
  stat_pointinterval(position = position_dodge(width = .2, preserve = "single"),
                     .width = c(.50,.80,.90)) +
  geom_vline(xintercept = 0, linetype="dashed") +
  scale_color_manual(values = c('ambiguous' = '#0B5F47',
                                'unambiguous' = '#9FB39B')) +
  scale_fill_manual(values = c('ambiguous' = '#0B5F47',
                               'unambiguous' = '#9FB39B')) +
  labs(y=NULL) +
  theme(axis.text.y = element_blank(),
        #axis.text.x = element_blank(),
        panel.grid = element_blank(),
        panel.border = element_blank(),
        strip.text = element_text(size=10),
        plot.title = element_text(size=22),
        axis.line.x = element_line(size = 0.5), axis.ticks.y = element_blank(),
        plot.background = element_rect(fill = "transparent", colour = NA)) +
  labs(
    title = "Basque RoBERTa"
  )


plot.eu.tr.ext <- draws.eu.tr.ext.long%>%
  filter(Level %in% 'Population ') %>%
  mutate(Condition =
           ifelse(Condition %in% 'diff_Amb', 'ambiguous', 'unambiguous'))  %>%
  ggplot(aes(x = delta_surprisal, y = Condition,color=Condition, fill= Condition)) +
  stat_slab(alpha = .5) +
  stat_pointinterval(position = position_dodge(width = .2, preserve = "single"),
                     .width = c(.50,.80,.90)) +
  geom_vline(xintercept = 0, linetype="dashed") +
  scale_color_manual(values = c('ambiguous' = '#0B5F47',
                                'unambiguous' = '#9FB39B')) +
  scale_fill_manual(values = c('ambiguous' = '#0B5F47',
                               'unambiguous' = '#9FB39B')) +
  labs(y=NULL) +
  theme(axis.text.y = element_blank(),
        #axis.text.x = element_blank(),
        panel.grid = element_blank(),
        panel.border = element_blank(),
        strip.text = element_text(size=10),
        plot.title = element_text(size=22),
        axis.line.x = element_line(size = 0.5), axis.ticks.y = element_blank(),
        plot.background = element_rect(fill = "transparent", colour = NA)) +
  labs(
    title = "Basque RoBERTa"
  )
```

```
plot.eu.gpt.ext <- draws.eu.gpt.ext.long%>%
  filter(Level %in% 'Population ') %>%
  mutate(Condition =
           ifelse(Condition %in% 'diff_Amb', 'ambiguous', 'unambiguous'))  %>%
  ggplot(aes(x = delta_surprisal,
             y = Condition,color=Condition, fill= Condition)) +
  stat_slab(alpha = .5) +
  stat_pointinterval(position = position_dodge(width = .2, preserve = "single"),
                     .width = c(.50,.80,.90)) +
  geom_vline(xintercept = 0, linetype="dashed") +
  scale_color_manual(values = c('ambiguous' = '#0B5F47',
                               'unambiguous' = '#9FB39B')) +
  scale_fill_manual(values = c('ambiguous' = '#0B5F47',
                              'unambiguous' = '#9FB39B')) +
  labs(y=NULL) +
  theme(axis.text.y = element_blank(),
        #axis.text.x = element_blank(),
        panel.grid = element_blank(),
        panel.border = element_blank(),
        strip.text = element_text(size=10),
        axis.line.x = element_line(size = 0.5), axis.ticks.y = element_blank(),
        plot.background = element_rect(fill = "transparent", colour = NA)) +
  labs(
    title = "Basque RoBERTa"
  )
```

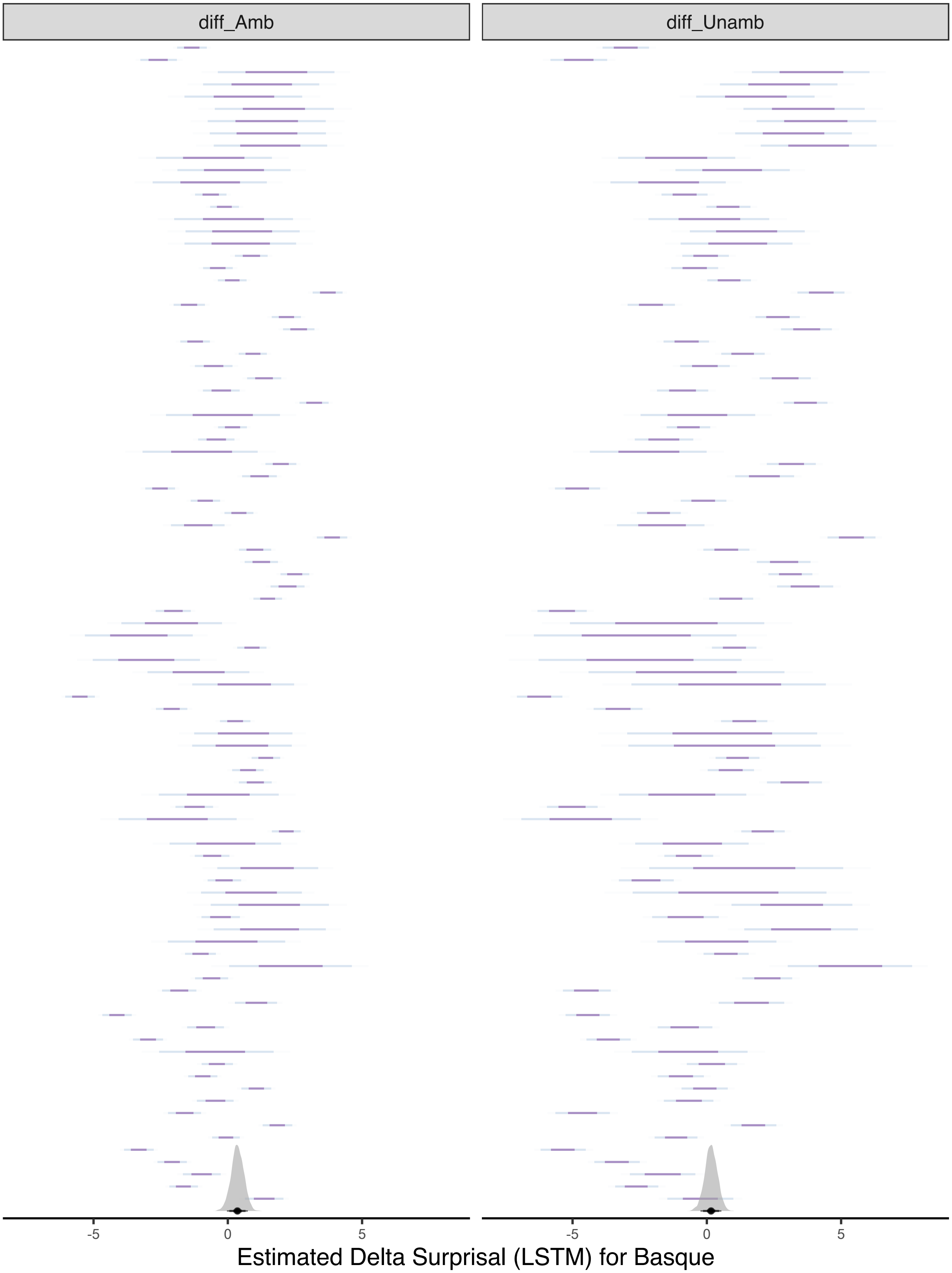

Figure S11: posterior distributions of the estimated surprisal difference between the critical conditions (agentive subject vs. patientive subject), displayed for both conditions (ambiguous vs. unambiguous). Surprisal is estimated by LSTMs and both population and group level estimates are displayed. LSTM and original dataset

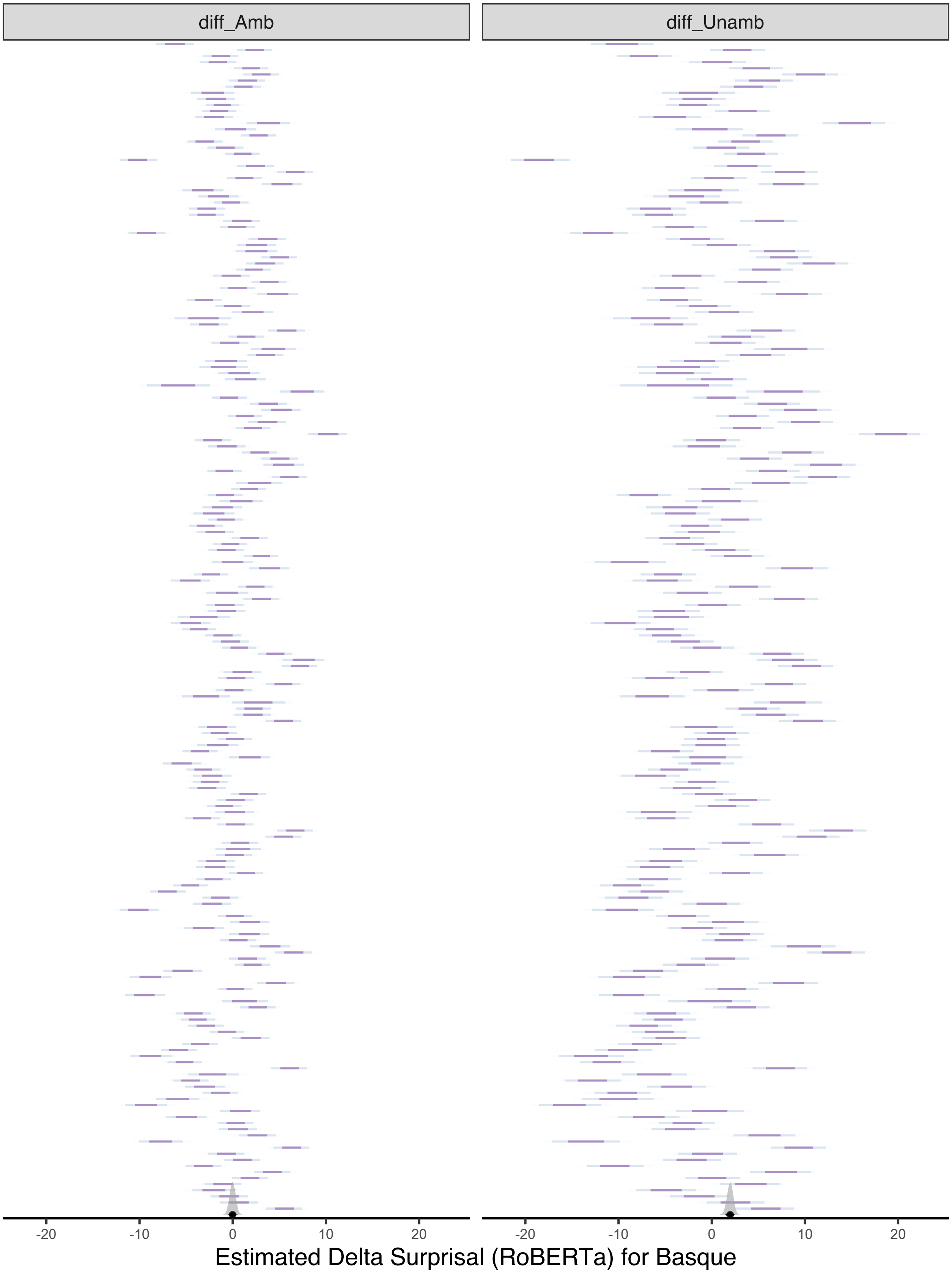

Figure S12: posterior distributions of the estimated surprisal difference between the critical conditions (agentive subject vs. patientive subject), displayed for both conditions (ambiguous vs. unambiguous). Surprisal is estimated by RoBERTa and both population and group level estimates are displayed. LSTM and original dataset

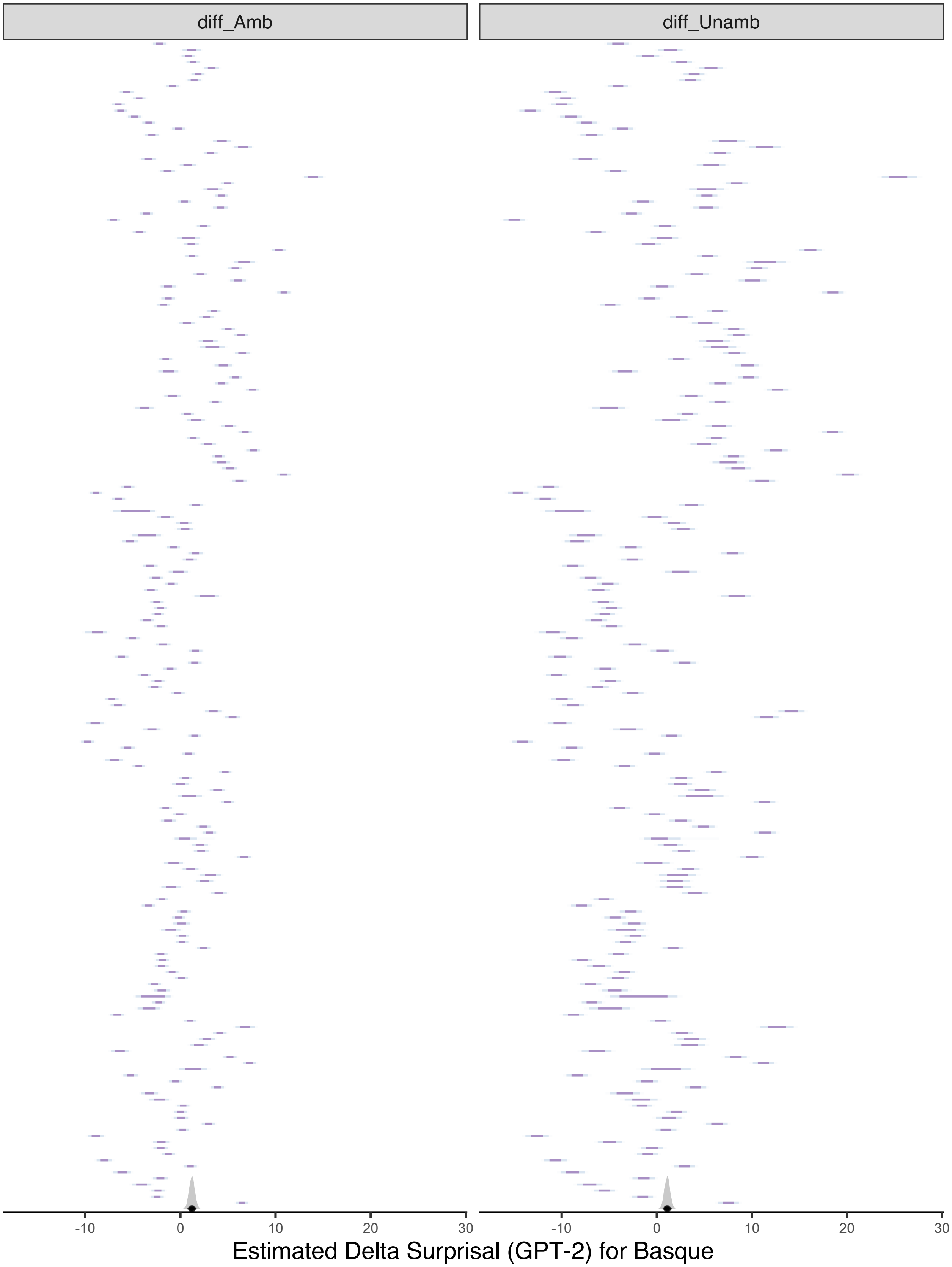

Figure S13: posterior distributions of the estimated surprisal difference between the critical conditions (agentive subject vs. patientive subject), displayed for both conditions (ambiguous vs. unambiguous). Surprisal is estimated by GPT-2 and both population and group level estimates are displayed. LSTM and original dataset

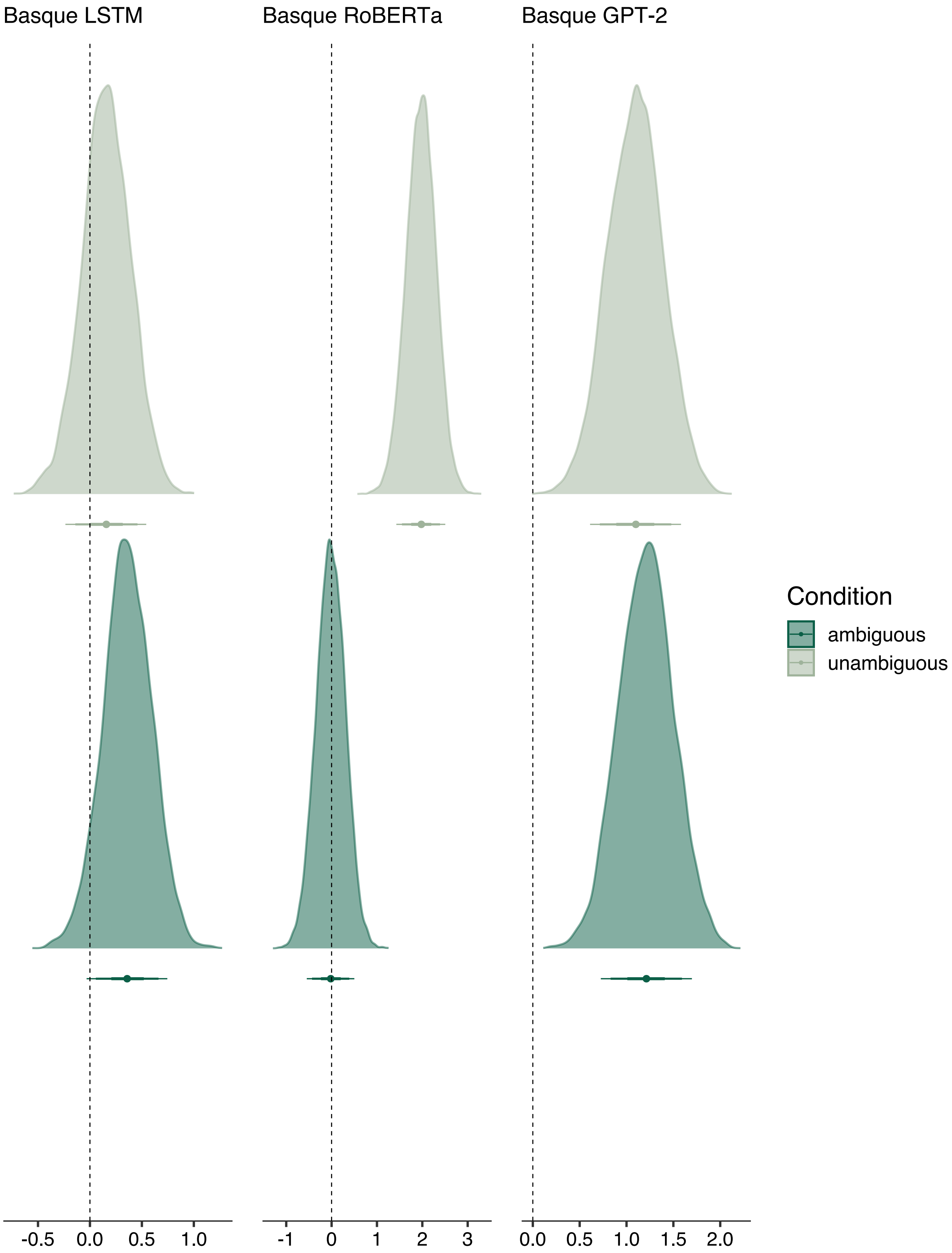

Figure S14: posterior distributions of the estimated surprisal difference between the critical conditions (agentive subject vs. patientive subject), displayed for both conditions (ambiguous vs. unambiguous). Surprisal is estimated by three types of language models. Only the population level is diplayed.

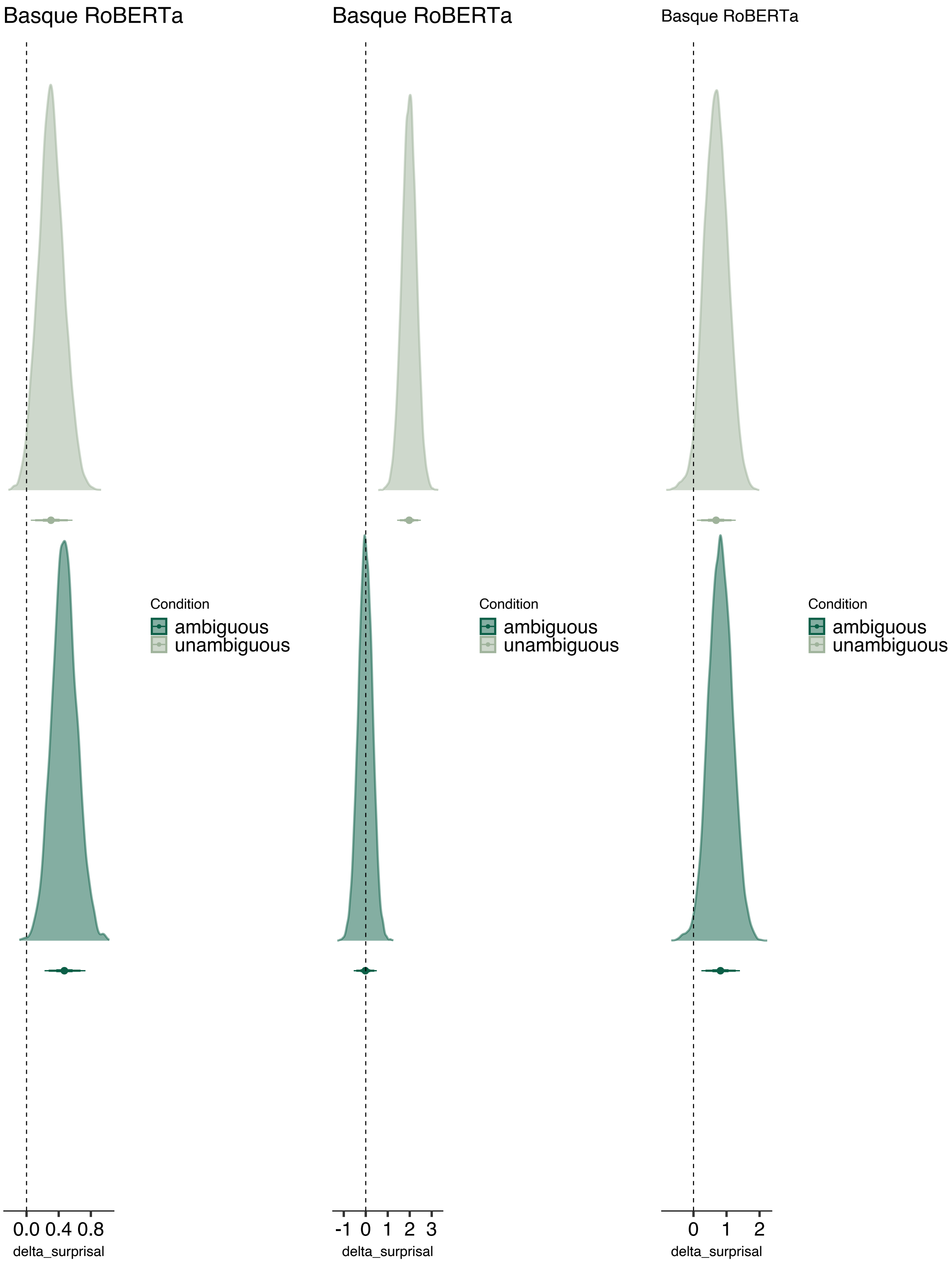

Figure S15: Results for the extended dataset: posterior distributions of the estimated surprisal difference between the critical conditions (agentive subject vs. patientive subject), displayed for both conditions (ambiguous vs. unambiguous). Surprisal is estimated by three types of language models. Only the population level is diplayed.

S3.5 T-tests

```
# relevel factors of main condition so that all should go into the same
# direction
```

```
de.lstm.original.m$first.NP <- relevel(de.lstm.original.m$first.NP,
                                       ref='subject')
de.tr.original.m$first.NP <- relevel(de.tr.original.m$first.NP, ref='subject')

de.gpt.original.m$first.NP <- relevel(de.gpt.original.m$first.NP, ref='subject')

eu.lstm.original.m$Semantic.Role <- relevel(eu.lstm.original.m$Semantic.Role,
                                       ref='Erg')
eu.tr.original.m$Semantic.Role <- relevel(eu.tr.original.m$Semantic.Role ,
                                          ref='Erg')

eu.gpt.original.m$Semantic.Role <- relevel(eu.gpt.original.m$Semantic.Role,
                                           ref='Erg')
hi.lstm.original.m$ambiguity <- relevel(hi.lstm.original.m$ambiguity,
                                            ref='UNAMB')
hi.tr.original.m$ambiguity <- relevel(hi.tr.original.m$ambiguity,
                                        ref='UNAMB')
hi.gpt.original.m$ambiguity <- relevel(hi.gpt.original.m$ambiguity,
                                      ref='UNAMB')
```

Filter out sentences that do not occur in both conditions for Hindi and Basque (not needed for German).

```
plus.hi.3 <- hi.lstm.original.m %>% group_by(sent_id) %>% count() %>% ungroup() %>%
  filter(n > 3)

hi.lstm.original.m.small <- hi.lstm.original.m %>% group_by(sent_id) %>%
  filter(sent_id %in% plus.hi.3$sent_id)

plus.eu.3 <- eu.lstm.original.m %>% group_by(sent_id) %>% count() %>% ungroup() %>%
  filter(n > 3)

eu.lstm.original.m.small <- eu.lstm.original.m %>% group_by(sent_id) %>%
  filter(sent_id %in% plus.eu.3$sent_id)
```

S3.5.1 German

```
by(de.lstm.original.m, de.lstm.original.m$verb.type, \(x) {
  t.test(
    formula = surprisal ~ first.NP,
    alternative = "two.sided",
    mu = 0,
    paired = TRUE,
    var.equal = FALSE,
    data = x
  )}
)
```

```
## de.lstm.original.m$verb.type: acc
##
##  Paired t-test
##
## data:  surprisal by first.NP
## t = -7.3837, df = 287, p-value = 1.677e-12
## alternative hypothesis: true mean difference is not equal to 0
## 95 percent confidence interval:
##  -1.2903013 -0.7471728
## sample estimates:
## mean difference
##       -1.018737
##
## ------------------------------------------------------------------------------------------------
## de.lstm.original.m$verb.type: dat
##
##  Paired t-test
##
## data:  surprisal by first.NP
## t = -2.8697, df = 223, p-value = 0.004503
## alternative hypothesis: true mean difference is not equal to 0
## 95 percent confidence interval:
##  -0.7815223 -0.1451631
## sample estimates:
## mean difference
##      -0.4633427
```

```
by(de.tr.original.m, de.tr.original.m$verb.type, \(x) {
  t.test(
    formula = surprisal ~ first.NP,
    alternative = "two.sided",
    mu = 0,
    paired = TRUE,
    var.equal = FALSE,
    data = x
  )}
)
```

```
## de.tr.original.m$verb.type: acc
##
##  Paired t-test
##
## data:  surprisal by first.NP
## t = -29.952, df = 1343, p-value < 2.2e-16
## alternative hypothesis: true mean difference is not equal to 0
## 95 percent confidence interval:
##  -2.571448 -2.255319
## sample estimates:
## mean difference
##       -2.413383
##
## ------------------------------------------------------------------------------------------------
## de.tr.original.m$verb.type: dat
##
```

```
##  Paired t-test
##
## data:  surprisal by first.NP
## t = -31.58, df = 1343, p-value < 2.2e-16
## alternative hypothesis: true mean difference is not equal to 0
## 95 percent confidence interval:
##  -2.593732 -2.290335
## sample estimates:
## mean difference
##       -2.442034
```

```r
by(de.gpt.original.m, de.gpt.original.m$verb.type, \(x) {
  t.test(
    formula = surprisal ~ first.NP,
    alternative = "two.sided",
    mu = 0,
    paired = TRUE,
    var.equal = FALSE,
    data = x
  )}
)
```

```
## de.gpt.original.m$verb.type: acc
##
##  Paired t-test
##
## data:  surprisal by first.NP
## t = -12.453, df = 335, p-value < 2.2e-16
## alternative hypothesis: true mean difference is not equal to 0
## 95 percent confidence interval:
##  -1.902362 -1.383350
## sample estimates:
## mean difference
##       -1.642856
##
## ---------------------------------------------------------------------------------------------
## de.gpt.original.m$verb.type: dat
##
##  Paired t-test
##
## data:  surprisal by first.NP
## t = -10.464, df = 335, p-value < 2.2e-16
## alternative hypothesis: true mean difference is not equal to 0
## 95 percent confidence interval:
##  -1.878461 -1.283965
## sample estimates:
## mean difference
##       -1.581213
```

S3.5.2 Hindi

```
by(hi.lstm.original.m.small, hi.lstm.original.m.small$aspect, \(x) {
  t.test(
    formula = surprisal ~ ambiguity,
    alternative = "two.sided",
    mu = 0,
    paired = TRUE,
    var.equal = FALSE,
    data = x
  )}
)
```

```
## hi.lstm.original.m.small$aspect: IPFV
##
##  Paired t-test
##
## data:  surprisal by ambiguity
## t = -1.9349, df = 18, p-value = 0.06888
## alternative hypothesis: true mean difference is not equal to 0
## 95 percent confidence interval:
##  -0.70858858  0.02915762
## sample estimates:
## mean difference
##      -0.3397155
##
## ------------------------------------------------------------------------------------------------------
## hi.lstm.original.m.small$aspect: PFV
##
##  Paired t-test
##
## data:  surprisal by ambiguity
## t = -0.76226, df = 18, p-value = 0.4558
## alternative hypothesis: true mean difference is not equal to 0
## 95 percent confidence interval:
##  -1.1928287  0.5576964
## sample estimates:
## mean difference
##      -0.3175661
```

```
by(hi.tr.original.m, hi.tr.original.m$aspect, \(x) {
  t.test(
    formula = surprisal ~ ambiguity,
    alternative = "two.sided",
    mu = 0,
    paired = TRUE,
    var.equal = FALSE,
    data = x
  )}
)
```

```
## hi.tr.original.m$aspect: IPFV
##
##  Paired t-test
```

```
##
## data:  surprisal by ambiguity
## t = -2.7676, df = 59, p-value = 0.007531
## alternative hypothesis: true mean difference is not equal to 0
## 95 percent confidence interval:
##  -1.5417153 -0.2478608
## sample estimates:
## mean difference
##       -0.894788
##
## ------------------------------------------------------------------------------------------
## hi.tr.original.m$aspect: PFV
##
##  Paired t-test
##
## data:  surprisal by ambiguity
## t = -0.82032, df = 59, p-value = 0.4153
## alternative hypothesis: true mean difference is not equal to 0
## 95 percent confidence interval:
##  -1.4282812  0.5977125
## sample estimates:
## mean difference
##      -0.4152843
```

```r
by(hi.gpt.original.m, hi.gpt.original.m$aspect, \(x) {
  t.test(
    formula = surprisal ~ ambiguity,
    alternative = "two.sided",
    mu = 0,
    paired = TRUE,
    var.equal = FALSE,
    data = x
  )}
)
```

```
## hi.gpt.original.m$aspect: IPFV
##
##  Paired t-test
##
## data:  surprisal by ambiguity
## t = -5.3152, df = 59, p-value = 1.711e-06
## alternative hypothesis: true mean difference is not equal to 0
## 95 percent confidence interval:
##  -0.6994853 -0.3168614
## sample estimates:
## mean difference
##      -0.5081734
##
## ------------------------------------------------------------------------------------------
## hi.gpt.original.m$aspect: PFV
##
##  Paired t-test
##
## data:  surprisal by ambiguity
```

```
## t = -6.9247, df = 59, p-value = 3.628e-09
## alternative hypothesis: true mean difference is not equal to 0
## 95 percent confidence interval:
##  -1.085329 -0.598703
## sample estimates:
## mean difference
##       -0.842016
```

S3.5.3 Basque

```r
by(eu.lstm.original.m.small, eu.lstm.original.m.small$Ambiguity, \(x) {
  t.test(
    formula = surprisal ~ Semantic.Role,
    alternative = "two.sided",
    mu = 0,
    paired = TRUE,
    var.equal = FALSE,
    data = x
  )}
)
```

```
## eu.lstm.original.m.small$Ambiguity: Amb
##
##  Paired t-test
##
## data:  surprisal by Semantic.Role
## t = -1.1133, df = 62, p-value = 0.2699
## alternative hypothesis: true mean difference is not equal to 0
## 95 percent confidence interval:
##  -0.7673943  0.2183654
## sample estimates:
## mean difference
##      -0.2745145
##
## ------------------------------------------------------------------------------------------------------
## eu.lstm.original.m.small$Ambiguity: Unamb
##
##  Paired t-test
##
## data:  surprisal by Semantic.Role
## t = -0.26373, df = 62, p-value = 0.7929
## alternative hypothesis: true mean difference is not equal to 0
## 95 percent confidence interval:
##  -0.5617005  0.4307620
## sample estimates:
## mean difference
##     -0.06546924
```

```r
by(eu.tr.original.m, eu.tr.original.m$Ambiguity, \(x) {
  t.test(
    formula = surprisal ~ Semantic.Role,
    alternative = "two.sided",
```

```r
    mu = 0,
    paired = TRUE,
    var.equal = FALSE,
    data = x
  )}
)
```

```
## eu.tr.original.m$Ambiguity: Amb
##
##  Paired t-test
##
## data:  surprisal by Semantic.Role
## t = 0.40248, df = 191, p-value = 0.6878
## alternative hypothesis: true mean difference is not equal to 0
## 95 percent confidence interval:
##  -0.4980268  0.7533769
## sample estimates:
## mean difference
##       0.1276751
##
## ------------------------------------------------------------------------------------------
## eu.tr.original.m$Ambiguity: Unamb
##
##  Paired t-test
##
## data:  surprisal by Semantic.Role
## t = -5.7423, df = 191, p-value = 3.627e-08
## alternative hypothesis: true mean difference is not equal to 0
## 95 percent confidence interval:
##  -2.759120 -1.348251
## sample estimates:
## mean difference
##       -2.053686
```

```r
by(eu.gpt.original.m, eu.gpt.original.m$Ambiguity, \(x) {
  t.test(
    formula = surprisal ~ Semantic.Role,
    alternative = "two.sided",
    mu = 0,
    paired = TRUE,
    var.equal = FALSE,
    data = x
  )}
)
```

```
## eu.gpt.original.m$Ambiguity: Amb
##
##  Paired t-test
##
## data:  surprisal by Semantic.Role
## t = -4.2648, df = 191, p-value = 3.145e-05
## alternative hypothesis: true mean difference is not equal to 0
## 95 percent confidence interval:
```

```
##  -1.9397948 -0.7129097
## sample estimates:
## mean difference
##       -1.326352
##
## ------------------------------------------------------------------------------------------------------------------------
## eu.gpt.original.m$Ambiguity: Unamb
##
##  Paired t-test
##
## data:  surprisal by Semantic.Role
## t = -3.9972, df = 191, p-value = 9.151e-05
## alternative hypothesis: true mean difference is not equal to 0
## 95 percent confidence interval:
##  -1.8955227 -0.6428979
## sample estimates:
## mean difference
##        -1.26921
```

3 Analysis 2: Predicting ERPs for German

S3: Study 2: Predicting ERPs (German)*

Eva Huber Sebastian Sauppe Arrate Isasi-Isasmendi
Ina Bornkessel-Schlesewsky Paola Merlo Balthasar Bickel

August 29, 2023

Contents

S3 German **1**
S3.1 Models . . . 1
S3.2 Model comparison with stacking . . . 14
S3.3 Model predictions . . . 15

REPRODUCIBILITY STATEMENT: All of the models included here were fitted on an M1 processor using R Version 4.2.2 and brms version 2.14.0.

S3 German

S3.1 Models

We model the EEG results in micro-volt (`mV` below). For this, we fit Bayesian Generalised Additive Models in `brms` with different predictors and compare models by means of stacking in order to find the model with highest predictive performance. In the model below `condition` refers to whether or not we expect a reanalysis (see main text).

We check convergence by ensuring that Effective Sample Size $ESS > 100$ and diagnostic $\hat{R} < 1.05$. Additionally, we perform posterior predictive checks to compare the predicted values to the observed data.

S3.1.1 Agent preference alone

```
german.data <- readRDS('../results/german_mv_surprisal_new.RDS')

if (!file.exists('../gams_fitted_models/gam_de_agent.RDS')){
  prior.brms <-  c(
    prior(normal(0,1), class = b),
    prior(exponential(1), class = sigma)
  )
  model.agent <-  brm(mV
                      ~ 1
                      + condition
```

*This document was `rmarkdown::render`'ed from an `R` script 'main_scripts/German_analysis2.R' available at https://osf.io/hbj67.

```r
                    + trial_number.Z
                    + t2(x, y, by = condition, k = 10)
                    + t2(x, y, Participant, bs = "re")
                    + t2(x, y, Stimulus, bs = "re"),
                    data = german.data,
                    sample_prior = TRUE,
                    prior = prior.brms,
                    chains = 4, iter = 2000, cores = 4
  )
  saveRDS(model.agent, '../gams_fitted_models/gam_de_agent.RDS')
}
model.de.agent <- readRDS('../gams_fitted_models/gam_de_agent.RDS')
summary(model.de.agent)
```

```
##  Family: gaussian
##   Links: mu = identity; sigma = identity
## Formula: mV ~ 1 + condition + trial_number.Z + t2(x, y, by = condition, k = 10) + t2(x, y, Participa
##    Data: german.data (Number of observations: 20961)
## Samples: 4 chains, each with iter = 2000; warmup = 1000; thin = 1;
##          total post-warmup samples = 4000
##
## Smooth Terms:
##                                 Estimate Est.Error l-95% CI u-95% CI Rhat Bulk_ESS Tail_ESS
## sds(t2xyconditionAbias_1)           1.49      1.34     0.06     5.09 1.00     2308     2694
## sds(t2xyconditionAbias_2)           2.92      2.73     0.11     9.37 1.00     1785     2716
## sds(t2xyconditionAbias_3)           5.74      3.25     0.63    13.38 1.00     1892     1462
## sds(t2xyconditionnonAbias_1)        1.12      1.08     0.03     3.99 1.00     2373     2194
## sds(t2xyconditionnonAbias_2)        3.02      2.64     0.11     9.81 1.00     1727     2718
## sds(t2xyconditionnonAbias_3)        5.73      2.68     1.40    12.22 1.00     1509     1192
## sds(t2xyParticipant_1)              0.15      0.11     0.01     0.39 1.00     2385     2704
## sds(t2xyStimulus_1)                 0.14      0.10     0.01     0.38 1.00     2264     1794
##
## Population-Level Effects:
##                             Estimate Est.Error l-95% CI u-95% CI Rhat Bulk_ESS Tail_ESS
## Intercept                       1.05      0.15     0.75     1.35 1.00     4096     3345
## condition2                      0.09      0.19    -0.28     0.48 1.00     3775     3296
## trial_number.Z                 -0.62      0.07    -0.76    -0.48 1.00     9384     2496
## t2xy:conditionAbias_1           0.09      0.19    -0.27     0.46 1.00     3308     3079
## t2xy:conditionAbias_2           0.09      0.15    -0.19     0.40 1.00     5073     3162
## t2xy:conditionAbias_3           0.05      0.20    -0.34     0.45 1.00     6395     3509
## t2xy:conditionnonAbias_1        0.53      0.16     0.22     0.83 1.00     2379     2059
## t2xy:conditionnonAbias_2        0.20      0.12    -0.06     0.43 1.00     5003     3267
## t2xy:conditionnonAbias_3        0.22      0.17    -0.11     0.55 1.00     6145     2789
##
## Family Specific Parameters:
##       Estimate Est.Error l-95% CI u-95% CI Rhat Bulk_ESS Tail_ESS
## sigma     9.39      0.05     9.29     9.48 1.00    10137     2674
##
## Samples were drawn using sampling(NUTS). For each parameter, Bulk_ESS
## and Tail_ESS are effective sample size measures, and Rhat is the potential
## scale reduction factor on split chains (at convergence, Rhat = 1).
```

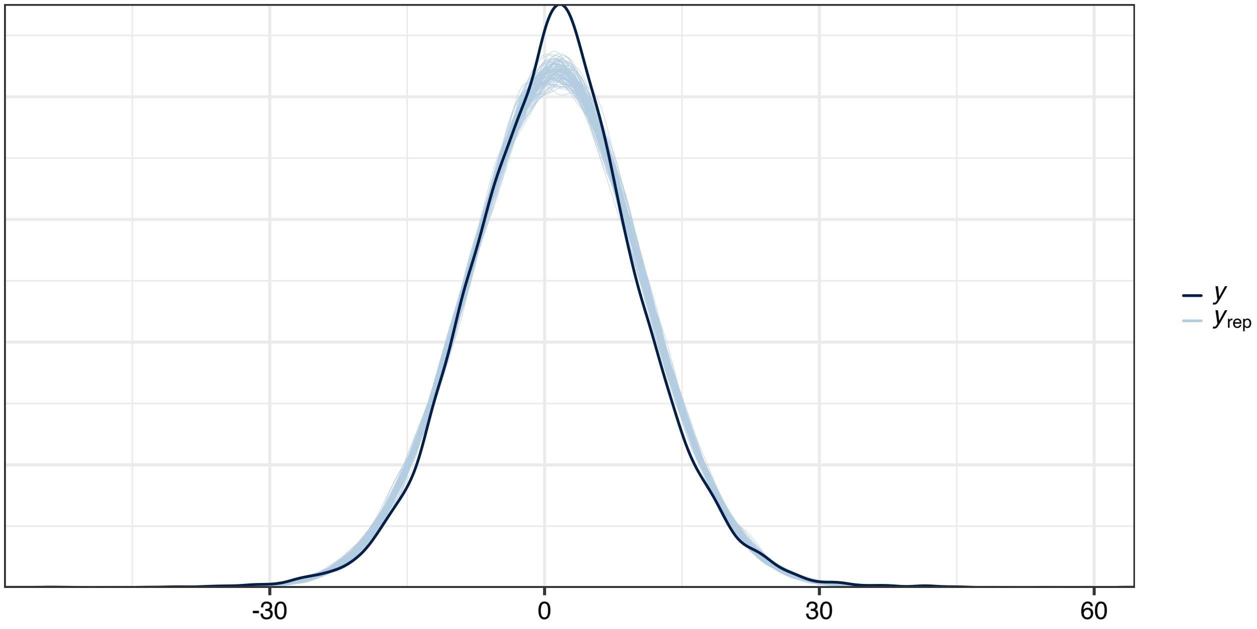

S3.1.2 Surprisal alone

LSTM-based surprisal:

```
if (!file.exists('../gams_fitted_models/gam_de_lstm.RDS')){
  prior.brms <-  c(
    prior(normal(0,1), class = b),
    prior(exponential(1), class = sigma)
  )
  model.surprisal.lstm <-  brm(mV
                               ~ 1
                               + trial_number.Z
                               + surprisal.lstm.Z
                               + t2(x, y, by = surprisal.lstm.Z, k = 10)
                               + t2(x, y, Participant, bs = "re")
                               + t2(x, y, Stimulus, bs = "re"),
                               prior = prior.brms,
                               sample_prior = TRUE,
                               data = german.data,
                               chains = 4, iter = 2000, cores = 4
  )
  saveRDS(model.surprisal.lstm,
          '../gams_fitted_models/gam_de_lstm.RDS')
}
model.de.surplstm <- readRDS('../gams_fitted_models/gam_de_lstm.RDS')
summary(model.de.surplstm)
```

```
##  Family: gaussian
##   Links: mu = identity; sigma = identity
## Formula: mV ~ 1 + trial_number.Z + surprisal.lstm.Z + t2(x, y, by = surprisal.lstm.Z, k = 10) + t2(x
##    Data: german.data (Number of observations: 20961)
## Samples: 4 chains, each with iter = 2000; warmup = 1000; thin = 1;
```

```
##          total post-warmup samples = 4000
##
## Smooth Terms:
##                               Estimate Est.Error l-95% CI u-95% CI Rhat Bulk_ESS Tail_ESS
## sds(t2xysurprisal.lstm.Z_1)       0.85      0.78     0.03     2.94 1.00     2441     2141
## sds(t2xysurprisal.lstm.Z_2)       1.82      1.67     0.06     6.19 1.00     2188     1952
## sds(t2xysurprisal.lstm.Z_3)       2.31      1.81     0.09     6.93 1.00     1671     1421
## sds(t2xyParticipant_1)            0.15      0.11     0.01     0.41 1.01     1616     1165
## sds(t2xyStimulus_1)               0.14      0.11     0.00     0.40 1.00     1767     1611
##
## Population-Level Effects:
##                           Estimate Est.Error l-95% CI u-95% CI Rhat Bulk_ESS Tail_ESS
## Intercept                     1.19      0.06     1.06     1.31 1.00     6957     3221
## trial_number.Z               -0.57      0.06    -0.70    -0.45 1.00     7189     2986
## surprisal.lstm.Z             -0.30      0.75    -1.78     1.16 1.00     2961     3055
## t2xy:surprisal.lstm.Z_1      -0.33      0.68    -1.65     0.99 1.00     2962     2869
## t2xy:surprisal.lstm.Z_2      -0.38      0.09    -0.56    -0.20 1.00     3340     3234
## t2xy:surprisal.lstm.Z_3       0.12      0.09    -0.04     0.30 1.00     3533     2926
## t2xy:surprisal.lstm.Z_4       0.07      0.12    -0.16     0.30 1.00     4991     2987
##
## Family Specific Parameters:
##       Estimate Est.Error l-95% CI u-95% CI Rhat Bulk_ESS Tail_ESS
## sigma     9.36      0.05     9.27     9.45 1.00    14016     2816
##
## Samples were drawn using sampling(NUTS). For each parameter, Bulk_ESS
## and Tail_ESS are effective sample size measures, and Rhat is the potential
## scale reduction factor on split chains (at convergence, Rhat = 1).
```

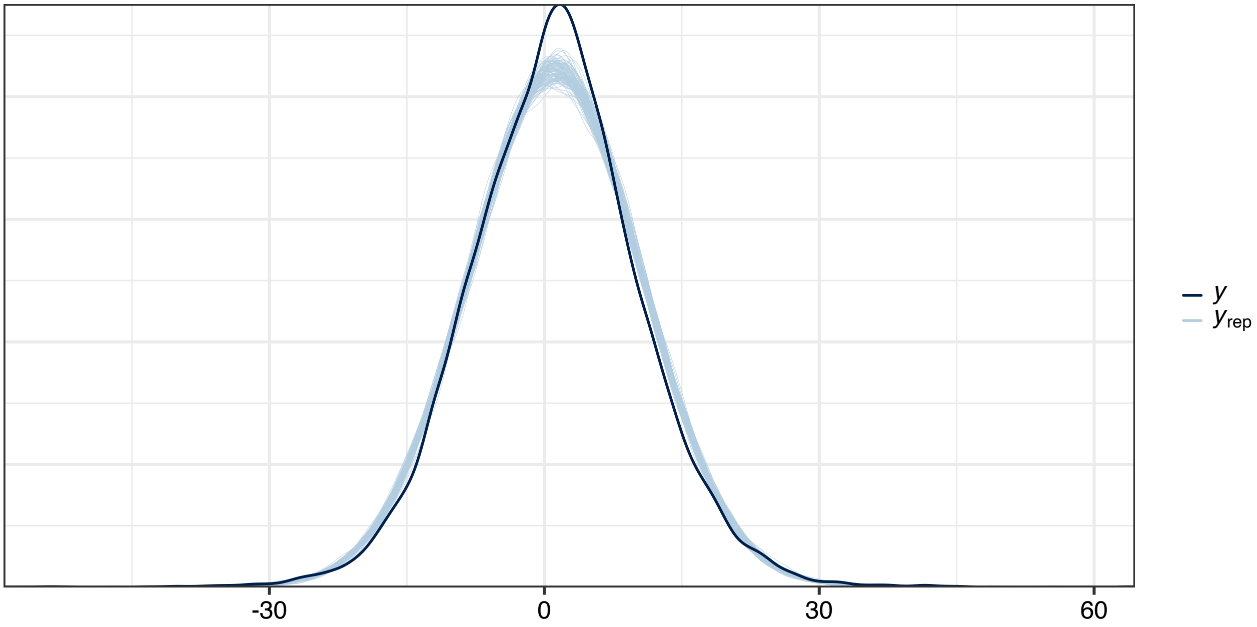

BERT-based surprisal:

```
if (!file.exists('../gams_fitted_models/gam_de_bert.RDS')){
  prior.brms <-  c(
    prior(normal(0,1), class = b),
    prior(exponential(1), class = sigma)
```

```r
  )
  model.surprisal.tr <-  brm(mV
                             ~ 1
                             + trial_number.Z
                             + surprisal.tr.Z
                             + t2(x, y, by = surprisal.tr.Z, k = 10)
                             + t2(x, y, Participant, bs = "re")
                             + t2(x, y, Stimulus, bs = "re"),
                             prior = prior.brms,
                             sample_prior = TRUE,
                             data = german.data,
                             chains = 4, iter = 2000, cores = 4
  )
  saveRDS(model.surprisal.tr,
          '../gams_fitted_models/gam_de_bert.RDS')
}
model.de.surptr <-readRDS('../gams_fitted_models/gam_de_bert.RDS')
summary(model.de.surptr)
```

```
##  Family: gaussian
##   Links: mu = identity; sigma = identity
## Formula: mV ~ 1 + trial_number.Z + surprisal.tr.Z + t2(x, y, by = surprisal.tr.Z, k = 10) + t2(x, y,
##    Data: german.data (Number of observations: 20961)
## Samples: 4 chains, each with iter = 2000; warmup = 1000; thin = 1;
##          total post-warmup samples = 4000
##
## Smooth Terms:
##                            Estimate Est.Error l-95% CI u-95% CI Rhat Bulk_ESS Tail_ESS
## sds(t2xysurprisal.tr.Z_1)      0.89      0.82     0.03     3.02 1.00     2227     2056
## sds(t2xysurprisal.tr.Z_2)      1.59      1.44     0.06     5.47 1.00     1985     1828
## sds(t2xysurprisal.tr.Z_3)      1.92      1.64     0.07     6.20 1.00     1902     1974
## sds(t2xyParticipant_1)         0.15      0.11     0.01     0.41 1.00     2096     1943
## sds(t2xyStimulus_1)            0.14      0.11     0.00     0.39 1.00     1668     1434
##
## Population-Level Effects:
##                        Estimate Est.Error l-95% CI u-95% CI Rhat Bulk_ESS Tail_ESS
## Intercept                  1.19      0.07     1.05     1.32 1.00     7341     2320
## trial_number.Z            -0.60      0.06    -0.72    -0.48 1.00     8825     2716
## surprisal.tr.Z            -0.15      0.76    -1.61     1.32 1.00     3104     2850
## t2xy:surprisal.tr.Z_1     -0.15      0.68    -1.47     1.18 1.00     3137     2986
## t2xy:surprisal.tr.Z_2     -0.22      0.09    -0.38    -0.04 1.00     4326     3394
## t2xy:surprisal.tr.Z_3      0.04      0.08    -0.12     0.21 1.00     5172     3362
## t2xy:surprisal.tr.Z_4     -0.08      0.12    -0.31     0.16 1.00     5300     3089
##
## Family Specific Parameters:
##       Estimate Est.Error l-95% CI u-95% CI Rhat Bulk_ESS Tail_ESS
## sigma     9.39      0.05     9.30     9.48 1.00    12235     2784
##
## Samples were drawn using sampling(NUTS). For each parameter, Bulk_ESS
## and Tail_ESS are effective sample size measures, and Rhat is the potential
## scale reduction factor on split chains (at convergence, Rhat = 1).
```

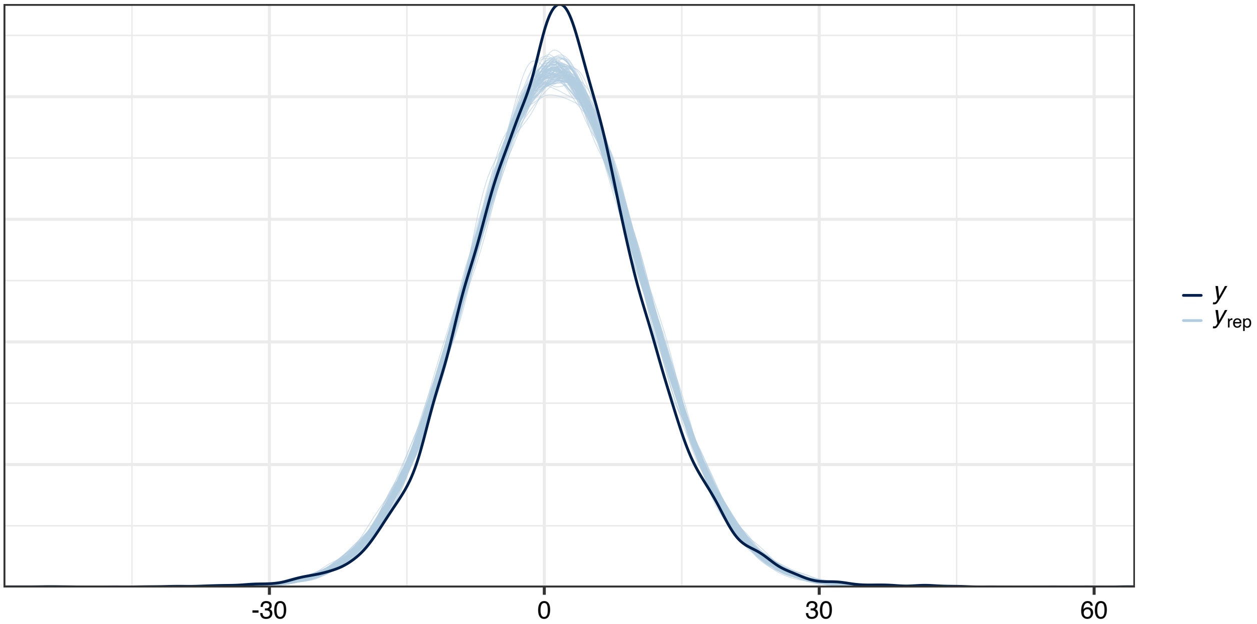

GPT-based surprisal:

```
if (!file.exists('../gams_fitted_models/gam_de_gpt.RDS')){
  prior.brms <-  c(
    prior(normal(0,1), class = b),
    prior(exponential(1), class = sigma)
  )
  model.surprisal.gpt <-  brm(mV
                               ~ 1
                               + trial_number.Z
                               + surprisal.gpt.Z
                               + t2(x, y, by = surprisal.gpt.Z, k = 10)
                               + t2(x, y, Participant, bs = "re")
                               + t2(x, y, Stimulus, bs = "re"),
                               prior = prior.brms,
                               sample_prior = TRUE,
                               data = german.data,
                               chains = 4, iter = 2000, cores = 4
  )
  saveRDS(model.surprisal.gpt,
          '../gams_fitted_models/gam_de_gpt.RDS')
}
model.de.surpgpt <- readRDS('../gams_fitted_models/gam_de_gpt.RDS')
summary(model.de.surpgpt)
```

```
##  Family: gaussian
##   Links: mu = identity; sigma = identity
## Formula: mV ~ 1 + trial_number.Z + surprisal.gpt.Z + t2(x, y, by = surprisal.gpt.Z, k = 10) + t2(x, 
##    Data: german.data (Number of observations: 20961)
## Samples: 4 chains, each with iter = 2000; warmup = 1000; thin = 1;
##          total post-warmup samples = 4000
##
## Smooth Terms:
##                               Estimate Est.Error l-95% CI u-95% CI Rhat Bulk_ESS Tail_ESS
```

```
## sds(t2xysurprisal.gpt.Z_1)     0.85      0.79     0.02     2.88 1.00     2478     1636
## sds(t2xysurprisal.gpt.Z_2)     1.78      1.60     0.07     5.91 1.00     1826     1841
## sds(t2xysurprisal.gpt.Z_3)     1.79      1.67     0.06     6.14 1.00     1953     2109
## sds(t2xyParticipant_1)         0.15      0.11     0.00     0.42 1.00     1848     1630
## sds(t2xyStimulus_1)            0.14      0.10     0.01     0.39 1.00     1900     1918
##
## Population-Level Effects:
##                           Estimate Est.Error l-95% CI u-95% CI Rhat Bulk_ESS Tail_ESS
## Intercept                     1.19      0.06     1.06     1.31 1.00     8010     2736
## trial_number.Z               -0.56      0.07    -0.69    -0.43 1.00     7363     2960
## surprisal.gpt.Z              -0.15      0.74    -1.66     1.31 1.00     3086     2788
## t2xy:surprisal.gpt.Z_1       -0.15      0.66    -1.47     1.17 1.00     3096     2814
## t2xy:surprisal.gpt.Z_2       -0.22      0.09    -0.38    -0.04 1.00     4095     3227
## t2xy:surprisal.gpt.Z_3        0.04      0.09    -0.12     0.22 1.00     3830     2668
## t2xy:surprisal.gpt.Z_4       -0.09      0.11    -0.32     0.12 1.00     4409     3015
##
## Family Specific Parameters:
##       Estimate Est.Error l-95% CI u-95% CI Rhat Bulk_ESS Tail_ESS
## sigma     9.39      0.05     9.30     9.48 1.00    12165     2817
##
## Samples were drawn using sampling(NUTS). For each parameter, Bulk_ESS
## and Tail_ESS are effective sample size measures, and Rhat is the potential
## scale reduction factor on split chains (at convergence, Rhat = 1).
```

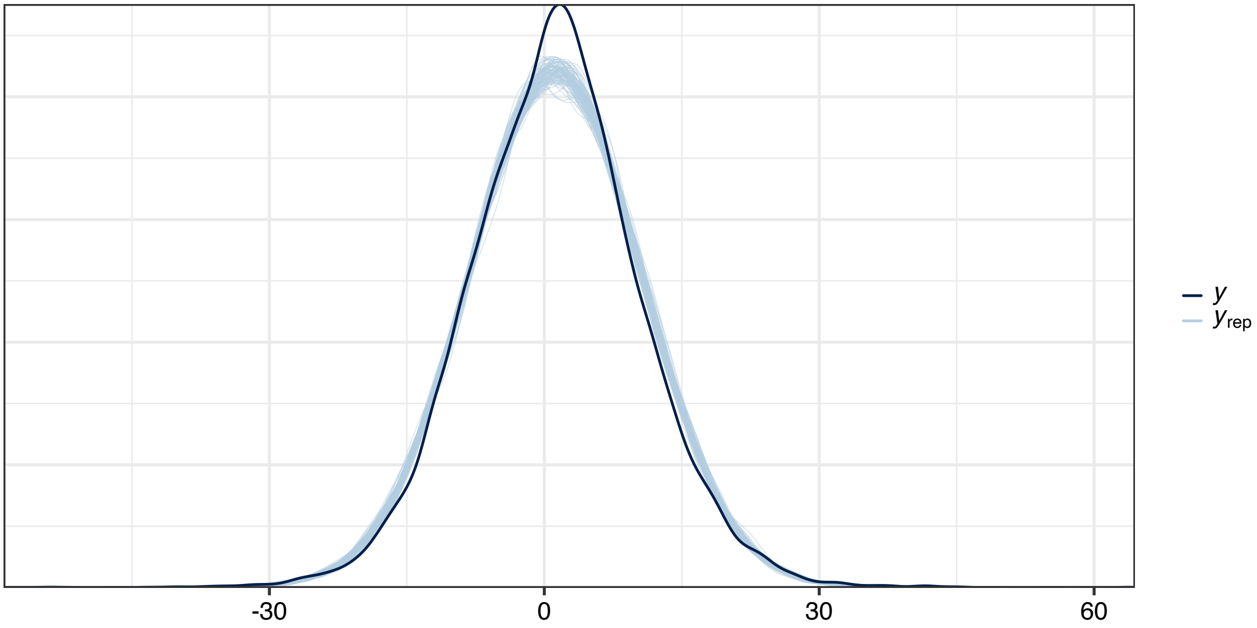

S3.1.3 Agent preference and surprisal

LSTM-based surprisal:

```
if (!file.exists('../gams_fitted_models/gam_de_agent_lstm.RDS')){
  prior.brms <-  c(
    prior(normal(0,1), class = b),
    prior(exponential(1), class = sigma)
```

```
  )
  model.agent.surprisal.lstm <-  brm(mV
                                     ~ 1
                                     + trial_number.Z
                                     + surprisal.lstm.Z
                                     + condition
                                     + t2(x, y, by = condition, k = 10)
                                     + t2(x, y, by = surprisal.lstm.Z, k = 10)
                                     + t2(x, y, Participant, bs = "re")
                                     + t2(x, y, Stimulus, bs = "re"),
                                     prior = prior.brms,
                                     sample_prior = TRUE,
                                     data = german.data,
                                     chains = 4, iter = 2000, cores = 4
  )
  saveRDS(model.agent.surprisal.lstm,
          '../gams_fitted_models/gam_de_agent_lstm.RDS')
}
model.de.agent.surplstm <- readRDS('../gams_fitted_models/gam_de_agent_lstm.RDS')

summary(model.de.agent.surplstm)
```

```
##  Family: gaussian
##   Links: mu = identity; sigma = identity
## Formula: mV ~ 1 + trial_number.Z + surprisal.lstm.Z + condition + t2(x, y, by = condition, k = 10) +
##    Data: german.data (Number of observations: 20961)
## Samples: 4 chains, each with iter = 2000; warmup = 1000; thin = 1;
##          total post-warmup samples = 4000
##
## Smooth Terms:
##                               Estimate Est.Error l-95% CI u-95% CI Rhat Bulk_ESS Tail_ESS
## sds(t2xyconditionAbias_1)         1.54      1.35     0.07     5.06 1.00     2058     2138
## sds(t2xyconditionAbias_2)         2.89      2.51     0.11     9.46 1.00     1805     2273
## sds(t2xyconditionAbias_3)         5.95      3.36     0.69    14.17 1.00     1068      695
## sds(t2xyconditionnonAbias_1)      1.10      1.01     0.03     3.83 1.00     2008     1992
## sds(t2xyconditionnonAbias_2)      2.95      2.59     0.10     9.48 1.00     1265     1468
## sds(t2xyconditionnonAbias_3)      5.64      2.68     1.58    11.99 1.00     1644     1247
## sds(t2xysurprisal.lstm.Z_1)       0.82      0.78     0.02     2.92 1.00     2316     1922
## sds(t2xysurprisal.lstm.Z_2)       1.79      1.66     0.06     6.32 1.00     1729     1640
## sds(t2xysurprisal.lstm.Z_3)       2.27      1.80     0.10     6.96 1.00     1657     1767
## sds(t2xyParticipant_1)            0.15      0.11     0.00     0.40 1.00     1708     1246
## sds(t2xyStimulus_1)               0.14      0.10     0.01     0.37 1.00     1810     1564
##
## Population-Level Effects:
##                           Estimate Est.Error l-95% CI u-95% CI Rhat Bulk_ESS Tail_ESS
## Intercept                     1.19      0.15     0.90     1.49 1.00     2510     2614
## trial_number.Z               -0.56      0.07    -0.69    -0.43 1.00     5756     3375
## surprisal.lstm.Z             -0.30      0.75    -1.77     1.20 1.00     2957     2673
## condition2                   -0.13      0.19    -0.52     0.24 1.00     2134     2939
## t2xy:conditionAbias_1         0.16      0.19    -0.20     0.54 1.00     1871     2313
## t2xy:conditionAbias_2         0.07      0.15    -0.22     0.36 1.00     3818     2810
## t2xy:conditionAbias_3         0.04      0.19    -0.33     0.42 1.00     4194     3139
## t2xy:conditionnonAbias_1      0.50      0.16     0.19     0.80 1.00     2066     2064
```

```
## t2xy:conditionnonAbias_2     0.21      0.12     -0.03     0.45 1.00     2764     2613
## t2xy:conditionnonAbias_3     0.23      0.16     -0.09     0.56 1.00     3065     3088
## t2xy:surprisal.lstm.Z_1     -0.35      0.67     -1.70     0.97 1.00     2971     2643
## t2xy:surprisal.lstm.Z_2     -0.35      0.09     -0.55    -0.18 1.00     3741     2971
## t2xy:surprisal.lstm.Z_3      0.13      0.09     -0.04     0.31 1.00     4040     3125
## t2xy:surprisal.lstm.Z_4      0.09      0.11     -0.14     0.32 1.00     4190     3274
##
## Family Specific Parameters:
##       Estimate Est.Error l-95% CI u-95% CI Rhat Bulk_ESS Tail_ESS
## sigma     9.35      0.05     9.27     9.44 1.00     8444     2514
##
## Samples were drawn using sampling(NUTS). For each parameter, Bulk_ESS
## and Tail_ESS are effective sample size measures, and Rhat is the potential
## scale reduction factor on split chains (at convergence, Rhat = 1).
```

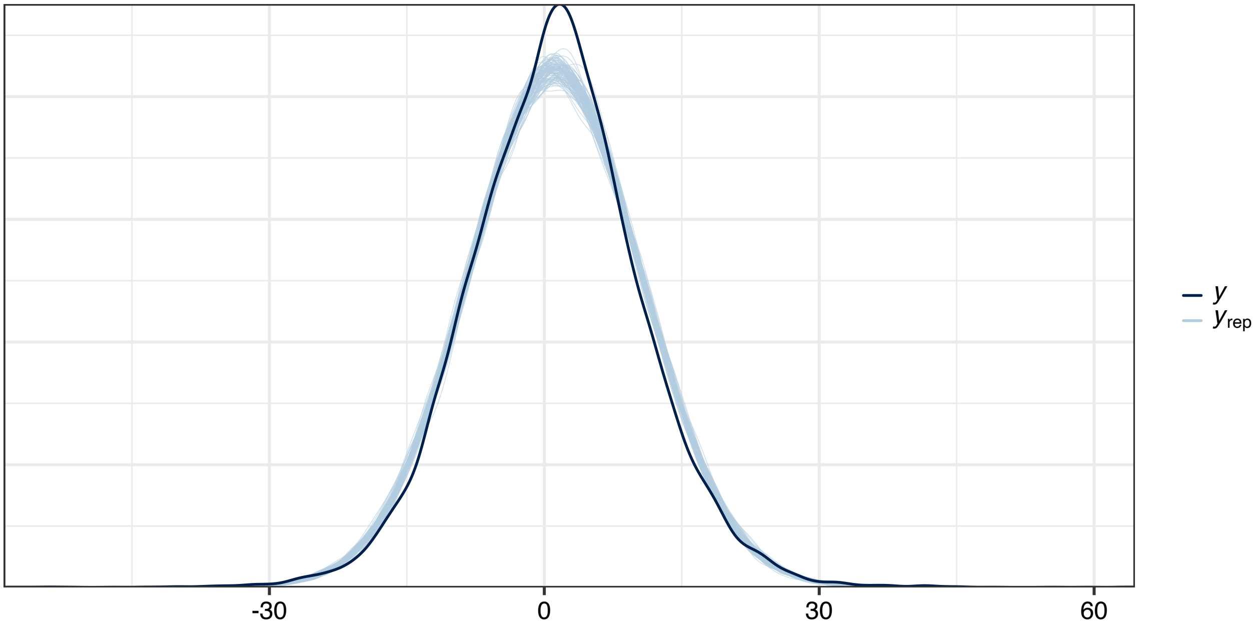

BERT-based surprisal:

```
if (!file.exists('../gams_fitted_models/gam_de_agent_bert.RDS')){
  prior.brms <-  c(
    prior(normal(0,1), class = b),
    prior(exponential(1), class = sigma)
  )
  model.agent.surprisal.tr <-  brm(mV
                                ~ 1
                                + trial_number.Z
                                + surprisal.tr.Z
                                + condition
                                + t2(x, y, by = condition, k = 10)
                                + t2(x, y, by = surprisal.tr.Z, k = 10)
                                + t2(x, y, Participant, bs = "re")
                                + t2(x, y, Stimulus, bs = "re"),
                                prior = prior.brms,
                                sample_prior = TRUE,
                                data = german.data,
```

```r
                                   chains = 4, iter = 2000, cores = 4
  )
  saveRDS(model.agent.surprisal.lstm,
          '../gams_fitted_models/gam_de_agent_bert.RDS')
}
model.de.agent.surptr <- readRDS('../gams_fitted_models/gam_de_agent_bert.RDS')

summary(model.de.agent.surptr)
```

```
##  Family: gaussian
##   Links: mu = identity; sigma = identity
## Formula: mV ~ 1 + trial_number.Z + surprisal.tr.Z + condition + t2(x, y, by = condition, k = 10) + t2
##    Data: german.data (Number of observations: 20961)
## Samples: 4 chains, each with iter = 2000; warmup = 1000; thin = 1;
##          total post-warmup samples = 4000
##
## Smooth Terms:
##                               Estimate Est.Error l-95% CI u-95% CI Rhat Bulk_ESS Tail_ESS
## sds(t2xyconditionAbias_1)         1.58      1.41     0.06     5.27 1.00     2336     2199
## sds(t2xyconditionAbias_2)         2.91      2.51     0.10     9.39 1.00     1973     1670
## sds(t2xyconditionAbias_3)         5.94      3.31     1.00    14.11 1.00     1674     1274
## sds(t2xyconditionnonAbias_1)      1.13      1.04     0.04     3.92 1.00     2083     2291
## sds(t2xyconditionnonAbias_2)      3.06      2.63     0.12     9.67 1.00     1293     2111
## sds(t2xyconditionnonAbias_3)      5.78      2.70     1.60    12.19 1.00     2096     1899
## sds(t2xysurprisal.tr.Z_1)         0.92      0.81     0.03     3.06 1.00     2247     2164
## sds(t2xysurprisal.tr.Z_2)         1.68      1.51     0.05     5.55 1.00     2276     2131
## sds(t2xysurprisal.tr.Z_3)         2.01      1.74     0.08     6.52 1.00     1929     1975
## sds(t2xyParticipant_1)            0.14      0.11     0.01     0.40 1.00     1688     2144
## sds(t2xyStimulus_1)               0.14      0.10     0.01     0.39 1.00     2026     1977
##
## Population-Level Effects:
##                           Estimate Est.Error l-95% CI u-95% CI Rhat Bulk_ESS Tail_ESS
## Intercept                     1.25      0.16     0.93     1.57 1.00     3040     2981
## trial_number.Z               -0.57      0.07    -0.71    -0.44 1.00     6925     3050
## surprisal.tr.Z               -0.17      0.75    -1.60     1.30 1.00     3233     3157
## condition2                   -0.23      0.21    -0.64     0.17 1.00     2967     2984
## t2xy:conditionAbias_1         0.19      0.19    -0.16     0.59 1.00     2666     3101
## t2xy:conditionAbias_2         0.05      0.15    -0.25     0.35 1.00     3389     2628
## t2xy:conditionAbias_3         0.08      0.21    -0.32     0.48 1.00     4166     3506
## t2xy:conditionnonAbias_1      0.48      0.16     0.17     0.79 1.00     2368     2595
## t2xy:conditionnonAbias_2      0.22      0.12    -0.04     0.46 1.00     2614     2296
## t2xy:conditionnonAbias_3      0.21      0.17    -0.13     0.53 1.00     3765     3188
## t2xy:surprisal.tr.Z_1        -0.17      0.67    -1.49     1.13 1.00     3217     3185
## t2xy:surprisal.tr.Z_2        -0.17      0.10    -0.35     0.04 1.00     3395     2793
## t2xy:surprisal.tr.Z_3         0.08      0.09    -0.10     0.26 1.00     3570     2748
## t2xy:surprisal.tr.Z_4        -0.06      0.12    -0.29     0.18 1.00     4941     3404
##
## Family Specific Parameters:
##       Estimate Est.Error l-95% CI u-95% CI Rhat Bulk_ESS Tail_ESS
## sigma     9.38      0.05     9.29     9.47 1.00    10208     2504
##
## Samples were drawn using sampling(NUTS). For each parameter, Bulk_ESS
## and Tail_ESS are effective sample size measures, and Rhat is the potential
```

```
## scale reduction factor on split chains (at convergence, Rhat = 1).
```

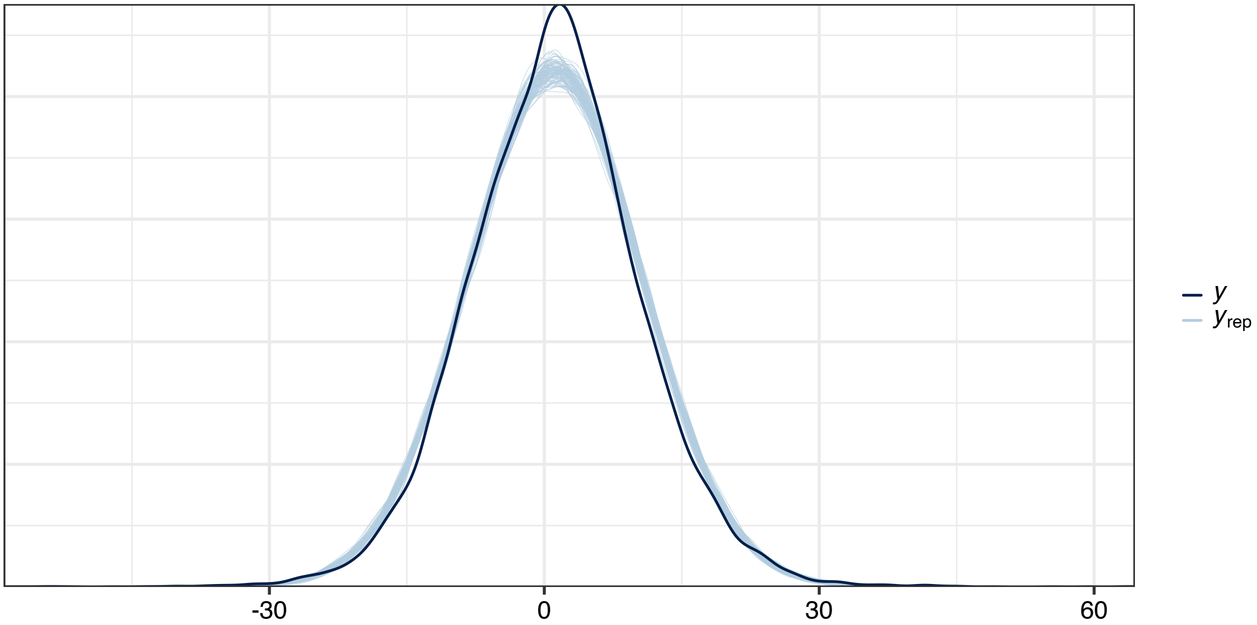

GPT-based surprisal

```
if (!file.exists('../gams_fitted_models/gam_de_agent_gpt.RDS')){
  prior.brms <-  c(
    prior(normal(0,1), class = b),
    prior(exponential(1), class = sigma)
  )
  model.agent.surprisal.gpt <-  brm(mV
                                    ~ 1
                                    + trial_number.Z
                                    + surprisal.gpt.Z
                                    + condition
                                    + t2(x, y, by = condition, k = 10)
                                    + t2(x, y, by = surprisal.gpt.Z, k = 10)
                                    + t2(x, y, Participant, bs = "re")
                                    + t2(x, y, Stimulus, bs = "re"),
                                    prior = prior.brms,
                                    sample_prior = TRUE,
                                    data = german.data,
                                    chains = 4, iter = 2000, cores = 4
  )
  saveRDS(model.agent.surprisal.gpt,
          '../gams_fitted_models/gam_de_agent_gpt.RDS')
}
model.de.agent.surpgpt <- readRDS('../gams_fitted_models/gam_de_agent_gpt.RDS')

summary(model.de.agent.surpgpt)
```

```
##  Family: gaussian
##   Links: mu = identity; sigma = identity
## Formula: mV ~ 1 + trial_number.Z + surprisal.gpt.Z + condition + t2(x, y, by = condition, k = 10) +
```

```
##    Data: german.data (Number of observations: 20961)
## Samples: 4 chains, each with iter = 2000; warmup = 1000; thin = 1;
##          total post-warmup samples = 4000
##
## Smooth Terms:
##                               Estimate Est.Error l-95% CI u-95% CI Rhat Bulk_ESS Tail_ESS
## sds(t2xyconditionAbias_1)         1.48      1.35     0.05     5.05 1.00     2176     1894
## sds(t2xyconditionAbias_2)         2.89      2.60     0.08     9.53 1.00     1414     1579
## sds(t2xyconditionAbias_3)         5.88      3.36     0.70    13.87 1.00     1640     1127
## sds(t2xyconditionnonAbias_1)      1.12      1.05     0.04     3.88 1.00     2208     1809
## sds(t2xyconditionnonAbias_2)      2.98      2.55     0.11     9.23 1.00     1173     1907
## sds(t2xyconditionnonAbias_3)      5.79      2.87     1.30    12.85 1.00     1412      869
## sds(t2xysurprisal.gpt.Z_1)        0.89      0.87     0.03     3.17 1.00     2475     1649
## sds(t2xysurprisal.gpt.Z_2)        1.82      1.60     0.06     5.88 1.00     1911     1674
## sds(t2xysurprisal.gpt.Z_3)        2.00      1.81     0.07     6.84 1.00     2452     2116
## sds(t2xyParticipant_1)            0.15      0.11     0.01     0.41 1.00     1738     1824
## sds(t2xyStimulus_1)               0.14      0.11     0.00     0.39 1.00     1869     1385
##
## Population-Level Effects:
##                           Estimate Est.Error l-95% CI u-95% CI Rhat Bulk_ESS Tail_ESS
## Intercept                     1.42      0.17     1.08     1.76 1.00     2914     2670
## trial_number.Z               -0.48      0.07    -0.62    -0.34 1.00     5452     2711
## surprisal.gpt.Z              -0.20      0.74    -1.64     1.23 1.00     2979     3227
## condition2                   -0.48      0.22    -0.93    -0.05 1.00     2908     2990
## t2xy:conditionAbias_1         0.20      0.19    -0.17     0.60 1.00     2223     2909
## t2xy:conditionAbias_2         0.03      0.16    -0.27     0.35 1.00     3401     2775
## t2xy:conditionAbias_3         0.10      0.21    -0.32     0.53 1.00     3480     2958
## t2xy:conditionnonAbias_1      0.47      0.16     0.16     0.81 1.00     1898     2712
## t2xy:conditionnonAbias_2      0.23      0.13    -0.02     0.47 1.00     3024     2754
## t2xy:conditionnonAbias_3      0.20      0.17    -0.14     0.52 1.00     3581     3234
## t2xy:surprisal.gpt.Z_1       -0.23      0.66    -1.53     1.05 1.00     2993     3131
## t2xy:surprisal.gpt.Z_2       -0.15      0.10    -0.34     0.06 1.00     2975     2861
## t2xy:surprisal.gpt.Z_3        0.09      0.10    -0.10     0.29 1.00     3128     3005
## t2xy:surprisal.gpt.Z_4       -0.07      0.13    -0.33     0.18 1.00     3743     2983
##
## Family Specific Parameters:
##       Estimate Est.Error l-95% CI u-95% CI Rhat Bulk_ESS Tail_ESS
## sigma     9.38      0.05     9.29     9.47 1.00     9439     2885
##
## Samples were drawn using sampling(NUTS). For each parameter, Bulk_ESS
## and Tail_ESS are effective sample size measures, and Rhat is the potential
## scale reduction factor on split chains (at convergence, Rhat = 1).
```

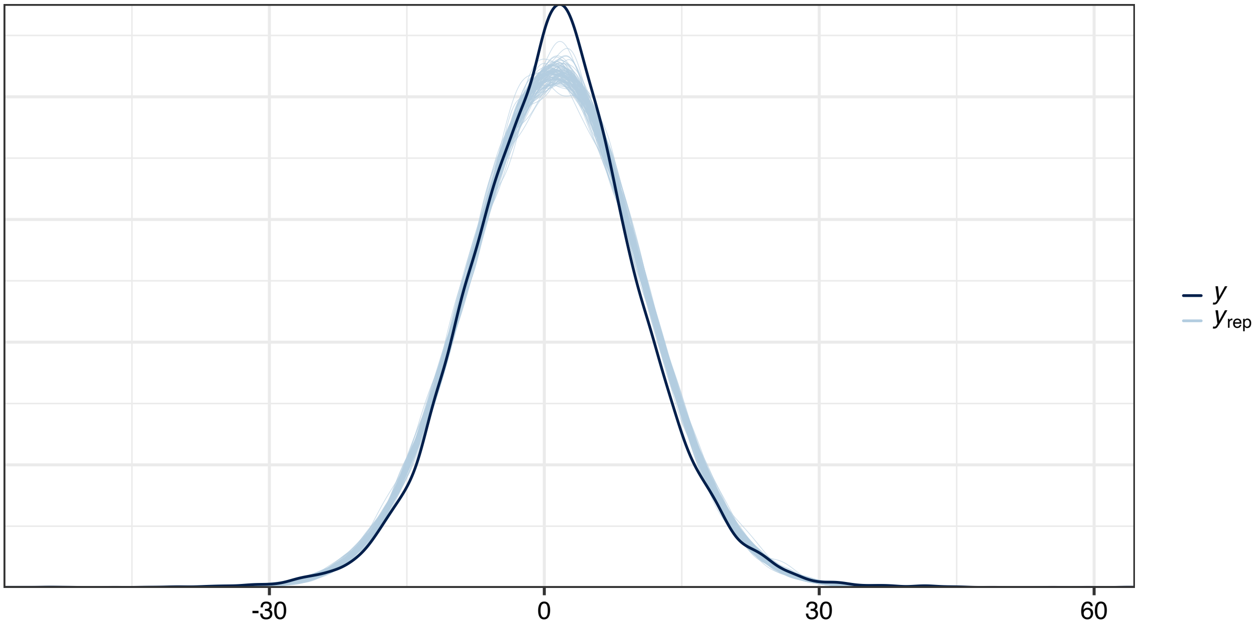

S3.1.4 Null model

```
if (!file.exists('../gams_fitted_models/gam_de_null.RDS')){
  prior.brms <-  c(
    prior(normal(0,1), class = b),
    prior(exponential(1), class = sigma)
  )
  model.null <-  brm(mV
                     ~ 1
                     + trial_number.Z
                     + t2(x, y, Participant, bs = "re")
                     + t2(x, y, Stimulus, bs = "re"),
                     prior = prior.brms,
                     sample_prior = TRUE,
                     data = german.data,
                     chains = 4, iter = 2000, cores = 4
  )
  saveRDS(model.null, '../gams_fitted_models/gam_de_null.RDS')
}
model.de.null <- readRDS('../gams_fitted_models/gam_de_null.RDS')
summary(model.de.null)
```

```
##  Family: gaussian
##   Links: mu = identity; sigma = identity
## Formula: mV ~ 1 + trial_number.Z + t2(x, y, Participant, bs = "re") + t2(x, y, Stimulus, bs = "re")
##    Data: german.data (Number of observations: 20961)
## Samples: 4 chains, each with iter = 2000; warmup = 1000; thin = 1;
##          total post-warmup samples = 4000
##
## Smooth Terms:
##                         Estimate Est.Error l-95% CI u-95% CI Rhat Bulk_ESS Tail_ESS
```

```
## sds(t2xyParticipant_1)     0.15      0.11     0.01     0.41 1.00     2126     2196
## sds(t2xyStimulus_1)        0.14      0.10     0.01     0.39 1.00     2274     2290
##
## Population-Level Effects:
##                Estimate Est.Error l-95% CI u-95% CI Rhat Bulk_ESS Tail_ESS
## Intercept          1.19      0.07     1.06     1.31 1.00     8112     3016
## trial_number.Z    -0.61      0.07    -0.74    -0.48 1.00     6337     2926
##
## Family Specific Parameters:
##       Estimate Est.Error l-95% CI u-95% CI Rhat Bulk_ESS Tail_ESS
## sigma     9.40      0.05     9.30     9.49 1.00     8667     2523
##
## Samples were drawn using sampling(NUTS). For each parameter, Bulk_ESS
## and Tail_ESS are effective sample size measures, and Rhat is the potential
## scale reduction factor on split chains (at convergence, Rhat = 1).
```

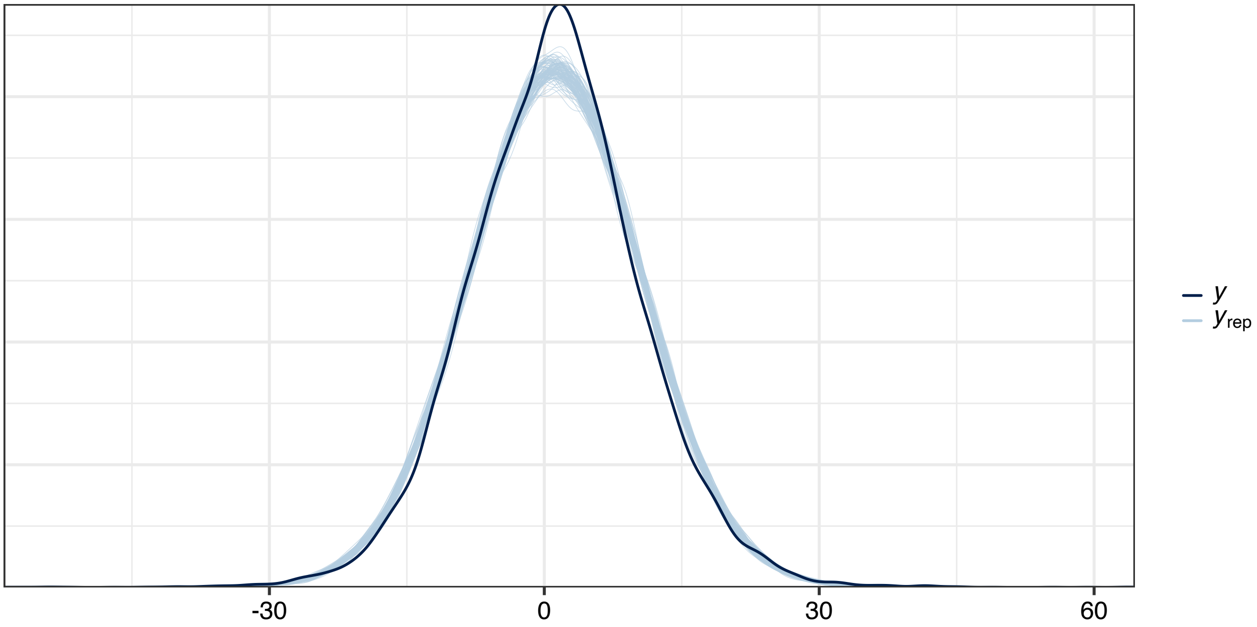

S3.2 Model comparison with stacking

Here we perform model comparison and report the results:

```
if (!file.exists('../temp_data/weights_brms_german_stacking.RDS')){
  loo.agent <- loo(model.de.agent)#, moment_match = T)
  loo.surplstm <- loo(model.de.surplstm)#, moment_match = T)
  loo.surptr <- loo(model.de.surptr)#, moment_match = T)
  loo.surpgpt <- loo(model.de.surpgpt)
  loo.agent.surplstm <- loo(model.de.agent.surplstm)#, moment_match = T)
  loo.agent.surptr <- loo(model.de.agent.surptr)#, moment_match = T)
  loo.agent.surpgpt <- loo(model.de.agent.surpgpt)#, moment_match = T)
  loo.null <- loo(model.de.null)#, moment_match = T)
  lweights <- loo_model_weights(list(
    "agent bias only"= loo.agent,
    "surprisal lstm" =  loo.surplstm,
```

```
    "surprisal bert" = loo.surptr,
    "surprisal lstm and agent bias" = loo.agent.surplstm,
    "surprisal bert and agent bias" = loo.agent.surptr,
    "null" = loo.null,
    "surprisal gpt" = loo.surpgpt,
    "surprisal gpt and agent bias" = loo.agent.surpgpt
  ))
  dfweights <- as.data.frame(as.matrix(lweights))
  saveRDS(dfweights,'../temp_data/weights_brms_german_stacking.RDS')
}
```

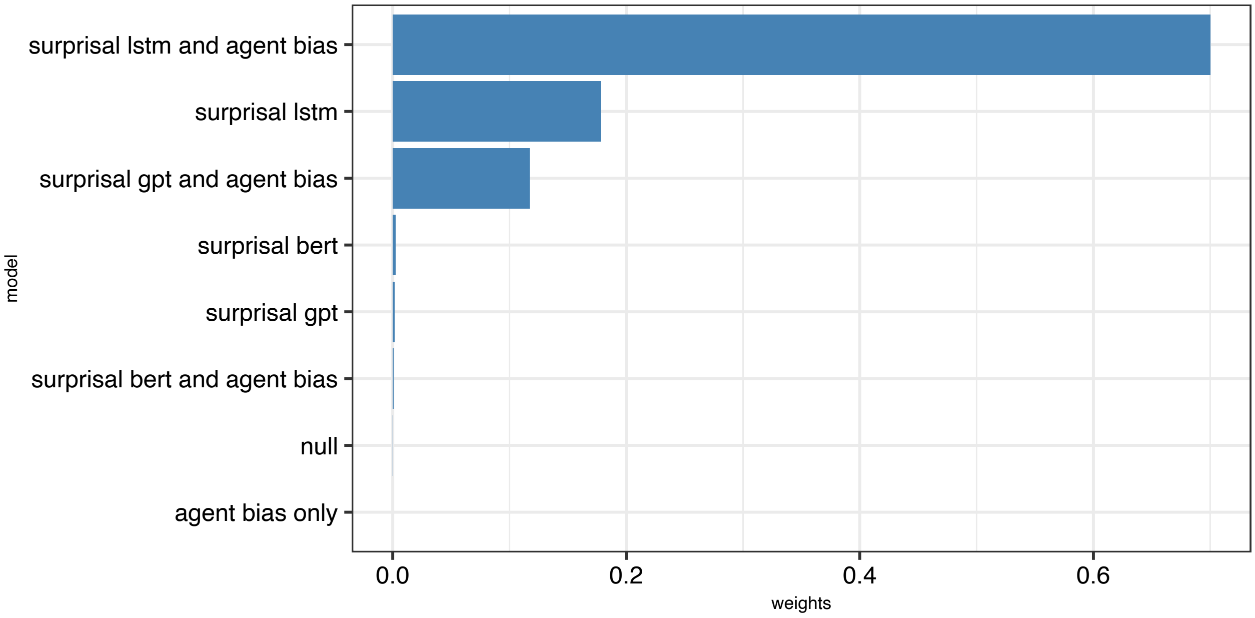

S3.3 Model predictions

In what follows we compute and plot the mean posterior predicted effects across the smoothed scalp surfaces. In each plot, the lef-hand side shows the difference between conditions. For surprisal we plot the difference between pedictions at +2 and at -2 standard deviations from the mean. Panels on the right-hand side quantify the evidence of the effect in terms of the proportion of the posterior distributions that is below or above 0.

S3.3.1 Agent preference alone

```
n_samps <- 4000
newdata.agent.de <- expand_grid(
  x = seq(min(german.data$x), max(german.data$x), length.out = 30),
  y = seq(min(german.data$y), max(german.data$y),length.out = 30),
  Participant = "01ax",
  Stimulus_Cond = c("1-3:3_Abias", "15-2:2_nonAbias"),
  trial_number.Z = 0,
)
```

```r
newdata.agent.de <- newdata.agent.de %>%
  separate(Stimulus_Cond, c('Stimulus', 'condition'), sep='_')

preds.agent.de <- posterior_epred(model.de.agent,
                                  newdata=newdata.agent.de, re.form=NA)

preds.agent.de_long <- as.data.frame(preds.agent.de) %>%
  mutate(samp = 1:nrow(preds.agent.de)) %>%
  pivot_longer(-samp)

preds.agent.de_long$x <- rep(newdata.agent.de$x, n_samps)
preds.agent.de_long$y <- rep(newdata.agent.de$y, n_samps)
preds.agent.de_long$condition <- rep(newdata.agent.de$condition, n_samps)

preds.agent.de_long$x <- (-(preds.agent.de_long$x))

preds.agent.de_summary <- preds.agent.de_long %>%
  group_by(x,y,samp) %>%
  mutate(diff_cond = value[condition == 'Abias'] -
           value[condition == 'nonAbias']) %>%
  ungroup() %>%
  group_by(x,y) %>%
  summarise(PP =
              ifelse(mean(diff_cond) > 0, mean(diff_cond > 0),
                     mean(diff_cond < 0)),
            mean_diff = mean(diff_cond))

plot.agent.de <- preds.agent.de_summary %>%
  mutate(sig = ifelse(PP>0.80, 1, 0)) %>%
  ggplot(aes(x=x, y=y, fill=mean_diff, alpha = sig)) +
  geom_tile()+
  scale_fill_gradient2(low = '#0053d3', high = '#b22709', midpoint=0,
                       aesthetics='fill')+
  labs(fill=(expression(paste(Delta, paste(mu, "V")))),
       x=element_blank(),
       y=element_blank()) +
  guides(alpha = "none") +
  theme(panel.grid = element_blank(), panel.border = element_blank(),
        strip.background = element_blank(),strip.text.x = element_text(size=5),
        axis.text.y=element_blank(),
        axis.text.x = element_blank(),
        axis.ticks.y=element_blank(),
        axis.ticks.x=element_blank(),
        plot.background = element_rect(fill = "transparent", colour = NA))

plot.agent.de.pp <- preds.agent.de_summary %>%
  ggplot(aes(x=x, y=y, fill=PP)) +
  geom_tile()+
  scale_fill_gradientn(colours = c('white','grey', 'black'),limits = c(0.8, 1),
                       na.value='white' ) +
  labs(fill=(expression(paste('P(|',Delta,mu, V, '| > 0)', ))),
       x='electrode coordinate x',
       y='electrode coordinate y') +
```

```
  theme(panel.grid = element_blank(),
        panel.border = element_blank(),
        strip.background = element_blank(),
        axis.ticks.x = element_blank(),
        axis.ticks.y=element_blank(),
        axis.text.x=element_blank(),
        axis.text.y = element_blank(),
        plot.background = element_rect(fill = "transparent", colour = NA),
        title = element_blank())
```

```
wrap_plots(list(plot.agent.de, plot.agent.de.pp))
```

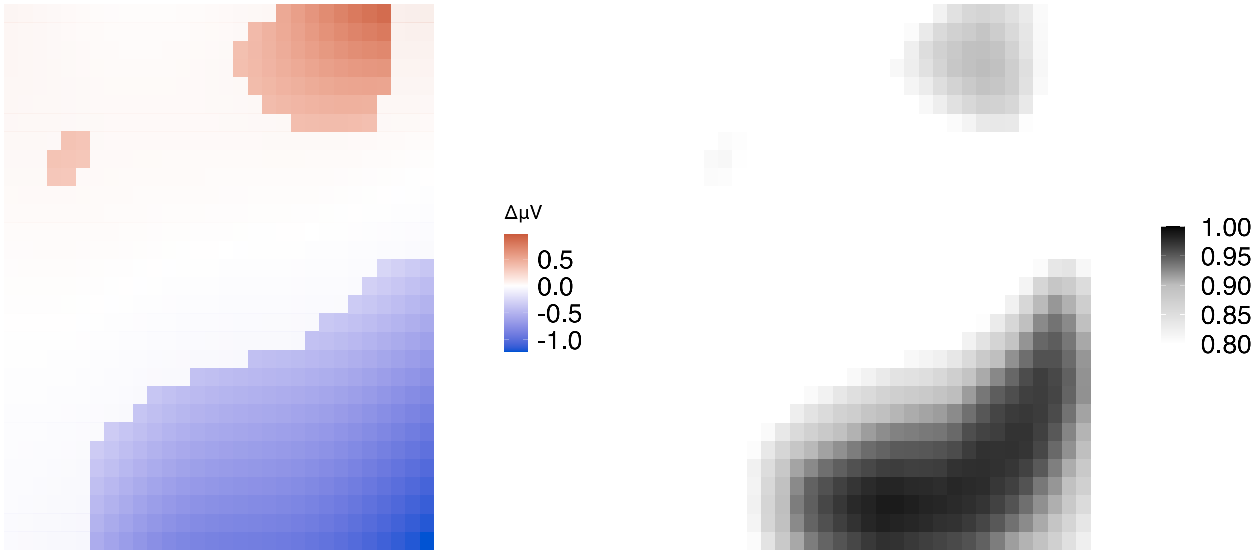

Figure S1: Left: Pair-wise fitted differences of micro-volt for sentences with vs. sentences without the predicted reanalysis from model with agent preference predictor alone. Right: Percentage of posterior probability mass of micro-volt mean difference below or above 0.

S3.3.2 LSTM-based surprisal alone

```
newdata.de.surplstm <- expand_grid(
  x = seq(min(german.data$x), max(german.data$x), length.out = 30),
  y = seq(min(german.data$y), max(german.data$y),length.out = 30),
  surprisal.lstm.Z = seq(from=-2,to=2, length.out=17),
  Participant = "01ax",
  Stimulus = c("1-3:3"),
  trial_number.Z = 0,
)

preds.de.surplstm <- posterior_epred(model.de.surplstm,
                                     newdata=newdata.de.surplstm , re.form=NA)
preds.de.surplstm_long <- as.data.frame(preds.de.surplstm) %>%
  mutate(samp = 1:nrow(preds.de.surplstm)) %>%
  pivot_longer(-samp)
```

```r
preds.de.surplstm_long$x <- rep(newdata.de.surplstm$x, n_samps)
preds.de.surplstm_long$y <- rep(newdata.de.surplstm$y, n_samps)
preds.de.surplstm_long$surprisal <- rep(newdata.de.surplstm$surprisal.lstm.Z,
                                        n_samps)

summary_de_surplstm <- preds.de.surplstm_long %>%
  group_by(x,y,samp) %>%
  mutate(diff_surprisal = value[surprisal == 2] - value[surprisal == -2]) %>%
  ungroup() %>%
  group_by(x,y) %>%
  summarise(PP = ifelse(mean(diff_surprisal) > 0, mean(diff_surprisal > 0),
                        mean(diff_surprisal < 0)),
            mean_diff = mean(diff_surprisal)) %>%
  mutate(x = -x)

plot.lstm.de <- summary_de_surplstm %>%
  mutate(sig = ifelse(PP>0.80, 1, 0)) %>%
  ggplot(aes(x=x, y=y, fill=mean_diff, alpha = sig)) +
  geom_tile()+
  scale_fill_gradient2(low = '#0053d3', high = '#b22709', midpoint=0,
                       aesthetics='fill')+
  labs(fill=(expression(paste(Delta, paste(mu, "V")))),
       x=element_blank(),
       y=element_blank()) +
  guides(alpha = "none") +
  theme(panel.grid = element_blank(), panel.border = element_blank(),
        strip.background = element_blank(),strip.text.x = element_text(size=5),
        axis.text.y=element_blank(),
        axis.text.x = element_blank(),
        axis.ticks.y=element_blank(),
        axis.ticks.x=element_blank(),
        plot.background = element_rect(fill = "transparent", colour = NA))

plot.lstm.de.pp <- summary_de_surplstm %>%
  ggplot(aes(x=x, y=y, fill=PP)) +
  geom_tile()+
  scale_fill_gradientn(colours = c('white','grey', 'black'),limits = c(0.8, 1),
                       na.value='white' ) +
  labs(fill=(expression(paste('P(|',Delta,mu, V, '| > 0)', ))),
       x=element_blank(),
       y=element_blank()) +
  theme(panel.grid = element_blank(), panel.border = element_blank(),
        strip.background = element_blank(),strip.text.x = element_text(size=5),
        axis.text.y=element_blank(),
        axis.ticks.y=element_blank(),
        plot.background = element_rect(fill = "transparent", colour = NA))
```

```r
wrap_plots(list(plot.lstm.de, plot.lstm.de.pp))
```

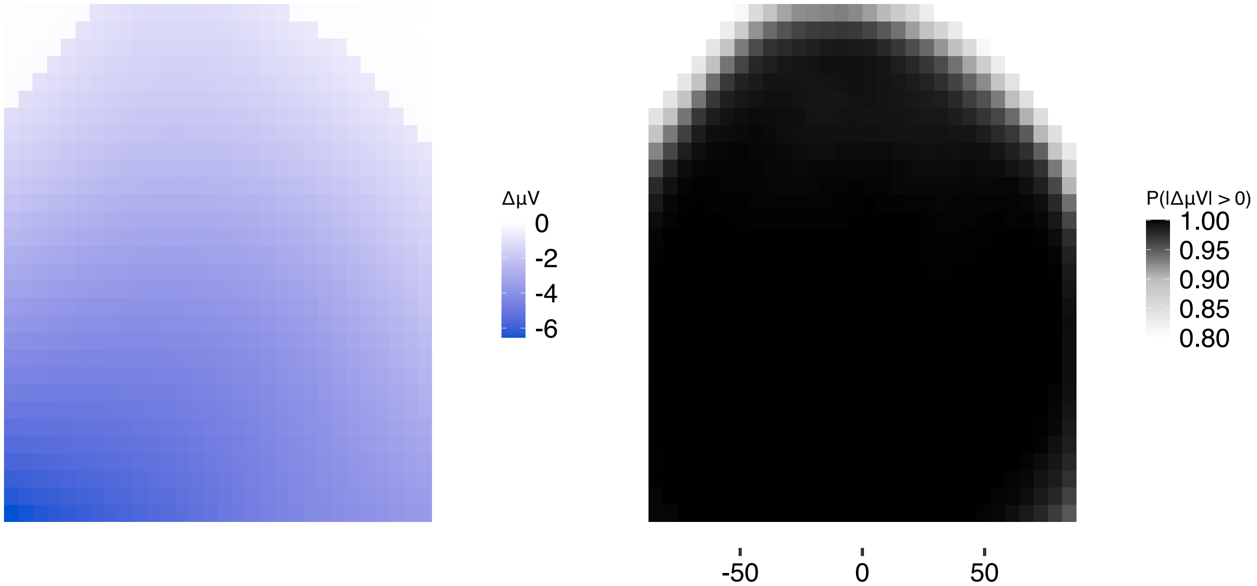

Figure S2: Left: Pair-wise fitted differences of micro-volt for sentences with high vs. sentences with low (+2 vs. -2 std. dev. from the mean) from model with LSTM surprisal as predictor alone. Right: Percentage of posterior probability mass of micro-volt mean difference below or above 0.

S3.3.3 BERT-based surprisal alone

```
newdata.de.surptr <- dplyr::rename(newdata.de.surplstm,
                                   surprisal.tr.Z = surprisal.lstm.Z)
preds.de.surptr <- posterior_epred(model.de.surptr,
                                   newdata=newdata.de.surptr, re.form=NA)
preds.de.surptr_long <- as.data.frame(preds.de.surptr) %>%
  mutate(samp = 1:nrow(preds.de.surptr)) %>%
  pivot_longer(-samp)

preds.de.surptr_long$x <- rep(newdata.de.surptr$x, n_samps)
preds.de.surptr_long$y <- rep(newdata.de.surptr$y, n_samps)
preds.de.surptr_long$surprisal <- rep(newdata.de.surptr$surprisal.tr.Z, n_samps)

summary_de_surptr <- preds.de.surptr_long %>%
  group_by(x,y,samp) %>%
  mutate(diff_surprisal_low_mean = value[surprisal == 2] -
           value[surprisal == -2]) %>%
  ungroup() %>%
  group_by(x,y) %>%
  summarise(PP = ifelse(mean(diff_surprisal_low_mean) > 0,
                        mean(diff_surprisal_low_mean > 0),
                        mean(diff_surprisal_low_mean < 0)),
            mean_diff = mean(diff_surprisal_low_mean))  %>%
  mutate(x = -x)

plot.tr.de <- summary_de_surptr %>%
  mutate(sig = ifelse(PP>0.80, 1, 0)) %>%
  ggplot(aes(x=x, y=y, fill=mean_diff, alpha = sig)) +
```

```r
  geom_tile()+
  scale_fill_gradient2(low = '#0053d3', high = '#b22709', midpoint=0,
                       aesthetics='fill')+
  labs(fill=(expression(paste(Delta, paste(mu, "V")))),
       x=element_blank(),
       y=element_blank()) +
  guides(alpha = "none") +
  theme(panel.grid = element_blank(), panel.border = element_blank(),
        strip.background = element_blank(),strip.text.x = element_text(size=5),
        axis.text.y=element_blank(),
        axis.text.x = element_blank(),
        axis.ticks.y=element_blank(),
        axis.ticks.x=element_blank(),
        plot.background = element_rect(fill = "transparent", colour = NA))

plot.tr.de.pp <- summary_de_surptr %>%
  ggplot(aes(x=x, y=y, fill=PP)) +
  geom_tile()+
  scale_fill_gradientn(colours = c('white','grey', 'black'),limits = c(0.8, 1),
                       na.value='white' ) +
  labs(fill=(expression(paste('P(|',Delta,mu, V, '| > 0)', ))),
       x=element_blank(),
       y=element_blank()) +
  theme(panel.grid = element_blank(), panel.border = element_blank(),
        strip.background = element_blank(),strip.text.x = element_text(size=5),
        axis.text.y=element_blank(),
        axis.ticks.y=element_blank(),
        plot.background = element_rect(fill = "transparent", colour = NA))
```

```r
wrap_plots(list(plot.tr.de, plot.tr.de.pp))
```

S3.3.4 GPT-based surprisal alone

```r
newdata.de.surpgpt <- dplyr::rename(newdata.de.surplstm,
                                    surprisal.gpt.Z = surprisal.lstm.Z)
preds.de.surpgpt <- posterior_epred(model.de.surpgpt,
                                    newdata=newdata.de.surpgpt, re.form=NA)
preds.de.surpgpt_long <- as.data.frame(preds.de.surpgpt) %>%
  mutate(samp = 1:nrow(preds.de.surpgpt)) %>%
  pivot_longer(-samp)

preds.de.surpgpt_long$x <- rep(newdata.de.surpgpt$x, n_samps)
preds.de.surpgpt_long$y <- rep(newdata.de.surpgpt$y, n_samps)
preds.de.surpgpt_long$surprisal <- rep(newdata.de.surpgpt$surprisal.gpt.Z, n_samps)

summary_de_surpgpt <- preds.de.surpgpt_long %>%
  group_by(x,y,samp) %>%
  mutate(diff_surprisal_low_mean = value[surprisal == 2] -
           value[surprisal == -2]) %>%
  ungroup() %>%
```

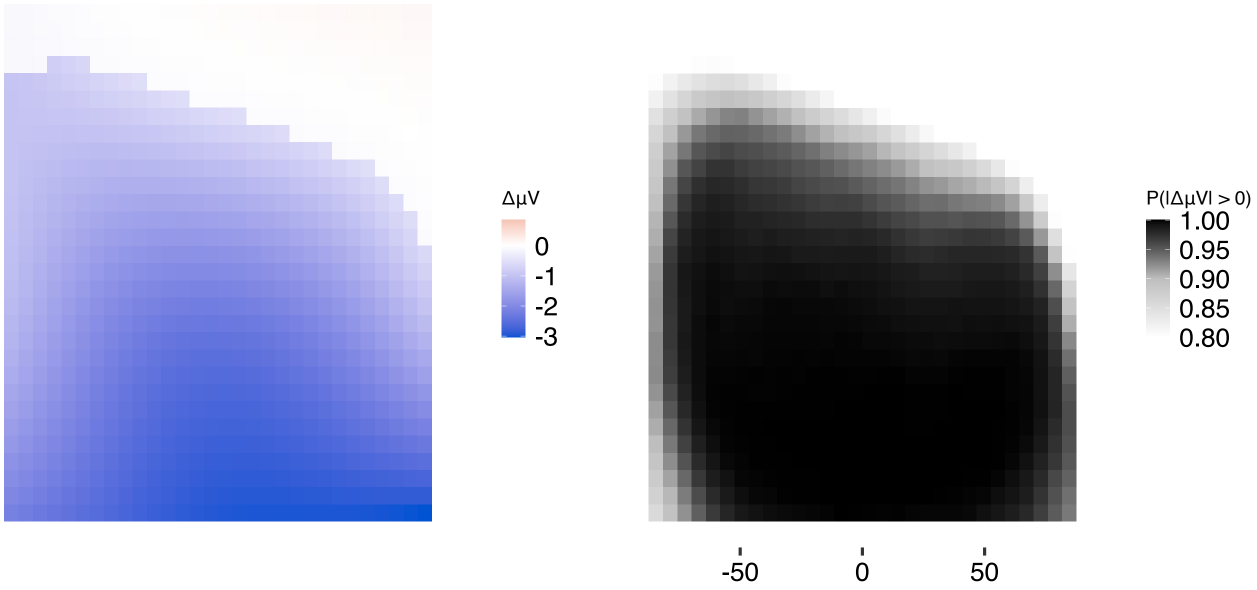

Figure S3: Left: Pair-wise fitted differences of micro-volt for sentences with high vs. sentences with low (+2 vs. -2 std. dev. from the mean) from model with BERT surprisal as predictor alone. Right: Percentage of posterior probability mass of micro-volt mean difference below or above 0.

```
  group_by(x,y) %>%
  summarise(PP = ifelse(mean(diff_surprisal_low_mean) > 0,
                        mean(diff_surprisal_low_mean > 0),
                        mean(diff_surprisal_low_mean < 0)),
            mean_diff = mean(diff_surprisal_low_mean))  %>%
  mutate(x = -x)

plot.gpt.de <- summary_de_surpgpt %>%
  mutate(sig = ifelse(PP>0.80, 1, 0)) %>%
  ggplot(aes(x=x, y=y, fill=mean_diff, alpha = sig)) +
  geom_tile()+
  scale_fill_gradient2(low = '#0053d3', high = '#b22709', midpoint=0,
                       aesthetics='fill')+
  labs(fill=(expression(paste(Delta, paste(mu, "V")))),
       x=element_blank(),
       y=element_blank()) +
  guides(alpha = "none") +
  theme(panel.grid = element_blank(), panel.border = element_blank(),
        strip.background = element_blank(), strip.text.x = element_text(size=5),
        axis.text.y=element_blank(),
        axis.text.x = element_blank(),
        axis.ticks.y=element_blank(),
        axis.ticks.x=element_blank(),
        plot.background = element_rect(fill = "transparent", colour = NA))

plot.gpt.de.pp <- summary_de_surpgpt %>%
  ggplot(aes(x=x, y=y, fill=PP)) +
  geom_tile()+
  scale_fill_gradientn(colours = c('white','grey', 'black'),limits = c(0.8, 1),
                       na.value='white' ) +
```

```
  labs(fill=(expression(paste('P(|',Delta,mu, V, '| > 0)', ))),
       x=element_blank(),
       y=element_blank()) +
  theme(panel.grid = element_blank(), panel.border = element_blank(),
        strip.background = element_blank(),strip.text.x = element_text(size=5),
        axis.text.y=element_blank(),
        axis.ticks.y=element_blank(),
        plot.background = element_rect(fill = "transparent", colour = NA))
```

```
wrap_plots(list(plot.gpt.de, plot.gpt.de.pp))
```

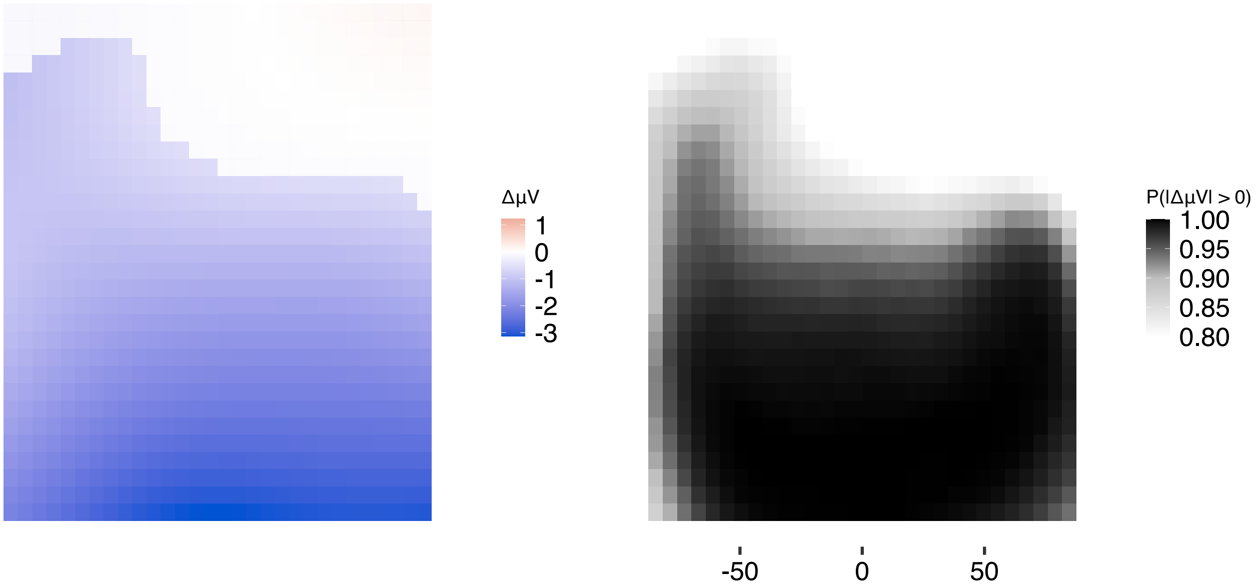

Figure S4: Left: Pair-wise fitted differences of micro-volt for sentences with high vs. sentences with low (+2 vs. -2 std. dev. from the mean) from model with GPT-2 surprisal as predictor alone. Right: Percentage of posterior probability mass of micro-volt mean difference below or above 0.

S3.3.5 LSTM-based surprisal and Agent Preference

```
n_samps <- 4000
newdata.de.agent.surplstm <- expand_grid(
  x = seq(min(german.data$x), max(german.data$x), length.out = 30),
  y = seq(min(german.data$y), max(german.data$y),length.out = 30),
  surprisal.lstm.Z = seq(from=-2,to=2, length.out=17),
  Participant = "01ax",
  Stimulus_Cond = c("1-3:3_Abias", "15-2:2_nonAbias"),
  trial_number.Z = 0,
)

newdata.de.agent.surplstm <- newdata.de.agent.surplstm  %>%
  separate(Stimulus_Cond, c('Stimulus', 'condition'), sep='_')
```

```r
preds.de.agent.surplstm <- posterior_epred(model.de.agent.surplstm,
                                           newdata=newdata.de.agent.surplstm,
                                           re.form=NA)

preds.de.agent.surplstm_long <- as.data.frame(preds.de.agent.surplstm) %>%
  mutate(samp = 1:nrow(preds.de.agent.surplstm)) %>%
  pivot_longer(-samp)

preds.de.agent.surplstm_long$x <-
  rep(newdata.de.agent.surplstm$x, n_samps)
preds.de.agent.surplstm_long$y <-
  rep(newdata.de.agent.surplstm$y, n_samps)
preds.de.agent.surplstm_long$surprisal <-
  rep(newdata.de.agent.surplstm$surprisal.lstm.Z, n_samps)
preds.de.agent.surplstm_long$condition <-
  rep(newdata.de.agent.surplstm$condition, n_samps)

summary_de_agent_surplstm <- preds.de.agent.surplstm_long %>%
  filter(condition %in% 'Abias') %>%
  group_by(x,y,samp) %>%
  mutate(diff_surprisal_low_mean = value[surprisal == 2] -
           value[surprisal == -2]) %>%
  ungroup() %>%
  group_by(x,y) %>%
  summarise(PP = ifelse(mean(diff_surprisal_low_mean) > 0,
                        mean(diff_surprisal_low_mean > 0),
                        mean(diff_surprisal_low_mean < 0)),
            mean_diff = mean(diff_surprisal_low_mean)) %>%
  mutate(condition = 'Abias') %>%
  mutate(x = -x)

plot.lstm.agent.surp.de <- summary_de_agent_surplstm %>%
  mutate(sig = ifelse(PP>0.80, 1, 0)) %>%
  ggplot(aes(x=x, y=y, fill=mean_diff, alpha = sig)) +
  geom_tile()+
  scale_fill_gradient2(low = '#0053d3', high = '#b22709', midpoint=0, aesthetics='fill')+
  guides(alpha = "none") +
  labs(fill=(expression(paste(Delta, paste(mu, "V")))),
       x=element_blank(),
       y=element_blank()) +
  theme(panel.grid = element_blank(), panel.border = element_blank(),
        strip.background = element_blank(),strip.text.x = element_text(size=5),
        axis.text.y=element_blank(),
        axis.text.x=element_blank(),
        axis.ticks.y=element_blank(),
        axis.ticks.x=element_blank(),
        plot.background = element_rect(fill = "transparent", colour = NA))

plot.lstm.agent.surp.de.pp <- summary_de_agent_surplstm %>%
  ggplot(aes(x=x, y=y, fill=PP)) +
  geom_tile()+
  scale_fill_gradientn(colours = c('white','grey', 'black'),limits = c(0.8, 1),
                       na.value='white' ) +
```

```
  labs(fill=(expression(paste('P(|',Delta,mu, V, '| > 0)', ))),
       x=element_blank(),
       y=element_blank()) +
  theme(panel.grid = element_blank(), panel.border = element_blank(),
        strip.background = element_blank(),strip.text.x = element_text(size=5),
        axis.text.y=element_blank(),
        axis.text.x=element_blank(),
        axis.ticks.y=element_blank(),
        axis.ticks.x=element_blank(),
        plot.background = element_rect(fill = "transparent", colour = NA))

summary_de_agent_surplstm.conds <- preds.de.agent.surplstm_long %>%
  filter(surprisal == 0) %>%
  group_by(x,y,samp) %>%
  mutate(diff_condition = value[condition == 'Abias'] -
           value[condition == 'nonAbias']) %>%
  ungroup() %>%
  group_by(x,y) %>%
  summarise(PP = ifelse(mean(diff_condition) > 0,
                        mean(diff_condition > 0),
                        mean(diff_condition < 0)),
            mean_diff = mean(diff_condition)) %>%
  mutate(x = -x)

plot.lstm.agent.de.cond <- summary_de_agent_surplstm.conds  %>%
  mutate(sig = ifelse(PP>0.80, 1, 0)) %>%
  ggplot(aes(x=x, y=y, fill=mean_diff, alpha = sig)) +
  geom_tile()+
  scale_fill_gradient2(low = '#0053d3', high = '#b22709', midpoint=0,
                       aesthetics='fill')+
  labs(fill=(expression(paste(Delta, paste(mu, "V")))),
       x=element_blank(),
       y=element_blank()) +
  guides(alpha = "none") +
  theme(panel.grid = element_blank(), panel.border = element_blank(),
        strip.background = element_blank(),strip.text.x = element_text(size=5),
        axis.text.y=element_blank(),
        axis.text.x = element_blank(),
        axis.ticks.y=element_blank(),
        axis.ticks.x=element_blank(),
        plot.background = element_rect(fill = "transparent", colour = NA))

plot.lstm.agent.de.cond.pp <- summary_de_agent_surplstm.conds  %>%
  ggplot(aes(x=x, y=y, fill=PP)) +
  geom_tile()+
  scale_fill_gradientn(colours = c('white','grey', 'black'),limits = c(0.8, 1),
                       na.value='white' ) +
  labs(fill=(expression(paste('P(|',Delta,mu, V, '| > 0)', ))),
       x=element_blank(),
       y=element_blank()) +
  guides(alpha = "none") +
  theme(panel.grid = element_blank(), panel.border = element_blank(),
        strip.background = element_blank(),strip.text.x = element_text(size=5),
```

```r
        axis.text.y=element_blank(),
        axis.text.x=element_blank(),
        axis.ticks.x=element_blank(),
        axis.ticks.y=element_blank(),
        plot.background = element_rect(fill = "transparent", colour = NA))
```

```r
# pdf('../plots/german_fitted_plot.pdf', width=10, height=7.5)
wrap_plots(list(plot.lstm.agent.de.cond, plot.lstm.agent.surp.de,
                plot.lstm.agent.de.cond.pp, plot.lstm.agent.surp.de.pp))
```

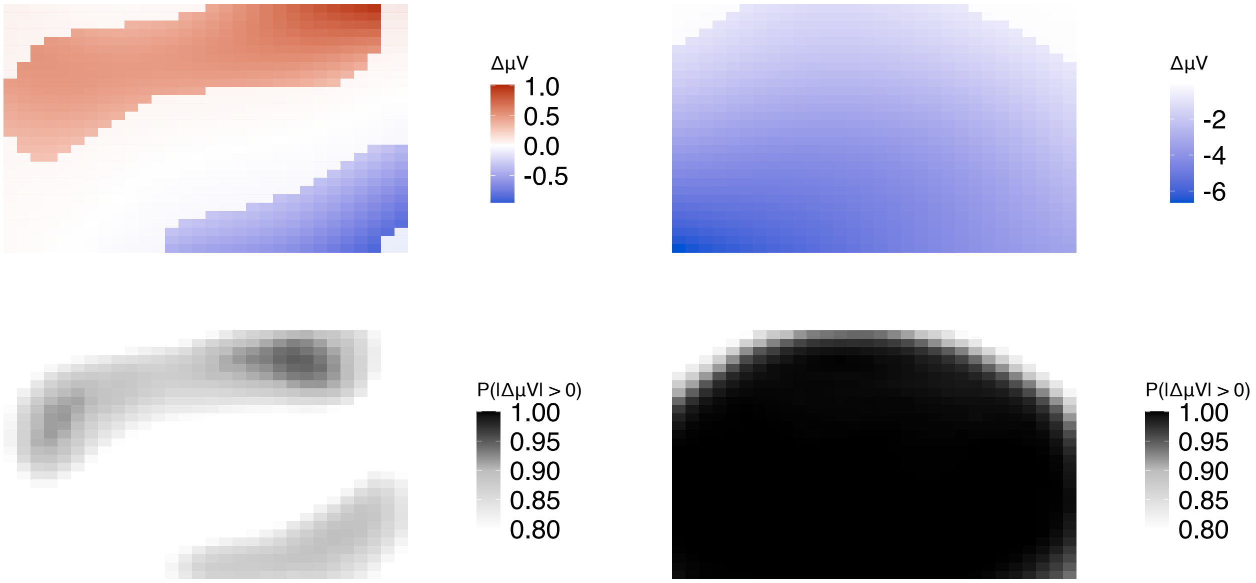

Figure S5: Left: Pair-wise fitted differences of micro-volt for sentences with vs. sentences without the predicted reanalysis from model with LSTM surprisal as well as agent preference predictor. Right: Percentage of posterior probability mass of micro-volt mean difference below or above 0.

```r
# dev.off()
```

S3.3.6 BERT-based surprisal and Agent Preference

```r
newdata.de.agent.surptr <- newdata.de.agent.surplstm %>%
  dplyr::rename(surprisal.tr.Z =  surprisal.lstm.Z)
preds.de.agent.surptr <- posterior_epred(model.de.agent.surptr,
                                         newdata=newdata.de.agent.surptr,
                                         re.form=NA)

preds.de.agent.surptr_long <- as.data.frame(preds.de.agent.surptr) %>%
  mutate(samp = 1:nrow(preds.de.agent.surptr)) %>%
  pivot_longer(-samp)

preds.de.agent.surptr_long$x <- rep(newdata.de.agent.surptr$x, n_samps)
preds.de.agent.surptr_long$y <- rep(newdata.de.agent.surptr$y, n_samps)
preds.de.agent.surptr_long$surprisal <- rep(newdata.de.agent.surptr$surprisal.tr.Z,
```

```
                                                            n_samps)
preds.de.agent.surptr_long$condition <- rep(newdata.de.agent.surptr$condition,
                                                            n_samps)

summary_de_agent_surptr.abias <- preds.de.agent.surptr_long %>%
  filter(condition %in% 'Abias') %>%
  group_by(x,y,samp) %>%
  mutate(diff_surprisal_low_mean = value[surprisal == 2] -
           value[surprisal == -2]) %>%
  ungroup() %>%
  group_by(x,y) %>%
  summarise(PP = ifelse(mean(diff_surprisal_low_mean) > 0,
                        mean(diff_surprisal_low_mean > 0),
                        mean(diff_surprisal_low_mean < 0)),
            mean_diff = mean(diff_surprisal_low_mean)) %>%
  mutate('condition' = 'Abias')

summary_de_agent_surptr.nonabias <- preds.de.agent.surptr_long %>%
  filter(condition %in% 'Abias') %>%
  group_by(x,y,samp) %>%
  mutate(diff_surprisal_low_mean = value[surprisal == 2] -
           value[surprisal == -2]) %>%
  ungroup() %>%
  group_by(x,y) %>%
  summarise(PP = ifelse(mean(diff_surprisal_low_mean) > 0,
                        mean(diff_surprisal_low_mean > 0),
                        mean(diff_surprisal_low_mean < 0)),
            mean_diff = mean(diff_surprisal_low_mean)) %>%
  mutate('condition' = 'nonAbias')

summary_de_agent_surptr <- rbind(summary_de_agent_surptr.nonabias,
                                 summary_de_agent_surptr.abias)  %>%
  mutate(x = -x)

plot.tr.agent.surp.de <- summary_de_agent_surptr %>%
  ggplot(aes(x=x, y=y, fill=mean_diff, alpha = PP)) +
  geom_tile()+
  scale_fill_gradient2(low = '#0053d3', high = '#b22709', midpoint=0,
                       aesthetics='fill')+
  labs(fill=(expression(paste(Delta, paste(mu, "V")))),
       x=element_blank(),
       y=element_blank()) +
  guides(alpha = "none") +
  theme(panel.grid = element_blank(), panel.border = element_blank(),
        strip.background = element_blank(),strip.text.x = element_text(size=5),
        axis.text.y=element_blank(),
        axis.text.x = element_blank(),
        axis.ticks.y=element_blank(),
        axis.ticks.x=element_blank(),
        plot.background = element_rect(fill = "transparent", colour = NA))

plot.tr.agent.surp.de.pp <- summary_de_agent_surptr %>%
  ggplot(aes(x=x, y=y, fill=PP)) +
```

```
  geom_tile()+
  scale_fill_gradientn(colours = c('white','grey', 'black'),limits = c(0.8, 1),
                       na.value='white' ) +
  labs(fill=(expression(paste('P(|',Delta,mu, V, '| > 0)', ))),
       x=element_blank(),
       y=element_blank()) +
  guides(alpha = "none") +
  theme(panel.grid = element_blank(), panel.border = element_blank(),
        strip.background = element_blank(),strip.text.x = element_text(size=5),
        axis.text.y=element_blank(),
        axis.ticks.y=element_blank(),
        plot.background = element_rect(fill = "transparent", colour = NA))

summary_de_agent_surptr.condition <- preds.de.agent.surptr_long %>%
  filter(surprisal == 0) %>%
  group_by(x,y,samp) %>%
  mutate(diff_condition = value[condition == 'Abias'] -
           value[condition == 'nonAbias']) %>%
  ungroup() %>%
  group_by(x,y) %>%
  summarise(PP = ifelse(mean(diff_condition) > 0,
                        mean(diff_condition > 0),
                        mean(diff_condition < 0)),
            mean_diff = mean(diff_condition))  %>%
  mutate(x = -x)

plot.tr.agent.de.cond <- summary_de_agent_surptr.condition %>%
  mutate(sig = ifelse(PP>0.80, 1, 0)) %>%
  ggplot(aes(x=x, y=y, fill=mean_diff, alpha = sig)) +
  geom_tile()+
  scale_fill_gradient2(low = '#0053d3', high = '#b22709', midpoint=0,
                       aesthetics='fill')+
  labs(fill=(expression(paste(Delta, paste(mu, "V")))),
       x=element_blank(),
       y=element_blank()) +
  guides(alpha = "none") +
  theme(panel.grid = element_blank(), panel.border = element_blank(),
        strip.background = element_blank(),strip.text.x = element_text(size=5),
        axis.text.y=element_blank(),
        axis.text.x = element_blank(),
        axis.ticks.y=element_blank(),
        axis.ticks.x=element_blank(),
        plot.background = element_rect(fill = "transparent", colour = NA))

plot.tr.agent.de.cond.pp <- summary_de_agent_surptr.condition %>%
  ggplot(aes(x=x, y=y, fill=PP)) +
  geom_tile()+
  scale_fill_gradientn(colours = c('white','grey', 'black'),limits = c(0.8, 1),
                       na.value='white' ) +
  labs(fill=(expression(paste('P(|',Delta,mu, V, '| > 0)', ))),
       x=element_blank(),
       y=element_blank()) +
  guides(alpha = "none") +
```

```r
  theme(panel.grid = element_blank(), panel.border = element_blank(),
        strip.background = element_blank(),strip.text.x = element_text(size=5),
        axis.text.y=element_blank(),
        axis.ticks.y=element_blank(),
        plot.background = element_rect(fill = "transparent", colour = NA))
```

```r
wrap_plots(list(plot.tr.agent.de.cond, plot.tr.agent.surp.de,
                plot.tr.agent.de.cond.pp, plot.tr.agent.surp.de.pp))
```

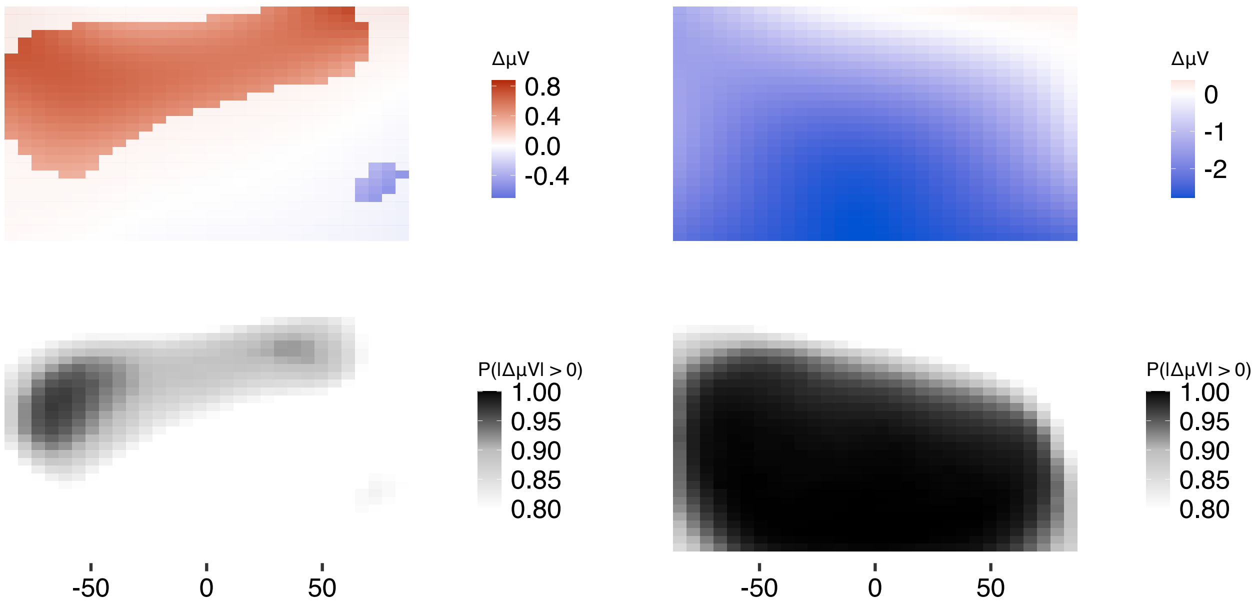

Figure S6: Left: Pair-wise fitted differences of micro-volt for sentences with vs. sentences without the predicted reanalysis from model with BERT surprisal as well as agent preference predictor. Right: Percentage of posterior probability mass of micro-volt mean difference below or above 0.

S3.3.7 GPT-based surprisal and Agent Preference

```r
newdata.de.agent.surpgpt <- newdata.de.agent.surplstm %>%
  dplyr::rename(surprisal.gpt.Z =  surprisal.lstm.Z)
preds.de.agent.surpgpt <- posterior_epred(model.de.agent.surpgpt,
                                          newdata=newdata.de.agent.surpgpt,
                                          re.form=NA)

preds.de.agent.surpgpt_long <- as.data.frame(preds.de.agent.surpgpt) %>%
  mutate(samp = 1:nrow(preds.de.agent.surpgpt)) %>%
  pivot_longer(-samp)

preds.de.agent.surpgpt_long$x <- rep(newdata.de.agent.surpgpt$x, n_samps)
preds.de.agent.surpgpt_long$y <- rep(newdata.de.agent.surpgpt$y, n_samps)
preds.de.agent.surpgpt_long$surprisal <- rep(newdata.de.agent.surpgpt$surprisal.gpt.Z,
                                             n_samps)
preds.de.agent.surpgpt_long$condition <- rep(newdata.de.agent.surpgpt$condition,
                                             n_samps)
```

```
summary_de_agent_surpgpt.abias <- preds.de.agent.surpgpt_long %>%
  filter(condition %in% 'Abias') %>%
  group_by(x,y,samp) %>%
  mutate(diff_surprisal_low_mean = value[surprisal == 2] -
           value[surprisal == -2]) %>%
  ungroup() %>%
  group_by(x,y) %>%
  summarise(PP = ifelse(mean(diff_surprisal_low_mean) > 0,
                        mean(diff_surprisal_low_mean > 0),
                        mean(diff_surprisal_low_mean < 0)),
            mean_diff = mean(diff_surprisal_low_mean)) %>%
  mutate('condition' = 'Abias')

summary_de_agent_surpgpt.nonabias <- preds.de.agent.surpgpt_long %>%
  filter(condition %in% 'Abias') %>%
  group_by(x,y,samp) %>%
  mutate(diff_surprisal_low_mean = value[surprisal == 2] -
           value[surprisal == -2]) %>%
  ungroup() %>%
  group_by(x,y) %>%
  summarise(PP = ifelse(mean(diff_surprisal_low_mean) > 0,
                        mean(diff_surprisal_low_mean > 0),
                        mean(diff_surprisal_low_mean < 0)),
            mean_diff = mean(diff_surprisal_low_mean)) %>%
  mutate('condition' = 'nonAbias')

summary_de_agent_surpgpt <- rbind(summary_de_agent_surpgpt.nonabias,
                                  summary_de_agent_surpgpt.abias)  %>%
  mutate(x = -x)

plot.gpt.agent.surp.de <- summary_de_agent_surpgpt %>%
  ggplot(aes(x=x, y=y, fill=mean_diff, alpha = PP)) +
  geom_tile()+
  scale_fill_gradient2(low = '#0053d3', high = '#b22709', midpoint=0,
                       aesthetics='fill')+
  labs(fill=(expression(paste(Delta, paste(mu, "V")))),
       x=element_blank(),
       y=element_blank()) +
  guides(alpha = "none") +
  theme(panel.grid = element_blank(), panel.border = element_blank(),
        strip.background = element_blank(),strip.text.x = element_text(size=5),
        axis.text.y=element_blank(),
        axis.text.x = element_blank(),
        axis.ticks.y=element_blank(),
        axis.ticks.x=element_blank(),
        plot.background = element_rect(fill = "transparent", colour = NA))

plot.gpt.agent.surp.de.pp <- summary_de_agent_surpgpt %>%
  ggplot(aes(x=x, y=y, fill=PP)) +
  geom_tile()+
  scale_fill_gradientn(colours = c('white','grey', 'black'),limits = c(0.8, 1),
                       na.value='white' ) +
  labs(fill=(expression(paste('P(|',Delta,mu, V, '| > 0)', ))),
```

```
       x=element_blank(),
       y=element_blank()) +
  guides(alpha = "none") +
  theme(panel.grid = element_blank(), panel.border = element_blank(),
        strip.background = element_blank(),strip.text.x = element_text(size=5),
        axis.text.y=element_blank(),
        axis.ticks.y=element_blank(),
        plot.background = element_rect(fill = "transparent", colour = NA))

summary_de_agent_surpgpt.condition <- preds.de.agent.surpgpt_long %>%
  filter(surprisal == 0) %>%
  group_by(x,y,samp) %>%
  mutate(diff_condition = value[condition == 'Abias'] -
           value[condition == 'nonAbias']) %>%
  ungroup() %>%
  group_by(x,y) %>%
  summarise(PP = ifelse(mean(diff_condition) > 0,
                        mean(diff_condition > 0),
                        mean(diff_condition < 0)),
            mean_diff = mean(diff_condition))  %>%
  mutate(x = -x)

plot.gpt.agent.de.cond <- summary_de_agent_surpgpt.condition %>%
  mutate(sig = ifelse(PP>0.80, 1, 0)) %>%
  ggplot(aes(x=x, y=y, fill=mean_diff, alpha = sig)) +
  geom_tile()+
  scale_fill_gradient2(low = '#0053d3', high = '#b22709', midpoint=0,
                       aesthetics='fill')+
  labs(fill=(expression(paste(Delta, paste(mu, "V")))),
       x=element_blank(),
       y=element_blank()) +
  guides(alpha = "none") +
  theme(panel.grid = element_blank(), panel.border = element_blank(),
        strip.background = element_blank(),strip.text.x = element_text(size=5),
        axis.text.y=element_blank(),
        axis.text.x = element_blank(),
        axis.ticks.y=element_blank(),
        axis.ticks.x=element_blank(),
        plot.background = element_rect(fill = "transparent", colour = NA))

plot.gpt.agent.de.cond.pp <- summary_de_agent_surpgpt.condition %>%
  ggplot(aes(x=x, y=y, fill=PP)) +
  geom_tile()+
  scale_fill_gradientn(colours = c('white','grey', 'black'),limits = c(0.8, 1),
                       na.value='white' ) +
  labs(fill=(expression(paste('P(|',Delta,mu, V, '| > 0)', ))),
       x=element_blank(),
       y=element_blank()) +
  guides(alpha = "none") +
  theme(panel.grid = element_blank(), panel.border = element_blank(),
        strip.background = element_blank(),strip.text.x = element_text(size=5),
        axis.text.y=element_blank(),
        axis.ticks.y=element_blank(),
```

```
        plot.background = element_rect(fill = "transparent", colour = NA))
```

```
wrap_plots(list(plot.gpt.agent.de.cond, plot.gpt.agent.surp.de,
                plot.gpt.agent.de.cond.pp, plot.gpt.agent.surp.de.pp))
```

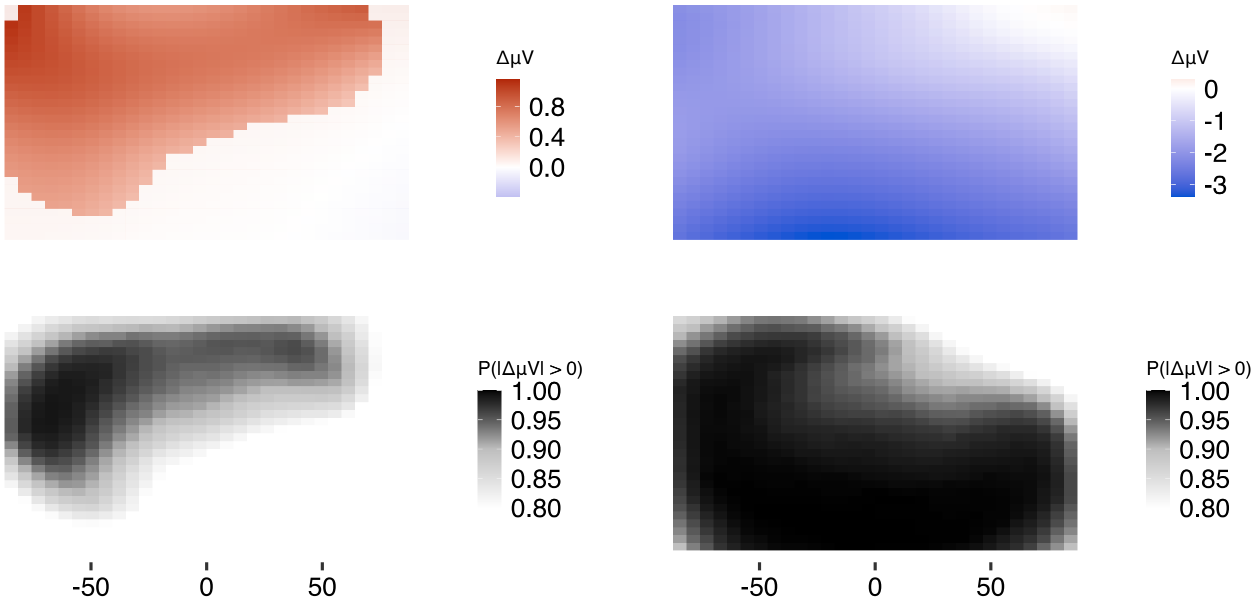

Figure S7: Left: Pair-wise fitted differences of micro-volt for sentences with vs. sentences without the predicted reanalysis from model with GPT-2 surprisal as well as agent preference predictor. Right: Percentage of posterior probability mass of micro-volt mean difference below or above 0.

S3.3.8 Strongest effect summary

Table S1: German: This table contains the lowest mean delta microVolt (i.e. strongest effect size across the scalp, blue in the plots above) and the maximum proportion of the posterior distribution that is lower than 0 for the highest-ranked model, agent preference + bert surprisal.

predictor	$min(mean(\Delta\mu V))$	$max(P\Delta\mu V < 0)$	NA
agent preference and lstm surprisal	surprisal	-6.625	1.0
agent preference and lstm surprisal	agent preference	-0.951	0.9

4 Analysis 2: Predicting ERPs for Hindi

S4: Study 2: Predicting ERPs (Hindi)*

Eva Huber Sebastian Sauppe Arrate Isasi-Isasmendi
Ina Bornkessel-Schlesewsky Paola Merlo Balthasar Bickel

2023-08-29

Contents

S4 Hindi **1**
S4.1 Models . . . 1
S4.2 Model comparison with stacking . . . 12
S4.3 Model predictions . . . 13

REPRODUCIBILITY STATEMENT: All of the models included here were fitted on an M1 processor using R Version 4.2.2 and brms version 2.14.0.

S4 Hindi

S4.1 Models

We model the EEG results in micro-volt (`mV` below). For this, we fit Bayesian Generalised Additive Models in `brms` with different predictors and compare models by means of stacking in order to find the model with highest predictive performance. In the model below `condition` refers to whether or not we expect a reanalysis (see main text).

We check convergence by ensuring that Effective Sample Size $ESS > 100$ and diagnostic $\hat{R} < 1.05$. Additionally, we perform posterior predictive checks to compare the predicted values to the observed data.

S4.1.1 Agent preference alone

```
hindi.data <- readRDS('../results/hindi_mv_surprisal_0506.RDS')

if (!file.exists('../gams_fitted_models/gam_hi_agent.RDS')){
  prior.brms <-  c(
    prior(normal(0,1), class = b),
    prior(exponential(1), class = sigma)
  )
  model.agent <-  brm(mV
                    ~ 1
                    + condition
```

*This document was `rmarkdown::render`'ed from an `R` script 'main_scripts/Hindi_analysis2.R' available at https://osf.io/hbj67.

```r
                      + trial_number.Z
                      + t2(x, y, by = condition, k = 10)
                      + t2(x, y, Participant, bs = "re")
                      + t2(x, y, Stimulus, bs = "re"),
                      data = hindi.data,
                      sample_prior = TRUE,
                      prior = prior.brms,
                      chains = 4, iter = 2000, cores = 4
  )
  saveRDS(model.agent, '../gams_fitted_models/gam_hi_agent.RDS')
}
model.hi.agent <- readRDS('../gams_fitted_models/gam_hi_agent.RDS')
summary(model.hi.agent)
```

```
##  Family: gaussian
##   Links: mu = identity; sigma = identity
## Formula: mV ~ 1 + trial_number.Z + condition + t2(x, y, by = condition, k = 10) + t2(x, y, Participan
##    Data: hindi.data (Number of observations: 31104)
## Samples: 4 chains, each with iter = 2000; warmup = 1000; thin = 1;
##          total post-warmup samples = 4000
##
## Smooth Terms:
##                                 Estimate Est.Error l-95% CI u-95% CI Rhat Bulk_ESS Tail_ESS
## sds(t2xyconditionAbias_1)           0.77      0.71     0.03     2.52 1.00     2423     2674
## sds(t2xyconditionAbias_2)           7.93      2.54     4.37    13.96 1.00     1850     2757
## sds(t2xyconditionAbias_3)           4.42      1.84     1.84     8.77 1.00     2396     2703
## sds(t2xyconditionnonAbias_1)        0.75      0.69     0.02     2.51 1.00     2056     2198
## sds(t2xyconditionnonAbias_2)        6.93      2.29     3.54    12.33 1.00     2149     2968
## sds(t2xyconditionnonAbias_3)        3.14      1.63     0.73     7.05 1.00     1944     1651
## sds(t2xyParticipant_1)              0.23      0.11     0.02     0.44 1.01      609      320
## sds(t2xyStimulus_1)                 0.06      0.05     0.00     0.17 1.00     3415     2120
##
## Population-Level Effects:
##                             Estimate Est.Error l-95% CI u-95% CI Rhat Bulk_ESS Tail_ESS
## Intercept                       0.32      0.07     0.18     0.45 1.00     4593     3199
## trial_number.Z                 -0.11      0.04    -0.19    -0.04 1.00     8761     3030
## condition2                     -0.01      0.09    -0.19     0.18 1.00     3915     3094
## t2xy:conditionAbias_1          -0.09      0.08    -0.24     0.06 1.00     3182     2729
## t2xy:conditionAbias_2           1.15      0.07     1.01     1.29 1.00     4351     2850
## t2xy:conditionAbias_3           0.05      0.10    -0.14     0.25 1.00     2852     3236
## t2xy:conditionnonAbias_1       -0.19      0.07    -0.33    -0.06 1.00     3622     3144
## t2xy:conditionnonAbias_2        1.22      0.06     1.09     1.34 1.00     3626     3011
## t2xy:conditionnonAbias_3       -0.01      0.09    -0.17     0.17 1.00     3409     3036
##
## Family Specific Parameters:
##       Estimate Est.Error l-95% CI u-95% CI Rhat Bulk_ESS Tail_ESS
## sigma     6.59      0.03     6.54     6.64 1.00     8963     3079
##
## Samples were drawn using sampling(NUTS). For each parameter, Bulk_ESS
## and Tail_ESS are effective sample size measures, and Rhat is the potential
## scale reduction factor on split chains (at convergence, Rhat = 1).
```

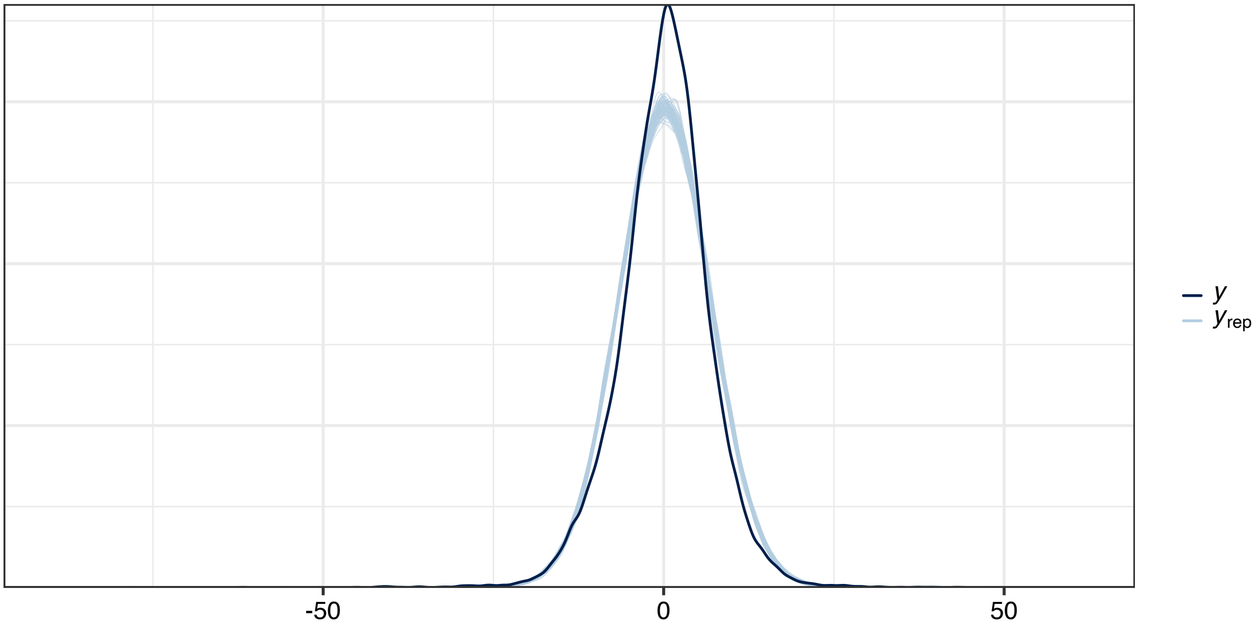

S4.1.2 Surprisal alone

LSTM-based surprisal:

```
if (!file.exists('../gams_fitted_models/gam_hi_lstm.RDS')){
  model.surprisal.lstm <-  brm(mV
                                ~ 1
                                + trial_number.Z
                                + surprisal.lstm.Z
                                + t2(x, y, by = surprisal.lstm.Z, k = 10)
                                + t2(x, y, Participant, bs = "re")
                                + t2(x, y, Stimulus, bs = "re"),
                                prior = prior.brms,
                                sample_prior = TRUE,
                                data = hindi.data,
                                chains = 4, iter = 2000, cores = 4
  )
  saveRDS(model.surprisal.lstm, '../gams_fitted_models/gam_hi_lstm.RDS')
}
model.hi.surplstm <- readRDS('../gams_fitted_models/gam_hi_lstm.RDS')
summary(model.hi.surplstm)
```

```
##  Family: gaussian
##   Links: mu = identity; sigma = identity
## Formula: mV ~ 1 + trial_number.Z + surprisal.lstm.Z + t2(x, y, by = surprisal.lstm.Z, k = 10) + t2(x
##    Data: hindi.data (Number of observations: 31104)
## Samples: 4 chains, each with iter = 2000; warmup = 1000; thin = 1;
##          total post-warmup samples = 4000
##
## Smooth Terms:
##                              Estimate Est.Error l-95% CI u-95% CI Rhat Bulk_ESS Tail_ESS
## sds(t2xysurprisal.lstm.Z_1)      0.38      0.36     0.01     1.34 1.00     2546     2566
```

```
## sds(t2xysurprisal.lstm.Z_2)     0.81      0.75     0.03     2.80 1.00     2790     2176
## sds(t2xysurprisal.lstm.Z_3)     0.91      0.82     0.03     3.09 1.00     2109     1936
## sds(t2xyParticipant_1)          0.22      0.10     0.02     0.42 1.00     1006     1010
## sds(t2xyStimulus_1)             0.06      0.05     0.00     0.17 1.00     3134     2159
##
## Population-Level Effects:
##                         Estimate Est.Error l-95% CI u-95% CI Rhat Bulk_ESS Tail_ESS
## Intercept                   0.13      0.04     0.06     0.21 1.00     8694     2731
## trial_number.Z             -0.11      0.04    -0.19    -0.04 1.00     8484     2781
## surprisal.lstm.Z           -0.01      0.71    -1.41     1.40 1.00     4863     3267
## t2xy:surprisal.lstm.Z_1     0.02      0.68    -1.31     1.35 1.00     4845     3185
## t2xy:surprisal.lstm.Z_2     0.06      0.04    -0.02     0.15 1.00     7020     3552
## t2xy:surprisal.lstm.Z_3    -0.03      0.04    -0.11     0.06 1.00     8443     3088
## t2xy:surprisal.lstm.Z_4     0.00      0.05    -0.09     0.09 1.00     7996     3415
##
## Family Specific Parameters:
##       Estimate Est.Error l-95% CI u-95% CI Rhat Bulk_ESS Tail_ESS
## sigma     6.70      0.03     6.64     6.75 1.00    13108     2637
##
## Samples were drawn using sampling(NUTS). For each parameter, Bulk_ESS
## and Tail_ESS are effective sample size measures, and Rhat is the potential
## scale reduction factor on split chains (at convergence, Rhat = 1).
```

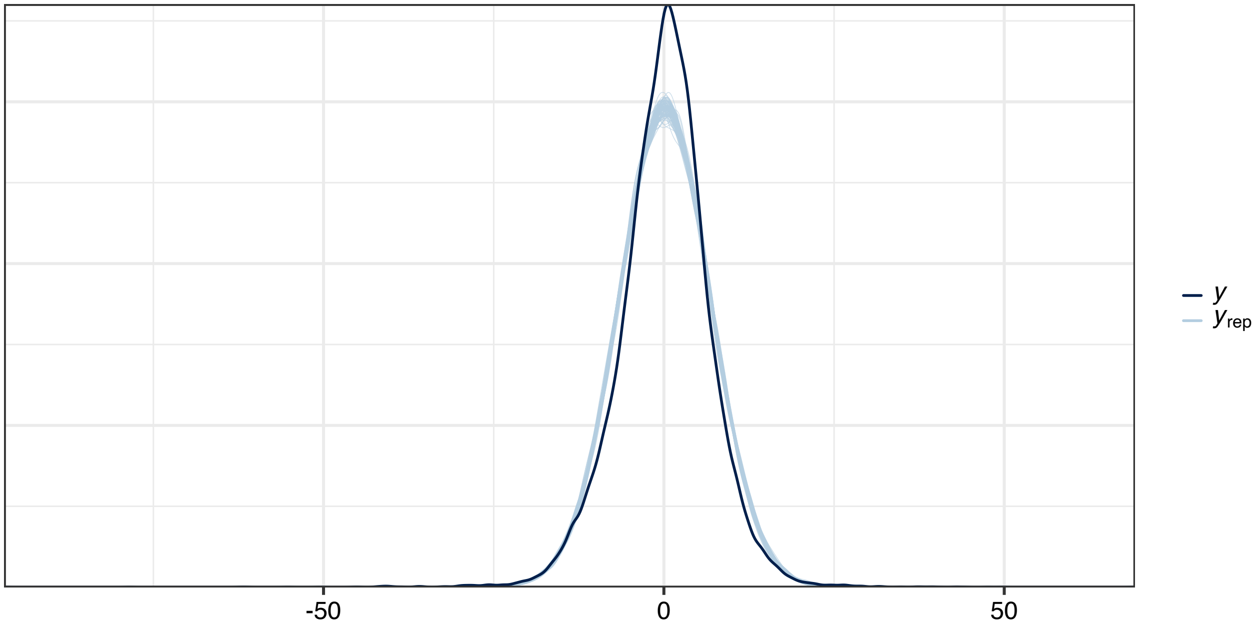

BERT-based surprisal:

```
if (!file.exists('../gams_fitted_models/gam_hi_bert.RDS')){
  model.surprisal.tr <-  brm(mV
                             ~ 1
                             + trial_number.Z
                             + surprisal.tr.Z
                             + t2(x, y, by = surprisal.tr.Z, k = 10)
                             + t2(x, y, Participant, bs = "re")
                             + t2(x, y, Stimulus, bs = "re"),
                             prior = prior.brms,
```

```r
                              sample_prior = TRUE,
                              data = hindi.data,
                              chains = 4, iter = 2000, cores = 4
  )
  saveRDS(model.surprisal.tr,'../gams_fitted_models/gam_hi_bert.RDS')
}
model.hi.surptr <- readRDS('../gams_fitted_models/gam_hi_bert.RDS')
summary(model.hi.surptr)
```

```
##  Family: gaussian
##   Links: mu = identity; sigma = identity
## Formula: mV ~ 1 + trial_number.Z + surprisal.tr.Z + t2(x, y, by = surprisal.tr.Z, k = 10) + t2(x, y,
##    Data: hindi.data (Number of observations: 31104)
## Samples: 4 chains, each with iter = 2000; warmup = 1000; thin = 1;
##          total post-warmup samples = 4000
##
## Smooth Terms:
##                            Estimate Est.Error l-95% CI u-95% CI Rhat Bulk_ESS Tail_ESS
## sds(t2xysurprisal.tr.Z_1)     0.42      0.38     0.01     1.45 1.00     2512     2427
## sds(t2xysurprisal.tr.Z_2)     0.84      0.82     0.03     2.88 1.00     2861     2605
## sds(t2xysurprisal.tr.Z_3)     0.86      0.80     0.03     2.99 1.00     2915     2362
## sds(t2xyParticipant_1)        0.22      0.10     0.02     0.41 1.00      935     1053
## sds(t2xyStimulus_1)           0.06      0.05     0.00     0.17 1.00     2562     1632
##
## Population-Level Effects:
##                        Estimate Est.Error l-95% CI u-95% CI Rhat Bulk_ESS Tail_ESS
## Intercept                  0.13      0.04     0.06     0.21 1.00     9762     2807
## trial_number.Z            -0.12      0.04    -0.19    -0.04 1.00    10493     2667
## surprisal.tr.Z            -0.07      0.73    -1.52     1.40 1.00     5283     2848
## t2xy:surprisal.tr.Z_1     -0.07      0.70    -1.48     1.31 1.00     5253     2923
## t2xy:surprisal.tr.Z_2      0.02      0.04    -0.06     0.10 1.00     6375     3207
## t2xy:surprisal.tr.Z_3      0.05      0.04    -0.03     0.13 1.00     8209     3003
## t2xy:surprisal.tr.Z_4     -0.00      0.05    -0.10     0.09 1.00     7685     3077
##
## Family Specific Parameters:
##       Estimate Est.Error l-95% CI u-95% CI Rhat Bulk_ESS Tail_ESS
## sigma     6.70      0.03     6.65     6.75 1.00     8193     2223
##
## Samples were drawn using sampling(NUTS). For each parameter, Bulk_ESS
## and Tail_ESS are effective sample size measures, and Rhat is the potential
## scale reduction factor on split chains (at convergence, Rhat = 1).
```

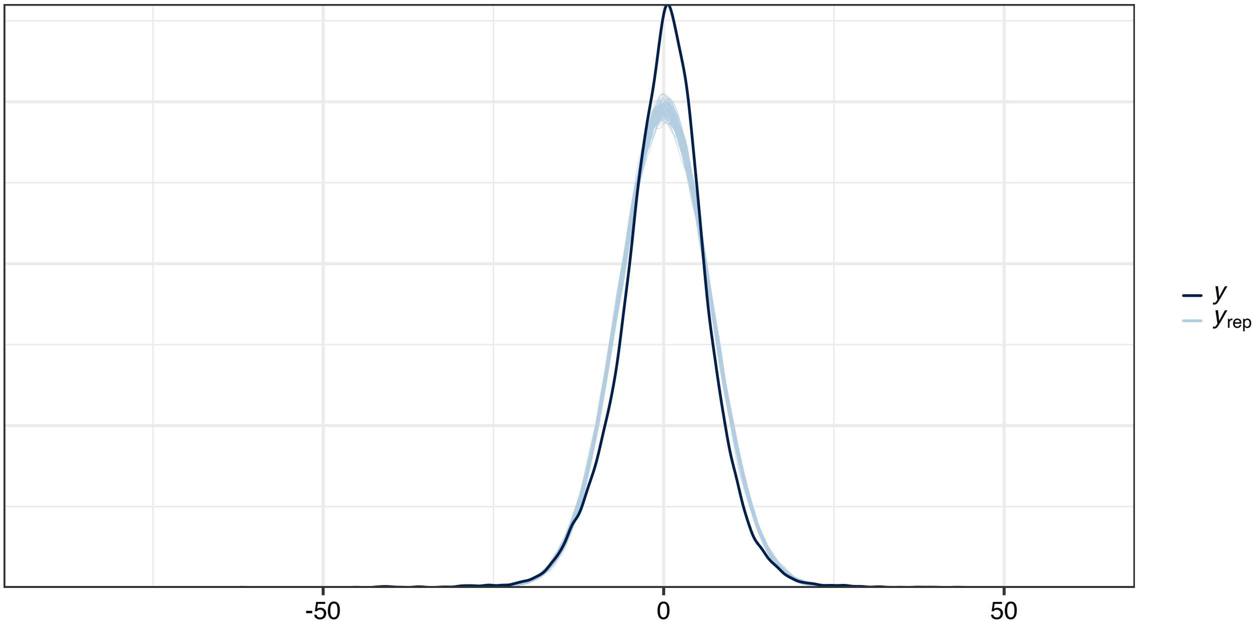

GPT-based surprisal

```
if (!file.exists('../gams_fitted_models/gam_hi_gpt.RDS')){
  model.surprisal.gpt <-  brm(mV
                              ~ 1
                              + trial_number.Z
                              + surprisal.gpt.Z
                              + t2(x, y, by = surprisal.gpt.Z, k = 10)
                              + t2(x, y, Participant, bs = "re")
                              + t2(x, y, Stimulus, bs = "re"),
                              prior = prior.brms,
                              sample_prior = TRUE,
                              data = hindi.data,
                              chains = 4, iter = 2000, cores = 4
  )
  saveRDS(model.surprisal.gpt,'../gams_fitted_models/gam_hi_gpt.RDS')
}
model.hi.surpgpt <- readRDS('../gams_fitted_models/gam_hi_gpt.RDS')
summary(model.hi.surpgpt)
```

```
##  Family: gaussian
##   Links: mu = identity; sigma = identity
## Formula: mV ~ 1 + trial_number.Z + surprisal.gpt.Z + t2(x, y, by = surprisal.gpt.Z, k = 10) + t2(x, 
##    Data: hindi.data (Number of observations: 31104)
## Samples: 4 chains, each with iter = 2000; warmup = 1000; thin = 1;
##          total post-warmup samples = 4000
##
## Smooth Terms:
##                            Estimate Est.Error l-95% CI u-95% CI Rhat Bulk_ESS Tail_ESS
## sds(t2xysurprisal.gpt.Z_1)     0.28      0.27     0.01     1.00 1.00     2294     2808
## sds(t2xysurprisal.gpt.Z_2)     2.71      1.00     1.33     5.31 1.00     2295     2998
## sds(t2xysurprisal.gpt.Z_3)     1.46      0.75     0.45     3.35 1.00     1966     1682
## sds(t2xyParticipant_1)         0.21      0.10     0.02     0.42 1.01      943      729
## sds(t2xyStimulus_1)            0.06      0.05     0.00     0.17 1.00     3155     2276
```

```
##
## Population-Level Effects:
##                          Estimate Est.Error l-95% CI u-95% CI Rhat Bulk_ESS Tail_ESS
## Intercept                    0.13      0.05     0.04     0.22 1.00     4303     2502
## trial_number.Z              -0.11      0.04    -0.19    -0.04 1.00     8526     2673
## surprisal.gpt.Z              0.03      0.74    -1.43     1.45 1.00     2915     2745
## t2xy:surprisal.gpt.Z_1       0.03      0.70    -1.32     1.44 1.00     2920     2739
## t2xy:surprisal.gpt.Z_2       0.04      0.03    -0.01     0.09 1.00     4083     2982
## t2xy:surprisal.gpt.Z_3      -0.33      0.02    -0.38    -0.28 1.00     4809     3135
## t2xy:surprisal.gpt.Z_4       0.02      0.03    -0.05     0.08 1.00     4199     3143
##
## Family Specific Parameters:
##       Estimate Est.Error l-95% CI u-95% CI Rhat Bulk_ESS Tail_ESS
## sigma     6.66      0.03     6.61     6.72 1.00     6654     2492
##
## Samples were drawn using sampling(NUTS). For each parameter, Bulk_ESS
## and Tail_ESS are effective sample size measures, and Rhat is the potential
## scale reduction factor on split chains (at convergence, Rhat = 1).
```

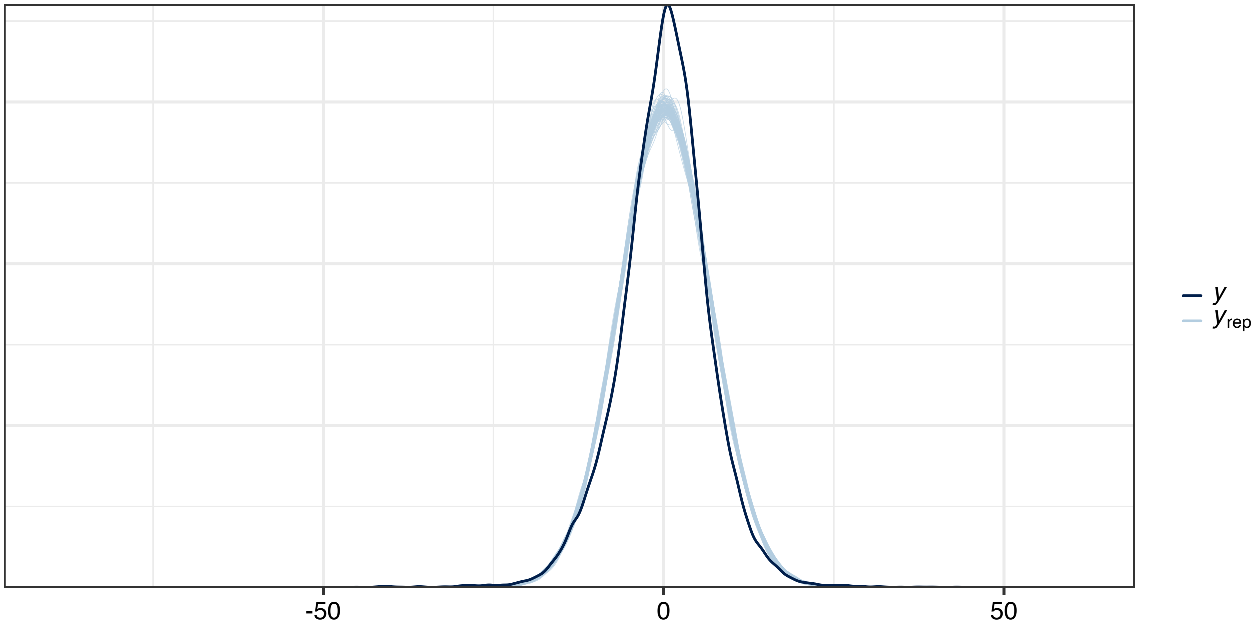

S4.1.3 Agent preference and surprisal

LSTM-based surprisal:

```
if (!file.exists('../gams_fitted_models/gam_hi_agent_lstm.RDS')){
  model.agent.surprisal.lstm <-  brm(mV
                                     ~ 1
                                     + trial_number.Z
                                     + surprisal.lstm.Z
                                     + condition
                                     + t2(x, y, by = condition, k = 10)
                                     + t2(x, y, by = surprisal.lstm.Z, k = 10)
                                     + t2(x, y, Participant, bs = "re")
```

```r
                                  + t2(x, y, Stimulus, bs = "re"),
                                  prior = prior.brms,
                                  sample_prior = TRUE,
                                  data = hindi.data,
                                  chains = 4, iter = 2000, cores = 4
  )
  saveRDS(model.agent.surprisal.lstm, '../gams_fitted_models/gam_hi_agent_lstm.RDS')
}
model.hi.agent.surplstm <- readRDS('../gams_fitted_models/gam_hi_agent_lstm.RDS')
summary(model.hi.agent.surplstm)
```

```
##  Family: gaussian
##   Links: mu = identity; sigma = identity
## Formula: mV ~ 1 + trial_number.Z + surprisal.lstm.Z + condition + t2(x, y, by = condition, k = 10) +
##    Data: hindi.data (Number of observations: 31104)
## Samples: 4 chains, each with iter = 2000; warmup = 1000; thin = 1;
##          total post-warmup samples = 4000
##
## Smooth Terms:
##                                 Estimate Est.Error l-95% CI u-95% CI Rhat Bulk_ESS Tail_ESS
## sds(t2xyconditionAbias_1)           0.77      0.73     0.02     2.71 1.00     2097     1899
## sds(t2xyconditionAbias_2)           7.97      2.52     4.36    14.02 1.00     2363     2741
## sds(t2xyconditionAbias_3)           4.44      1.87     1.84     9.01 1.00     2573     2811
## sds(t2xyconditionnonAbias_1)        0.74      0.67     0.03     2.49 1.00     2303     2284
## sds(t2xyconditionnonAbias_2)        6.80      2.28     3.55    12.46 1.00     1824     2515
## sds(t2xyconditionnonAbias_3)        3.19      1.65     0.73     7.18 1.00     2290     2081
## sds(t2xysurprisal.lstm.Z_1)         0.39      0.36     0.01     1.33 1.00     2835     2285
## sds(t2xysurprisal.lstm.Z_2)         0.79      0.76     0.02     2.81 1.00     3083     2736
## sds(t2xysurprisal.lstm.Z_3)         0.91      0.82     0.04     3.04 1.00     2198     2481
## sds(t2xyParticipant_1)              0.24      0.10     0.02     0.44 1.00      718      389
## sds(t2xyStimulus_1)                 0.06      0.05     0.00     0.17 1.00     3610     2300
##
## Population-Level Effects:
##                           Estimate Est.Error l-95% CI u-95% CI Rhat Bulk_ESS Tail_ESS
## Intercept                     0.31      0.07     0.18     0.45 1.00     4788     3436
## trial_number.Z               -0.11      0.04    -0.19    -0.04 1.00    10193     2966
## surprisal.lstm.Z              0.03      0.73    -1.38     1.44 1.00     4252     3121
## condition2                    0.00      0.09    -0.18     0.19 1.00     4238     3265
## t2xy:conditionAbias_1         0.09      0.08    -0.07     0.24 1.00     4024     3116
## t2xy:conditionAbias_2        -1.15      0.07    -1.29    -1.01 1.00     5362     3237
## t2xy:conditionAbias_3         0.05      0.10    -0.14     0.24 1.00     3455     3134
## t2xy:conditionnonAbias_1      0.19      0.07     0.06     0.33 1.00     3921     3154
## t2xy:conditionnonAbias_2     -1.22      0.06    -1.34    -1.09 1.00     4934     3187
## t2xy:conditionnonAbias_3     -0.01      0.09    -0.18     0.16 1.00     3590     3214
## t2xy:surprisal.lstm.Z_1      -0.01      0.70    -1.36     1.34 1.00     4196     3290
## t2xy:surprisal.lstm.Z_2       0.06      0.04    -0.02     0.15 1.00     7171     3550
## t2xy:surprisal.lstm.Z_3      -0.03      0.04    -0.10     0.05 1.00     6627     3197
## t2xy:surprisal.lstm.Z_4       0.00      0.05    -0.09     0.10 1.00     7109     2944
##
## Family Specific Parameters:
##       Estimate Est.Error l-95% CI u-95% CI Rhat Bulk_ESS Tail_ESS
## sigma     6.59      0.03     6.54     6.64 1.00    10150     2984
##
```

```
## Samples were drawn using sampling(NUTS). For each parameter, Bulk_ESS
## and Tail_ESS are effective sample size measures, and Rhat is the potential
## scale reduction factor on split chains (at convergence, Rhat = 1).
```

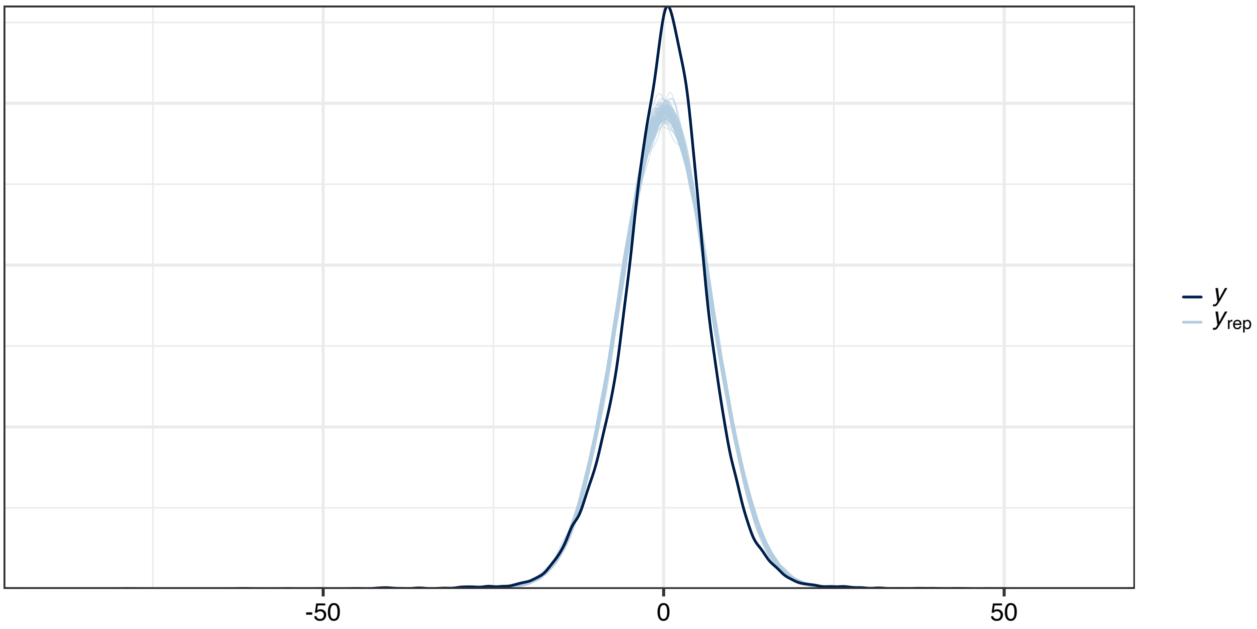

BERT-based surprisal:

```
if (!file.exists('../gams_fitted_models/gam_hi_agent_bert.RDS')){
  model.agent.surprisal.tr <-  brm(mV
                                   ~ 1
                                   + trial_number.Z
                                   + surprisal.tr.Z
                                   + condition
                                   + t2(x, y, by = condition, k = 10)
                                   + t2(x, y, by = surprisal.tr.Z, k = 10)
                                   + t2(x, y, Participant, bs = "re")
                                   + t2(x, y, Stimulus, bs = "re"),
                                   prior = prior.brms,
                                   sample_prior = TRUE,
                                   data = hindi.data,
                                   chains = 4, iter = 2000, cores = 4
  )
  saveRDS(model.agent.surprisal.tr,
          '../gams_fitted_models/gam_hi_agent_bert.RDS')
}
model.hi.agent.surptr <- readRDS('../gams_fitted_models/gam_hi_agent_bert.RDS')
```

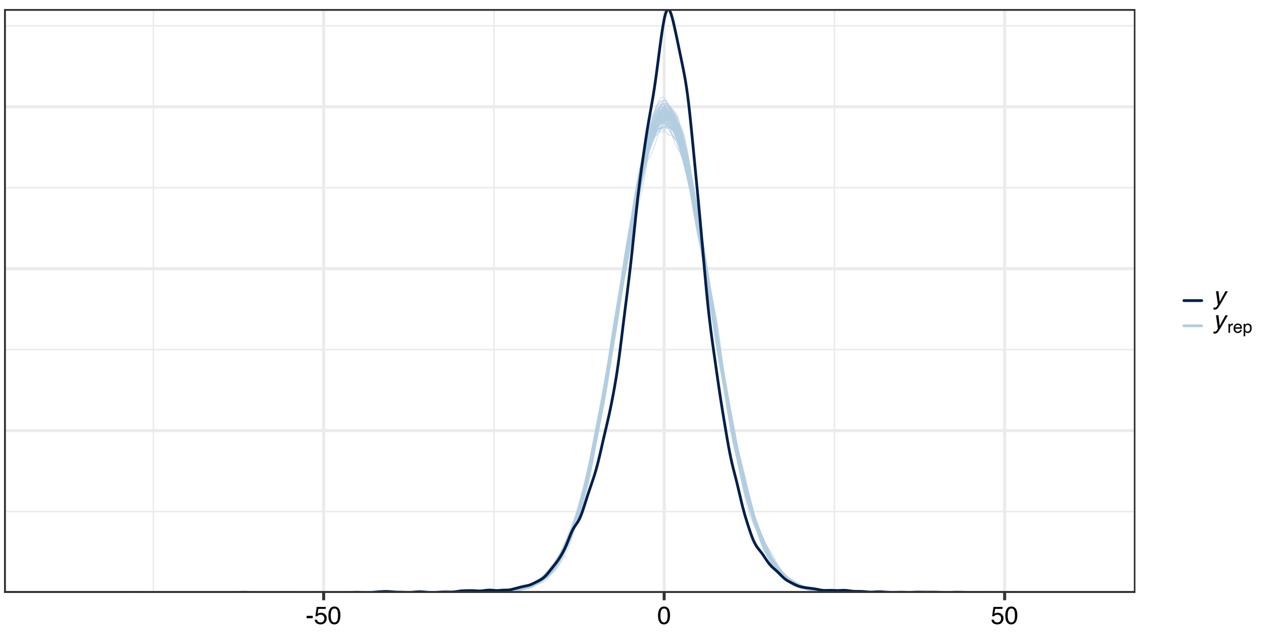

GPT-based surprisal:

```
if (!file.exists('../gams_fitted_models/gam_hi_agent_gpt.RDS')){
  model.agent.surprisal.gpt <-  brm(mV
                                    ~ 1
                                    + trial_number.Z
                                    + surprisal.gpt.Z
                                    + condition
                                    + t2(x, y, by = condition, k = 10)
                                    + t2(x, y, by = surprisal.gpt.Z, k = 10)
                                    + t2(x, y, Participant, bs = "re")
                                    + t2(x, y, Stimulus, bs = "re"),
                                    prior = prior.brms,
                                    sample_prior = TRUE,
                                    data = hindi.data,
                                    chains = 4, iter = 2000, cores = 4
  )
  saveRDS(model.agent.surprisal.gpt,
          '../gams_fitted_models/gam_hi_agent_gpt.RDS')
}
model.hi.agent.surpgpt <- readRDS('../gams_fitted_models/gam_hi_agent_gpt.RDS')
```

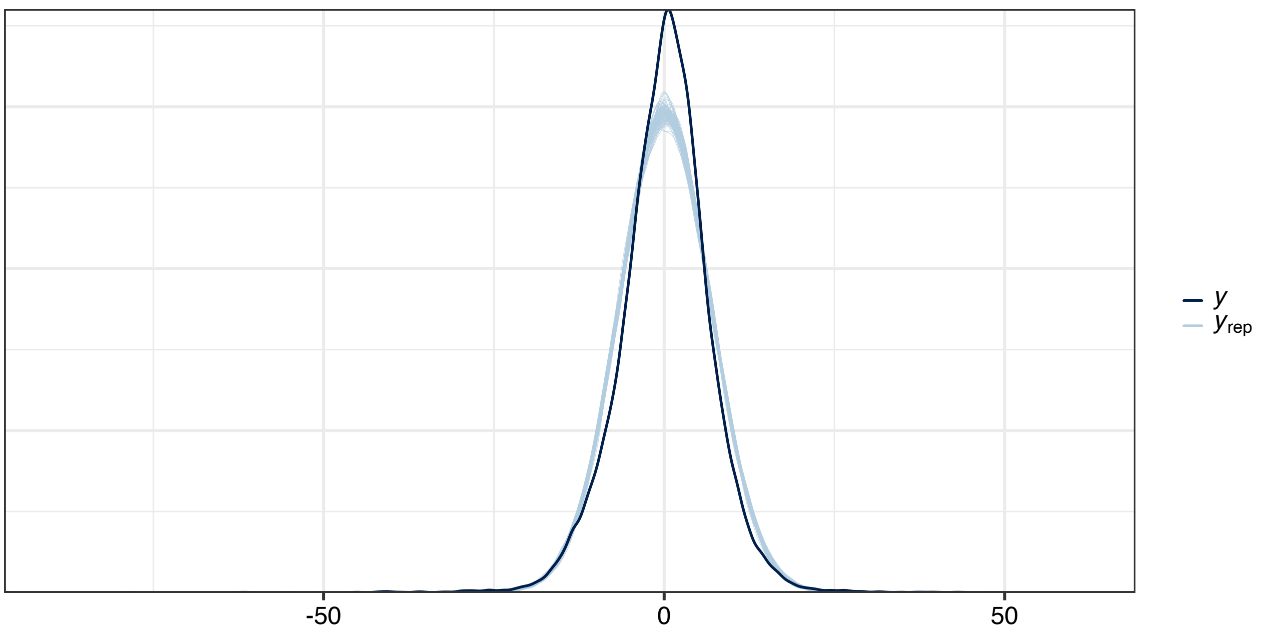

S4.1.4 Null model

```
if (!file.exists('../gams_fitted_models/gam_hi_null.RDS')){
  model.null <-  brm(mV
                     ~ 1
                     + trial_number.Z
                     + t2(x, y, Participant, bs = "re")
                     + t2(x, y, Stimulus, bs = "re"),
                     prior = prior.brms,
                     sample_prior = TRUE,
                     data = hindi.data,
                     chains = 4, iter = 2000, cores = 4
  )
  saveRDS(model.null, '../gams_fitted_models/gam_hi_null.RDS.RDS')
}
model.hi.null <- readRDS('../gams_fitted_models/gam_hi_null.RDS')
summary(model.hi.null)
```

```
##  Family: gaussian
##   Links: mu = identity; sigma = identity
## Formula: mV ~ 1 + trial_number.Z + t2(x, y, Participant, bs = "re") + t2(x, y, Stimulus, bs = "re")
##    Data: hindi.data (Number of observations: 31104)
## Samples: 4 chains, each with iter = 2000; warmup = 1000; thin = 1;
##          total post-warmup samples = 4000
##
## Smooth Terms:
##                          Estimate Est.Error l-95% CI u-95% CI Rhat Bulk_ESS Tail_ESS
## sds(t2xyParticipant_1)       0.22      0.10     0.03     0.42 1.00     1014     1295
## sds(t2xyStimulus_1)          0.06      0.05     0.00     0.17 1.00     2724     1666
##
## Population-Level Effects:
```

```
##                  Estimate Est.Error l-95% CI u-95% CI Rhat Bulk_ESS Tail_ESS
## Intercept            0.13      0.04     0.07     0.21 1.00     6421     3106
## trial_number.Z      -0.12      0.04    -0.19    -0.04 1.00     7135     2605
##
## Family Specific Parameters:
##       Estimate Est.Error l-95% CI u-95% CI Rhat Bulk_ESS Tail_ESS
## sigma     6.70      0.03     6.64     6.75 1.00     9181     2847
##
## Samples were drawn using sampling(NUTS). For each parameter, Bulk_ESS
## and Tail_ESS are effective sample size measures, and Rhat is the potential
## scale reduction factor on split chains (at convergence, Rhat = 1).
```

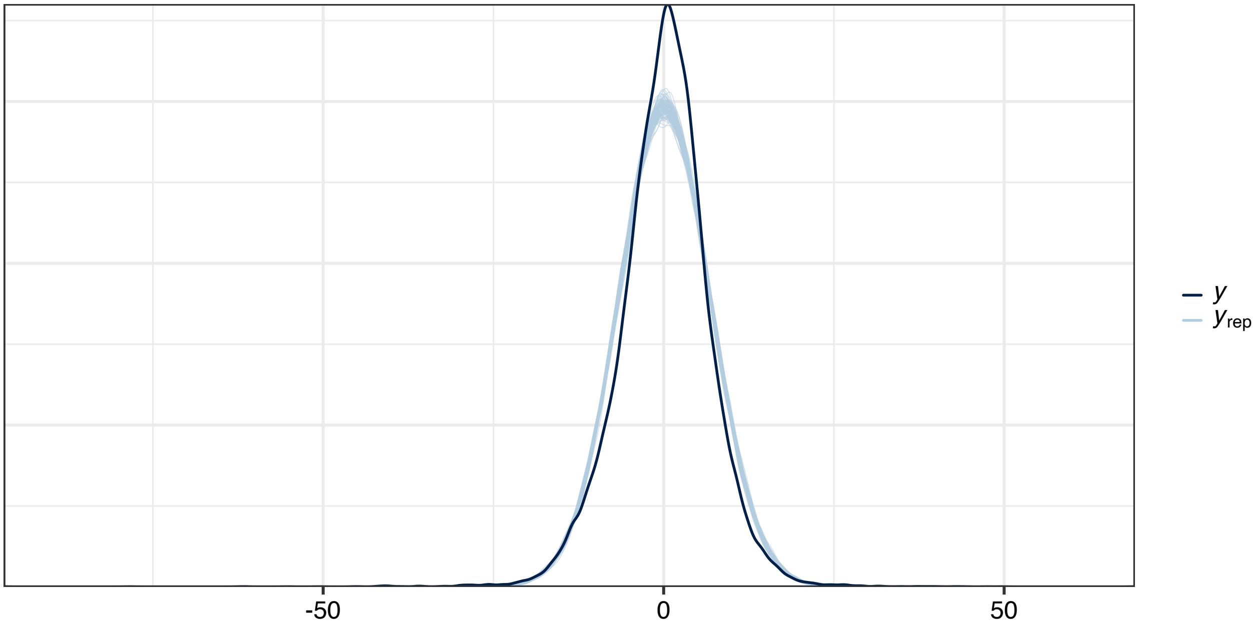

S4.2 Model comparison with stacking

Here we perform model comparison and report the results:

```
if (!file.exists('../temp_data/weights_brms_hindi_stacking.RDS')){
  loo.agent <- loo(model.hi.agent)#, moment_match = T)
  loo.surplstm <- loo(model.hi.surplstm)#, moment_match = T)
  loo.surptr <- loo(model.hi.surptr)#, moment_match = T)
  loo.surpgpt <- loo(model.hi.surpgpt)
  loo.agent.surplstm <- loo(model.hi.agent.surplstm)#, moment_match = T)
  loo.agent.surptr <- loo(model.hi.agent.surptr)#, moment_match = T)
  loo.agent.surpgpt <- loo(model.hi.agent.surpgpt)
  loo.null <- loo(model.hi.null)#, moment_match = T)
  lweights <- loo_model_weights(list(
    "agent bias only"= loo.agent,
    "surprisal lstm" =  loo.surplstm,
    "surprisal transformer" = loo.surptr,
    "surprisal gpt" = loo.surpgpt,
    "surprisal lstm and agent bias" = loo.agent.surplstm,
    "surprisal transformer and agent bias" = loo.agent.surptr,
```

```
    "surprisal gpt and agent bias" = loo.agent.surpgpt,
    "null" = loo.null), method='stacking')
  dfweights <- as.data.frame(as.matrix(lweights))
  saveRDS(dfweights,'../temp_data/weights_brms_hindi_stacking.RDS')
}
```

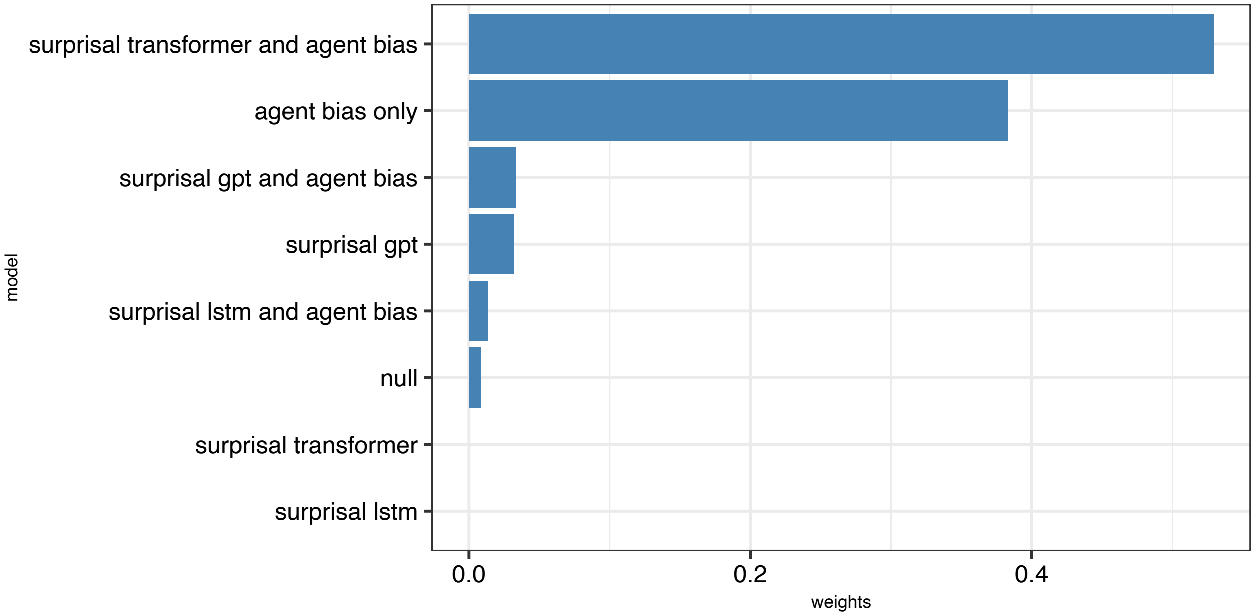

Figure S1: Hindi: Weights of models (method = stacking)

S4.3 Model predictions

In what follows we compute and plot the mean posterior predicted effects across the smoothed scalp surfaces. In each plot, the lef-hand side shows the difference between conditions. For surprisal we plot the difference between pedictions at +2 and at -2 standard deviations from the mean. Panels on the right-hand side quantify the evidence of the effect in terms of the proportion of the posterior distributions that is below or above 0.

S4.3.1 Agent preference alone

```
n_samps <- 4000

newdata.hi.agent <- expand_grid(
  x = seq(min(hindi.data$x), max(hindi.data$x), length.out = 30),
  y = seq(min(hindi.data$y), max(hindi.data$y),length.out = 30),
  Participant = "AD0001",
  Stimulus_Cond = c("1:Abias", "1:nonAbias"),
  trial_number.Z = 0,
)

newdata.hi.agent <- newdata.hi.agent %>%
  separate(Stimulus_Cond, c('Stimulus', 'condition'), sep=':')
```

```r
preds.hi.agent <- posterior_epred(model.hi.agent,
                                  newdata=newdata.hi.agent, re.form=NA)

preds.hi.agent_long <- as.data.frame(preds.hi.agent) %>%
  mutate(samp = 1:nrow(preds.hi.agent)) %>%
  pivot_longer(-samp)

preds.hi.agent_long$x <- rep(newdata.hi.agent$x, n_samps)
preds.hi.agent_long$y <- rep(newdata.hi.agent$y, n_samps)
preds.hi.agent_long$condition <- rep(newdata.hi.agent$condition, n_samps)

preds.hi.agent_summary <- preds.hi.agent_long %>%
  group_by(x,y,samp) %>%
  mutate(diff_cond = value[condition == 'Abias'] -
           value[condition == 'nonAbias']) %>%
  ungroup() %>%
  group_by(x,y) %>%
  summarise(PP = ifelse(mean(diff_cond) > 0, mean(diff_cond > 0),
                        mean(diff_cond < 0)),
            mean_diff = mean(diff_cond))  %>%
  rename(xax = y) %>% #axes need to be changed so that electrodes fit scalp
  rename(yax = x) %>%
  mutate(x = xax) %>%
  mutate(y = yax) %>%
  mutate(x = -x) %>%
  mutate(y = -y)

plot.agent.hi <- preds.hi.agent_summary %>%
  mutate(sig = ifelse(PP > 0.80, 1, 0)) %>%
  ggplot(aes(x=x, y=y, fill=mean_diff, alpha = sig)) +
  geom_tile()+
  scale_fill_gradient2(low = '#0053d3', high = '#b22709', midpoint=0,
                       aesthetics='fill')+
  guides(alpha = "none") +
  theme(panel.grid = element_blank(), panel.border = element_blank(),
        strip.background = element_blank(),strip.text.x = element_text(size=5),
        axis.text.y=element_blank(),
        axis.ticks.y=element_blank(),
        axis.text.x=element_blank(),
        axis.ticks.x =element_blank(),
        plot.background = element_rect(fill = "transparent", colour = NA))   +
  labs(fill=(expression(paste(Delta, paste(mu, "V")))),
       x = element_blank(), y = element_blank())

plot.agent.hi.pp <- preds.hi.agent_summary %>%
  ggplot(aes(x=x, y=y, fill=PP)) +
  geom_tile()+
  scale_fill_gradientn(colours = c('white','grey', 'black'),limits = c(0.8, 1),
                       na.value='white' ) +
  labs(fill=(expression(paste('P(|',Delta,mu, V, '| > 0)', ))),
       x=element_blank(),
       y=element_blank()) +
  theme(panel.grid = element_blank(),
```

```
        panel.border = element_blank(),
        strip.background = element_blank(),
        axis.ticks.x = element_blank(),
        axis.ticks.y=element_blank(),
        axis.text.x=element_blank(),
        axis.text.y = element_blank(),
        plot.background = element_rect(fill = "transparent", colour = NA))
```

```
wrap_plots(list(plot.agent.hi, plot.agent.hi.pp))
```

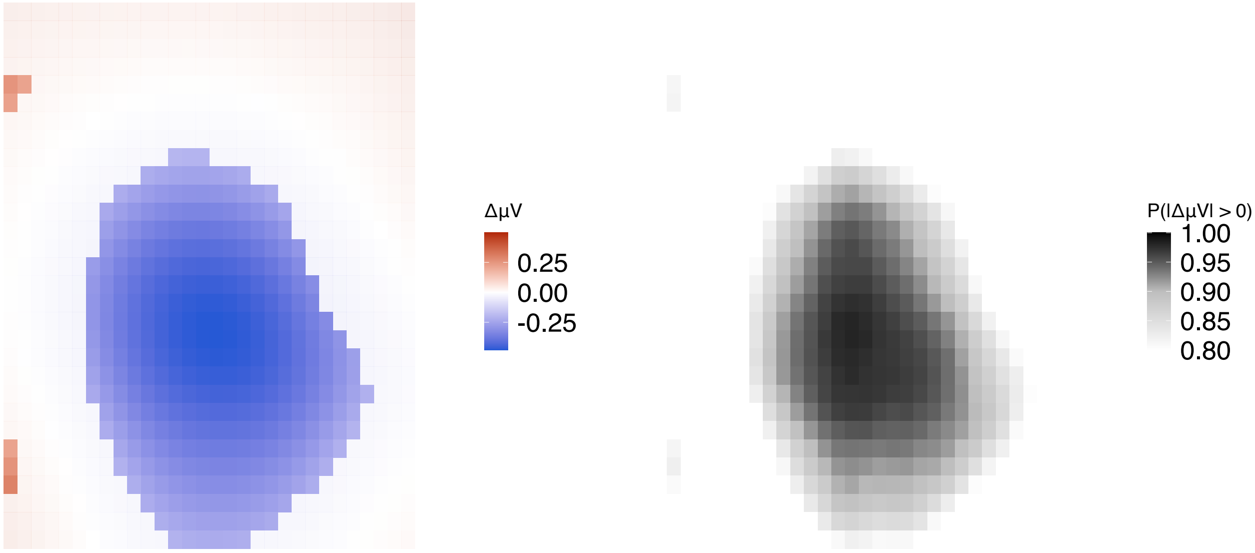

Figure S2: Left: Pair-wise fitted differences of μV for sentences with vs. sentences without the predicted reanalysis from model with agent preference predictor alone. Right: Percentage of posterior probability mass of μV mean difference below or above 0.

S4.3.2 LSTM-based surprisal alone

```
n_samps <- 4000

newdata.hi.surplstm <- expand_grid(
  x = seq(min(hindi.data$x), max(hindi.data$x), length.out = 30),
  y = seq(min(hindi.data$y), max(hindi.data$y),length.out = 30),
  surprisal.lstm.Z = seq(from=-2,to=2, length.out=17),
  Participant = "AD0001",
  Stimulus = c("1"),
  trial_number.Z = 0,
)

preds.hi.surplstm <- posterior_epred(model.hi.surplstm,
                                     newdata=newdata.hi.surplstm , re.form=NA)
preds.hi.surplstm_long <- as.data.frame(preds.hi.surplstm) %>%
  mutate(samp = 1:nrow(preds.hi.surplstm)) %>%
  pivot_longer(-samp)
```

```
preds.hi.surplstm_long$x <- rep(newdata.hi.surplstm$x, n_samps)
preds.hi.surplstm_long$y <- rep(newdata.hi.surplstm$y, n_samps)
preds.hi.surplstm_long$surprisal <- rep(newdata.hi.surplstm$surprisal.lstm.Z,
                                        n_samps)

summary_hi_surplstm <- preds.hi.surplstm_long %>%
  group_by(x,y,samp) %>%
  mutate(diff_surprisal = value[surprisal == 2] - value[surprisal == -2]) %>%
  ungroup() %>%
  group_by(x,y) %>%
  summarise(PP = ifelse(mean(diff_surprisal) > 0, mean(diff_surprisal > 0),
                      mean(diff_surprisal < 0)),
            mean_diff = mean(diff_surprisal)) %>%
  rename(xax = y) %>% #axes need to be changed so that electrodes fit scalp
  rename(yax = x) %>%
  mutate(x = xax) %>%
  mutate(y = yax) %>%
  mutate(x = -x) %>%
  mutate(y = -y)

plot.lstm.hi <- summary_hi_surplstm %>%
  mutate(sig = ifelse(PP>0.80, 1, 0)) %>%
  ggplot(aes(x=x, y=y, fill=mean_diff, alpha = sig)) +
  geom_tile()+
  scale_fill_gradient2(low = '#0053d3', high = '#b22709', midpoint=0,
                     aesthetics='fill')+
  guides(alpha = "none") +
  theme(panel.grid = element_blank(), panel.border = element_blank(),
        strip.background = element_blank(),strip.text.x = element_text(size=5),
        axis.text.y=element_blank(),
        axis.ticks.y=element_blank(),
        axis.text.x=element_blank(),
        axis.ticks.x =element_blank(),
        plot.background = element_rect(fill = "transparent", colour = NA))   +
  labs(fill=(expression(paste(Delta, paste(mu, "V")))),
       x = element_blank(), y = element_blank())

plot.lstm.hi.pp <- summary_hi_surplstm %>%
  ggplot(aes(x=x, y=y, fill=PP)) +
  geom_tile()+
  scale_fill_gradientn(colours = c('white','grey', 'black'),limits = c(0.8, 1),
                     na.value='white' ) +
  labs(fill=(expression(paste('P(|',Delta,mu, V, '| > 0)', ))),
       x=element_blank(),
       y=element_blank()) +
  theme(panel.grid = element_blank(), panel.border = element_blank(),
        strip.background = element_blank(),strip.text.x = element_text(size=5),
        axis.text.y=element_blank(),
        axis.ticks.y=element_blank(),
        plot.background = element_rect(fill = "transparent", colour = NA))
```

```
wrap_plots(list(plot.lstm.hi, plot.lstm.hi.pp))
```

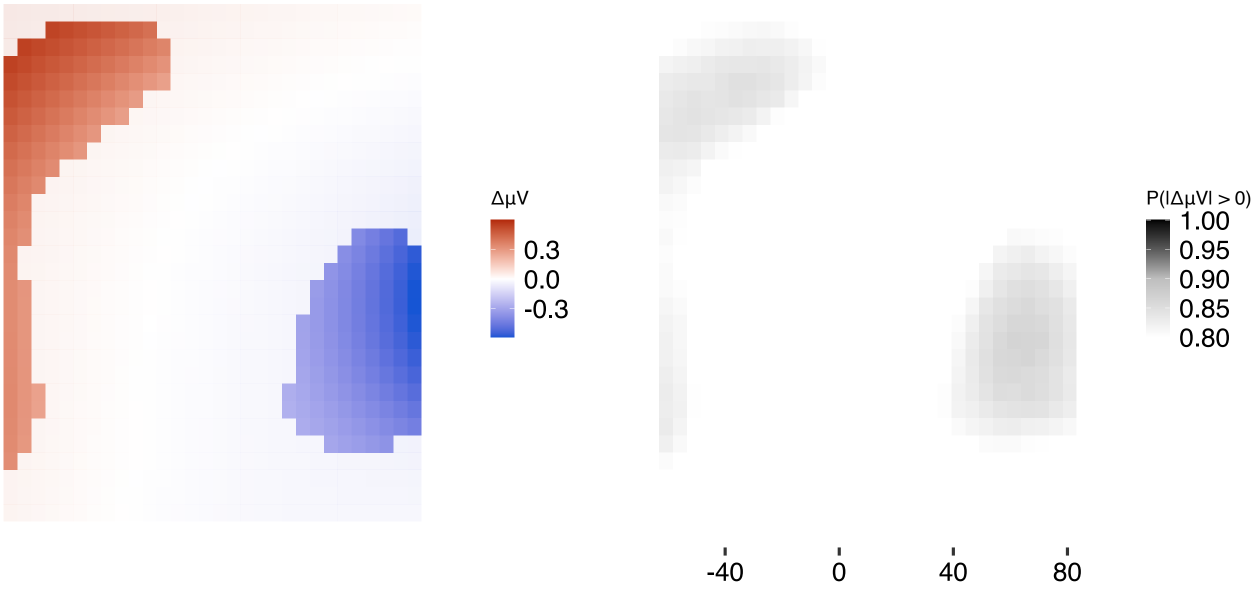

Figure S3: Left: Pair-wise fitted differences of μV for sentences with high vs. sentences with low (+2 vs. -2 std. dev. from the mean) from model with LSTM surprisal as predictor alone. Right: Percentage of posterior probability mass of μV mean difference below or above 0.

S4.3.3 BERT-based surprisal alone

```
n_samps <- brms::nsamples(model.hi.surptr)

newdata.hi.surptr <- rename(newdata.hi.surplstm,
                            surprisal.tr.Z = surprisal.lstm.Z)
preds.hi.surptr <- posterior_epred(model.hi.surptr,
                                   newdata=newdata.hi.surptr, re.form=NA)
preds.hi.surptr_long <- as.data.frame(preds.hi.surptr) %>%
  mutate(samp = 1:nrow(preds.hi.surptr)) %>%
  pivot_longer(-samp)

preds.hi.surptr_long$x <- rep(newdata.hi.surptr$x, n_samps)
preds.hi.surptr_long$y <- rep(newdata.hi.surptr$y, n_samps)
preds.hi.surptr_long$surprisal <- rep(newdata.hi.surptr$surprisal.tr.Z, n_samps)

summary_hi_surptr <- preds.hi.surptr_long %>%
  group_by(x,y,samp) %>%
  mutate(diff_surprisal_low_mean = value[surprisal == 2] -
         value[surprisal == -2]) %>%
  ungroup() %>%
  group_by(x,y) %>%
  summarise(PP = ifelse(mean(diff_surprisal_low_mean) > 0,
                        mean(diff_surprisal_low_mean > 0),
                        mean(diff_surprisal_low_mean < 0)),
            mean_diff = mean(diff_surprisal_low_mean)) %>%
  rename(xax = y) %>% #axes need to be changed so that electrodes fit scalp
  rename(yax = x) %>%
  mutate(x = xax) %>%
```

```
  mutate(y = yax) %>%
  mutate(x = -x) %>%
  mutate(y = -y)

plot.tr.hi <- summary_hi_surptr %>%
  mutate(sig = ifelse(PP>0.80, 1, 0)) %>%
  ggplot(aes(x=x, y=y, fill=mean_diff, alpha = sig)) +
  geom_tile()+
  scale_fill_gradient2(low = '#0053d3', high = '#b22709', midpoint=0,
                       aesthetics='fill')+
  guides(alpha = "none") +
  theme(panel.grid = element_blank(), panel.border = element_blank(),
        strip.background = element_blank(),strip.text.x = element_text(size=5),
        axis.text.y=element_blank(),
        axis.ticks.y=element_blank(),
        axis.text.x=element_blank(),
        axis.ticks.x =element_blank(),
        plot.background = element_rect(fill = "transparent", colour = NA))   +
  labs(fill=(expression(paste(Delta, paste(mu, "V")))),
       x = element_blank(), y = element_blank())

plot.tr.hi.pp <- summary_hi_surptr %>%
  ggplot(aes(x=x, y=y, fill=PP)) +
  geom_tile()+
  scale_fill_gradientn(colours = c('white','grey', 'black'),limits = c(0.8, 1),
                       na.value='white' ) +
  labs(fill=(expression(paste('P(|',Delta,mu, V, '| > 0)', ))),
       x=element_blank(),
       y=element_blank()) +
  theme(panel.grid = element_blank(), panel.border = element_blank(),
        strip.background = element_blank(),strip.text.x = element_text(size=5),
        axis.text.y=element_blank(),
        axis.ticks.y=element_blank(),
        plot.background = element_rect(fill = "transparent", colour = NA))
```

```
wrap_plots(list(plot.tr.hi, plot.tr.hi.pp))
```

S4.3.4 GPT-based surprisal alone

```
n_samps <- brms::nsamples(model.hi.surpgpt)

newdata.hi.surpgpt <- rename(newdata.hi.surplstm,
                             surprisal.gpt.Z = surprisal.lstm.Z)
preds.hi.surpgpt <- posterior_epred(model.hi.surpgpt,
                                    newdata=newdata.hi.surpgpt, re.form=NA)
preds.hi.surpgpt_long <- as.data.frame(preds.hi.surpgpt) %>%
  mutate(samp = 1:nrow(preds.hi.surpgpt)) %>%
  pivot_longer(-samp)

preds.hi.surpgpt_long$x <- rep(newdata.hi.surpgpt$x, n_samps)
```

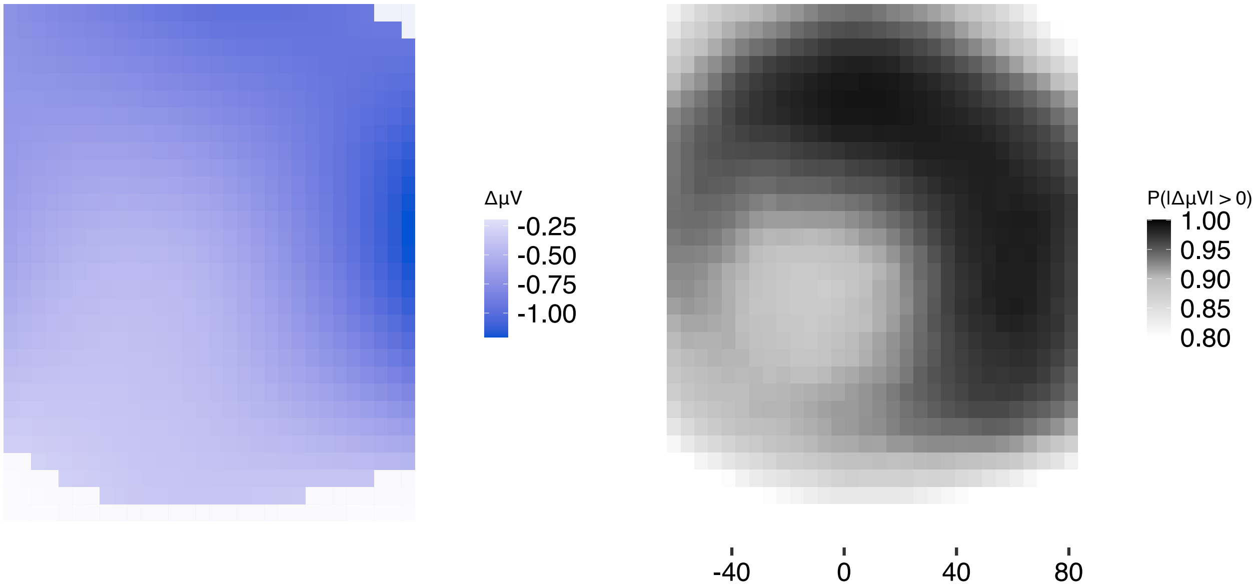

Figure S4: Left: Pair-wise fitted differences of μV for sentences with high vs. sentences with low (+2 vs. -2 std. dev. from the mean) from model with BERT surprisal as predictor alone. Right: Percentage of posterior probability mass of μV mean difference below or above 0.

```
preds.hi.surpgpt_long$y <- rep(newdata.hi.surpgpt$y, n_samps)
preds.hi.surpgpt_long$surprisal <- rep(newdata.hi.surpgpt$surprisal.gpt.Z, n_samps)

summary_hi_surpgpt <- preds.hi.surpgpt_long %>%
  group_by(x,y,samp) %>%
  mutate(diff_surprisal_low_mean = value[surprisal == 2] -
           value[surprisal == -2]) %>%
  ungroup() %>%
  group_by(x,y) %>%
  summarise(PP = ifelse(mean(diff_surprisal_low_mean) > 0,
                        mean(diff_surprisal_low_mean > 0),
                        mean(diff_surprisal_low_mean < 0)),
            mean_diff = mean(diff_surprisal_low_mean)) %>%
  rename(xax = y) %>% #axes need to be changed so that electrodes fit scalp
  rename(yax = x) %>%
  mutate(x = xax) %>%
  mutate(y = yax) %>%
  mutate(x = -x) %>%
  mutate(y = -y)

plot.gpt.hi <- summary_hi_surpgpt %>%
  mutate(sig = ifelse(PP>0.80, 1, 0)) %>%
  ggplot(aes(x=x, y=y, fill=mean_diff, alpha = sig)) +
  geom_tile()+
  scale_fill_gradient2(low = '#0053d3', high = '#b22709', midpoint=0,
                       aesthetics='fill')+
  guides(alpha = "none") +
  theme(panel.grid = element_blank(), panel.border = element_blank(),
        strip.background = element_blank(),strip.text.x = element_text(size=5),
        axis.text.y=element_blank(),
```

```
        axis.ticks.y=element_blank(),
        axis.text.x=element_blank(),
        axis.ticks.x =element_blank(),
        plot.background = element_rect(fill = "transparent", colour = NA))   +
  labs(fill=(expression(paste(Delta, paste(mu, "V")))),
       x = element_blank(), y = element_blank())

plot.gpt.hi.pp <- summary_hi_surpgpt %>%
  ggplot(aes(x=x, y=y, fill=PP)) +
  geom_tile()+
  scale_fill_gradientn(colours = c('white','grey', 'black'),limits = c(0.8, 1),
                       na.value='white' ) +
  labs(fill=(expression(paste('P(|',Delta,mu, V, '| > 0)', ))),
       x=element_blank(),
       y=element_blank()) +
  theme(panel.grid = element_blank(), panel.border = element_blank(),
        strip.background = element_blank(),strip.text.x = element_text(size=5),
        axis.text.y=element_blank(),
        axis.ticks.y=element_blank(),
        plot.background = element_rect(fill = "gptansparent", colour = NA))
```

```
wrap_plots(list(plot.gpt.hi, plot.gpt.hi.pp))
```

S4.3.5 LSTM-based surprisal and Agent preference

```
model.hi.agent.surplstm <- readRDS('../gams_fitted_models/gam_hi_agent_lstm.RDS')

n_samps <- 4000

newdata.hi.agent.surplstm <- expand_grid(
  x = seq(min(hindi.data$x), max(hindi.data$x), length.out = 30),
  y = seq(min(hindi.data$y), max(hindi.data$y),length.out = 30),
  surprisal.lstm.Z = seq(from=-2,to=2, length.out=17),
  Participant = "AD0001",
  Stimulus_Cond = c("1:Abias", "1:nonAbias"),
  trial_number.Z = 0,
) %>%
  separate(Stimulus_Cond, c('Stimulus', 'condition'), sep=':')

preds.hi.agent.surplstm <- posterior_epred(model.hi.agent.surplstm,
                                           newdata=newdata.hi.agent.surplstm,
                                           re.form=NA)

preds.hi.agent.surplstm_long <- as.data.frame(preds.hi.agent.surplstm) %>%
  mutate(samp = 1:nrow(preds.hi.agent.surplstm)) %>%
  pivot_longer(-samp)

preds.hi.agent.surplstm_long$x <- rep(newdata.hi.agent.surplstm$x, n_samps)
preds.hi.agent.surplstm_long$y <- rep(newdata.hi.agent.surplstm$y, n_samps)
preds.hi.agent.surplstm_long$surprisal <-
  rep(newdata.hi.agent.surplstm$surprisal.lstm.Z, n_samps)
```

```r
preds.hi.agent.surplstm_long$condition <-
  rep(newdata.hi.agent.surplstm$condition, n_samps)

summary_hi_agent_surplstm.abias <- preds.hi.agent.surplstm_long %>%
  filter(condition %in% 'Abias') %>%
  group_by(x,y,samp) %>%
  mutate(diff_surprisal_low_mean = value[surprisal == 2] -
           value[surprisal == -2]) %>%
  ungroup() %>%
  group_by(x,y) %>%
  summarise(PP = ifelse(mean(diff_surprisal_low_mean) > 0,
                        mean(diff_surprisal_low_mean > 0),
                        mean(diff_surprisal_low_mean < 0)),
            mean_diff = mean(diff_surprisal_low_mean)) %>%
  mutate(condition = 'Abias') %>%
  rename(xax = y) %>% #axes need to be changed so that electrodes fit scalp
  rename(yax = x) %>%
  mutate(x = xax) %>%
  mutate(y = yax) %>%
  mutate(x = -x) %>%
  mutate(y = -y)

summary_hi_agent_surplstm.nonabias <- preds.hi.agent.surplstm_long %>%
  filter(condition %in% 'nonAbias') %>%
  group_by(x,y,samp) %>%
  mutate(diff_surprisal_low_mean = value[surprisal == 2] -
           value[surprisal == -2]) %>%
  ungroup() %>%
  group_by(x,y) %>%
  summarise(PP = ifelse(mean(diff_surprisal_low_mean) > 0,
                        mean(diff_surprisal_low_mean > 0),
                        mean(diff_surprisal_low_mean < 0)),
            mean_diff = mean(diff_surprisal_low_mean)) %>%
  mutate(condition = 'nonAbias') %>%
  rename(xax = y) %>% #axes need to be changed so that electrodes fit scalp
  rename(yax = x) %>%
  mutate(x = xax) %>%
  mutate(y = yax) %>%
  mutate(x = -x) %>%
  mutate(y = -y)

summary_hi_agent_surplstm <- rbind(summary_hi_agent_surplstm.abias,
                                   summary_hi_agent_surplstm.nonabias)

plot.lstm.agent.surp.hi <- summary_hi_agent_surplstm %>%
  mutate(sig = ifelse(PP>0.80, 1, 0)) %>%
  ggplot(aes(x=x, y=y, fill=mean_diff, alpha = sig)) +
  geom_tile()+
  scale_fill_gradient2(low = '#0053d3', high = '#b22709', midpoint=0,
                       aesthetics='fill')+
  guides(alpha = "none") +
  theme(panel.grid = element_blank(), panel.border = element_blank(),
        strip.background = element_blank(),strip.text.x = element_text(size=5),
```

```r
        axis.text.y=element_blank(),
        axis.ticks.y=element_blank(),
        axis.text.x=element_blank(),
        axis.ticks.x =element_blank(),
        plot.background = element_rect(fill = "transparent", colour = NA)) +
  labs(fill=(expression(paste(Delta, paste(mu, "V")))),
       x = element_blank(), y = element_blank())

plot.lstm.agent.surp.hi.pp <- summary_hi_agent_surplstm %>%
  ggplot(aes(x=x, y=y, fill=PP)) +
  geom_tile()+
  scale_fill_gradientn(colours = c('white','grey', 'black'),
                       limits = c(0.8, 1),
                       na.value='white' ) +
  labs(fill=(expression(paste('P(|',Delta,mu, V, '| > 0)', ))),
       x=element_blank(),
       y=element_blank()) +
  theme(panel.grid = element_blank(), panel.border = element_blank(),
        strip.background = element_blank(),strip.text.x = element_text(size=5),
        axis.text.y=element_blank(),
        axis.ticks.y=element_blank(),
        plot.background = element_rect(fill = "transparent", colour = NA))

summary_hi_agent_surplstm.hi.conds <- preds.hi.agent.surplstm_long %>%
  filter(surprisal == 0) %>%
  group_by(x,y,samp) %>%
  mutate(diff_condition = value[condition == 'Abias'] -
         value[condition == 'nonAbias']) %>%
  ungroup() %>%
  group_by(x,y) %>%
  summarise(PP = ifelse(mean(diff_condition) > 0,
                        mean(diff_condition > 0),
                        mean(diff_condition < 0)),
            mean_diff = mean(diff_condition))  %>%
  rename(xax = y) %>% #axes need to be changed so that electrodes fit scalp
  rename(yax = x) %>%
  mutate(x = xax) %>%
  mutate(y = yax) %>%
  mutate(x = -x) %>%
  mutate(y = -y)

plot.lstm.agent.hi.cond <- summary_hi_agent_surplstm.hi.conds  %>%
  mutate(sig = ifelse(PP>0.80, 1, 0)) %>%
  ggplot(aes(x=x, y=y, fill=mean_diff, alpha = sig)) +
  geom_tile()+
  scale_fill_gradient2(low = '#0053d3', high = '#b22709', midpoint=0,
                       aesthetics='fill')+
  guides(alpha = "none") +
  theme(panel.grid = element_blank(), panel.border = element_blank(),
        strip.background = element_blank(),strip.text.x = element_text(size=5),
        axis.text.y=element_blank(),
        axis.ticks.y=element_blank(),
        axis.text.x=element_blank(),
```

```
        axis.ticks.x =element_blank(),
        plot.background = element_rect(fill = "transparent", colour = NA))   +
  labs(fill=(expression(paste(Delta, paste(mu, "V")))),
       x = element_blank(), y = element_blank())

plot.lstm.agent.hi.cond.pp <- summary_hi_agent_surplstm.hi.conds  %>%
  ggplot(aes(x=x, y=y, fill=PP)) +
  geom_tile()+
  scale_fill_gradientn(colours = c('white','grey', 'black'),
                       limits = c(0.8, 1), na.value='white' ) +
  labs(fill=(expression(paste('P(|',Delta,mu, V, '| > 0)', ))),
       x=element_blank(),
       y=element_blank()) +
  theme(panel.grid = element_blank(), panel.border = element_blank(),
        strip.background = element_blank(),strip.text.x = element_text(size=5),
        axis.text.y=element_blank(),
        axis.ticks.y=element_blank(),
        plot.background = element_rect(fill = "transparent", colour = NA))
```

```
wrap_plots(list(plot.lstm.agent.hi.cond, plot.lstm.agent.surp.hi,
                plot.lstm.agent.hi.cond.pp,plot.lstm.agent.surp.hi.pp))
```

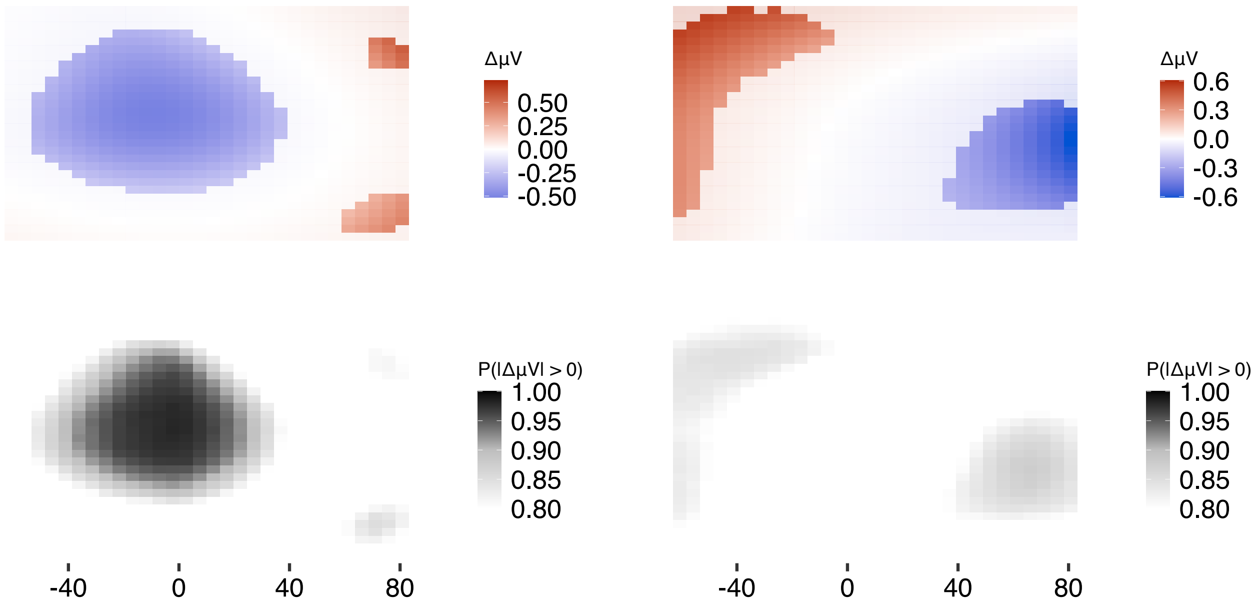

Figure S5: Left: Pair-wise fitted differences of μV for sentences with vs. sentences without the predicted reanalysis from model with LSTM surprisal as well as agent preference predictor. Right: Percentage of posterior probability mass of μV mean difference below or above 0.

S4.3.6 BERT-based surprisal and Agent preference

```
n_samps <- 4000

newdata.hi.agent.surptr <- newdata.hi.agent.surplstm %>%
```

```
  rename(surprisal.tr.Z = surprisal.lstm.Z)
preds.hi.agent.surptr <- posterior_epred(model.hi.agent.surptr,
                                         newdata=newdata.hi.agent.surptr,
                                         re.form=NA)

preds.hi.agent.surptr_long <- as.data.frame(preds.hi.agent.surptr) %>%
  mutate(samp = 1:nrow(preds.hi.agent.surptr)) %>%
  pivot_longer(-samp)

preds.hi.agent.surptr_long$x <- rep(newdata.hi.agent.surptr$x, n_samps)
preds.hi.agent.surptr_long$y <- rep(newdata.hi.agent.surptr$y, n_samps)
preds.hi.agent.surptr_long$surprisal <-
  rep(newdata.hi.agent.surptr$surprisal.tr.Z, n_samps)
preds.hi.agent.surptr_long$condition <-
  rep(newdata.hi.agent.surptr$condition, n_samps)

summary_hi_agent_surptr <- preds.hi.agent.surptr_long %>%
  filter(condition %in% 'Abias') %>%
  group_by(x,y,samp) %>%
  mutate(diff_surprisal_low_mean = value[surprisal == 2] -
         value[surprisal == -2]) %>%
  ungroup() %>%
  group_by(x,y) %>%
  summarise(PP = ifelse(mean(diff_surprisal_low_mean) > 0,
                        mean(diff_surprisal_low_mean > 0),
                        mean(diff_surprisal_low_mean < 0)),
          mean_diff = mean(diff_surprisal_low_mean)) %>%
  mutate('condition' = 'Abias') %>%
  rename(xax = y) %>% #axes need to be changed so that electrodes fit scalp
  rename(yax = x) %>%
  mutate(x = xax) %>%
  mutate(y = yax) %>%
  mutate(x = -x) %>%
  mutate(y = -y)

plot.tr.agent.surp.hi <- summary_hi_agent_surptr %>%
  mutate(sig = ifelse(PP>0.80, 1, 0)) %>%
  ggplot(aes(x=x, y=y, fill=mean_diff, alpha = sig)) +
  geom_tile()+
  scale_fill_gradient2(low = '#0053d3', high = '#b22709', midpoint=0,
                       aesthetics='fill')+
  guides(alpha = "none") +
  theme(panel.grid = element_blank(), panel.border = element_blank(),
        strip.background = element_blank(),strip.text.x = element_text(size=5),
        axis.text.y=element_blank(),
        axis.ticks.y=element_blank(),
        axis.text.x = element_blank(),
        axis.ticks.x = element_blank(),
        plot.background = element_rect(fill = "transparent", colour = NA)) +
  labs(fill = (expression(paste(Delta, paste(mu, "V")))),
       x= element_blank(),
       y = element_blank())
```

```
plot.tr.agent.surp.hi.pp <- summary_hi_agent_surptr %>%
  ggplot(aes(x=x, y=y, fill=PP)) +
  geom_tile()+
  scale_fill_gradientn(colours = c('white','grey', 'black'),limits = c(0.8, 1),
                       na.value='white' ) +
  labs(fill=(expression(paste('P(|',Delta,mu, V, '| > 0)', ))),
       x=element_blank(),
       y=element_blank()) +
  theme(panel.grid = element_blank(), panel.border = element_blank(),
        strip.background = element_blank(),strip.text.x = element_text(size=5),
        axis.text.y=element_blank(),
        axis.text.x=element_blank(),
        axis.ticks.y=element_blank(),
        axis.ticks.x=element_blank(),
        plot.background = element_rect(fill = "transparent", colour = NA))

summary_hi_agent_surptr.condition <- preds.hi.agent.surptr_long %>%
  filter(surprisal == 0) %>%
  group_by(x,y,samp) %>%
  mutate(diff_condition = value[condition == 'Abias'] -
           value[condition == 'nonAbias']) %>%
  ungroup() %>%
  group_by(x,y) %>%
  summarise(PP = ifelse(mean(diff_condition) > 0,
                        mean(diff_condition > 0),
                        mean(diff_condition < 0)),
            mean_diff = mean(diff_condition))  %>%
  rename(xax = y) %>% #axes need to be changed so that electrodes fit scalp
  rename(yax = x) %>%
  mutate(x = xax) %>%
  mutate(y = yax) %>%
  mutate(x = -x) %>%
  mutate(y = -y)

plot.tr.agent.hi.cond <- summary_hi_agent_surptr.condition %>%
  mutate(sig = ifelse(PP>0.80, 1, 0)) %>%
  ggplot(aes(x=x, y=y, fill=mean_diff, alpha = sig)) +
  geom_tile()+
  scale_fill_gradient2(low = '#0053d3', high = '#b22709', midpoint=0,
                       aesthetics='fill')+
  guides(alpha = "none") +
  theme(panel.grid = element_blank(), panel.border = element_blank(),
        strip.background = element_blank(),strip.text.x = element_text(size=5),
        axis.text.y=element_blank(),
        axis.ticks.y=element_blank(),
        axis.text.x=element_blank(),
        axis.ticks.x =element_blank(),
        plot.background = element_rect(fill = "transparent", colour = NA))   +
  labs(fill=(expression(paste(Delta, paste(mu, "V")))),
       x = element_blank(), y = element_blank())

plot.tr.agent.hi.cond.pp <- summary_hi_agent_surptr.condition %>%
  ggplot(aes(x=x, y=y, fill=PP)) +
```

```
  geom_tile()+
  scale_fill_gradientn(colours = c('white','grey', 'black'),limits = c(0.8, 1),
                       na.value='white' ) +
  #scale_fill_gradient2(low = 'white' , high = 'black', limits = c(0.8, 1)) +
  labs(fill=(expression(paste('P(|',Delta,mu, V, '| > 0)', ))),
       x=element_blank(),
       y=element_blank()) +
  theme(panel.grid = element_blank(), panel.border = element_blank(),
        strip.background = element_blank(),strip.text.x = element_text(size=5),
        axis.text.y=element_blank(),
        axis.text.x=element_blank(),
        axis.ticks.y=element_blank(),
        axis.ticks.x=element_blank(),
        plot.background = element_rect(fill = "transparent", colour = NA))
```

```
wrap_plots(list(plot.tr.agent.hi.cond, plot.tr.agent.surp.hi,
                plot.tr.agent.hi.cond.pp,plot.tr.agent.surp.hi.pp))
```

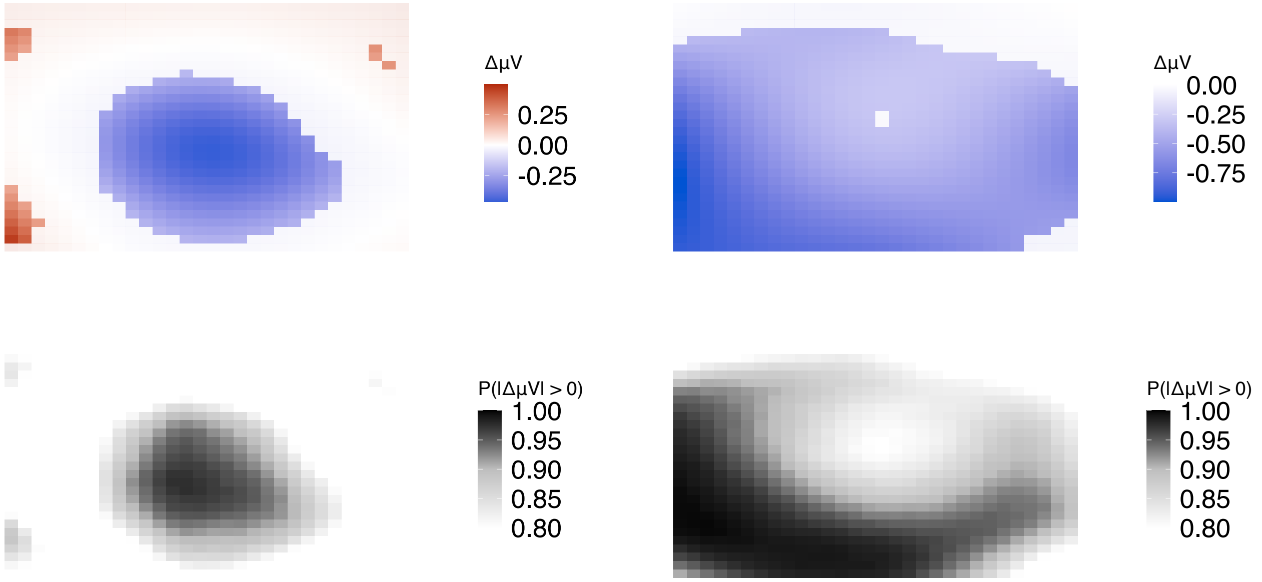

Figure S6: Left: Pair-wise fitted differences of μV for sentences with vs. sentences without the predicted reanalysis from model with BERT surprisal as well as agent preference predictor. Right: Percentage of posterior probability mass of μV mean difference below or above 0.

S4.3.7 GPT-based surprisal and Agent preference

```
n_samps <- 4000

newdata.hi.agent.surpgpt <- newdata.hi.agent.surplstm %>%
  rename(surprisal.gpt.Z = surprisal.lstm.Z)
preds.hi.agent.surpgpt <- posterior_epred(model.hi.agent.surpgpt,
                                          newdata=newdata.hi.agent.surpgpt,
                                          re.form=NA)
```

```
preds.hi.agent.surpgpt_long <- as.data.frame(preds.hi.agent.surpgpt) %>%
  mutate(samp = 1:nrow(preds.hi.agent.surpgpt)) %>%
  pivot_longer(-samp)

preds.hi.agent.surpgpt_long$x <- rep(newdata.hi.agent.surpgpt$x, n_samps)
preds.hi.agent.surpgpt_long$y <- rep(newdata.hi.agent.surpgpt$y, n_samps)
preds.hi.agent.surpgpt_long$surprisal <-
  rep(newdata.hi.agent.surpgpt$surprisal.gpt.Z, n_samps)
preds.hi.agent.surpgpt_long$condition <-
  rep(newdata.hi.agent.surpgpt$condition, n_samps)

summary_hi_agent_surpgpt <- preds.hi.agent.surpgpt_long %>%
  filter(condition %in% 'Abias') %>%
  group_by(x,y,samp) %>%
  mutate(diff_surprisal_low_mean = value[surprisal == 2] -
           value[surprisal == -2]) %>%
  ungroup() %>%
  group_by(x,y) %>%
  summarise(PP = ifelse(mean(diff_surprisal_low_mean) > 0,
                        mean(diff_surprisal_low_mean > 0),
                        mean(diff_surprisal_low_mean < 0)),
            mean_diff = mean(diff_surprisal_low_mean)) %>%
  mutate('condition' = 'Abias') %>%
  rename(xax = y) %>% #axes need to be changed so that electrodes fit scalp
  rename(yax = x) %>%
  mutate(x = xax) %>%
  mutate(y = yax) %>%
  mutate(x = -x) %>%
  mutate(y = -y)

plot.gpt.agent.surp.hi <- summary_hi_agent_surpgpt %>%
  mutate(sig = ifelse(PP>0.80, 1, 0)) %>%
  ggplot(aes(x=x, y=y, fill=mean_diff, alpha = sig)) +
  geom_tile()+
  scale_fill_gradient2(low = '#0053d3', high = '#b22709', midpoint=0,
                       aesthetics='fill')+
  guides(alpha = "none") +
  theme(panel.grid = element_blank(), panel.border = element_blank(),
        strip.background = element_blank(),strip.text.x = element_text(size=5),
        axis.text.y=element_blank(),
        axis.ticks.y=element_blank(),
        axis.text.x = element_blank(),
        axis.ticks.x = element_blank(),
        plot.background = element_rect(fill = "transparent", colour = NA)) +
  labs(fill = (expression(paste(Delta, paste(mu, "V")))),
       x= element_blank(),
       y = element_blank())

plot.gpt.agent.surp.hi.pp <- summary_hi_agent_surpgpt %>%
  ggplot(aes(x=x, y=y, fill=PP)) +
  geom_tile()+
  scale_fill_gradientn(colours = c('white','grey', 'black'),limits = c(0.8, 1),
                       na.value='white' ) +
```

```
  labs(fill=(expression(paste('P(|',Delta,mu, V, '| > 0)', ))),
       x=element_blank(),
       y=element_blank()) +
  theme(panel.grid = element_blank(), panel.border = element_blank(),
        strip.background = element_blank(),strip.text.x = element_text(size=5),
        axis.text.y=element_blank(),
        axis.text.x=element_blank(),
        axis.ticks.y=element_blank(),
        axis.ticks.x=element_blank(),
        plot.background = element_rect(fill = "transparent", colour = NA))

summary_hi_agent_surpgpt.condition <- preds.hi.agent.surpgpt_long %>%
  filter(surprisal == 0) %>%
  group_by(x,y,samp) %>%
  mutate(diff_condition = value[condition == 'Abias'] -
           value[condition == 'nonAbias']) %>%
  ungroup() %>%
  group_by(x,y) %>%
  summarise(PP = ifelse(mean(diff_condition) > 0,
                        mean(diff_condition > 0),
                        mean(diff_condition < 0)),
            mean_diff = mean(diff_condition)) %>%
  rename(xax = y) %>% #axes need to be changed so that electrodes fit scalp
  rename(yax = x) %>%
  mutate(x = xax) %>%
  mutate(y = yax) %>%
  mutate(x = -x) %>%
  mutate(y = -y)

plot.gpt.agent.hi.cond <- summary_hi_agent_surpgpt.condition %>%
  mutate(sig = ifelse(PP>0.80, 1, 0)) %>%
  ggplot(aes(x=x, y=y, fill=mean_diff, alpha = sig)) +
  geom_tile()+
  scale_fill_gradient2(low = '#0053d3', high = '#b22709', midpoint=0,
                       aesthetics='fill')+
  guides(alpha = "none") +
  theme(panel.grid = element_blank(), panel.border = element_blank(),
        strip.background = element_blank(),strip.text.x = element_text(size=5),
        axis.text.y=element_blank(),
        axis.ticks.y=element_blank(),
        axis.text.x=element_blank(),
        axis.ticks.x =element_blank(),
        plot.background = element_rect(fill = "transparent", colour = NA))   +
  labs(fill=(expression(paste(Delta, paste(mu, "V")))),
       x = element_blank(), y = element_blank())

plot.gpt.agent.hi.cond.pp <- summary_hi_agent_surpgpt.condition %>%
  ggplot(aes(x=x, y=y, fill=PP)) +
  geom_tile()+
  scale_fill_gradientn(colours = c('white','grey', 'black'),limits = c(0.8, 1),
                       na.value='white' ) +
  #scale_fill_gradient2(low = 'white' , high = 'black', limits = c(0.8, 1)) +
  labs(fill=(expression(paste('P(|',Delta,mu, V, '| > 0)', ))),
```

```
      x=element_blank(),
      y=element_blank()) +
  theme(panel.grid = element_blank(), panel.border = element_blank(),
        strip.background = element_blank(),strip.text.x = element_text(size=5),
        axis.text.y=element_blank(),
        axis.text.x=element_blank(),
        axis.ticks.y=element_blank(),
        axis.ticks.x=element_blank(),
        plot.background = element_rect(fill = "transparent", colour = NA))
```

```
wrap_plots(list(plot.gpt.agent.hi.cond, plot.gpt.agent.surp.hi,
                plot.gpt.agent.hi.cond.pp,plot.gpt.agent.surp.hi.pp))
```

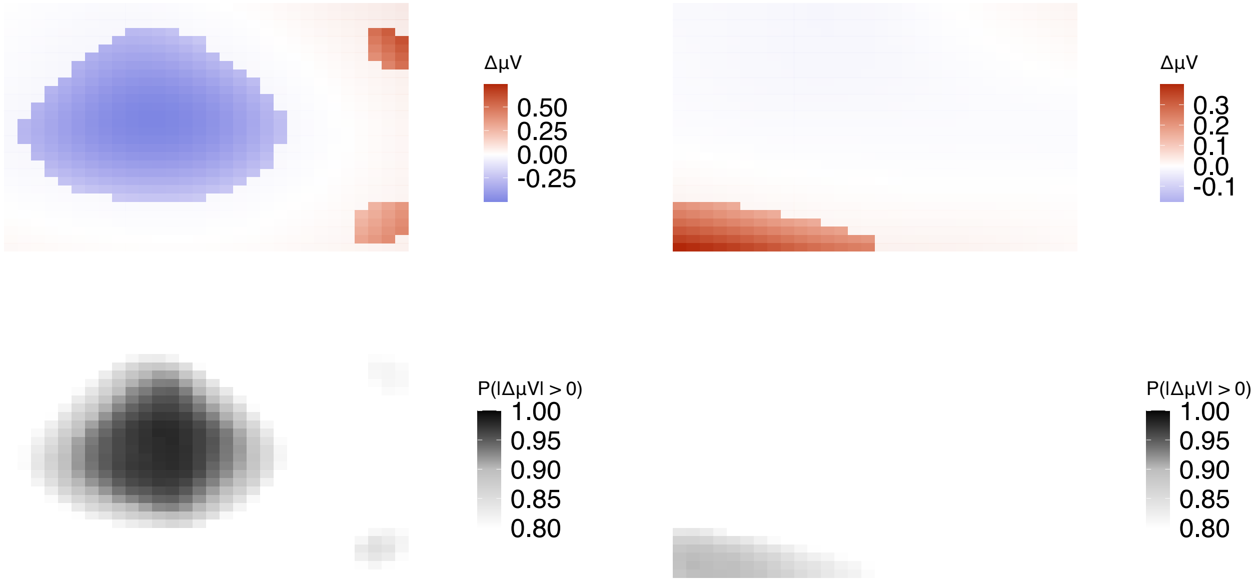

Figure S7: Left: Pair-wise fitted differences of μV for sentences with vs. sentences without the predicted reanalysis from model with GPT-2 surprisal as well as agent preference predictor. Right: Percentage of posterior probability mass of μV mean difference below or above 0.

S4.3.8 Strongest effect summary

Table S1: Hindi: This table contains the lowest mean delta microVolt (i.e. strongest effect size across the scalp, blue in the plots above) and the maximum proportion of the posterior distribution that is lower than 0 for the highest-ranked model, agent preference + bert surprisal.

predictor	$min(mean(\Delta\mu V))$	$max(P\Delta\mu V < 0)$
surprisal BERT	-0.99	0.998
agent preference condition	-0.46	0.975

5 Analysis 2: Predicting ERPs for Basque

S5: Study 2: Predicting ERPs (Basque)*

Eva Huber Sebastian Sauppe Arrate Isasi-Isasmendi
Ina Bornkessel-Schlesewsky Paola Merlo Balthasar Bickel

2023-08-29

Contents

S5 Basque **1**
S5.1 Models . . . 1
S5.2 Model comparison with stacking . . . 14
S5.3 Model predictions . . . 15

S6 References **32**

REPRODUCIBILITY STATEMENT: All of the models included here were fitted on an M1 processor using R Version 4.2.2 and brms version 2.14.0.

S5 Basque

S5.1 Models

We model the EEG results in micro-volt (`mV` below). For this, we fit Bayesian Generalised Additive Models in `brms` with different predictors and compare models by means of stacking in order to find the model with highest predictive performance. In the model below `condition` refers to whether or not we expect a reanalysis (see main text).

We check convergence by ensuring that Effective Sample Size $ESS > 100$ and diagnostic $\hat{R} < 1.05$. Additionally, we perform posterior predictive checks to compare the predicted values to the observed data.

S5.1.1 Agent preference alone

```
basque.data <- readRDS('../results/basque_mv_surprisal_new.RDS')

if (!file.exists('../gams_fitted_models/gam_eu_agent.RDS')){
  prior.brms <-  c(
    prior(normal(0,1), class = b),
    prior(exponential(1), class = sigma)
  )
  model.agent <-  brm(mV
                      ~ 1
```

*This document was `rmarkdown::render`'ed from an `R` script 'main_scripts/Basque_analysis2.R' available at https://osf.io/hbj67.

```
                      + Condition
                      + trial_number.Z
                      + t2(x, y, by = Condition, k = 10)
                      + t2(x, y, Participant, bs = "re")
                      + t2(x, y, Stimulus, bs = "re"),
                      data = basque.data,
                      sample_prior = TRUE,
                      prior = prior.brms,
                      chains = 4, iter = 2000, cores = 4
  )
  saveRDS(model.agent, '../gams_fitted_models/gam_eu_agent.RDS')
}
model.eu.agent <- readRDS('../gams_fitted_models/gam_eu_agent.RDS')
summary(model.eu.agent)
```

```
##  Family: gaussian
##   Links: mu = identity; sigma = identity
## Formula: mV ~ 1 + condition + trial_number.Z + t2(x, y, by = condition, k = 10) + t2(x, y, Participan
##    Data: basque.data (Number of observations: 68600)
## Samples: 4 chains, each with iter = 2000; warmup = 1000; thin = 1;
##          total post-warmup samples = 4000
##
## Smooth Terms:
##                                 Estimate Est.Error l-95% CI u-95% CI Rhat Bulk_ESS Tail_ESS
## sds(t2xyconditionAbias_1)           2.05      1.88     0.05     6.92 1.00     1464     2028
## sds(t2xyconditionAbias_2)          12.21      3.63     6.97    21.20 1.00     2956     3143
## sds(t2xyconditionAbias_3)          13.74      4.10     7.84    23.76 1.00     2563     2761
## sds(t2xyconditionnonAbias_1)        3.73      2.41     0.47     9.59 1.01      956     1516
## sds(t2xyconditionnonAbias_2)       10.82      3.37     5.85    18.77 1.00     2471     3082
## sds(t2xyconditionnonAbias_3)       12.50      3.74     7.09    21.46 1.00     2360     2794
## sds(t2xyParticipant_1)              0.48      0.12     0.26     0.73 1.00     1662     2253
## sds(t2xyStimulus_1)                 0.09      0.06     0.00     0.24 1.00     2448     1654
##
## Population-Level Effects:
##                             Estimate Est.Error l-95% CI u-95% CI Rhat Bulk_ESS Tail_ESS
## Intercept                      -2.34      0.11    -2.55    -2.11 1.00     3740     3141
## condition2                      0.61      0.15     0.31     0.89 1.00     2690     3002
## trial_number.Z                  0.60      0.04     0.52     0.67 1.00    13557     2617
## t2xy:conditionAbias_1          -1.32      0.14    -1.59    -1.02 1.00     3128     2652
## t2xy:conditionAbias_2          -0.06      0.13    -0.31     0.20 1.00     3810     3365
## t2xy:conditionAbias_3           0.08      0.21    -0.32     0.48 1.00     3593     3050
## t2xy:conditionnonAbias_1       -1.32      0.13    -1.58    -1.05 1.00     2723     2656
## t2xy:conditionnonAbias_2        0.12      0.11    -0.11     0.35 1.00     2467     2390
## t2xy:conditionnonAbias_3       -0.02      0.17    -0.35     0.32 1.00     3240     2991
##
## Family Specific Parameters:
##       Estimate Est.Error l-95% CI u-95% CI Rhat Bulk_ESS Tail_ESS
## sigma    10.07      0.03    10.02    10.12 1.00     5959     2238
##
## Samples were drawn using sampling(NUTS). For each parameter, Bulk_ESS
## and Tail_ESS are effective sample size measures, and Rhat is the potential
## scale reduction factor on split chains (at convergence, Rhat = 1).
```

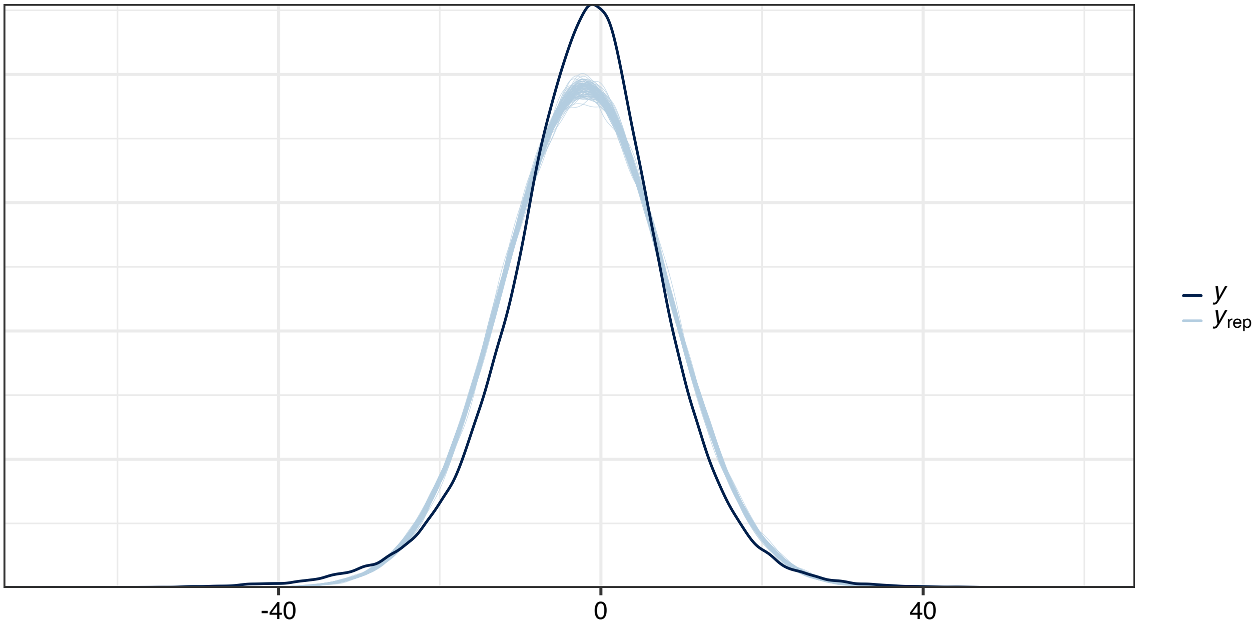

S5.1.2 Surprisal alone

LSTM-based surprisal:

```
if (!file.exists('../gams_fitted_models/gam_eu_lstm.RDS')){
  prior.brms <-  c(
    prior(normal(0,1), class = b),
    prior(exponential(1), class = sigma)
  )

  model.surprisal.lstm <-  brm(mV
                               ~ 1
                               + trial_number.Z
                               + surprisal.lstm.Z
                               + t2(x, y, by = surprisal.lstm.Z, k = 10)
                               + t2(x, y, Participant, bs = "re")
                               + t2(x, y, Stimulus, bs = "re"),
                               prior = prior.brms,
                               sample_prior = TRUE,
                               data = basque.data,
                               chains = 4, iter = 2000, cores = 4
  )
  saveRDS(model.surprisal.lstm,
          '../gams_fitted_models/gam_eu_lstm.RDS')
}
model.eu.surp.lstm <- readRDS('../gams_fitted_models/gam_eu_lstm.RDS')

summary(model.eu.surp.lstm)
```

```
##  Family: gaussian
##   Links: mu = identity; sigma = identity
## Formula: mV ~ 1 + trial_number.Z + surprisal.lstm.Z + t2(x, y, by = surprisal.lstm.Z, k = 10) + t2(x
```

```
##    Data: basque.data (Number of observations: 68600)
## Samples: 4 chains, each with iter = 2000; warmup = 1000; thin = 1;
##          total post-warmup samples = 4000
##
## Smooth Terms:
##                                Estimate Est.Error l-95% CI u-95% CI Rhat Bulk_ESS Tail_ESS
## sds(t2xysurprisal.lstm.Z_1)        0.56      0.50     0.02     1.80 1.00     2076     2554
## sds(t2xysurprisal.lstm.Z_2)        1.28      1.05     0.05     3.87 1.00     1783     2052
## sds(t2xysurprisal.lstm.Z_3)        0.97      0.93     0.03     3.46 1.00     2391     2098
## sds(t2xyParticipant_1)             0.46      0.12     0.25     0.71 1.01     1371     1578
## sds(t2xyStimulus_1)                0.09      0.07     0.00     0.25 1.00     2958     1878
##
## Population-Level Effects:
##                            Estimate Est.Error l-95% CI u-95% CI Rhat Bulk_ESS Tail_ESS
## Intercept                     -2.07      0.04    -2.14    -2.00 1.00     9619     2764
## trial_number.Z                 0.59      0.04     0.52     0.67 1.00    10395     3135
## surprisal.lstm.Z               0.07      0.70    -1.28     1.45 1.00     4663     2628
## t2xy:surprisal.lstm.Z_1        0.05      0.68    -1.29     1.37 1.00     4691     2558
## t2xy:surprisal.lstm.Z_2        0.14      0.05     0.05     0.23 1.00     6972     3057
## t2xy:surprisal.lstm.Z_3       -0.04      0.05    -0.13     0.05 1.00     5699     2760
## t2xy:surprisal.lstm.Z_4        0.01      0.06    -0.10     0.12 1.00     7306     2938
##
## Family Specific Parameters:
##       Estimate Est.Error l-95% CI u-95% CI Rhat Bulk_ESS Tail_ESS
## sigma    10.17      0.03    10.11    10.22 1.00     6894     2248
##
## Samples were drawn using sampling(NUTS). For each parameter, Bulk_ESS
## and Tail_ESS are effective sample size measures, and Rhat is the potential
## scale reduction factor on split chains (at convergence, Rhat = 1).
```

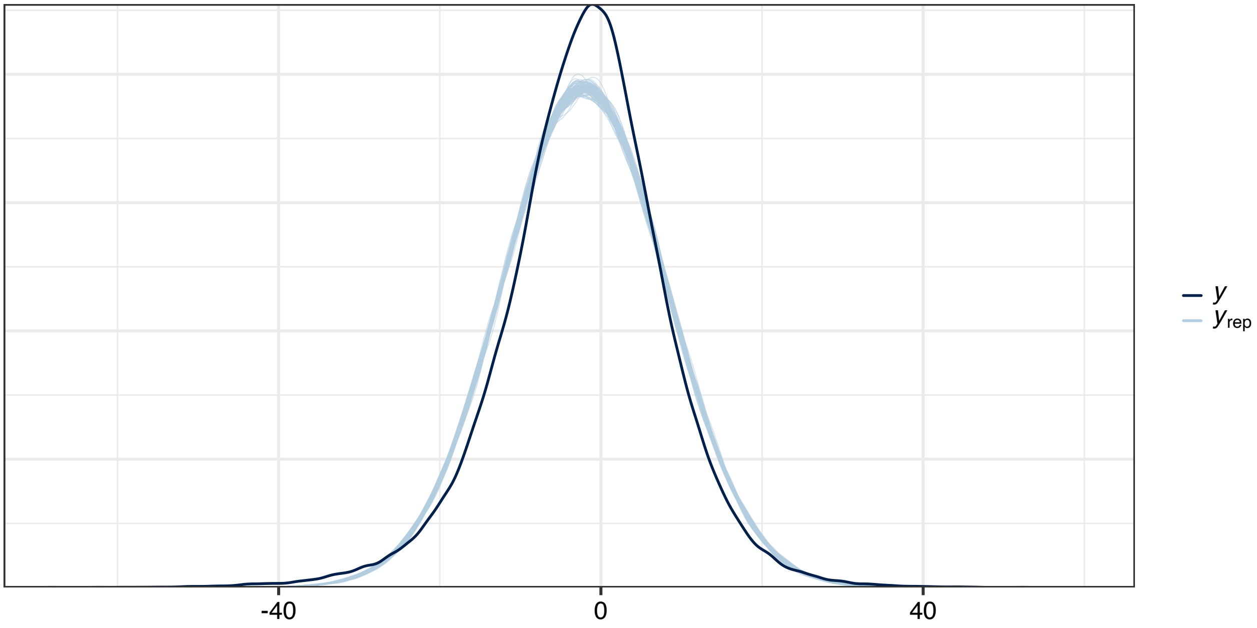

RoBERTa-based surprisal

```r
if (!file.exists('../gams_fitted_models/gam_eu_roberta.RDS')){
  prior.brms <-  c(
    prior(normal(0,1), class = b),
    prior(exponential(1), class = sigma)
  )

  model.surprisal.tr <-  brm(mV
                              ~ 1
                              + trial_number.Z
                              + surprisal.tr.Z
                              + t2(x, y, by = surprisal.tr.Z, k = 10)
                              + t2(x, y, Participant, bs = "re")
                              + t2(x, y, Stimulus, bs = "re"),
                              prior = prior.brms,
                              sample_prior = TRUE,
                              data = basque.data,
                              chains = 4, iter = 2000, cores = 4
  )
  saveRDS(model.surprisal.tr,
          '../gams_fitted_models/gam_eu_roberta.RDS')
}
model.eu.surp.roberta <- readRDS('../gams_fitted_models/gam_eu_roberta.RDS')
summary(model.eu.surp.roberta)
```

```
##  Family: gaussian
##   Links: mu = identity; sigma = identity
## Formula: mV ~ 1 + trial_number.Z + surprisal.tr.Z + t2(x, y, by = surprisal.tr.Z, k = 10) + t2(x, y,
##    Data: basque.data (Number of observations: 68600)
## Samples: 4 chains, each with iter = 2000; warmup = 1000; thin = 1;
##          total post-warmup samples = 4000
##
## Smooth Terms:
##                            Estimate Est.Error l-95% CI u-95% CI Rhat Bulk_ESS Tail_ESS
## sds(t2xysurprisal.tr.Z_1)      0.54      0.50     0.02     1.84 1.00     1852     2695
## sds(t2xysurprisal.tr.Z_2)      1.12      1.04     0.04     3.84 1.00     2393     2657
## sds(t2xysurprisal.tr.Z_3)      1.02      0.95     0.03     3.43 1.00     2256     2398
## sds(t2xyParticipant_1)         0.46      0.12     0.25     0.70 1.00     1847     2311
## sds(t2xyStimulus_1)            0.08      0.06     0.00     0.24 1.00     2296     2350
##
## Population-Level Effects:
##                        Estimate Est.Error l-95% CI u-95% CI Rhat Bulk_ESS Tail_ESS
## Intercept                 -2.07      0.04    -2.15    -1.99 1.00    10847     2439
## trial_number.Z             0.60      0.04     0.52     0.68 1.00    13640     2513
## surprisal.tr.Z            -0.16      0.73    -1.61     1.28 1.00     5454     3263
## t2xy:surprisal.tr.Z_1     -0.14      0.71    -1.54     1.26 1.00     5543     3236
## t2xy:surprisal.tr.Z_2      0.08      0.05    -0.01     0.17 1.00     7206     3115
## t2xy:surprisal.tr.Z_3     -0.01      0.04    -0.10     0.07 1.00     7821     3252
## t2xy:surprisal.tr.Z_4      0.04      0.06    -0.07     0.15 1.00     8455     3028
##
## Family Specific Parameters:
##       Estimate Est.Error l-95% CI u-95% CI Rhat Bulk_ESS Tail_ESS
## sigma    10.17      0.03    10.11    10.22 1.00     6562     2531
##
```

```
## Samples were drawn using sampling(NUTS). For each parameter, Bulk_ESS
## and Tail_ESS are effective sample size measures, and Rhat is the potential
## scale reduction factor on split chains (at convergence, Rhat = 1).
```

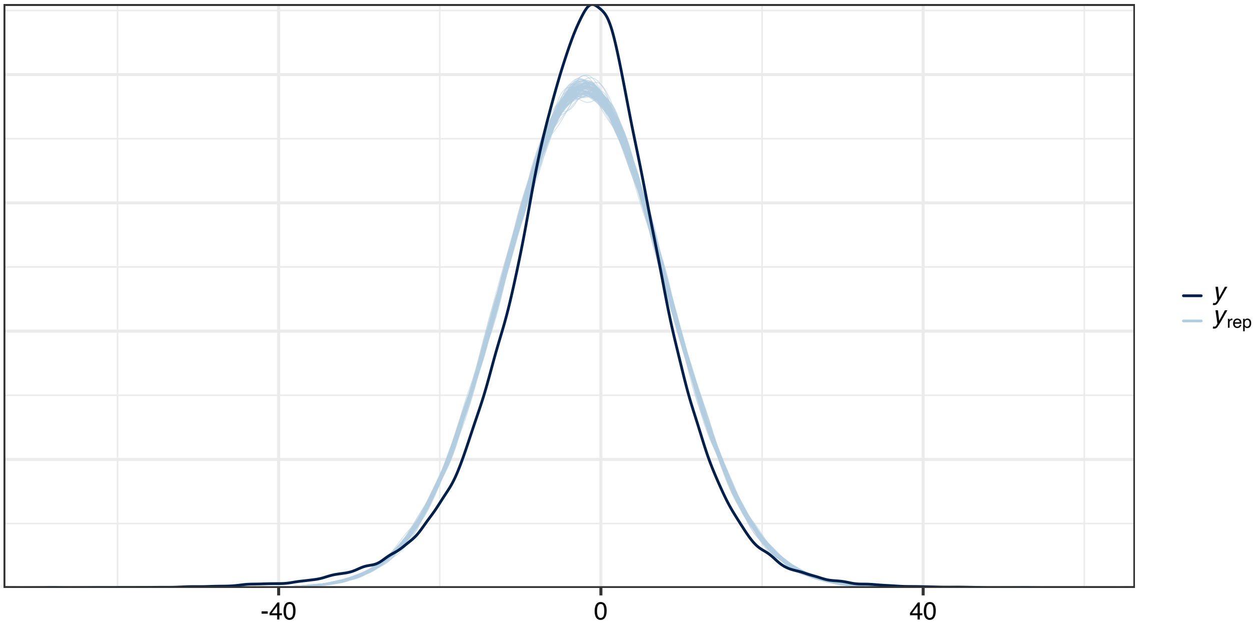

GPT-based surprisal:

```
if (!file.exists('../gams_fitted_models/gam_eu_gpt.RDS')){
  prior.brms <-  c(
    prior(normal(0,1), class = b),
    prior(exponential(1), class = sigma)
  )
  model.surprisal.gpt <-  brm(mV
                              ~ 1
                              + trial_number.Z
                              + surprisal.gpt.Z
                              + t2(x, y, by = surprisal.gpt.Z, k = 10)
                              + t2(x, y, Participant, bs = "re")
                              + t2(x, y, Stimulus, bs = "re"),
                              prior = prior.brms,
                              sample_prior = TRUE,
                              data = basque.data,
                              chains = 4, iter = 2000, cores = 4
  )
  saveRDS(model.surprisal.tr,
          '../gams_fitted_models/gam_eu_gpt.RDS')
}
model.eu.surp.gpt <- readRDS('../gams_fitted_models/gam_eu_gpt.RDS')
summary(model.eu.surp.gpt)
```

```
##  Family: gaussian
##   Links: mu = identity; sigma = identity
## Formula: mV ~ 1 + trial_number.Z + surprisal.gpt.Z + t2(x, y, by = surprisal.gpt.Z, k = 10) + t2(x, 
##    Data: basque.data (Number of observations: 68600)
```

```
## Samples: 4 chains, each with iter = 2000; warmup = 1000; thin = 1;
##          total post-warmup samples = 4000
##
## Smooth Terms:
##                              Estimate Est.Error l-95% CI u-95% CI Rhat Bulk_ESS Tail_ESS
## sds(t2xysurprisal.gpt.Z_1)       0.52      0.49     0.02     1.80 1.00     2530     2471
## sds(t2xysurprisal.gpt.Z_2)       1.08      0.99     0.04     3.71 1.00     2447     2436
## sds(t2xysurprisal.gpt.Z_3)       1.80      1.27     0.15     5.19 1.00     1844     1979
## sds(t2xyParticipant_1)           0.46      0.12     0.24     0.71 1.00     1534     1837
## sds(t2xyStimulus_1)              0.09      0.06     0.00     0.24 1.00     3184     2018
##
## Population-Level Effects:
##                          Estimate Est.Error l-95% CI u-95% CI Rhat Bulk_ESS Tail_ESS
## Intercept                   -2.07      0.04    -2.15    -2.00 1.00    11046     2869
## trial_number.Z               0.59      0.04     0.52     0.67 1.00    11606     2511
## surprisal.gpt.Z             -0.04      0.72    -1.46     1.37 1.00     5872     3240
## t2xy:surprisal.gpt.Z_1      -0.04      0.70    -1.42     1.34 1.00     5891     3296
## t2xy:surprisal.gpt.Z_2       0.10      0.05     0.01     0.20 1.00     7264     3391
## t2xy:surprisal.gpt.Z_3       0.00      0.04    -0.09     0.09 1.00     7976     3282
## t2xy:surprisal.gpt.Z_4       0.00      0.06    -0.11     0.12 1.00     7503     3123
##
## Family Specific Parameters:
##       Estimate Est.Error l-95% CI u-95% CI Rhat Bulk_ESS Tail_ESS
## sigma    10.17      0.03    10.12    10.22 1.00     6110     2502
##
## Samples were drawn using sampling(NUTS). For each parameter, Bulk_ESS
## and Tail_ESS are effective sample size measures, and Rhat is the potential
## scale reduction factor on split chains (at convergence, Rhat = 1).
```

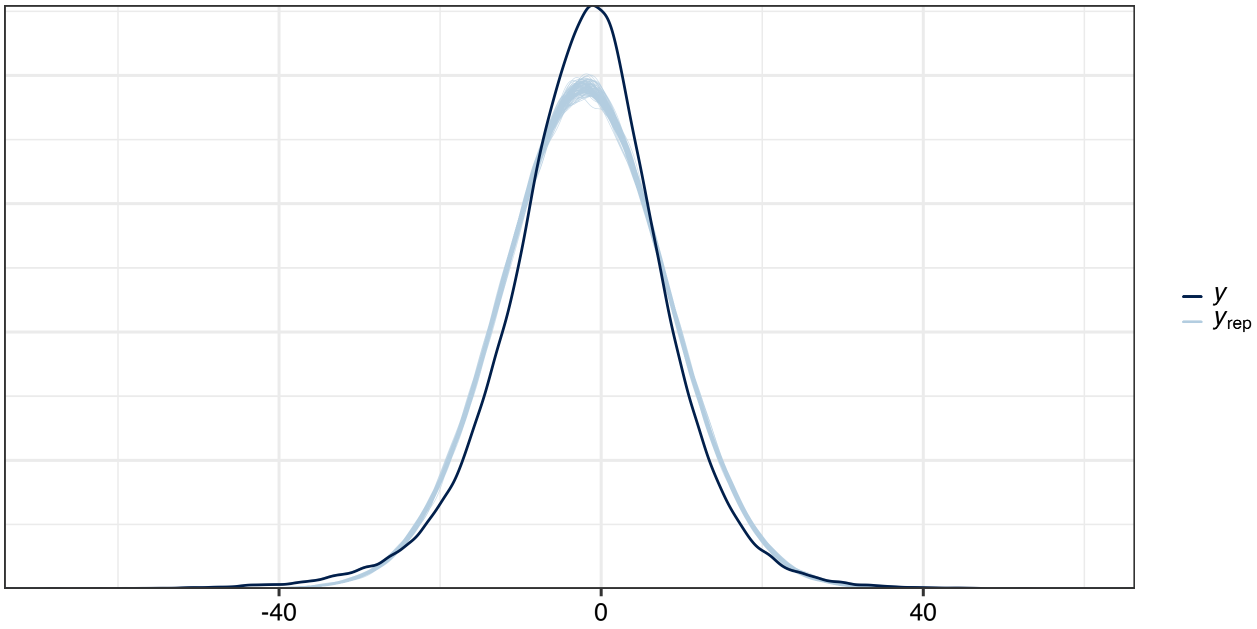

S5.1.3 Agent preference and surprisal

LSTM-based surprisal

```
if (!file.exists('../gams_fitted_models/gam_eu_agent_lstm.RDS')){
  prior.brms <-  c(
    prior(normal(0,1), class = b),
    prior(exponential(1), class = sigma)
  )
  model.agent.surprisal.lstm <-  brm(mV
                                     ~ 1
                                     + trial_number.Z
                                     + surprisal.lstm.Z
                                     + Condition
                                     + t2(x, y, by = Condition, k = 10)
                                     + t2(x, y, by = surprisal.lstm.Z, k = 10)
                                     + t2(x, y, Participant, bs = "re")
                                     + t2(x, y, Stimulus, bs = "re"),
                                     prior = prior.brms,
                                     sample_prior = TRUE,
                                     data = basque.data,
                                     chains = 4, iter = 2000, cores = 4
  )
  saveRDS(model.agent.surprisal.lstm,
          '../gams_fitted_models/gam_eu_agent_lstm.RDS')
}
model.eu.agent.surp.lstm <-
  readRDS('../gams_fitted_models/gam_eu_agent_lstm.RDS')
summary(model.eu.agent.surp.lstm)
```

```
##  Family: gaussian
##   Links: mu = identity; sigma = identity
## Formula: mV ~ 1 + trial_number.Z + surprisal.lstm.Z + condition + t2(x, y, by = condition, k = 10) +
##    Data: basque.data (Number of observations: 68600)
## Samples: 4 chains, each with iter = 2000; warmup = 1000; thin = 1;
##          total post-warmup samples = 4000
##
## Smooth Terms:
##                                 Estimate Est.Error l-95% CI u-95% CI Rhat Bulk_ESS Tail_ESS
## sds(t2xyconditionAbias_1)           2.04      1.82     0.07     6.77 1.00     1180     2113
## sds(t2xyconditionAbias_2)          12.13      3.68     6.81    20.92 1.00     3145     2951
## sds(t2xyconditionAbias_3)          13.60      4.10     7.78    23.67 1.00     2725     3012
## sds(t2xyconditionnonAbias_1)        3.86      2.48     0.46     9.99 1.00      792     1241
## sds(t2xyconditionnonAbias_2)       10.74      3.29     5.98    18.73 1.00     2664     2617
## sds(t2xyconditionnonAbias_3)       12.51      3.81     7.07    21.87 1.00     2167     2617
## sds(t2xysurprisal.lstm.Z_1)         0.54      0.50     0.02     1.85 1.00     2353     2307
## sds(t2xysurprisal.lstm.Z_2)         1.29      1.07     0.04     4.00 1.00     1896     1654
## sds(t2xysurprisal.lstm.Z_3)         0.94      0.88     0.03     3.29 1.00     2077     2333
## sds(t2xyParticipant_1)              0.48      0.12     0.26     0.73 1.00     1755     2502
## sds(t2xyStimulus_1)                 0.08      0.06     0.00     0.23 1.00     2811     2091
##
## Population-Level Effects:
##                         Estimate Est.Error l-95% CI u-95% CI Rhat Bulk_ESS Tail_ESS
## Intercept                  -2.36      0.11    -2.56    -2.13 1.00     4202     3111
## trial_number.Z              0.59      0.04     0.52     0.67 1.00     9432     2768
## surprisal.lstm.Z            0.05      0.72    -1.35     1.45 1.00     5106     2747
## condition2                  0.63      0.15     0.33     0.92 1.00     2711     3048
```

```
## t2xy:conditionAbias_1        1.33      0.15     1.03     1.61 1.00     3168     2845
## t2xy:conditionAbias_2       -0.05      0.13    -0.31     0.20 1.00     3840     2963
## t2xy:conditionAbias_3       -0.08      0.20    -0.47     0.33 1.00     3958     2999
## t2xy:conditionnonAbias_1     1.31      0.13     1.05     1.59 1.00     2644     2803
## t2xy:conditionnonAbias_2     0.12      0.11    -0.09     0.36 1.00     2986     3269
## t2xy:conditionnonAbias_3     0.01      0.17    -0.32     0.34 1.00     3186     2860
## t2xy:surprisal.lstm.Z_1      0.09      0.70    -1.27     1.45 1.00     5097     2789
## t2xy:surprisal.lstm.Z_2     -0.14      0.05    -0.23    -0.05 1.00     7804     3239
## t2xy:surprisal.lstm.Z_3     -0.04      0.04    -0.13     0.05 1.00     8046     3055
## t2xy:surprisal.lstm.Z_4     -0.01      0.06    -0.12     0.10 1.00     7906     3082
##
## Family Specific Parameters:
##       Estimate Est.Error l-95% CI u-95% CI Rhat Bulk_ESS Tail_ESS
## sigma    10.07      0.03    10.01    10.12 1.00     5700     2257
##
## Samples were drawn using sampling(NUTS). For each parameter, Bulk_ESS
## and Tail_ESS are effective sample size measures, and Rhat is the potential
## scale reduction factor on split chains (at convergence, Rhat = 1).
```

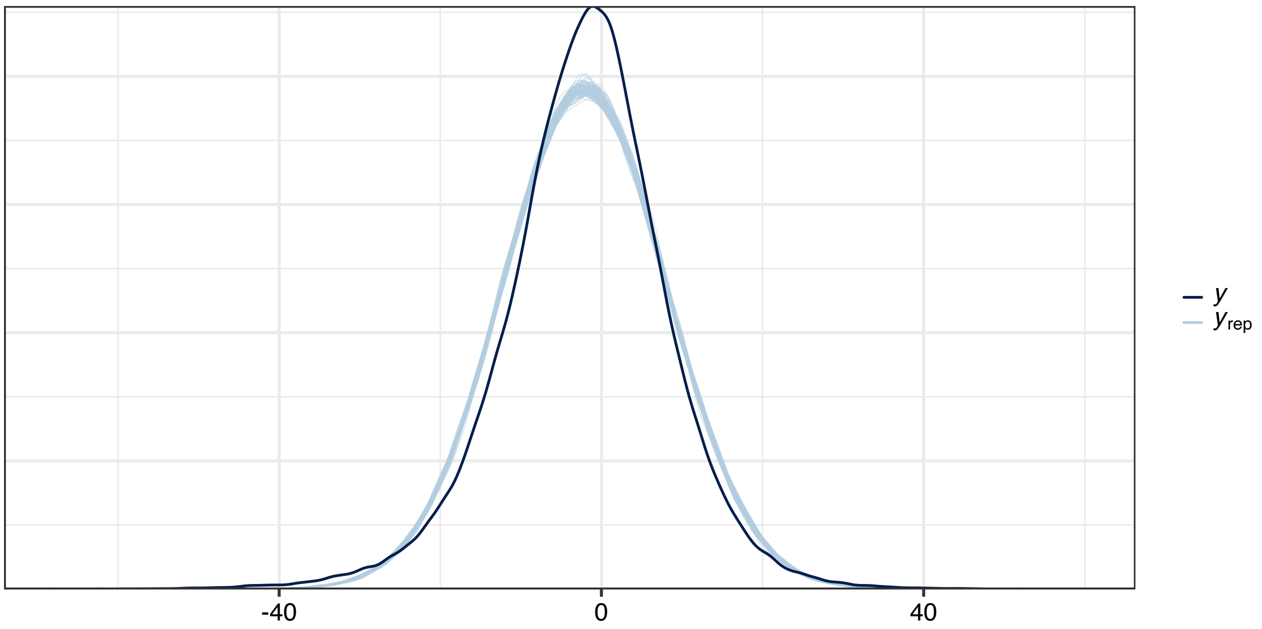

RoBERTa-based surprisal:

```
if (!file.exists('../gams_fitted_models/gam_eu_agent_roberta.RDS')){
  prior.brms <-  c(
    prior(normal(0,1), class = b),
    prior(exponential(1), class = sigma)
  )
  model.agent.surprisal.tr <-  brm(mV
                                   ~ 1
                                   + trial_number.Z
                                   + surprisal.tr.Z
                                   + Condition
                                   + t2(x, y, by = Condition, k = 10)
                                   + t2(x, y, by = surprisal.tr.Z, k = 10)
                                   + t2(x, y, Participant, bs = "re")
```

```
                                  + t2(x, y, Stimulus, bs = "re"),
                                  prior = prior.brms,
                                  sample_prior = TRUE,
                                  data = basque.data,
                                  chains = 4, iter = 2000, cores = 4
  )
  saveRDS(model.agent.surprisal.tr,
          '../gams_fitted_models/gam_eu_agent_roberta.RDS')
}
model.eu.agent.surp.roberta <-
  readRDS('../gams_fitted_models/gam_eu_agent_roberta.RDS')
summary(model.eu.agent.surp.roberta)
```

```
##  Family: gaussian
##   Links: mu = identity; sigma = identity
## Formula: mV ~ 1 + trial_number.Z + surprisal.tr.Z + condition + t2(x, y, by = condition, k = 10) + t2
##    Data: basque.data (Number of observations: 68600)
## Samples: 4 chains, each with iter = 2000; warmup = 1000; thin = 1;
##          total post-warmup samples = 4000
##
## Smooth Terms:
##                                Estimate Est.Error l-95% CI u-95% CI Rhat Bulk_ESS Tail_ESS
## sds(t2xyconditionAbias_1)          2.04      1.85     0.08     6.83 1.00     1367     2188
## sds(t2xyconditionAbias_2)         12.29      3.72     6.83    20.99 1.00     2832     2968
## sds(t2xyconditionAbias_3)         13.49      4.03     7.77    23.43 1.00     2768     3218
## sds(t2xyconditionnonAbias_1)       3.68      2.44     0.43     9.52 1.00      790     1460
## sds(t2xyconditionnonAbias_2)      10.84      3.37     5.88    18.85 1.00     2480     2449
## sds(t2xyconditionnonAbias_3)      12.49      3.89     6.93    21.88 1.00     2128     2615
## sds(t2xysurprisal.tr.Z_1)          0.54      0.49     0.02     1.81 1.00     2168     2201
## sds(t2xysurprisal.tr.Z_2)          1.10      1.03     0.03     3.90 1.00     2136     1934
## sds(t2xysurprisal.tr.Z_3)          1.00      0.92     0.04     3.39 1.00     2780     2771
## sds(t2xyParticipant_1)             0.48      0.12     0.25     0.73 1.00     1406     1943
## sds(t2xyStimulus_1)                0.09      0.06     0.00     0.23 1.00     3156     2509
##
## Population-Level Effects:
##                            Estimate Est.Error l-95% CI u-95% CI Rhat Bulk_ESS Tail_ESS
## Intercept                     -2.34      0.11    -2.56    -2.11 1.00     3901     3387
## trial_number.Z                 0.60      0.04     0.52     0.67 1.00    11285     2447
## surprisal.tr.Z                -0.14      0.73    -1.57     1.29 1.00     5293     2992
## condition2                     0.61      0.15     0.31     0.91 1.00     2572     2865
## t2xy:conditionAbias_1          1.31      0.14     1.02     1.59 1.00     2711     2026
## t2xy:conditionAbias_2         -0.06      0.13    -0.31     0.19 1.00     3471     2795
## t2xy:conditionAbias_3         -0.08      0.20    -0.46     0.31 1.00     4097     3196
## t2xy:conditionnonAbias_1       1.31      0.13     1.05     1.58 1.00     2734     2788
## t2xy:conditionnonAbias_2       0.12      0.11    -0.09     0.35 1.00     2763     2949
## t2xy:conditionnonAbias_3       0.01      0.17    -0.33     0.35 1.00     3322     2891
## t2xy:surprisal.tr.Z_1         -0.17      0.70    -1.56     1.22 1.00     5245     3216
## t2xy:surprisal.tr.Z_2         -0.08      0.05    -0.17     0.01 1.00     8732     2913
## t2xy:surprisal.tr.Z_3         -0.01      0.04    -0.10     0.08 1.00     7982     3276
## t2xy:surprisal.tr.Z_4         -0.04      0.06    -0.15     0.07 1.00     7961     3168
##
## Family Specific Parameters:
##       Estimate Est.Error l-95% CI u-95% CI Rhat Bulk_ESS Tail_ESS
```

```
## sigma     10.06      0.03     10.01     10.12 1.00     5096     2281
##
## Samples were drawn using sampling(NUTS). For each parameter, Bulk_ESS
## and Tail_ESS are effective sample size measures, and Rhat is the potential
## scale reduction factor on split chains (at convergence, Rhat = 1).
```

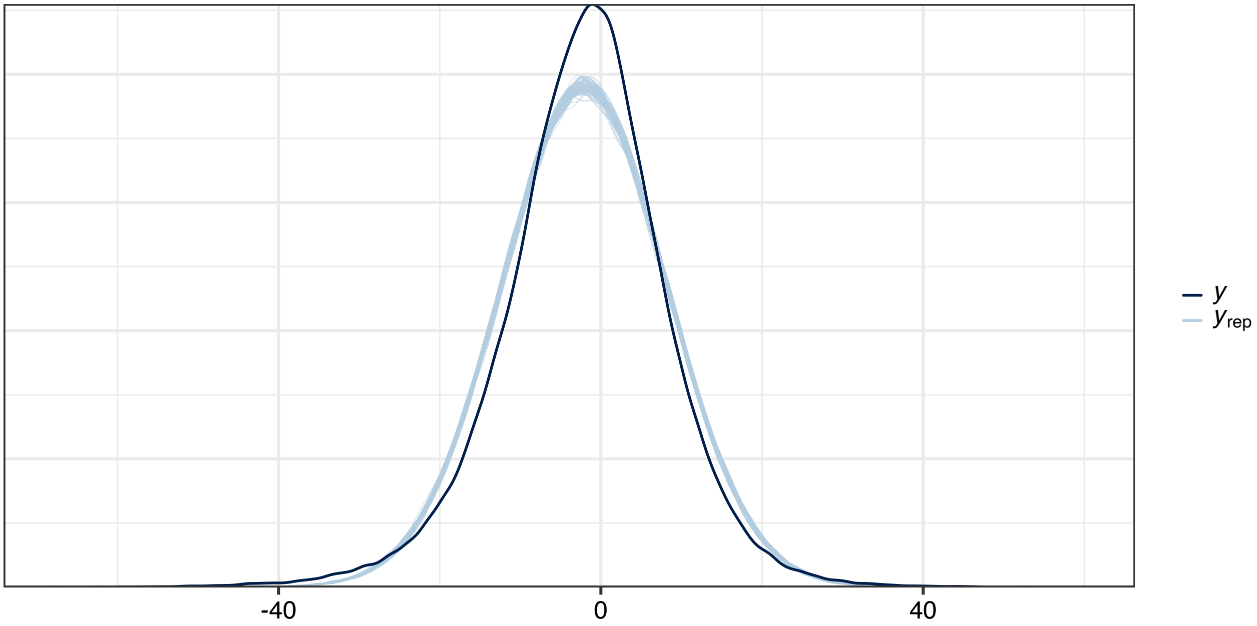

GPT-based surprisal:

```
if (!file.exists('../gams_fitted_models/gam_eu_agent_gpt.RDS')){
  prior.brms <-  c(
    prior(normal(0,1), class = b),
    prior(exponential(1), class = sigma)
  )
  model.agent.surprisal.gpt <-  brm(mV
                                  ~ 1
                                  + trial_number.Z
                                  + surprisal.gpt.Z
                                  + Condition
                                  + t2(x, y, by = Condition, k = 10)
                                  + t2(x, y, by = surprisal.gpt.Z, k = 10)
                                  + t2(x, y, Participant, bs = "re")
                                  + t2(x, y, Stimulus, bs = "re"),
                                  prior = prior.brms,
                                  sample_prior = TRUE,
                                  data = basque.data,
                                  chains = 4, iter = 2000, cores = 4
  )
  saveRDS(model.agent.surprisal.gpt,
          '../gams_fitted_models/gam_eu_agent_gpt.RDS')
}
model.eu.agent.surp.gpt <-
  readRDS('../gams_fitted_models/gam_eu_agent_gpt.RDS')
summary(model.eu.agent.surp.gpt)
```

```
##  Family: gaussian
##   Links: mu = identity; sigma = identity
## Formula: mV ~ 1 + trial_number.Z + surprisal.gpt.Z + condition + t2(x, y, by = condition, k = 10) + [illegible]
##    Data: basque.data (Number of observations: 68600)
## Samples: 4 chains, each with iter = 2000; warmup = 1000; thin = 1;
##          total post-warmup samples = 4000
##
## Smooth Terms:
##                               Estimate Est.Error l-95% CI u-95% CI Rhat Bulk_ESS Tail_ESS
## sds(t2xyconditionAbias_1)        2.04      1.79     0.06     6.73 1.00     1628     2056
## sds(t2xyconditionAbias_2)       12.24      3.70     6.84    21.06 1.00     2889     3254
## sds(t2xyconditionAbias_3)       13.44      3.99     7.70    23.62 1.00     2741     2718
## sds(t2xyconditionnonAbias_1)     3.73      2.46     0.43     9.90 1.01      819     1375
## sds(t2xyconditionnonAbias_2)    10.90      3.31     6.08    18.91 1.00     2737     3344
## sds(t2xyconditionnonAbias_3)    12.70      3.85     7.13    22.15 1.00     2529     2903
## sds(t2xysurprisal.gpt.Z_1)       0.50      0.46     0.02     1.71 1.00     2158     2460
## sds(t2xysurprisal.gpt.Z_2)       1.09      1.03     0.04     3.83 1.00     2200     2464
## sds(t2xysurprisal.gpt.Z_3)       1.70      1.29     0.10     5.11 1.00     1613     1852
## sds(t2xyParticipant_1)           0.48      0.12     0.26     0.73 1.00     1683     1718
## sds(t2xyStimulus_1)              0.09      0.07     0.00     0.24 1.00     3188     2156
##
## Population-Level Effects:
##                         Estimate Est.Error l-95% CI u-95% CI Rhat Bulk_ESS Tail_ESS
## Intercept                  -2.33      0.11    -2.54    -2.10 1.00     4324     3392
## trial_number.Z              0.60      0.04     0.52     0.67 1.00    11071     2875
## surprisal.gpt.Z            -0.03      0.73    -1.49     1.39 1.00     5704     2995
## condition2                  0.59      0.15     0.30     0.88 1.00     3182     3340
## t2xy:conditionAbias_1       1.34      0.14     1.05     1.61 1.00     3173     2607
## t2xy:conditionAbias_2      -0.06      0.13    -0.31     0.19 1.00     3317     3087
## t2xy:conditionAbias_3      -0.07      0.21    -0.47     0.36 1.00     3659     2855
## t2xy:conditionnonAbias_1    1.30      0.13     1.04     1.57 1.00     2798     2741
## t2xy:conditionnonAbias_2    0.12      0.11    -0.09     0.35 1.00     2754     2623
## t2xy:conditionnonAbias_3    0.01      0.17    -0.30     0.35 1.00     3291     3055
## t2xy:surprisal.gpt.Z_1     -0.01      0.70    -1.39     1.39 1.00     5736     3032
## t2xy:surprisal.gpt.Z_2     -0.11      0.05    -0.20    -0.01 1.00     7969     3025
## t2xy:surprisal.gpt.Z_3      0.01      0.04    -0.07     0.10 1.00     8066     3286
## t2xy:surprisal.gpt.Z_4      0.00      0.06    -0.12     0.12 1.00     7700     3239
##
## Family Specific Parameters:
##       Estimate Est.Error l-95% CI u-95% CI Rhat Bulk_ESS Tail_ESS
## sigma    10.07      0.03    10.02    10.12 1.01     5685     2200
##
## Samples were drawn using sampling(NUTS). For each parameter, Bulk_ESS
## and Tail_ESS are effective sample size measures, and Rhat is the potential
## scale reduction factor on split chains (at convergence, Rhat = 1).
```

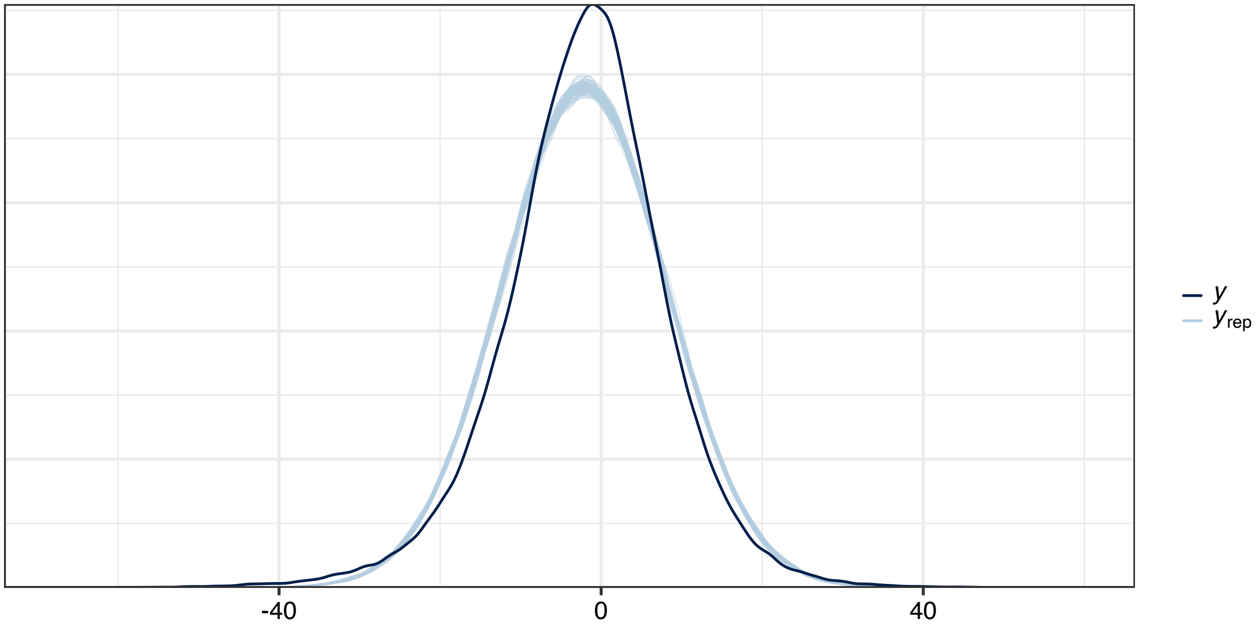

S5.1.4 Null model

```
if (!file.exists('../gams_fitted_models/gam_eu_null.RDS')){
  prior.brms <-  c(
    prior(normal(0,1), class = b),
    prior(exponential(1), class = sigma)
  )
  model.null <-  brm(mV
                     ~ 1
                     + trial_number.Z
                     + t2(x, y, Participant, bs = "re")
                     + t2(x, y, Stimulus, bs = "re"),
                     prior = prior.brms,
                     sample_prior = TRUE,
                     data = basque.data,
                     chains = 4, iter = 2000, cores = 4
  )
  saveRDS(model.null, '../gams_fitted_models/gam_eu_null.RDS')
}
model.eu.null <- readRDS('../gams_fitted_models/gam_eu_null.RDS')
summary(model.eu.null)
```

```
##  Family: gaussian
##   Links: mu = identity; sigma = identity
## Formula: mV ~ 1 + trial_number.Z + t2(x, y, Participant, bs = "re") + t2(x, y, Stimulus, bs = "re")
##    Data: basque.data (Number of observations: 68600)
## Samples: 4 chains, each with iter = 2000; warmup = 1000; thin = 1;
##          total post-warmup samples = 4000
##
## Smooth Terms:
##                         Estimate Est.Error l-95% CI u-95% CI Rhat Bulk_ESS Tail_ESS
```

```
## sds(t2xyParticipant_1)     0.46      0.12     0.24     0.71 1.00     1480     1890
## sds(t2xyStimulus_1)        0.08      0.06     0.00     0.23 1.00     2716     2240
##
## Population-Level Effects:
##                Estimate Est.Error l-95% CI u-95% CI Rhat Bulk_ESS Tail_ESS
## Intercept         -2.07      0.04    -2.15    -1.99 1.00     7845     2418
## trial_number.Z     0.59      0.04     0.52     0.67 1.00     6886     2789
##
## Family Specific Parameters:
##       Estimate Est.Error l-95% CI u-95% CI Rhat Bulk_ESS Tail_ESS
## sigma    10.17      0.03    10.12    10.22 1.00     5701     2591
##
## Samples were drawn using sampling(NUTS). For each parameter, Bulk_ESS
## and Tail_ESS are effective sample size measures, and Rhat is the potential
## scale reduction factor on split chains (at convergence, Rhat = 1).
```

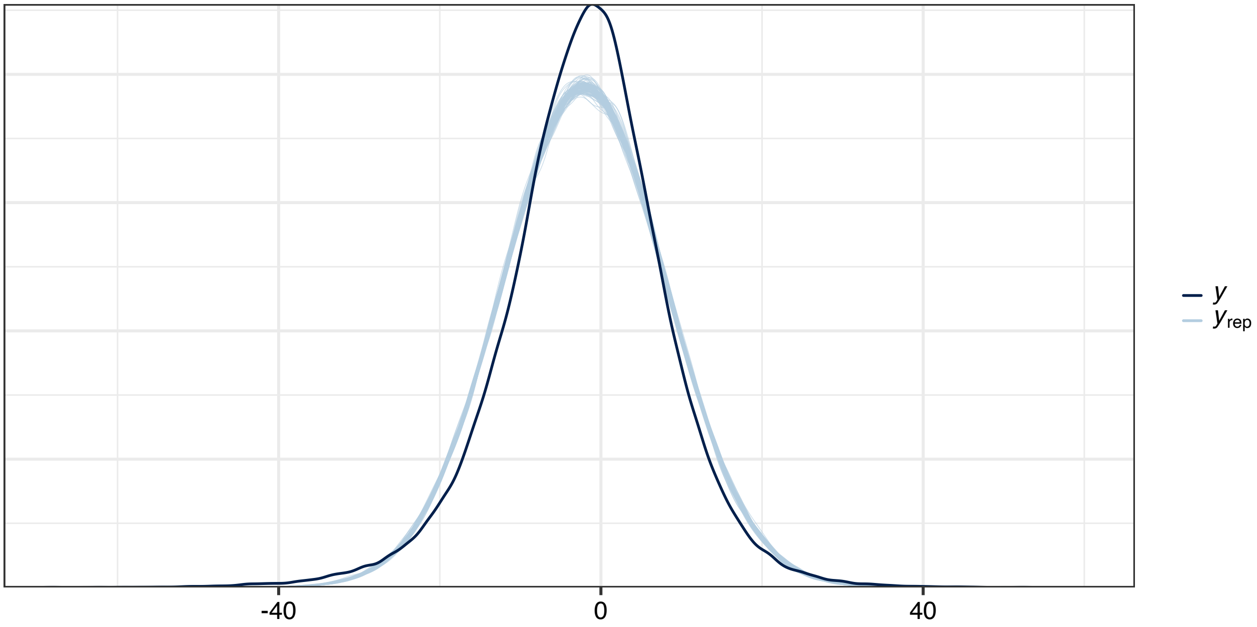

S5.2 Model comparison with stacking

Here we perform model comparison.

```
if (!file.exists('../temp_data/weights_eu_stacking.RDS')){
  loo.eu.agent <- loo(model.eu.agent)
  loo.eu.lstm <- loo(model.eu.surp.lstm)
  loo.eu.rob <- loo(model.eu.surp.roberta)
  loo.eu.gpt <- loo(model.eu.surp.gpt)
  loo.eu.agent.lstm <- loo(model.eu.agent.surp.lstm)
  loo.eu.agent.rob <- loo(model.eu.agent.surp.roberta)
  loo.eu.agent.gpt <- loo(model.eu.agent.surp.gpt)
  loo.eu.null <- loo(model.eu.null)
  lweights <- loo_model_weights(list(
    "agent bias only"= loo.eu.agent,
    "surprisal lstm" =  loo.eu.lstm,
```

```
    "surprisal roberta" = loo.eu.rob,
    "surprisal gpt" = loo.eu.gpt,
    "surprisal lstm and agent bias" = loo.eu.agent.lstm,
    "surprisal roberta and agent bias" = loo.eu.agent.rob,
    "surprisal gpt and agent bias" = loo.eu.agent.gpt,
    "null" = loo.eu.null), method='stacking')

  dfweights <- as.data.frame(as.matrix(lweights))
  saveRDS(dfweights,'../temp_data/weights_eu_stacking.RDS')
}
```

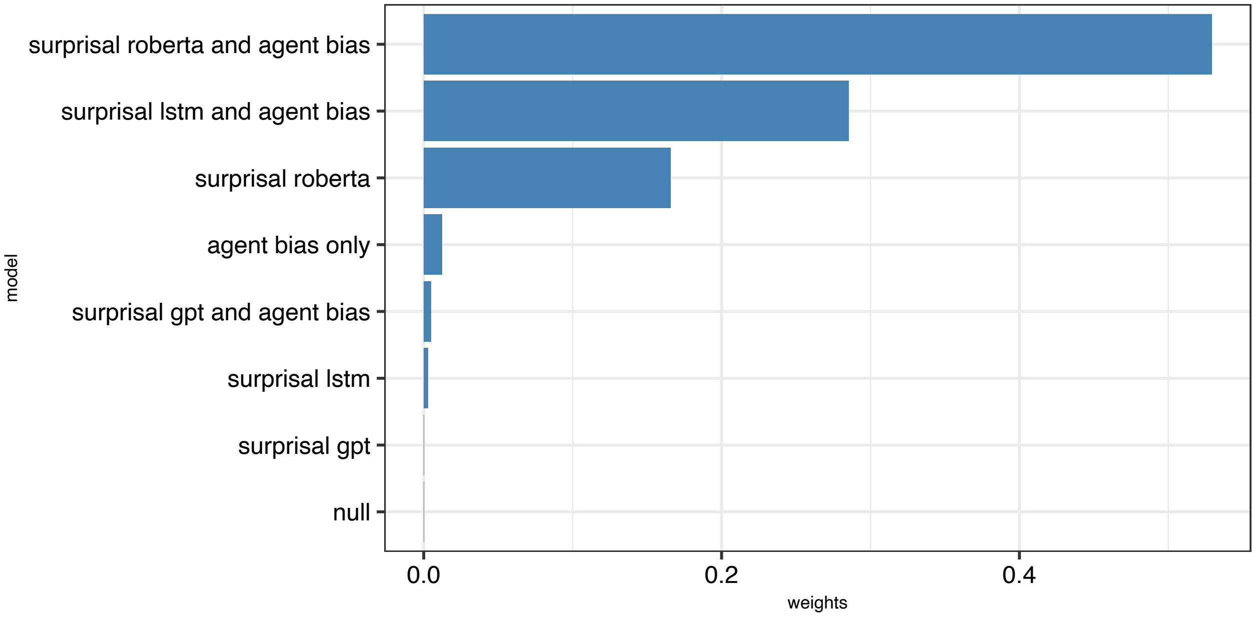

Figure S1: Basque: Weights of models (method = stacking)

S5.3 Model predictions

In what follows we compute and plot the mean posterior predicted effects across the smoothed scalp surfaces. In each plot, the lef-hand side shows the difference between conditions. For surprisal we plot the difference between pedictions at +2 and at -2 standard deviations from the mean. Panels on the right-hand side quantify the evidence of the effect in terms of the proportion of the posterior distributions that is below or above 0.

S5.3.1 Agent preference alone

```
n_samps <- 4000
newdata.eu.agent <- expand_grid(
  x = seq(min(basque.data$x), max(basque.data$x), length.out = 30),
  y = seq(min(basque.data$y), max(basque.data$y),length.out = 30),
  Participant = "01",
  Stimulus_Cond = c("1_1:Abias", "1_2:nonAbias"),
  trial_number.Z = 0,
```

```
)

newdata.eu.agent <- newdata.eu.agent %>%
  separate(Stimulus_Cond, c('Stimulus', 'condition'), sep=':')

preds.eu.agent <- posterior_epred(model.eu.agent, newdata=newdata.eu.agent,
                                  re.form=NA)

preds.eu.agent_long <- as.data.frame(preds.eu.agent) %>%
  mutate(samp = 1:nrow(preds.eu.agent)) %>%
  pivot_longer(-samp)

preds.eu.agent_long$x <- rep(newdata.eu.agent$x, n_samps)
preds.eu.agent_long$y <- rep(newdata.eu.agent$y, n_samps)
preds.eu.agent_long$condition <- rep(newdata.eu.agent$condition, n_samps)

preds.eu.agent_summary <- preds.eu.agent_long %>%
  group_by(x,y,samp) %>%
  mutate(diff_cond = value[condition == 'Abias'] -
           value[condition == 'nonAbias']) %>%
  ungroup() %>%
  group_by(x,y) %>%
  summarise(PP = ifelse(mean(diff_cond) > 0, mean(diff_cond > 0),
                        mean(diff_cond < 0)),
            mean_diff = mean(diff_cond))

plot.agent <- preds.eu.agent_summary %>%
  mutate(sig = ifelse(PP>0.80, 1, 0)) %>%
  ggplot(aes(x=x, y=y, fill=mean_diff, alpha = sig)) +
  geom_tile()+
  scale_fill_gradient2(low = '#0053d3', high = '#b22709', midpoint=0,
                       aesthetics='fill')+
  guides(alpha = "none") +
  theme(panel.grid = element_blank(), panel.border = element_blank(),
        strip.background = element_blank(),strip.text.x = element_text(size=5),
        axis.ticks.x = element_blank(),
        axis.ticks.y=element_blank(),
        axis.text.x=element_blank(),
        axis.text.y = element_blank(),
        plot.background = element_rect(fill = "transparent", colour = NA))   +
  labs(fill=(expression(paste(Delta, paste(mu, "V")))),
       x=element_blank(),
       y=element_blank())

plot.agent.pp <- preds.eu.agent_summary %>%
  ggplot(aes(x=x, y=y, fill=PP)) +
  geom_tile()+
  scale_fill_gradientn(colours = c('white','grey', 'black'),
                       limits = c(0.8, 1), na.value='white' ) +
  labs(fill=(expression(paste('P(|',Delta,mu, V, '| > 0)', ))),
       x=element_blank(),
       y=element_blank()) +
  theme(panel.grid = element_blank(),
```

```
        panel.border = element_blank(),
        strip.background = element_blank(),
        axis.ticks.x = element_blank(),
        axis.ticks.y=element_blank(),
        axis.text.x=element_blank(),
        axis.text.y = element_blank(),
        plot.background = element_rect(fill = "transparent", colour = NA))
```

```
wrap_plots(list(plot.agent, plot.agent.pp))
```

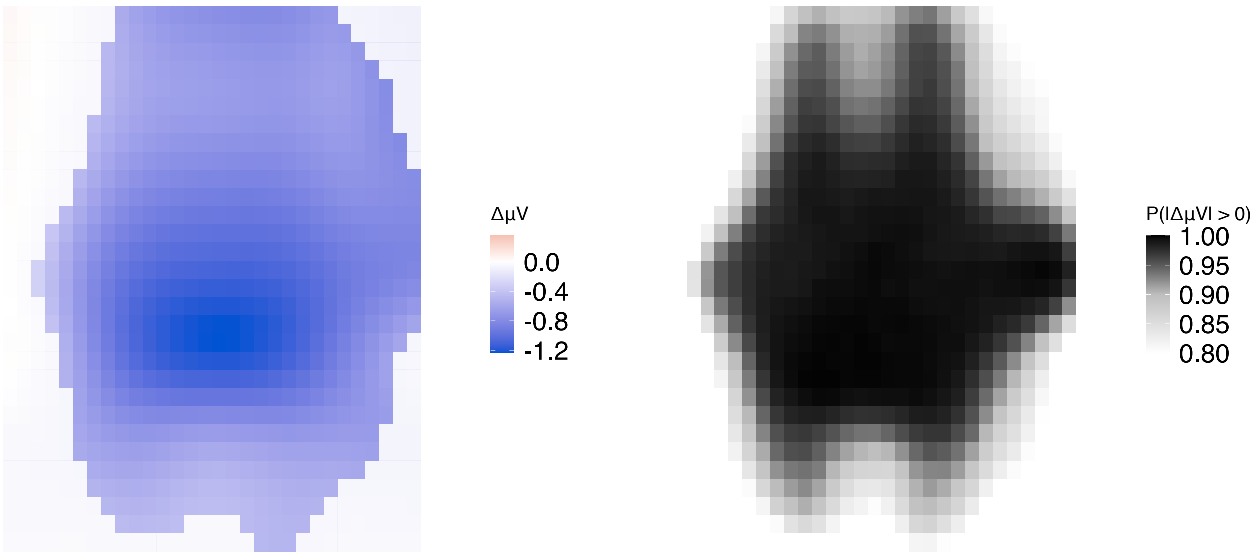

Figure S2: Left: Pair-wise fitted differences of micro-volt for sentences with vs. sentences without the predicted reanalysis from model with agent preference predictor alone. Right: Percentage of posterior probability mass of micro-volt mean difference below or above 0.

S5.3.2 LSTM-based surprisal alone

```
n_samps <- 4000
newdata.eu.surplstm <- expand_grid(
  x = seq(min(basque.data$x), max(basque.data$x), length.out = 30),
  y = seq(min(basque.data$y), max(basque.data$y),length.out = 30),
  surprisal.lstm.Z = seq(from=-2,to=2, length.out=17),
  Participant = "01",
  Stimulus = "1_1",
  trial_number.Z = 0,
)

preds.eu.surplstm <- posterior_epred(model.eu.surp.lstm,
                                     newdata=newdata.eu.surplstm, re.form=NA)
preds.eu.surplstm_long <- as.data.frame(preds.eu.surplstm) %>%
  mutate(samp = 1:nrow(preds.eu.surplstm)) %>%
  pivot_longer(-samp)
```

```r
preds.eu.surplstm_long$x <- rep(newdata.eu.surplstm$x, n_samps)
preds.eu.surplstm_long$y <- rep(newdata.eu.surplstm$y, n_samps)
preds.eu.surplstm_long$surprisal <-
  rep(newdata.eu.surplstm$surprisal.lstm.Z, n_samps)

summary_eu_surplstm <- preds.eu.surplstm_long %>%
  group_by(x,y,samp) %>%
  mutate(diff_surprisal_low_mean = value[surprisal == 2] -
           value[surprisal == -2]) %>%
  ungroup() %>%
  group_by(x,y) %>%
  summarise(PP = ifelse(mean(diff_surprisal_low_mean) > 0,
                        mean(diff_surprisal_low_mean > 0),
                        mean(diff_surprisal_low_mean < 0)),
            mean_diff = mean(diff_surprisal_low_mean))

plot.lstm <- summary_eu_surplstm %>%
  mutate(sig = ifelse(PP>0.80, 1, 0)) %>%
  ggplot(aes(x=x, y=y, fill=mean_diff, alpha = sig)) +
  geom_tile()+
  scale_fill_gradient2(low = '#0053d3', high = '#b22709', midpoint=0,
                       aesthetics='fill')+
  guides(alpha = "none") +
  theme(panel.grid = element_blank(), panel.border = element_blank(),
        strip.background = element_blank(),strip.text.x = element_text(size=5),
        axis.ticks.x = element_blank(),
        axis.ticks.y=element_blank(),
        axis.text.x=element_blank(),
        axis.text.y = element_blank(),
        plot.background = element_rect(fill = "transparent", colour = NA))   +
  labs(fill=(expression(paste(Delta, paste(mu, "V")))),
       x=element_blank(),
       y=element_blank())

plot.lstm.pp <- summary_eu_surplstm %>%
  ggplot(aes(x=x, y=y, fill=PP)) +
  geom_tile()+
  scale_fill_gradientn(colours = c('white','grey', 'black'),
                       limits = c(0.8, 1), na.value='white' ) +
  labs(fill=(expression(paste('P(|',Delta,mu, V, '| > 0)', ))),
       x=element_blank(),
       y=element_blank()) +
  theme(panel.grid = element_blank(),
        panel.border = element_blank(),
        strip.background = element_blank(),
        axis.ticks.x = element_blank(),
        axis.ticks.y=element_blank(),
        axis.text.x=element_blank(),
        axis.text.y = element_blank(),
        plot.background = element_rect(fill = "transparent", colour = NA))
```

```r
wrap_plots(list(plot.lstm, plot.lstm.pp))
```

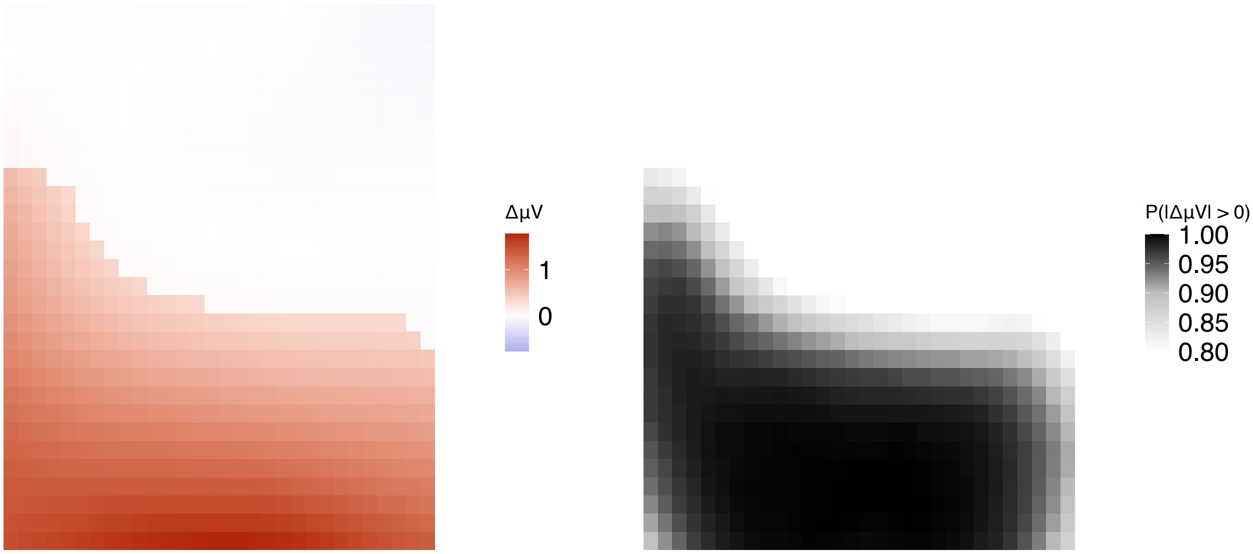

Figure S3: Left: Pair-wise fitted differences of μV for sentences with high vs. sentences with low (+2 vs. -2 std. dev. from the mean) from model with LSTM surprisal as predictor alone. Right: Percentage of posterior probability mass of μV mean difference below or above 0.

S5.3.3 RoBERTa-based surprisal alone

```
n_samps <- 4000
newdata.eu.surptr <- rename(newdata.eu.surplstm, surprisal.tr.Z =
                              surprisal.lstm.Z)
preds.eu.surptr <- posterior_epred(model.eu.surp.roberta,
                                   newdata=newdata.eu.surptr,
                                   re.form=NA)
preds.eu.surptr_long <- as.data.frame(preds.eu.surptr) %>%
  mutate(samp = 1:nrow(preds.eu.surptr)) %>%
  pivot_longer(-samp)

preds.eu.surptr_long$x <- rep(newdata.eu.surptr$x, n_samps)
preds.eu.surptr_long$y <- rep(newdata.eu.surptr$y, n_samps)
preds.eu.surptr_long$surprisal <- rep(newdata.eu.surptr$surprisal.tr.Z, n_samps)

summary_eu_surptr <- preds.eu.surptr_long %>%
  group_by(x,y,samp) %>%
  mutate(diff_surprisal_low_mean = value[surprisal == 2] -
           value[surprisal == -2]) %>%
  ungroup() %>%
  group_by(x,y) %>%
  summarise(PP = ifelse(mean(diff_surprisal_low_mean) > 0,
                        mean(diff_surprisal_low_mean > 0),
                        mean(diff_surprisal_low_mean < 0)),
            mean_diff = mean(diff_surprisal_low_mean))

plot.tr <- summary_eu_surptr %>%
  mutate(sig = ifelse(PP>0.80, 1, 0)) %>%
  ggplot(aes(x=x, y=y, fill=mean_diff, alpha = sig)) +
```

```
  geom_tile()+
  scale_fill_gradient2(low = '#0053d3', high = '#b22709', midpoint=0,
                       aesthetics='fill')+
  guides(alpha = "none") +
  theme(panel.grid = element_blank(), panel.border = element_blank(),
        strip.background = element_blank(),strip.text.x = element_text(size=5),
        axis.ticks.x = element_blank(),
        axis.ticks.y=element_blank(),
        axis.text.x=element_blank(),
        axis.text.y = element_blank(),
        plot.background = element_rect(fill = "transparent", colour = NA))   +
  labs(fill=(expression(paste(Delta, paste(mu, "V")))),
       x=element_blank(),
       y=element_blank())

plot.tr.pp <- summary_eu_surptr %>%
  ggplot(aes(x=x, y=y, fill=PP)) +
  geom_tile()+
  scale_fill_gradientn(colours = c('white','grey', 'black'),
                       limits = c(0.8, 1), na.value='white' ) +
  labs(fill=(expression(paste('P(|',Delta,mu, V, '| > 0)', ))),
       x=element_blank(),
       y=element_blank()) +
  theme(panel.grid = element_blank(),
        panel.border = element_blank(),
        strip.background = element_blank(),
        axis.ticks.x = element_blank(),
        axis.ticks.y=element_blank(),
        axis.text.x=element_blank(),
        axis.text.y = element_blank(),
        plot.background = element_rect(fill = "transparent", colour = NA))
```

```
wrap_plots(list(plot.tr, plot.tr.pp))
```

S5.3.4 GPT-based surprisal alone

```
n_samps <- 4000
newdata.eu.surpgpt <- rename(newdata.eu.surplstm, surprisal.gpt.Z =
                               surprisal.lstm.Z)
preds.eu.surpgpt <- posterior_epred(model.eu.surp.gpt,
                                    newdata=newdata.eu.surpgpt,
                                    re.form=NA)
preds.eu.surpgpt_long <- as.data.frame(preds.eu.surpgpt) %>%
  mutate(samp = 1:nrow(preds.eu.surpgpt)) %>%
  pivot_longer(-samp)

preds.eu.surpgpt_long$x <- rep(newdata.eu.surpgpt$x, n_samps)
preds.eu.surpgpt_long$y <- rep(newdata.eu.surpgpt$y, n_samps)
preds.eu.surpgpt_long$surprisal <- rep(newdata.eu.surpgpt$surprisal.gpt.Z, n_samps)

summary_eu_surpgpt <- preds.eu.surpgpt_long %>%
```

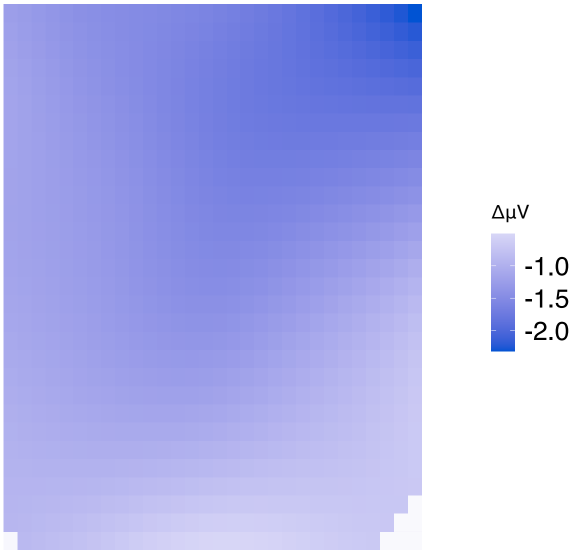

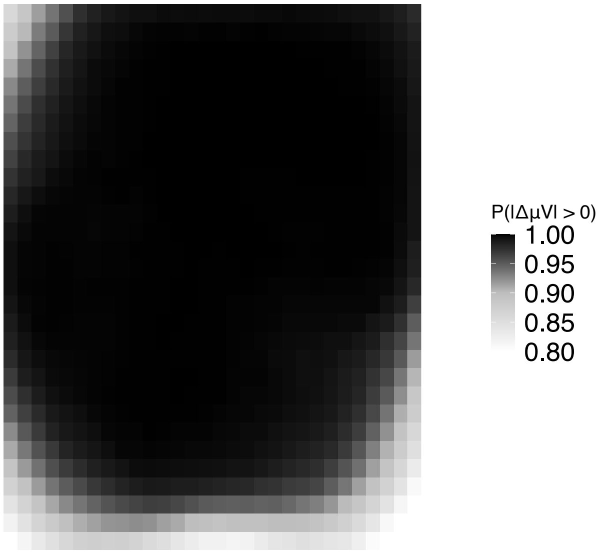

Figure S4: Left: Pair-wise fitted differences of μV for sentences with high vs. sentences with low (+2 vs. -2 std. dev. from the mean) from model with RoBERTa surprisal as predictor alone. Right: Percentage of posterior probability mass of μV mean difference below or above 0.

```
  group_by(x,y,samp) %>%
  mutate(diff_surprisal_low_mean = value[surprisal == 2] -
           value[surprisal == -2]) %>%
  ungroup() %>%
  group_by(x,y) %>%
  summarise(PP = ifelse(mean(diff_surprisal_low_mean) > 0,
                        mean(diff_surprisal_low_mean > 0),
                        mean(diff_surprisal_low_mean < 0)),
            mean_diff = mean(diff_surprisal_low_mean))

plot.gpt <- summary_eu_surpgpt %>%
  mutate(sig = ifelse(PP>0.80, 1, 0)) %>%
  ggplot(aes(x=x, y=y, fill=mean_diff, alpha = sig)) +
  geom_tile()+
  scale_fill_gradient2(low = '#0053d3', high = '#b22709', midpoint=0,
                       aesthetics='fill')+
  guides(alpha = "none") +
  theme(panel.grid = element_blank(), panel.border = element_blank(),
        strip.background = element_blank(),
        strip.text.x = element_text(size=5),
        axis.ticks.x = element_blank(),
        axis.ticks.y=element_blank(),
        axis.text.x=element_blank(),
        axis.text.y = element_blank(),
        plot.background = element_rect(fill = "transparent", colour = NA))   +
  labs(fill=(expression(paste(Delta, paste(mu, "V")))),
       x=element_blank(),
       y=element_blank())

plot.gpt.pp <- summary_eu_surpgpt %>%
  ggplot(aes(x=x, y=y, fill=PP)) +
```

```
  geom_tile()+
  scale_fill_gradientn(colours = c('white','grey', 'black'),
                       limits = c(0.8, 1), na.value='white' ) +
  labs(fill=(expression(paste('P(|',Delta,mu, V, '| > 0)', ))),
       x=element_blank(),
       y=element_blank()) +
  theme(panel.grid = element_blank(),
        panel.border = element_blank(),
        strip.background = element_blank(),
        axis.ticks.x = element_blank(),
        axis.ticks.y=element_blank(),
        axis.text.x=element_blank(),
        axis.text.y = element_blank(),
        plot.background = element_rect(fill = "transparent", colour = NA))
```

```
wrap_plots(list(plot.gpt, plot.gpt.pp))
```

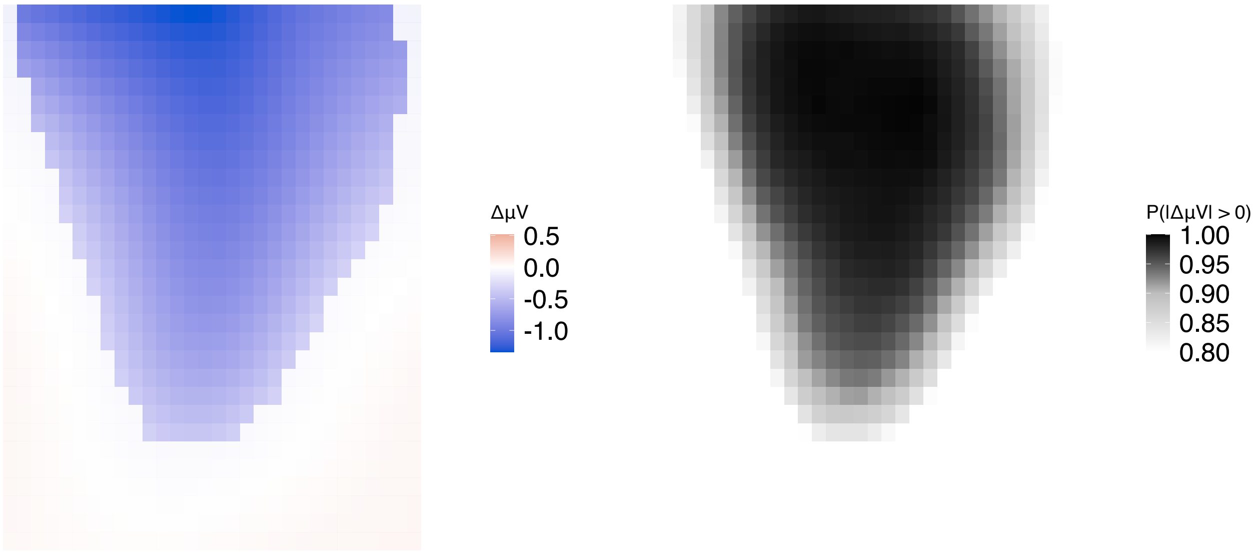

Figure S5: Left: Pair-wise fitted differences of μV for sentences with high vs. sentences with low (+2 vs. -2 std. dev. from the mean) from model with GPT-2 surprisal as predictor alone. Right: Percentage of posterior probability mass of μV mean difference below or above 0.

S5.3.5 LSTM-based surprisal and Agent Preference

```
n_samps <- 4000
newdata.eu.agent.surplstm <- expand_grid(
  x = seq(min(basque.data$x), max(basque.data$x), length.out = 30),
  y = seq(min(basque.data$y), max(basque.data$y),length.out = 30),
  surprisal.lstm.Z = seq(from=-2,to=2, length.out=17),
  Participant = "01",
  Stimulus_Cond = c("1_1:Abias", "1_2:nonAbias"),
  trial_number.Z = 0,
)
```

```
newdata.eu.agent.surplstm <- separate(newdata.eu.agent.surplstm,
                                      Stimulus_Cond, c('Stimulus', 'condition'),
                                      sep=':')

preds.eu.agent.surplstm <- posterior_epred(model.eu.agent.surp.lstm,
                                           newdata=newdata.eu.agent.surplstm,
                                           re.form=NA)

preds.eu.agent.surplstm_long <- as.data.frame(preds.eu.agent.surplstm) %>%
  mutate(samp = 1:nrow(preds.eu.agent.surplstm)) %>%
  pivot_longer(-samp)

preds.eu.agent.surplstm_long$x <- rep(newdata.eu.agent.surplstm$x, n_samps)
preds.eu.agent.surplstm_long$y <- rep(newdata.eu.agent.surplstm$y, n_samps)
preds.eu.agent.surplstm_long$surprisal <-
  rep(newdata.eu.agent.surplstm$surprisal.lstm.Z, n_samps)
preds.eu.agent.surplstm_long$condition <-
  rep(newdata.eu.agent.surplstm$condition, n_samps)

summary_eu_agent_surplstm.abias <- preds.eu.agent.surplstm_long %>%
  filter(condition %in% 'Abias')%>%
  group_by(x,y,samp) %>%
  summarise(diff_surprisal_low_mean = value[surprisal == 2] -
              value[surprisal == -2]) %>%
  ungroup() %>%
  group_by(x,y) %>%
  summarise(PP = ifelse(mean(diff_surprisal_low_mean) > 0,
                        mean(diff_surprisal_low_mean > 0),
                        mean(diff_surprisal_low_mean < 0)),
            mean_diff = mean(diff_surprisal_low_mean)) %>%
  mutate(condition = 'Abias')

summary_eu_agent_surplstm.nonabias <- preds.eu.agent.surplstm_long %>%
  filter(condition %in% 'nonAbias') %>%
  group_by(x,y,samp) %>%
  mutate(diff_surprisal_low_mean = value[surprisal == 2] -
           value[surprisal == -2]) %>%
  ungroup() %>%
  group_by(x,y) %>%
  summarise(PP = ifelse(mean(diff_surprisal_low_mean) > 0,
                        mean(diff_surprisal_low_mean > 0),
                        mean(diff_surprisal_low_mean < 0)),
            mean_diff = mean(diff_surprisal_low_mean)) %>%
  mutate(condition = 'nonAbias')

summary_eu_agent_surplstm <- rbind(summary_eu_agent_surplstm.abias,
                                   summary_eu_agent_surplstm.nonabias)

plot.lstm.agent.surp <- summary_eu_agent_surplstm %>%
  mutate(sig = ifelse(PP>0.80, 1, 0)) %>%
  ggplot(aes(x=x, y=y, fill=mean_diff, alpha = sig)) +
  geom_tile()+
  scale_fill_gradient2(low = '#0053d3', high = '#b22709', midpoint=0,
```

```
                       aesthetics='fill')+
  guides(alpha = "none") +
  theme(panel.grid = element_blank(), panel.border = element_blank(),
        strip.background = element_blank(),strip.text.x = element_text(size=5),
        axis.ticks.x = element_blank(),
        axis.ticks.y=element_blank(),
        axis.text.x=element_blank(),
        axis.text.y = element_blank(),
        plot.background = element_rect(fill = "transparent", colour = NA))   +
  labs(fill=(expression(paste(Delta, paste(mu, "V")))),
       x=element_blank(),
       y=element_blank())

plot.lstm.agent.surp.pp <- summary_eu_agent_surplstm %>%
  ggplot(aes(x=x, y=y, fill=PP)) +
  geom_tile()+
  scale_fill_gradientn(colours = c('white','grey', 'black'),
                       limits = c(0.8, 1), na.value='white' ) +
  labs(fill=(expression(paste('P(|',Delta,mu, V, '| > 0)', ))),
       x=element_blank(),
       y=element_blank()) +
  theme(panel.grid = element_blank(),
        panel.border = element_blank(),
        strip.background = element_blank(),
        axis.ticks.x = element_blank(),
        axis.ticks.y=element_blank(),
        axis.text.x=element_blank(),
        axis.text.y = element_blank(),
        plot.background = element_rect(fill = "transparent", colour = NA))

summary_eu_agent_surplstm.conds <- preds.eu.agent.surplstm_long %>%
  filter(surprisal == 0) %>%
  group_by(x,y,samp) %>%
  mutate(diff_condition = value[condition == 'Abias'] -
           value[condition == 'nonAbias']) %>%
  ungroup() %>%
  group_by(x,y) %>%
  summarise(PP = ifelse(mean(diff_condition) > 0,
                        mean(diff_condition > 0),
                        mean(diff_condition < 0)),
            mean_diff = mean(diff_condition))

plot.lstm.agent.cond <- summary_eu_agent_surplstm.conds  %>%
  mutate(sig = ifelse(PP>0.80, 1, 0)) %>%
  ggplot(aes(x=x, y=y, fill=mean_diff, alpha = sig)) +
  geom_tile()+
  scale_fill_gradient2(low = '#0053d3', high = '#b22709', midpoint=0,
                       aesthetics='fill')+
  guides(alpha = "none") +
  theme(panel.grid = element_blank(), panel.border = element_blank(),
        strip.background = element_blank(),strip.text.x = element_text(size=5),
        axis.ticks.x = element_blank(),
        axis.ticks.y=element_blank(),
```

```
        axis.text.x=element_blank(),
        axis.text.y = element_blank(),
        plot.background = element_rect(fill = "transparent", colour = NA))   +
  labs(fill=(expression(paste(Delta, paste(mu, "V")))),
       x=element_blank(),
       y=element_blank())

plot.lstm.agent.cond.pp <- summary_eu_agent_surplstm.conds  %>%
  ggplot(aes(x=x, y=y, fill=PP)) +
  geom_tile()+
  scale_fill_gradientn(colours = c('white','grey', 'black'),limits = c(0.8, 1),
                      na.value='white' ) +
  guides(alpha = "none") +
  theme(panel.grid = element_blank(), panel.border = element_blank(),
        strip.background = element_blank(),strip.text.x = element_text(size=5),
        axis.ticks.x = element_blank(),
        axis.ticks.y=element_blank(),
        axis.text.x=element_blank(),
        axis.text.y = element_blank(),
        plot.background = element_rect(fill = "transparent", colour = NA))   +
  labs(fill=(expression(paste(Delta, paste(mu, "V")))),
       x=element_blank(),
       y=element_blank())
```

```
wrap_plots(list(plot.lstm.agent.cond, plot.lstm.agent.surp,
                plot.lstm.agent.cond.pp, plot.lstm.agent.surp.pp))
```

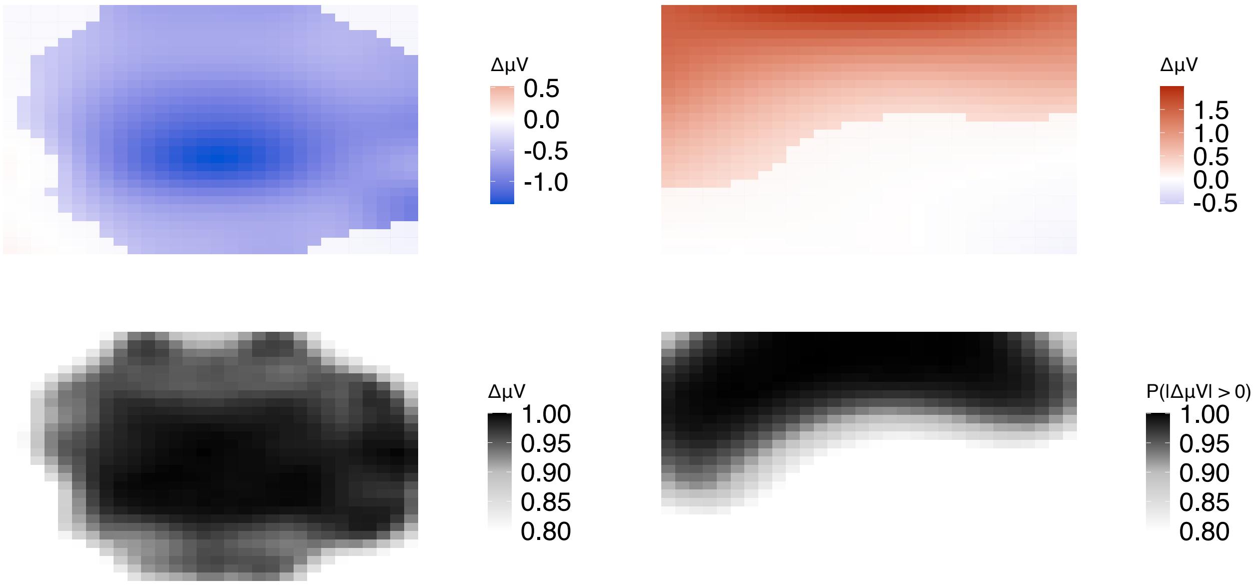

Figure S6: Left: Pair-wise fitted differences of μV for sentences with vs. sentences without the predicted reanalysis from model with LSTM surprisal as well as agent preference predictor. Right: Percentage of posterior probability mass of μV mean difference below or above 0.

S5.3.6 RoBERTa-based surprisal and Agent Preference

```
n_samps <- 4000
newdata.eu.agent.surptr <- newdata.eu.agent.surplstm %>%
  rename(surprisal.tr.Z =  surprisal.lstm.Z)

preds.eu.agent.surptr <- posterior_epred(model.eu.agent.surp.roberta,
                                         newdata=newdata.eu.agent.surptr,
                                         re.form=NA)

preds.eu.agent.surptr_long <- as.data.frame(preds.eu.agent.surptr) %>%
  mutate(samp = 1:nrow(preds.eu.agent.surptr)) %>%
  pivot_longer(-samp)

preds.eu.agent.surptr_long$x <- rep(newdata.eu.agent.surptr$x, n_samps)
preds.eu.agent.surptr_long$y <- rep(newdata.eu.agent.surptr$y, n_samps)
preds.eu.agent.surptr_long$surprisal <-
  rep(newdata.eu.agent.surptr$surprisal.tr.Z, n_samps)
preds.eu.agent.surptr_long$condition <-
  rep(newdata.eu.agent.surptr$condition, n_samps)

summary_eu_agent_surptr <- preds.eu.agent.surptr_long %>%
  filter(condition %in% 'Abias') %>%
  group_by(x,y,samp) %>%
  mutate(diff_surprisal_low_mean = value[surprisal == 2] -
           value[surprisal == -2]) %>%
  ungroup() %>%
  group_by(x,y) %>%
  summarise(PP = ifelse(mean(diff_surprisal_low_mean) > 0,
                        mean(diff_surprisal_low_mean > 0),
                        mean(diff_surprisal_low_mean < 0)),
            mean_diff = mean(diff_surprisal_low_mean)) %>%
  mutate('condition' = 'Abias')

plot.tr.agent.surp <- summary_eu_agent_surptr %>%
  mutate(sig = ifelse(PP>0.80, 1, 0)) %>%
  ggplot(aes(x=x, y=y, fill=mean_diff)) +
  geom_tile(aes(alpha = sig))+
  scale_fill_gradient2(low = '#0053d3', high = '#b22709',
                       midpoint=0, aesthetics='fill')+
  guides(alpha = "none") +
  theme(panel.grid = element_blank(), panel.border = element_blank(),
        strip.background = element_blank(),strip.text.x = element_text(size=5),
        axis.ticks.x = element_blank(),
        axis.ticks.y=element_blank(),
        axis.text.x=element_blank(),
        axis.text.y = element_blank(),
        plot.background = element_rect(fill = "transparent", colour = NA))   +
  labs(fill=(expression(paste(Delta, paste(mu, "V")))),
       x=element_blank(),
       y=element_blank())

plot.tr.agent.surp.pp <- summary_eu_agent_surptr %>%
```

```
  ggplot(aes(x=x, y=y, fill=PP)) +
  geom_tile()+
  scale_fill_gradientn(colours = c('white','grey', 'black'),
                       limits = c(0.8, 1), na.value='white' ) +
  labs(fill=(expression(paste('P(|',Delta,mu, V, '| > 0)', ))),
       x=element_blank(),
       y=element_blank()) +
  theme(panel.grid = element_blank(),
        panel.border = element_blank(),
        strip.background = element_blank(),
        axis.ticks.x = element_blank(),
        axis.ticks.y=element_blank(),
        axis.text.x=element_blank(),
        axis.text.y = element_blank(),
        plot.background = element_rect(fill = "transparent", colour = NA))

summary_eu_agent_surptr.condition <- preds.eu.agent.surptr_long %>%
  filter(surprisal == 0) %>%
  group_by(x,y,samp) %>%
  mutate(diff_condition = value[condition == 'Abias'] -
           value[condition == 'nonAbias']) %>%
  ungroup() %>%
  group_by(x,y) %>%
  summarise(PP = ifelse(mean(diff_condition) > 0,
                        mean(diff_condition > 0),
                        mean(diff_condition < 0)),
            mean_diff = mean(diff_condition))

plot.tr.agent.cond <- summary_eu_agent_surptr.condition %>%
  mutate(sig = ifelse(PP>0.80, 1, 0)) %>%
  ggplot(aes(x=x, y=y, fill=mean_diff, alpha = sig)) +
  geom_tile()+
  scale_fill_gradient2(low = '#0053d3', high = '#b22709', midpoint=0,
                       aesthetics='fill')+
  guides(alpha = "none") +
  theme(panel.grid = element_blank(), panel.border = element_blank(),
        strip.background = element_blank(),strip.text.x = element_text(size=5),
        axis.ticks.x = element_blank(),
        axis.ticks.y=element_blank(),
        axis.text.x=element_blank(),
        axis.text.y = element_blank(),
        plot.background = element_rect(fill = "transparent", colour = NA))   +
  labs(fill=(expression(paste(Delta, paste(mu, "V")))),
       x=element_blank(),
       y=element_blank())

plot.tr.agent.cond.pp <- summary_eu_agent_surptr.condition %>%
  ggplot(aes(x=x, y=y, fill=PP)) +
  geom_tile() +
  scale_fill_gradientn(colours = c('white','grey', 'black'),
                       limits = c(0.8, 1), na.value='white' ) +
  labs(fill=(expression(paste('P(|',Delta,mu, V, '| > 0)', ))),
       x=element_blank(),
```

```r
      y=element_blank()) +
  theme(panel.grid = element_blank(),
        panel.border = element_blank(),
        strip.background = element_blank(),
        axis.ticks.x = element_blank(),
        axis.ticks.y=element_blank(),
        axis.text.x=element_blank(),
        axis.text.y = element_blank(),
        plot.background = element_rect(fill = "transparent", colour = NA))
```

```r
wrap_plots(list(plot.tr.agent.cond, plot.tr.agent.surp,
                plot.tr.agent.cond.pp, plot.tr.agent.surp.pp))
```

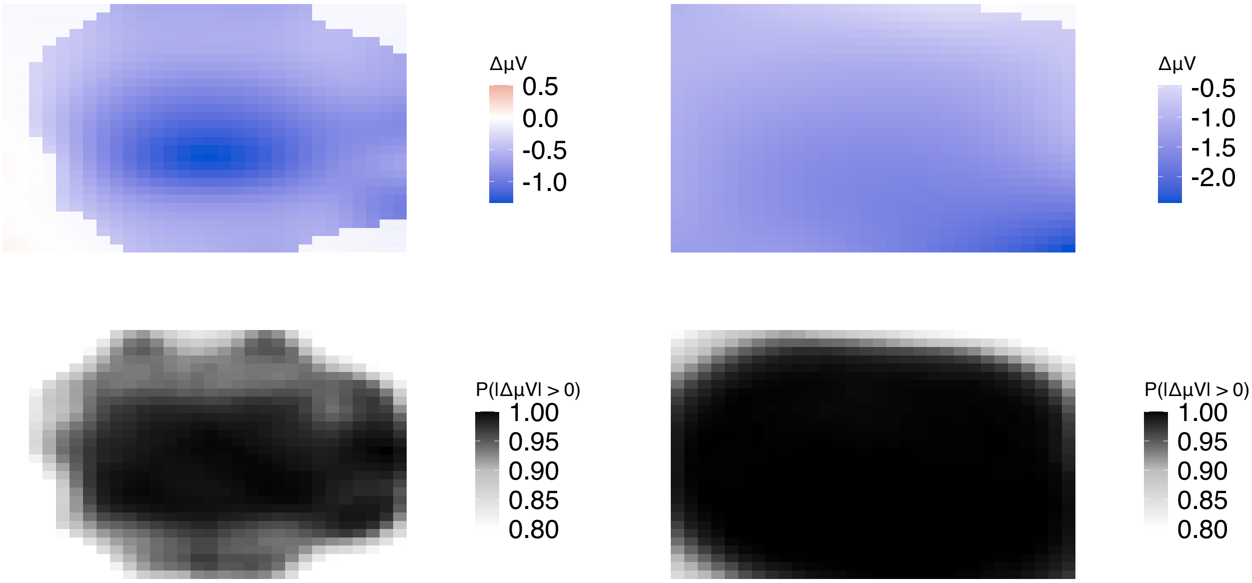

Figure S7: Left: Pair-wise fitted differences of μV for sentences with vs. sentences without the predicted reanalysis from model with RoBERTa surprisal as well as agent preference predictor. Right: Percentage of posterior probability mass of μV mean difference below or above 0.

S5.3.7 GPT-based surprisal and Agent Preference

```r
n_samps <- 4000
newdata.eu.agent.surpgpt <- newdata.eu.agent.surplstm %>%
  rename(surprisal.gpt.Z =  surprisal.lstm.Z)
preds.eu.agent.surpgpt <- posterior_epred(model.eu.agent.surp.gpt,
                                          newdata=newdata.eu.agent.surpgpt,
                                          re.form=NA)

preds.eu.agent.surpgpt_long <- as.data.frame(preds.eu.agent.surpgpt) %>%
  mutate(samp = 1:nrow(preds.eu.agent.surpgpt)) %>%
  pivot_longer(-samp)

preds.eu.agent.surpgpt_long$x <- rep(newdata.eu.agent.surpgpt$x, n_samps)
```

```r
preds.eu.agent.surpgpt_long$y <- rep(newdata.eu.agent.surpgpt$y, n_samps)
preds.eu.agent.surpgpt_long$surprisal <-
  rep(newdata.eu.agent.surpgpt$surprisal.gpt.Z, n_samps)
preds.eu.agent.surpgpt_long$condition <-
  rep(newdata.eu.agent.surpgpt$condition, n_samps)

summary_eu_agent_surpgpt <- preds.eu.agent.surpgpt_long %>%
  filter(condition %in% 'Abias') %>%
  group_by(x,y,samp) %>%
  mutate(diff_surprisal_low_mean = value[surprisal == 2] -
           value[surprisal == -2]) %>%
  ungroup() %>%
  group_by(x,y) %>%
  summarise(PP = ifelse(mean(diff_surprisal_low_mean) > 0,
                        mean(diff_surprisal_low_mean > 0),
                        mean(diff_surprisal_low_mean < 0)),
            mean_diff = mean(diff_surprisal_low_mean)) %>%
  mutate('condition' = 'Abias')

plot.gpt.agent.surp <- summary_eu_agent_surpgpt %>%
  mutate(sig = ifelse(PP>0.80, 1, 0)) %>%
  ggplot(aes(x=x, y=y, fill=mean_diff)) +
  geom_tile(aes(alpha = sig))+
  scale_fill_gradient2(low = '#0053d3', high = '#b22709', midpoint=0,
                       aesthetics='fill')+
  guides(alpha = "none") +
  theme(panel.grid = element_blank(), panel.border = element_blank(),
        strip.background = element_blank(),strip.text.x = element_text(size=5),
        axis.ticks.x = element_blank(),
        axis.ticks.y=element_blank(),
        axis.text.x=element_blank(),
        axis.text.y = element_blank(),
        plot.background = element_rect(fill = "transparent", colour = NA))   +
  labs(fill=(expression(paste(Delta, paste(mu, "V")))),
       x=element_blank(),
       y=element_blank())

plot.gpt.agent.surp.pp <- summary_eu_agent_surpgpt %>%
  ggplot(aes(x=x, y=y, fill=PP)) +
  geom_tile()+
  scale_fill_gradientn(colours = c('white','grey', 'black'),
                       limits = c(0.8, 1), na.value='white' ) +
  labs(fill=(expression(paste('P(|',Delta,mu, V, '| > 0)', ))),
       x=element_blank(),
       y=element_blank()) +
  theme(panel.grid = element_blank(),
        panel.border = element_blank(),
        strip.background = element_blank(),
        axis.ticks.x = element_blank(),
        axis.ticks.y=element_blank(),
        axis.text.x=element_blank(),
        axis.text.y = element_blank(),
        plot.background = element_rect(fill = "transparent", colour = NA))
```

```r
summary_eu_agent_surpgpt.condition <- preds.eu.agent.surpgpt_long %>%
  filter(surprisal == 0) %>%
  group_by(x,y,samp) %>%
  mutate(diff_condition = value[condition == 'Abias'] -
           value[condition == 'nonAbias']) %>%
  ungroup() %>%
  group_by(x,y) %>%
  summarise(PP = ifelse(mean(diff_condition) > 0,
                        mean(diff_condition > 0),
                        mean(diff_condition < 0)),
            mean_diff = mean(diff_condition))

plot.gpt.agent.cond <- summary_eu_agent_surpgpt.condition %>%
  mutate(sig = ifelse(PP>0.80, 1, 0)) %>%
  ggplot(aes(x=x, y=y, fill=mean_diff, alpha = sig)) +
  geom_tile()+
  scale_fill_gradient2(low = '#0053d3', high = '#b22709', midpoint=0,
                       aesthetics='fill')+
  guides(alpha = "none") +
  theme(panel.grid = element_blank(), panel.border = element_blank(),
        strip.background = element_blank(),strip.text.x = element_text(size=5),
        axis.ticks.x = element_blank(),
        axis.ticks.y=element_blank(),
        axis.text.x=element_blank(),
        axis.text.y = element_blank(),
        plot.background = element_rect(fill = "transparent", colour = NA))   +
  labs(fill=(expression(paste(Delta, paste(mu, "V")))),
       x=element_blank(),
       y=element_blank())

plot.gpt.agent.cond.pp <- summary_eu_agent_surpgpt.condition %>%
  ggplot(aes(x=x, y=y, fill=PP)) +
  geom_tile() +
  scale_fill_gradientn(colours = c('white','grey', 'black'),
                       limits = c(0.8, 1), na.value='white' ) +
  labs(fill=(expression(paste('P(|',Delta,mu, V, '| > 0)', ))),
       x=element_blank(),
       y=element_blank()) +
  theme(panel.grid = element_blank(),
        panel.border = element_blank(),
        strip.background = element_blank(),
        axis.ticks.x = element_blank(),
        axis.ticks.y=element_blank(),
        axis.text.x=element_blank(),
        axis.text.y = element_blank(),
        plot.background = element_rect(fill = "transparent", colour = NA))
```

```r
wrap_plots(list(plot.gpt.agent.cond, plot.gpt.agent.surp,
                plot.gpt.agent.cond.pp, plot.gpt.agent.surp.pp))
```

S5.3.8 Strongest effect summary

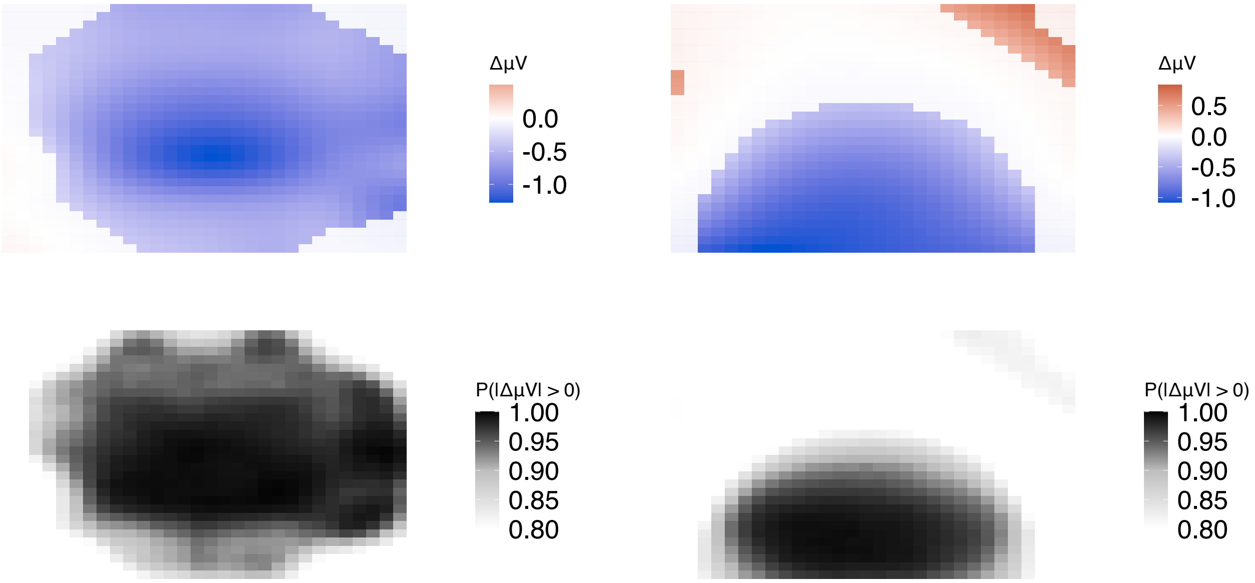

Figure S8: Left: Pair-wise fitted differences of μV for sentences with vs. sentences without the predicted reanalysis from model with GPT-2 surprisal as well as agent preference predictor. Right: Percentage of posterior probability mass of μV mean difference below or above 0.

Table S1: Basque: This table contains the lowest mean delta microVolt (i.e. strongest effect size across the scalp, blue in the plots above) and the maximum proportion of the posterior distribution that is lower than 0 for the highest-ranked model, agent preference + bert surprisal.

predictor	$min(mean(\Delta\mu V))$	$max(P\Delta\mu V < 0)$
surprisal RoBERTa	-2.43	1
agent preference condition	-1.33	1

S6 References

6 Residual Slopes in ERP models

S6: Residual Slopes in ERP models*

Eva Huber Sebastian Sauppe Arrate Isasi-Isasmendi
Ina Bornkessel-Schlesewsky Paola Merlo Balthasar Bickel

August 29, 2023

S1 Rationale

Because the GAM models didn't converge with random slopes for participants and items we visualize here the residual errors of participants and items by conditions. There is no apparent trend that would suggest that the residuals correlate in a systematic way with the conditions of interest, suggesting that the lack of random slopes does not inflate the effects of the conditions.

Note that part of the variation by participant and item is captured by the smooth surfaces (see `t2(x, y, Participant, bs = "re")` and `t2(x, y, Stimulus, bs = "re")` in the models fitted in the files `German_analysis2.{R,pdf}`, `Hindi_analysis2.{R,pdf}` and `Basque_analysis2.{R,pdf}` and S3-S5 respectively.).

S1.1 German

```
data.de <- readRDS('../results/german_mv_surprisal_second.RDS')

data.de$obs <- as.character(1:nrow(data.de))

model.de.agent.surplstm <-
  readRDS('../gams_fitted_models/gam_de_agent_lstm.RDS')

ppc_de_samp <- posterior_predict(model.de.agent.surplstm)
ppc_de_samp <- ppc_de_samp[1:100,]

colnames(ppc_de_samp) <- 1:nrow(data.de)

pp_df_de <- ppc_de_samp %>%
  as.data.frame() %>%
  mutate(sample = 1:n()) %>%
  pivot_longer(-sample, values_to = "prediction", names_to = "obs") %>%
  left_join(data.de) %>%
  mutate(residual = mV - prediction) %>%
  separate(sent_id, c('sent_id', 'condition_id'), sep=':')

pp_df_de <- pp_df_de %>%
```

*This document was `rmarkdown::render`'ed from an `R` script 'main_scripts/residuals_slopes.R' available at https://osf.io/hbj67

```r
  mutate(surprisal_binary = ifelse(surprisal.lstm.Z > 0, 'high', 'low'))

pp_df_de$surprisal <- as.factor(paste(pp_df_de$surprisal_binary))
pp_df_de$sent_id <- as.factor(pp_df_de$sent_id)

pp_df_de <- pp_df_de %>%
  unite(trial_id, c('trial_number', 'Subject'), sep="_", remove=F)
```

```r
pp_df_de %>% ggplot(aes(x=condition, y = residual)) +
  stat_slab(colour = 'black', size = 0.5, fill = NA) +
  facet_wrap(~Participant) +
  geom_hline(aes(yintercept = 0), linetype = "dashed")
```

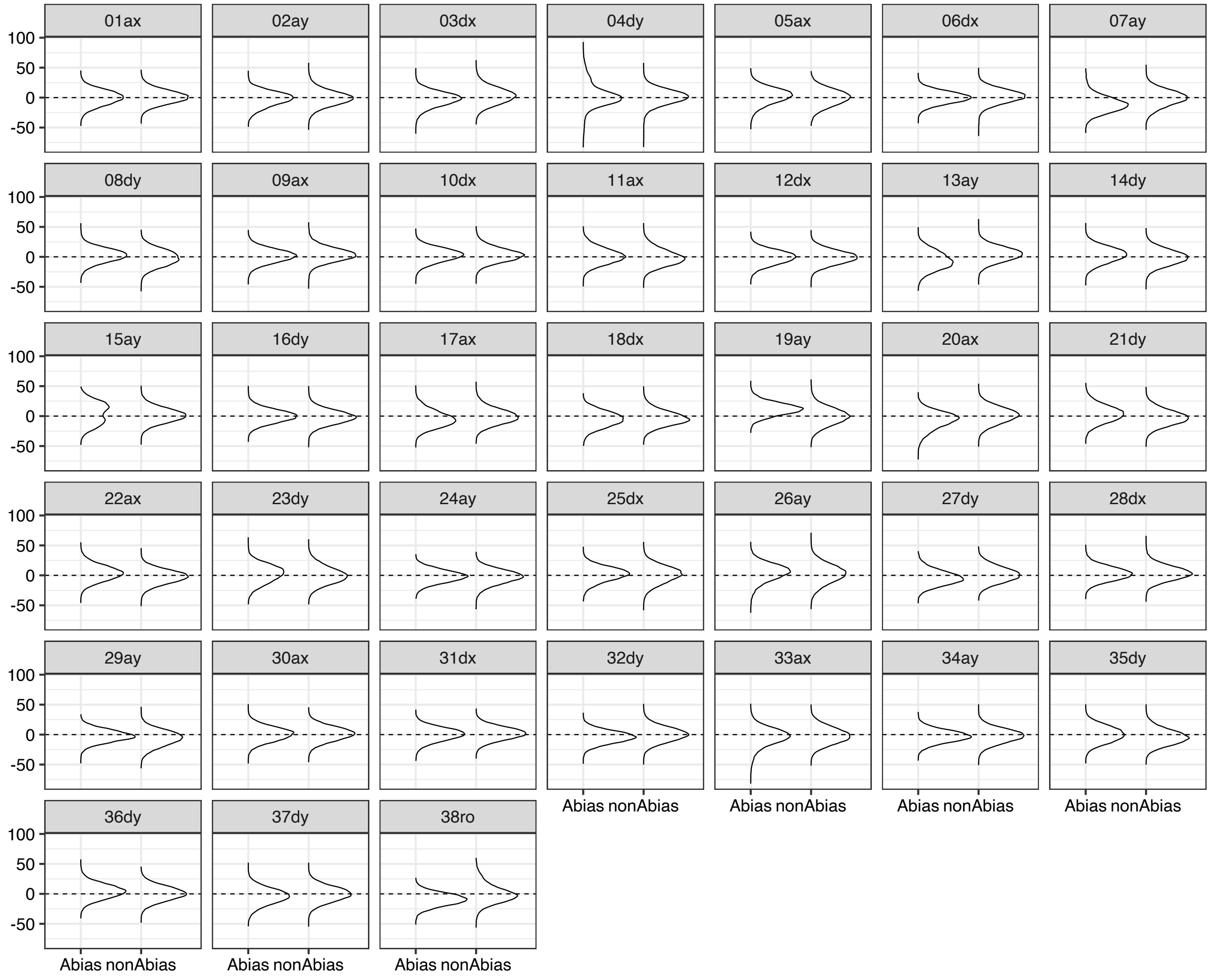

Figure S1: German: Residuals for each participant by condition

```r
pp_df_de %>% ggplot(aes(x=surprisal, y = residual)) +
  stat_slab(colour = 'black', size = 0.5, fill = NA) +
  facet_wrap(~Participant) +
  geom_hline(aes(yintercept = 0), linetype = "dashed")
```

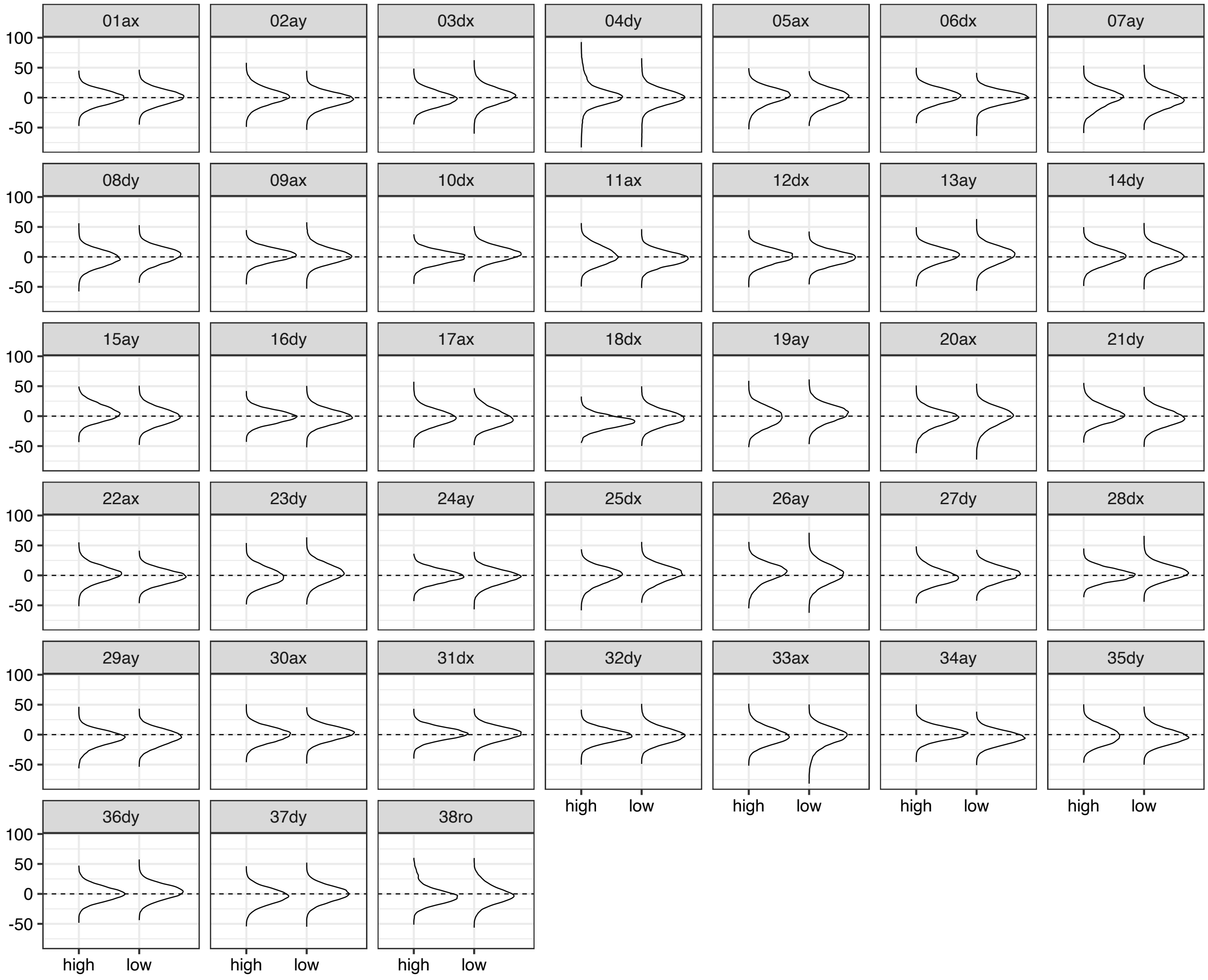

Figure S2: German: Residuals for each participant by surprisal

```
pp_df_de %>% ggplot(aes(x = condition, y = residual)) +
  stat_slab(colour = 'black', size = 0.5, fill = NA) +
  geom_hline(aes(yintercept = 0), linetype = "dashed") +
  facet_wrap(~sent_id)
```

```
pp_df_de %>% ggplot(aes(x = surprisal, y = residual)) +
  stat_slab(colour = 'black', size = 0.5, fill = NA) +
  geom_hline(aes(yintercept = 0), linetype = "dashed") +
  facet_wrap(~sent_id)
```

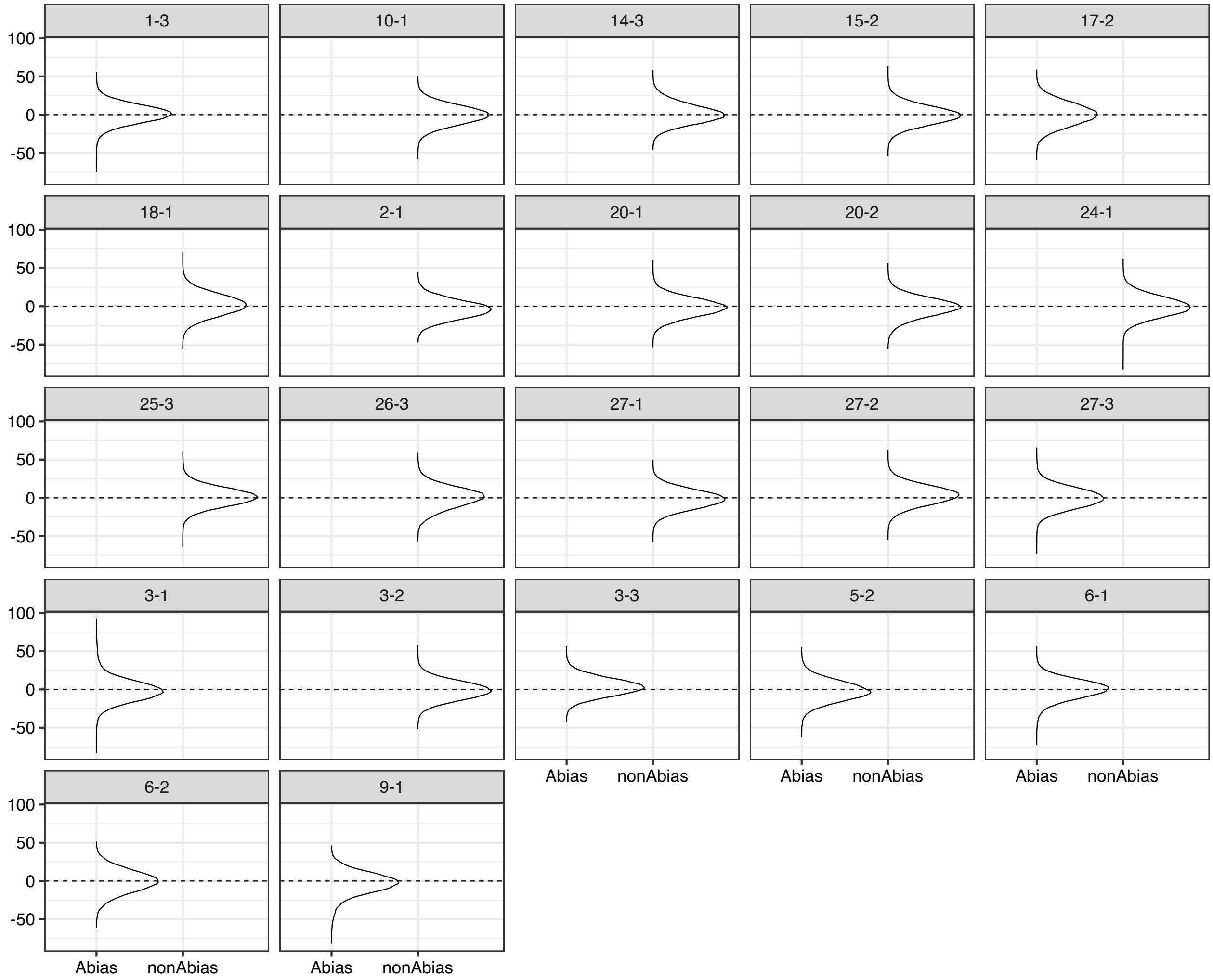

Figure S3: German: Residuals for each stimulus item by condition

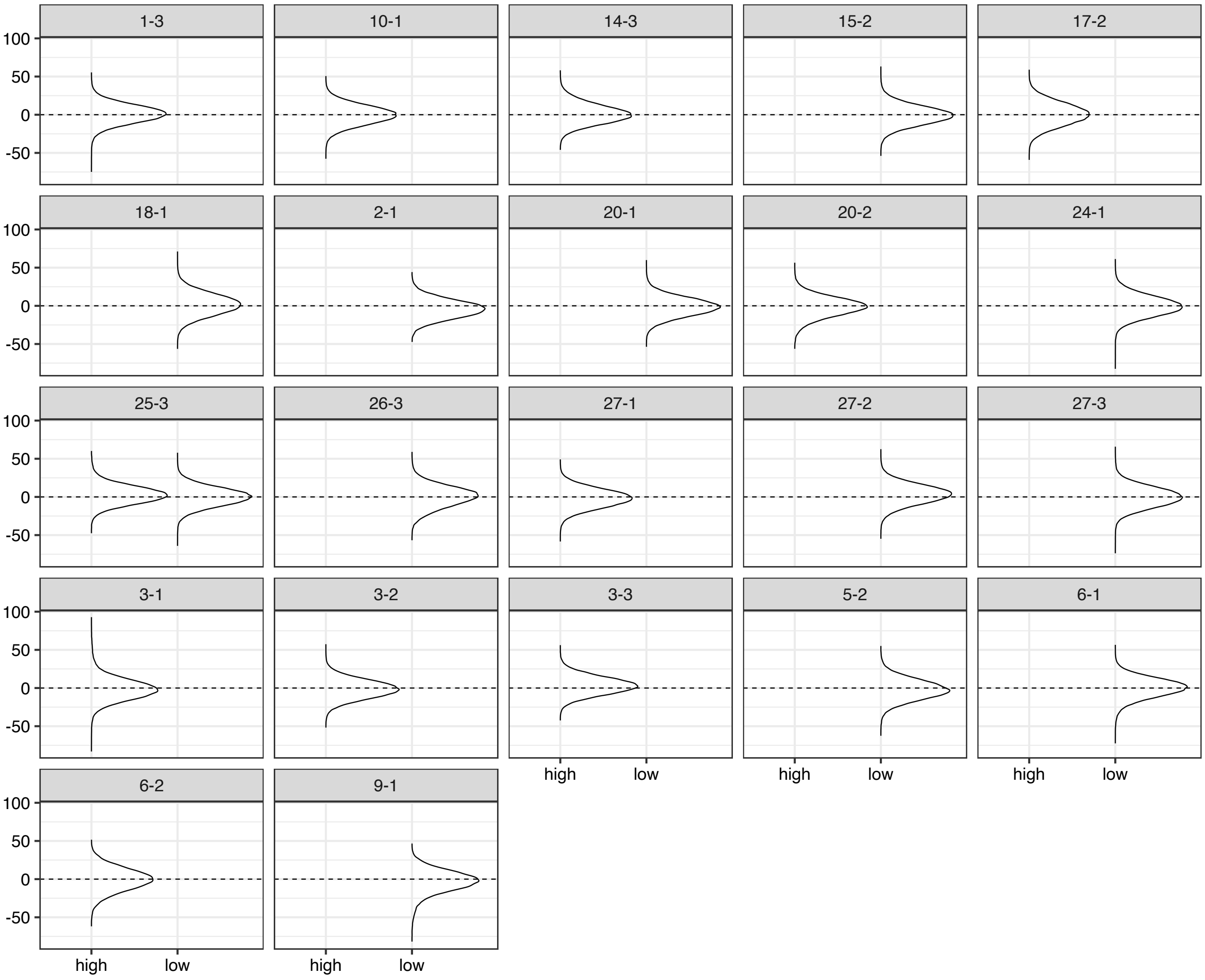

Figure S4: German: Residuals for each stimulus by surprisal

S1.2 Hindi

```
data.hi <- readRDS('../results/hindi_mv_surprisal_0506.RDS')

model.hi.agent.surpbert <-
  readRDS('../gams_fitted_models/gam_hi_agent_bert.RDS')

data.hi$obs <- as.character(1:nrow(data.hi))

ppc_hi_samp <- posterior_predict(model.hi.agent.surpbert, ndraws = 100)
ppc_hi_samp <- ppc_hi_samp[1:100,]
colnames(ppc_hi_samp) <- 1:nrow(data.hi)

pp_df_hi <- ppc_hi_samp %>%
  as.data.frame() %>%
  mutate(sample = 1:n()) %>%
  pivot_longer(-sample, values_to = "prediction", names_to = "obs") %>%
  left_join(data.hi) %>%
  mutate(residual = mV - prediction)

pp_df_hi <- pp_df_hi %>%
  mutate(surprisal_binary = ifelse(surprisal.tr.Z > 0, 'high', 'low'))

pp_df_hi$surprisal <- as.factor(paste(pp_df_hi$surprisal_binary))

pp_df_hi <- pp_df_hi %>%
  unite(trial_id, c('trial_number', 'Subject'), sep="_", remove=F)
```

```
pp_df_hi %>% ggplot(aes(x=condition, y = residual)) +
  stat_slab(colour = 'black', size = 0.5, fill = NA) +
  facet_wrap(~Participant) +
  geom_hline(aes(yintercept = 0), linetype = "dashed")
```

```
pp_df_hi %>% ggplot(aes(x=surprisal, y = residual)) +
  stat_slab(colour = 'black', size = 0.5, fill = NA) +
  facet_wrap(~Participant) +
  geom_hline(aes(yintercept = 0), linetype = "dashed")
```

```
pp_df_hi %>% ggplot(aes(x = condition , y = residual)) +
  stat_slab(colour = 'black', size = 0.5, fill = NA) +
  geom_hline(aes(yintercept = 0), linetype = "dashed") +
  facet_wrap(~sent_id)
```

```
pp_df_hi %>% ggplot(aes(x = surprisal, y = residual)) +
  stat_slab(colour = 'black', size = 0.5, fill = NA) +
  geom_hline(aes(yintercept = 0), linetype = "dashed") +
  facet_wrap(~sent_id)
```

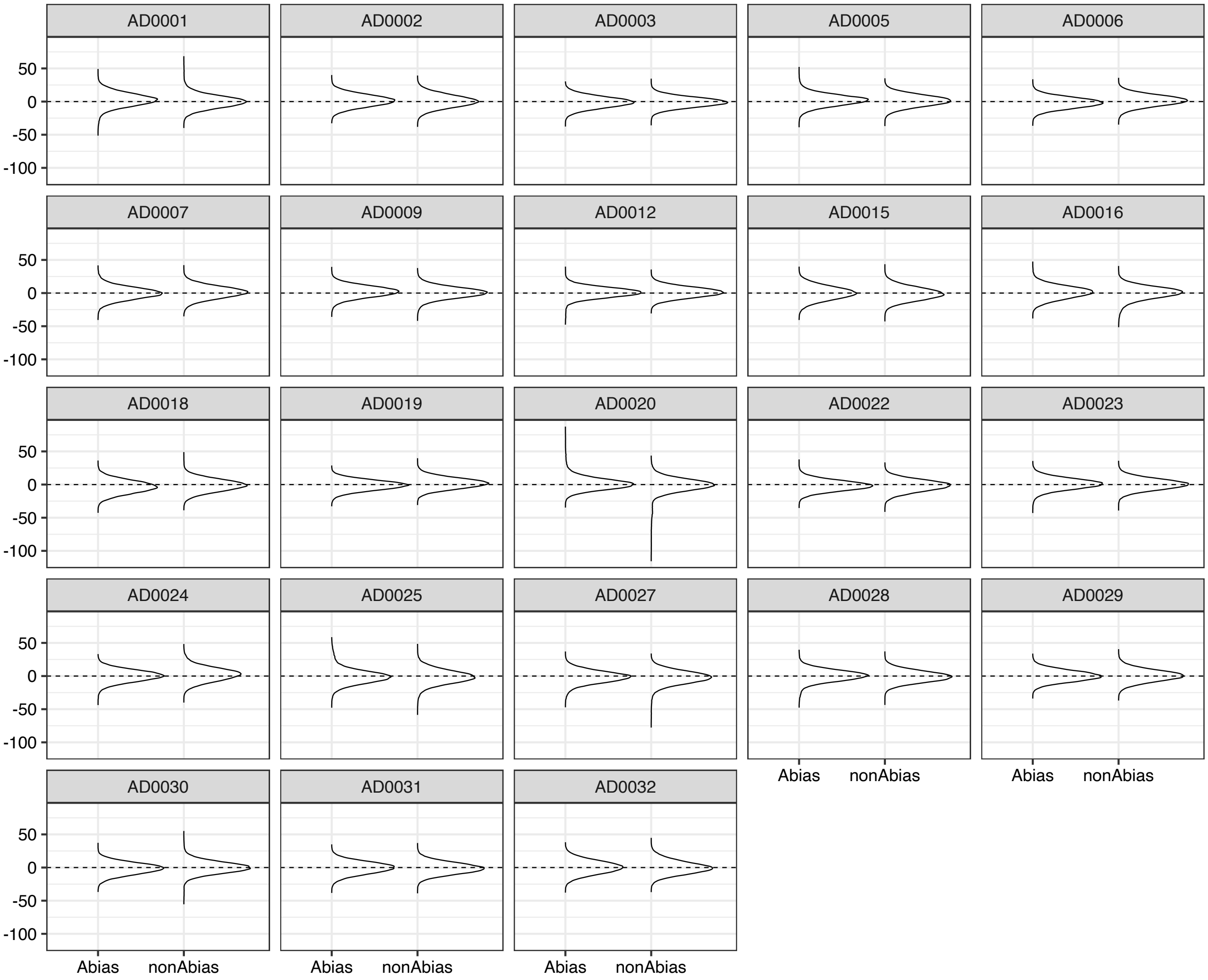

Figure S5: Hindi: Residuals for each participant by condition

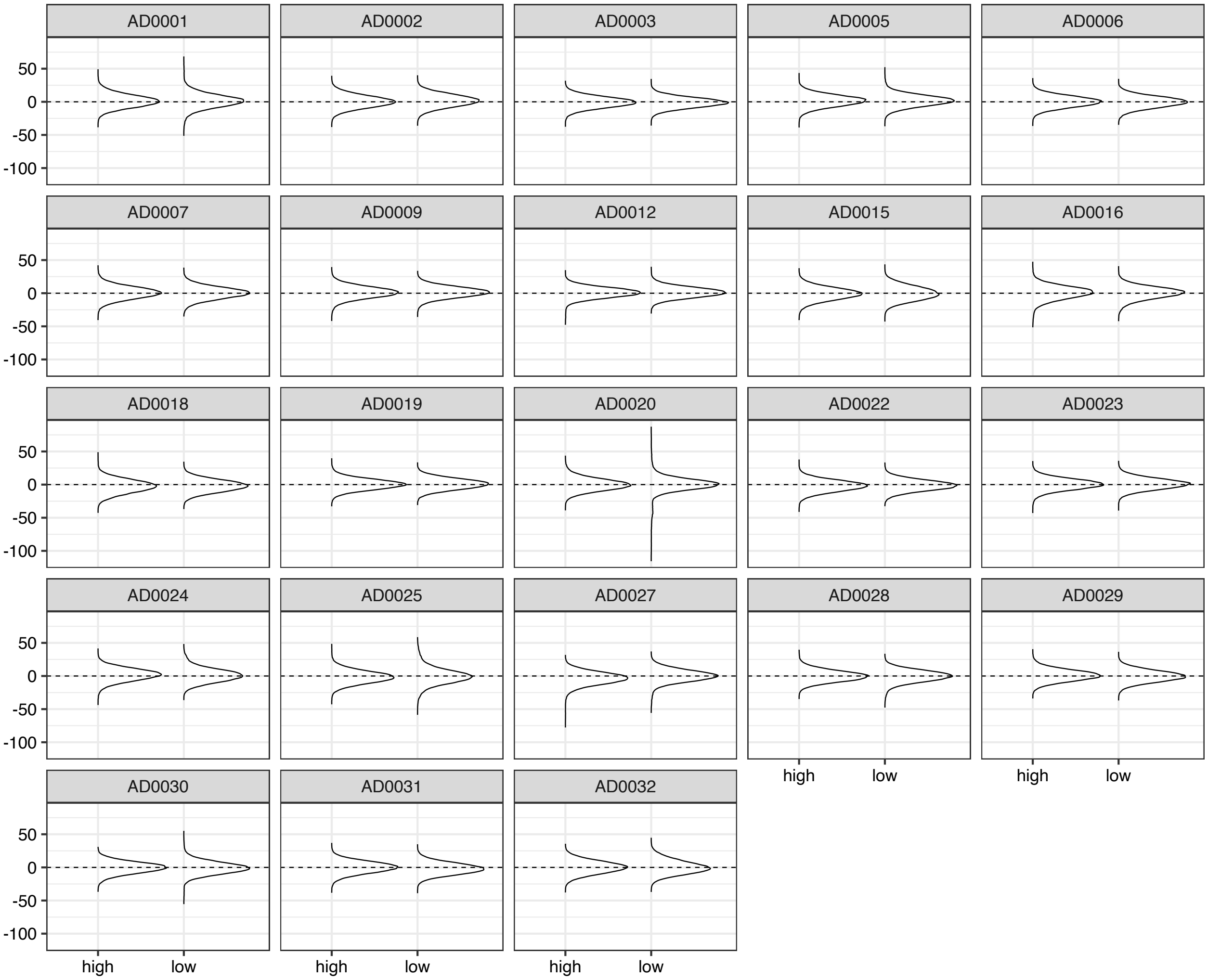

Figure S6: Hindi: Residuals for each participant by surprisal

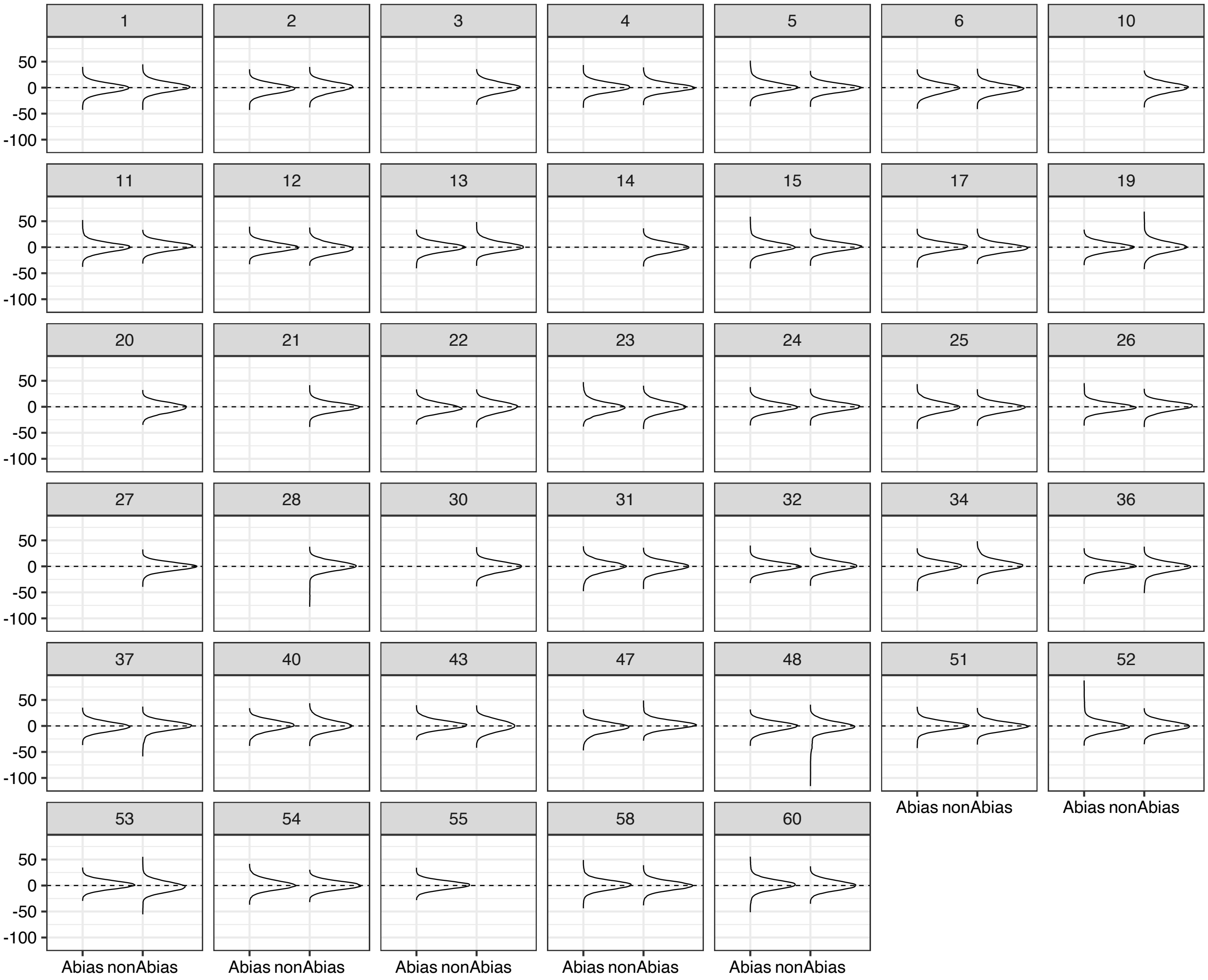

Figure S7: Hindi: Residuals for each stimulus item by condition

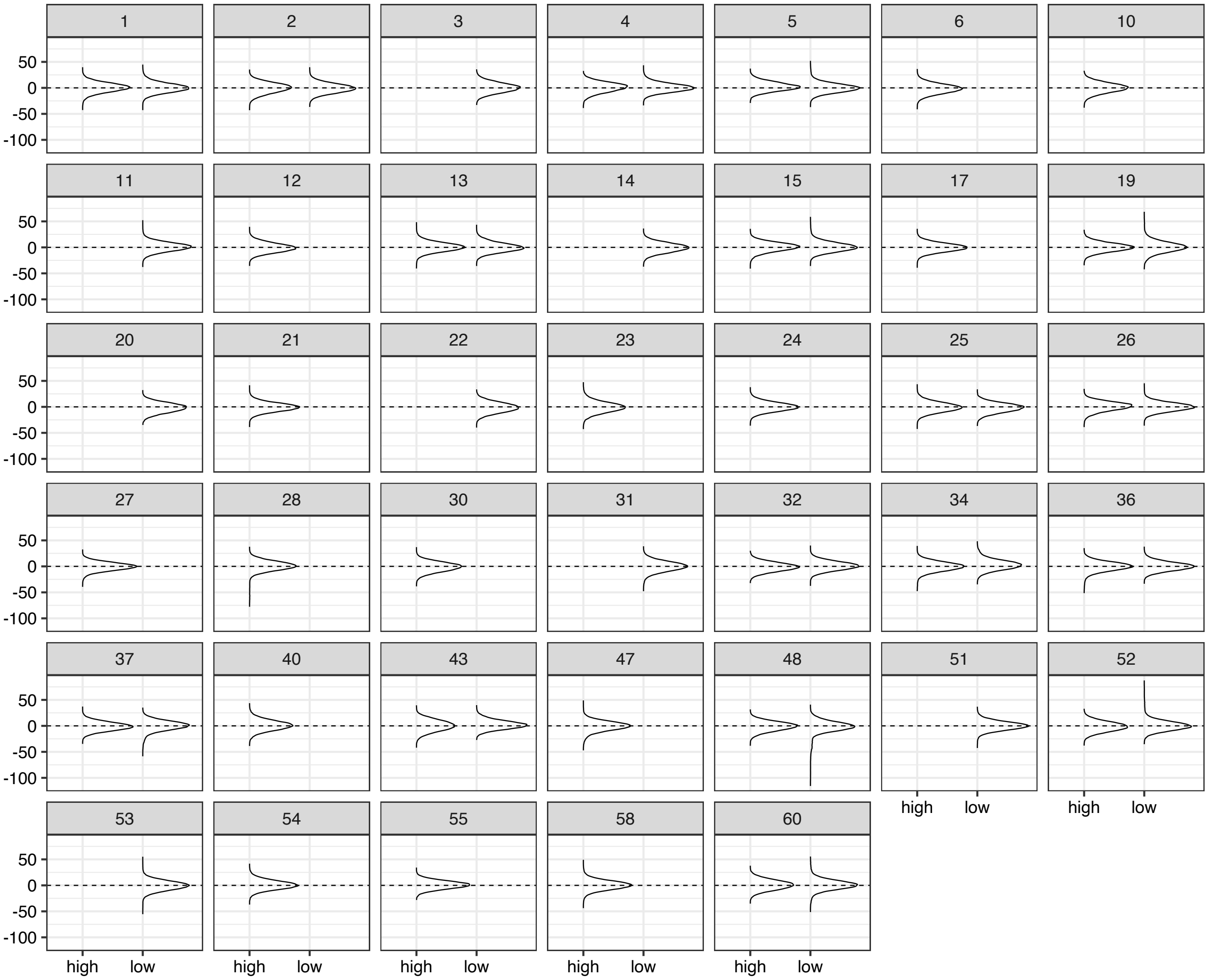

Figure S8: Hindi: Residuals for each stimulus item by surprisal

S1.3 Basque

```
data.eu <- readRDS('../results/basque_mv_surprisal_new.RDS')

model.eu.agent.surproberta <-
  readRDS('../gams_fitted_models/gam_eu_agent_roberta.RDS')

data.eu$obs <- as.character(1:nrow(data.eu))

ppc_eu_samp <- posterior_predict(model.eu.agent.surproberta, ndraws = 100)
ppc_eu_samp <- ppc_eu_samp[1:100,]
colnames(ppc_eu_samp) <- 1:nrow(data.eu)

pp_df_eu <- ppc_eu_samp %>%
  as.data.frame() %>%
  mutate(sample = 1:n()) %>%
  pivot_longer(-sample, values_to = "prediction", names_to = "obs") %>%
  left_join(data.eu) %>%
  mutate(residual = mV - prediction) %>%
  separate(sent_id, c('sent_id', 'condition_id'), sep='_')

pp_df_eu$sent_id <- as.factor(paste(pp_df_eu$sent_id))

pp_df_eu <- pp_df_eu %>%
  mutate(surprisal_binary = ifelse(surprisal.tr.Z > 0, 'high', 'low'))

pp_df_eu$surprisal <- as.factor(paste(pp_df_eu$surprisal_binary))

pp_df_eu <- pp_df_eu %>%
  unite(trial_id, c('trial_number', 'Subject'), sep="_", remove=F)
```

```
pp_df_eu %>% ggplot(aes(x=condition, y = residual)) +
  stat_slab(colour = 'black', size = 0.5, fill = NA) +
  facet_wrap(~Participant) +
  geom_hline(aes(yintercept = 0), linetype = "dashed")
```

```
pp_df_eu %>% ggplot(aes(x=surprisal, y = residual)) +
  stat_slab(colour = 'black', size = 0.5, fill = NA) +
  facet_wrap(~Participant) +
  geom_hline(aes(yintercept = 0), linetype = "dashed")
```

```
pp_df_eu <- pp_df_eu %>% separate(sent_id, c('sent_id', 'cond_id'), sep='_')

pp_df_eu %>% ggplot(aes(x = condition, y = residual)) +
  stat_slab(colour = 'black', size = 0.5, fill = NA) +
  geom_hline(aes(yintercept = 0), linetype = "dashed") +
  facet_wrap(~sent_id)+
  theme(axis.text.x = element_text(size = 9))
```

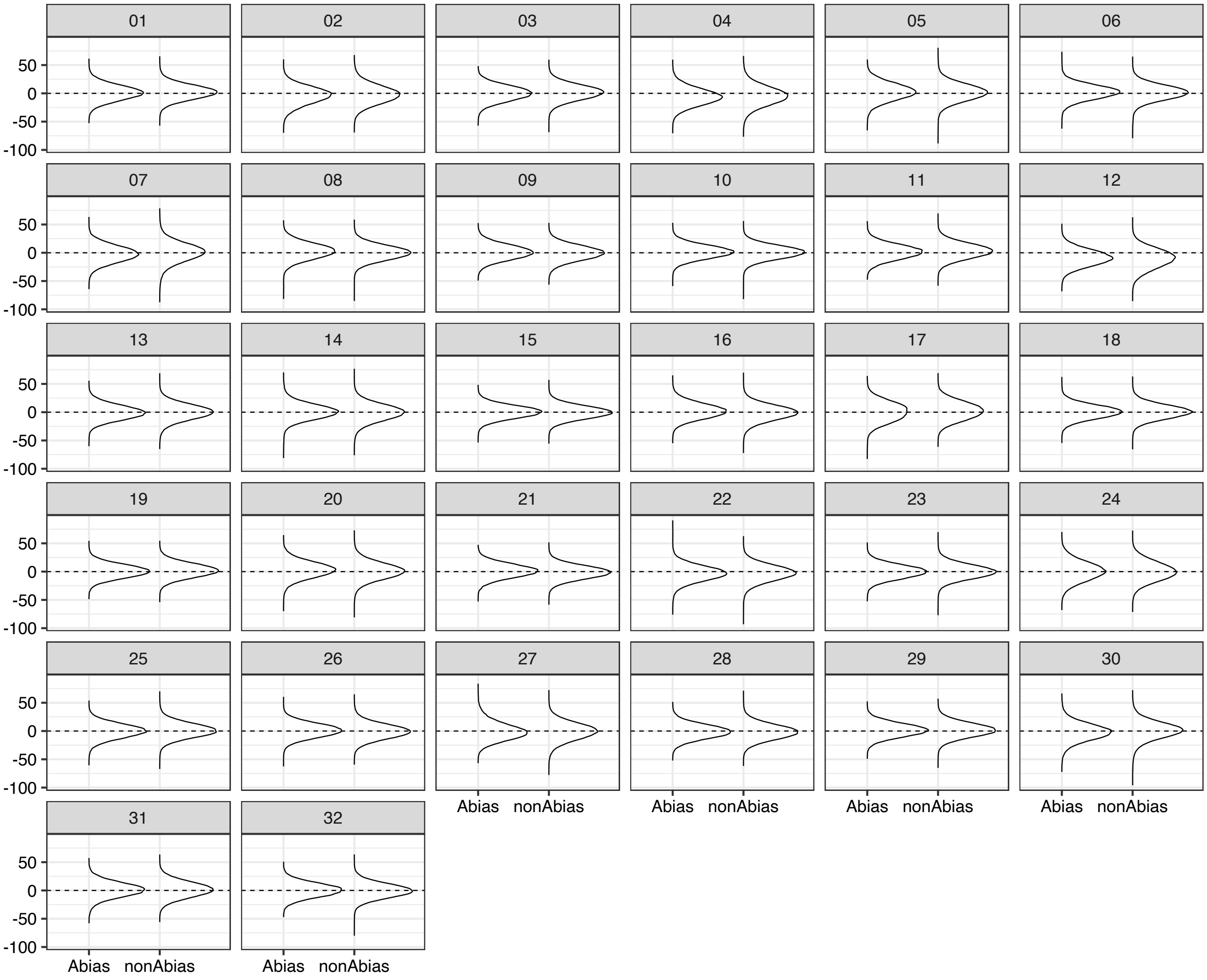

Figure S9: Basque: Residuals for each participant by condition

```
pp_df_eu %>% ggplot(aes(x = surprisal, y = residual)) +
  stat_slab(colour = 'black', size = 0.5, fill = NA) +
  geom_hline(aes(yintercept = 0), linetype = "dashed") +
  facet_wrap(~sent_id)
```

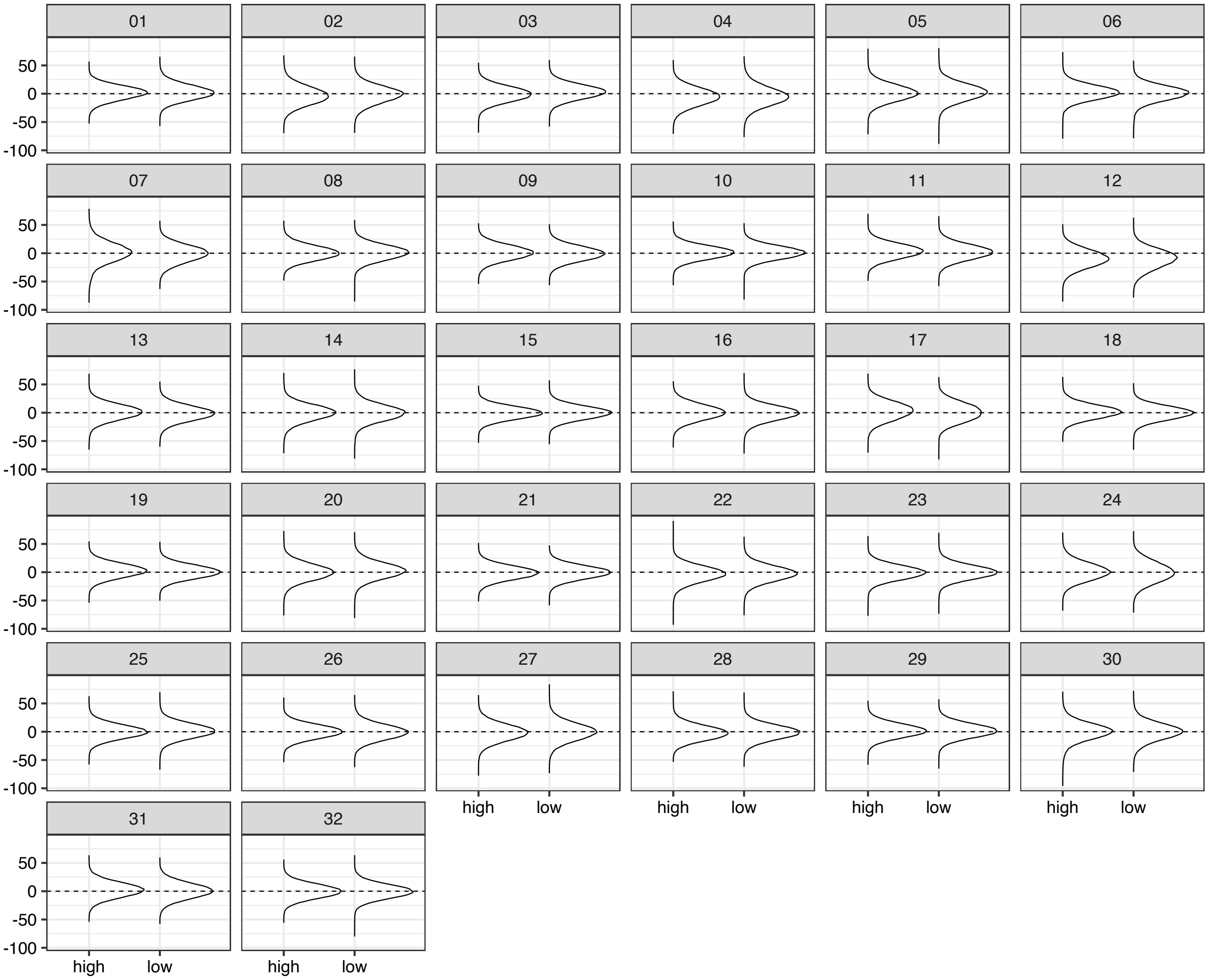

Figure S10: Basque: Residuals for each participant by surprisal

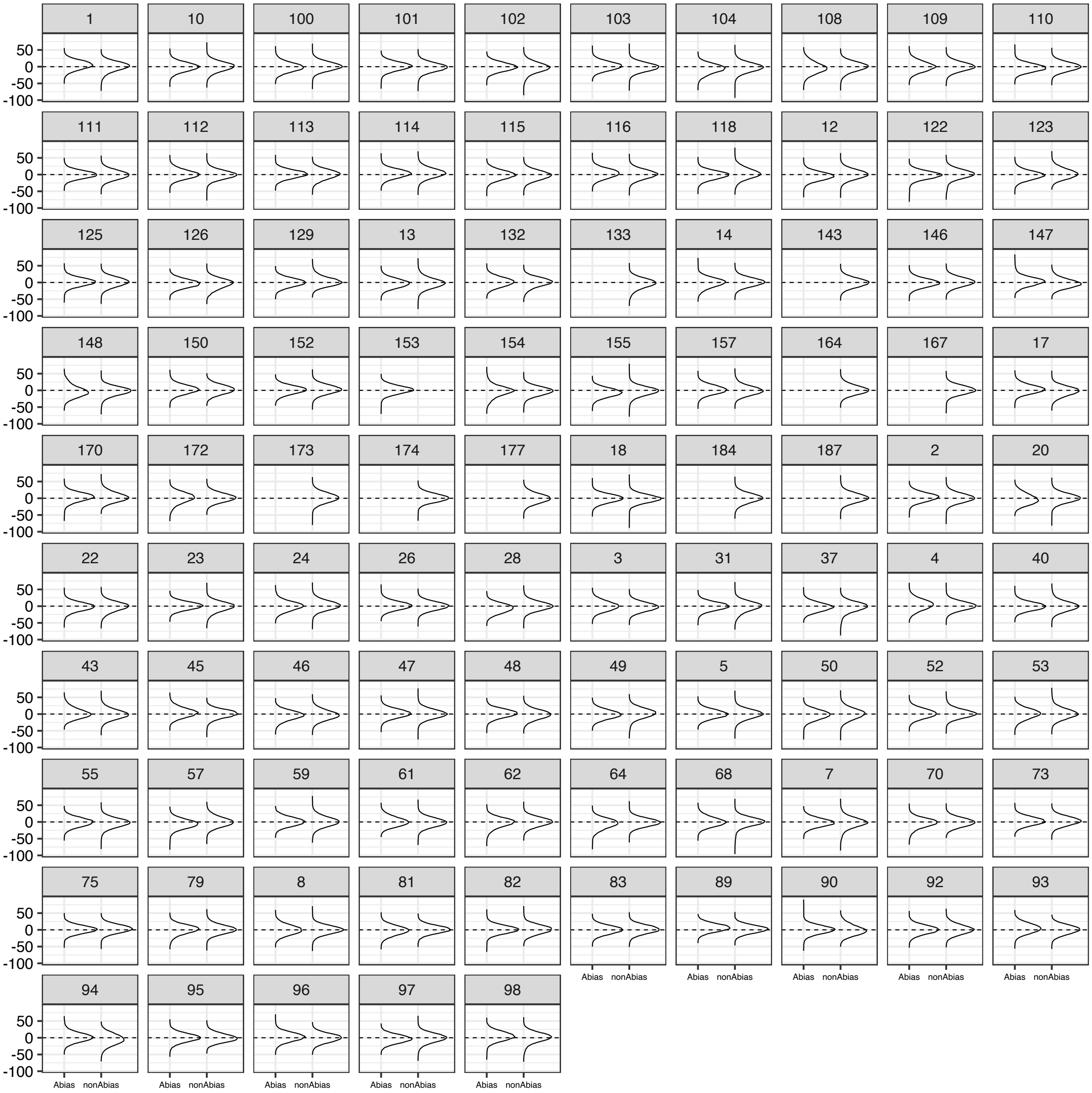

Figure S11: Basque: Residuals for each stimulus item by condition

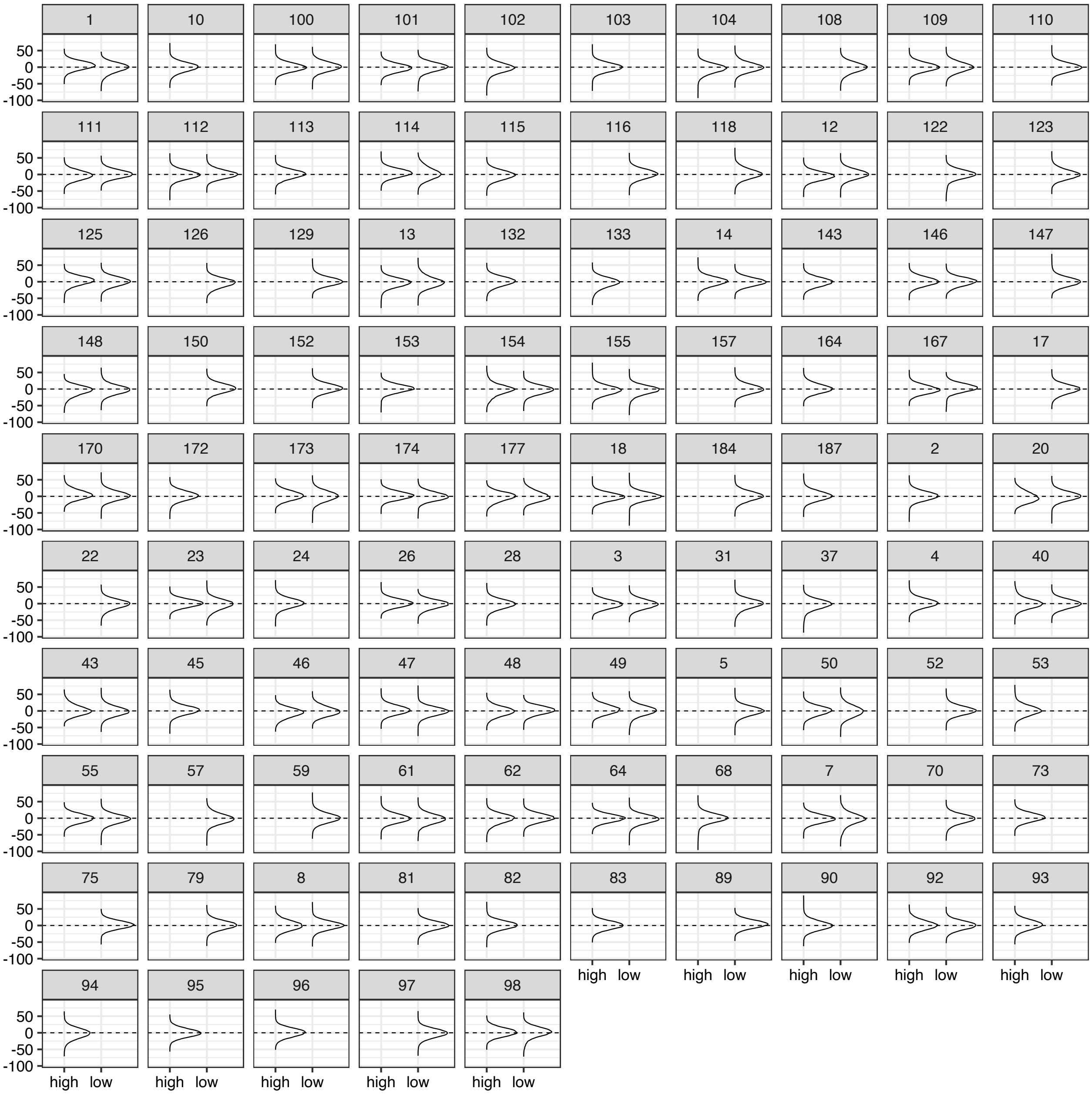

Figure S12: Basque: Residuals for each stimulus item by surprisal

7 Single Electrode Analyses

|S7: Single Electrode Analysis*

Eva Huber Sebastian Sauppe Arrate Isasi-Isasmendi
Ina Bornkessel-Schlesewsky Paola Merlo Balthasar Bickel

August 29, 2023

S7 Single Electrode Analysis

S7.1 Rationale

As noted in the main text, at the spatial resolution we have, the GAM approach cannot completely separate actual trends in the EEG signal from remnant autocorrelations (Simpson 2018, *Frontiers in Ecology and Evolution*). As a robustness analysis we model here the signal in those electrodes that the GAM analysis identified as showing the strongest effects. Effects and their posterior probabilities are expected to be lower because single electrodes only capture a subset of the relevant electrophysiological signal.

S7.2 German

```
de_data.df <- readRDS('../results/german_mv_surprisal_second.RDS')
```

*This document was `rmarkdown::render`'ed from an `R` script 'main_scripts/Single_electrode_analyses.R' available at https://osf.io/hbj67

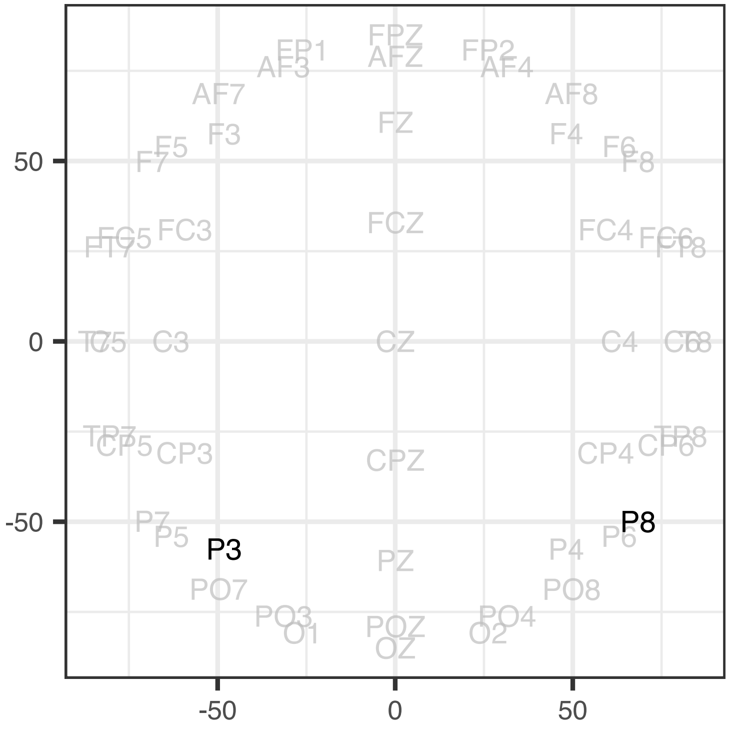

Figure S1: Electrode positions in the German experiment

```r
m.de <- mV ~ surprisal.lstm.Z + condition + trial_number.Z +
  (1 + condition + surprisal.lstm.Z | Participant) +
  (1 + surprisal.lstm.Z + condition | sent_id)

de_lstm_P8.m <-
  brm(formula = m.de,
    family = gaussian(),
    data = filter(de_data.df, electrode %in% "P8"),
    file = '../models_single_electrode/de_lstm_P8',
    control = list(adapt_delta = 0.9),
    cores = detectCores()-2
  )

de_lstm_P8.m %>%
  emmeans( ~ condition, # averaging over the other conditions
          epred = T) %>%
  contrast(method = 'pairwise') %>%
  gather_emmeans_draws() %>%
  summarise(PP = mean(.value<0), meanDelta = mean(.value))
```

```
## # A tibble: 1 x 3
##   contrast            PP meanDelta
##   <chr>            <dbl>     <dbl>
## 1 Abias - nonAbias 0.821     -1.01
```

```r
de_lstm_P3.m <-
  brm(formula = m.de,
      family = gaussian(),
      data = filter(de_data.df, electrode %in% "P3"),
      file = '../models_single_electrode/de_lstm_P3',
      control = list(adapt_delta = 0.9),
      cores = detectCores()-2
  )

de_lstm_P3.m %>%
  emmeans( ~ surprisal.lstm.Z,
          at = list(surprisal.lstm.Z = c(2, -2)),
          epred = T) %>%
  contrast(method = 'pairwise') %>%
  gather_emmeans_draws() %>%
  summarise(PP = mean(.value<0), meanDelta = mean(.value))
```

```
## # A tibble: 1 x 3
##   contrast                                        PP meanDelta
##   <chr>                                        <dbl>     <dbl>
## 1 surprisal.lstm.Z2 - (surprisal.lstm.Z-2) 0.982     -5.71
```

S7.3 Hindi

```
hi_data.df <- readRDS('../results/hindi_mv_surprisal_0506.RDS')
```

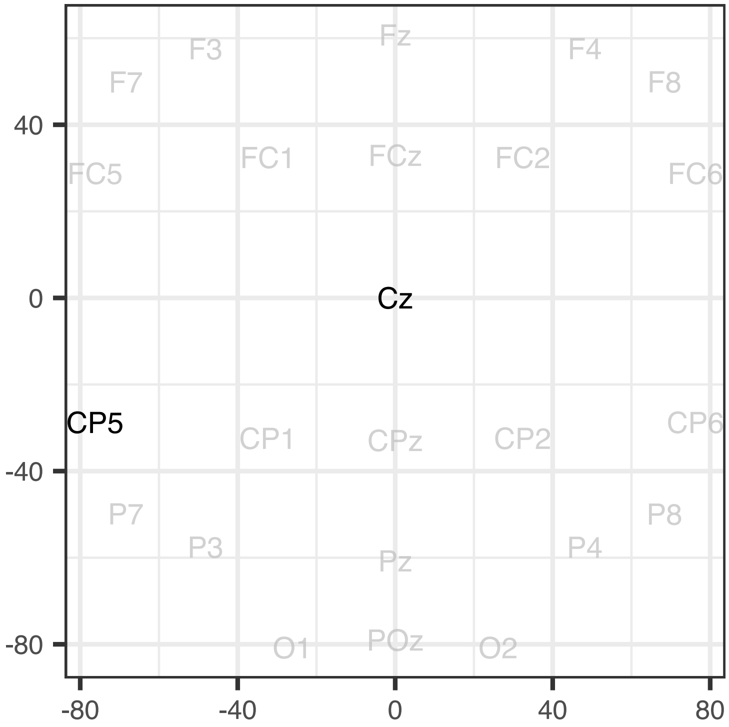

Figure S2: Electrode positions in the Hindi experiment

```
m.hi <- mV ~ surprisal.tr.Z + condition + trial_number.Z +
  (1 + condition + surprisal.tr.Z | Participant) +
  (1 + surprisal.tr.Z + condition | sent_id)

hi_tr_Cz.m <-
  brm(formula = m.hi,
      family = gaussian(),
      data = filter(hi_data.df, electrode %in% "Cz"),
      file = '../models_single_electrode/hi_tr_Cz',
      control = list(adapt_delta = 0.9),
      cores = detectCores()-2
  )

hi_tr_Cz.m %>%
  emmeans( ~ condition, # averaging over the other conditions
           epred = T) %>%
  contrast(method = 'pairwise') %>%
  gather_emmeans_draws() %>%
  summarise(PP = mean(.value<0), meanDelta = mean(.value))
```

```
## # A tibble: 1 x 3
##   contrast            PP meanDelta
```

```
##   <chr>             <dbl>     <dbl>
## 1 Abias - nonAbias 0.849    -0.489
```

```
hi_tr_CP5.m <-
  brm(formula = m.hi,
      family = gaussian(),
      data = filter(hi_data.df, electrode %in% "CP5"),
      file = '../models_single_electrode/hi_tr_CP5',
      control = list(adapt_delta = 0.9),
      cores = detectCores()-2
  )

hi_tr_CP5.m %>%
  emmeans( ~ surprisal.tr.Z,
           at = list(surprisal.tr.Z = c(2, -2)),
           epred = T) %>%
  contrast(method = 'pairwise') %>%
  gather_emmeans_draws() %>%
  summarise(PP = mean(.value<0), meanDelta = mean(.value))
```

```
## # A tibble: 1 x 3
##   contrast                                PP meanDelta
##   <chr>                                <dbl>     <dbl>
## 1 surprisal.tr.Z2 - (surprisal.tr.Z-2) 0.980     -1.65
```

S7.4 Basque

```
eu_data.df <- readRDS('../results/basque_mv_surprisal_new.RDS')
```

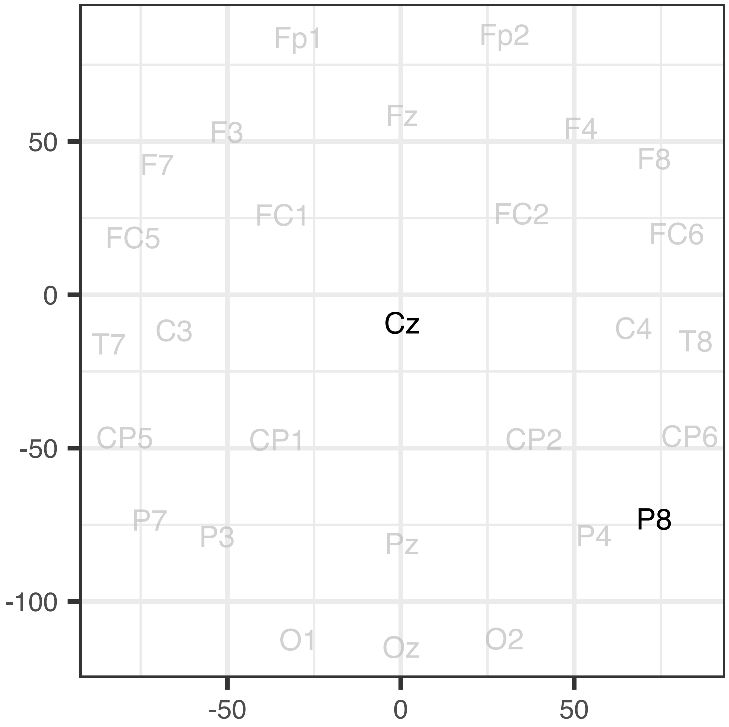

Figure S3: Electrode positions in the Basque experiment

```
m.eu <- mV ~ surprisal.tr.Z + condition + trial_number.Z +
  (1 + condition + surprisal.tr.Z | Participant) +
  (1 + surprisal.tr.Z + condition | sent_id)

eu_tr_Cz.m  <-
  brm(formula = m.eu,
      family = gaussian(),
      data = filter(eu_data.df, electrode %in% "Cz"),
      file = '../models_single_electrode/eu_tr_Cz',
      control = list(adapt_delta = 0.9),
      cores = detectCores()-2
  )

eu_tr_Cz.m  %>%
  emmeans( ~ condition, # averaging over the other conditions
           epred = T) %>%
  contrast(method = 'pairwise') %>%
  gather_emmeans_draws() %>%
  summarise(PP = mean(.value<0), meanDelta = mean(.value))
```

```
## # A tibble: 1 x 3
##   contrast             PP meanDelta
##   <chr>             <dbl>     <dbl>
## 1 Abias - nonAbias 0.972    -0.963
```

```
eu_tr_P8.m <-
  brm(formula = m.eu,
      family = gaussian(),
      data = filter(eu_data.df, electrode %in% "P8"),
      file = '../models_single_electrode/eu_tr_P8',
      control = list(adapt_delta = 0.96),
      cores = detectCores()-2
  )

eu_tr_P8.m  %>%
  emmeans( ~ surprisal.tr.Z,
           at = list(surprisal.tr.Z = c(2, -2)),
           epred = T) %>%
  contrast(method = 'pairwise') %>%
  gather_emmeans_draws() %>%
  summarise(PP = mean(.value<0), meanDelta = mean(.value))
```

```
## # A tibble: 1 x 3
##   contrast                                PP meanDelta
##   <chr>                                <dbl>     <dbl>
## 1 surprisal.tr.Z2 - (surprisal.tr.Z-2) 0.987     -1.76
```
